# Meplazumab, a CD147 antibody, for severe COVID-19: a double-blind, randomized, placebo-controlled, phase 3 clinical trial

**DOI:** 10.1038/s41392-025-02208-9

**Published:** 2025-04-14

**Authors:** Huijie Bian, Liang Chen, Zheng Zhang, Ai-Dong Wen, Zhao-Hui Zheng, Li-Qiang Song, Meng-Ying Yao, Ying-Xia Liu, Xi-Jing Zhang, Hong-Lin Dong, Jian-Qi Lian, Lei Pan, Yu Liu, Xing Gu, Hui Zhao, Jing-Wen Wang, Qing-Yi Wang, Kui Zhang, Jun-Feng Jia, Rong-Hua Xie, Xing Luo, Xiang-Hui Fu, Yan-Yan Jia, Jun-Na Hou, Qiu-Yue Tan, Xiao-Xia Chen, Liu-Qing Yang, Yuan-Long Lin, Xiao-Xia Wang, Lei Zhang, Qin-Jing Zeng, Wen-Jie Li, Rui-Xuan Wang, Yang Zhang, Xiu-Xuan Sun, Bin Wang, Xu Yang, Jian-Li Jiang, Ling Li, Jiao Wu, Xiang-Min Yang, Hai Zhang, Ying Shi, Xiao-Chun Chen, Hao Tang, Hong-Wei Shi, Shuang-Shuang Liu, Yong Yang, Tian-Yi Yang, Ding Wei, Zhi-Nan Chen, Ping Zhu

**Affiliations:** 1 Department of Cell Biology of National Translational Science Center for Molecular Medicine and Department of Clinical Immunology of Xijing Hospital, The Fourth Military Medical University, Xi’an, China; 2 State Key Laboratory of New Targets Discovery and Drug Development for Major Diseases, Xi’an, China; 3 School of Medicine, Shanghai University, Shanghai, China; 4 Department of Pharmacy, Xijing Hospital, The Fourth Military Medical University, Xi’an, China; 5 Department of Pulmonary and Critical Care Medicine, Xijing Hospital, The Fourth Military Medical University, Xi’an, China; 6 Department of Pulmonary, The First Affiliated Hospital of Zhengzhou University, Zhengzhou, China; 7 Department of Infectious Diseases, Shenzhen Third People’s Hospital, Shenzhen, China; 8 Department of Critical Care Medicine, Xijing Hospital, The Fourth Military Medical University, Xi’an, China; 9 Department of Vascular Surgery, Second Hospital of Shanxi Medical University, Taiyuan, China; 10 The Center for Diagnosis and Treatment of Infectious Diseases, Tangdu Hospital, The Fourth Military Medical University, Xi’an, China; 11 Department of Pulmonary and Critical Care Medicine, Tangdu Hospital, The Fourth Military Medical University, Xi’an, China; 12 Department of Critical Care Medicine, The First Affiliated Hospital of Xi’an Jiaotong University, Xi’an, China; 13 Department of Respiratory and Critical Care Medicine, Xi’an Chest Hospital, Xi’an, China; 14 School of Basic Medicine, The Fourth Military Medical University, Xi’an, China; 15 Department of Liver Disease, Shenzhen Third People’s Hospital, Shenzhen, China; 16 Department of Rheumatology and Immunology, Second Hospital of Shanxi Medical University, Taiyuan, China; 17 Jiangsu Pacific Meinuoke Biopharmaceutical Co. Ltd, Changzhou, China

**Keywords:** Infectious diseases, Respiratory tract diseases

## Abstract

Meplazumab, a humanized CD147 antibody, showed favorable safety and clinical benefits in phase 1 and phase 2/3 seamless clinical studies. Further evaluation of its therapeutic efficacy in patients with severe COVID-19 is needed. In this phase 3 add-on study, we randomized patients with severe COVID-19 in a 1:1 ratio to receive 0.2 mg/kg meplazumab or placebo via intravenous injection, and evaluated efficacy and safety within 56 days. Between February 2023 and November 2023, 108 patients with severe COVID-19 were randomized to two groups, with their baseline characteristics generally balanced. The primary endpoint, 28-day all-cause mortality was 1.96% in the meplazumab group vs 7.69% in the placebo group (*P* = 0.1703). Supplementary analysis using composite strategy indicated a significant reduction of 28-day all-cause mortality in meplazumab compared to placebo (3.92% vs 15.38%, *P* = 0.044). Meplazumab also significantly reduced the mortality in smoking subjects on day 28 (*P* = 0.047) compared to placebo in supplementary analysis. The secondary endpoint, 56-day all-cause mortality, was 1.96% in the meplazumab group and 11.54% in the placebo group (*P* = 0.048), which was 3.92% and 15.38%, respectively (*P* = 0.044) by supplementary analysis. Additional secondary endpoints showed potential benefits, including increased hospital discharge rates, improved clinical outcomes, and improved viral nucleotide conversion rate. Meplazumab demonstrated good safety and tolerability, with no grade ≥ 3 TEAEs observed. These promising results indicate that meplazumab reduces mortality and enhances clinical benefits in severe COVID-19 patients with a good safety profile, providing effective and specific therapeutics for severe COVID-19 (the trial was registered at ClinicalTrials.gov (NCT05679479)).

## Introduction

The COVID-19 pandemic has brought serious disruption to human society and the economy, resulting in millions of deaths and substantial morbidity worldwide. To date, the virus remains endemic in many regions with periodic recurrent waves driven by emerging dominant strains of concern, such as the JN.1 variant.^1^ As of November 2024, over 776.8 million confirmed COVID-19 cases and over 7 million confirmed deaths are reported worldwide, and it is estimated that the actual number of deaths could be 2.74 times higher than these officially reported cases.^2–5^ According to the *COVID-19 Epidemiological Update* from the World Health Organization (WHO), the weekly average of reported deaths was about 5200 in 2023 and 1400 in early 2024. Hospitalizations have reached about 40,000 per week in 2023 and 13,000 per week in 2024. Despite the significant decline in the numbers of reported deaths and hospitalizations, the ratios of deaths to hospitalizations, 0.13 in 2023 and 0.11 in 2024, remained stable. In China, a total of 9158 severe cases and 727 deaths have been reported since May 2023, with the ratio of reported deaths to severe cases hitting 0.08. The severe and critical cases are associated with a poor prognosis, elevated mortality, and long-term Covid.^6^ Therefore, clinical management of severe COVID-19 remains an urgent priority to reduce the mortality and the incidence of long COVID-19, so as to alleviate the burden on the healthcare system.^7^

The progression of severe COVID-19 is characterized by sustained and elevated viral load and cytokine storm.^8,9^ Corticosteroids, IL-6 receptor blockers, and baricitinib are WHO-recommended therapeutics for severe COVID-19, which have improved the clinical outcomes including reduced all-cause mortality and invasive mechanical ventilation by attenuating the cytokine storm.^10^ These drugs are immunomodulatory agents that target the excessive immune response triggered by SARS-CoV-2. They aim to mitigate the “cytokine storm” and the associated organ injury, ultimately reducing the risk of respiratory failure and death in patients with COVID-19. Although they exhibit clinical benefits, these medications also pose several challenges, including an increased risk of hyperglycemia and infections, and a lack of long-term outcomes.^11–13^ On the other hand, several studies reveal that high viral load combined with inflammatory markers is closely linked to the higher risk of poor prognosis in severe COVID-19.^14–16^ Therefore, antiviral treatment is indispensable in the treatment of severe COVID-19. Currently, antiviral therapies for COVID-19 are categorized into RNA-dependent RNA polymerase (RdRp) inhibitors and protease inhibitors. RdRp inhibitors, such as remdesivir and molnupiravir, target viral replication. Protease inhibitors, including nirmatrelvir and ensitrelvir, focus on the main protease (Mpro) of SARS-CoV-2, essential for viral protein processing. These therapies aim to reduce symptom severity, shorten the duration of infectiousness, and minimize hospitalizations and deaths. However, due to limitations such as inconsistent clinical efficacy, WHO conditionally recommends remdesivir for severe COVID-19 treatment only. Monoclonal antibodies are regarded as an effective strategy for treating and preventing viral infections due to their high specificity and ability to enhance the immune response.^17^ However, current neutralizing antibodies, primarily targeting the S1 subunit’s RBD of SARS-CoV-2 spike protein, are increasingly ineffective against emerging variants due to the hotspot mutations in RBD. These variants, such as Omicron, have reduced the potency of neutralizing antibody therapies like REGEN-COV and sotrovimab. Despite the relative conservation of the S2 subunit of the SARS-CoV-2 spike protein, current S2-targeting antibodies face several challenges, including weaker neutralizing activity, lower immunogenicity, and complexities in antibody production.^18^ The development of antiviral drugs must focus on new strategies, such as host-targeting antivirals, to maintain effectiveness against the ongoing viral evolution.^19^

Previously, we revealed that CD147, a type I transmembrane receptor, mediates a novel route for SARS-CoV-2 and its variants to infect host cells by interacting with the spike protein via ADP-ribosylation factor 6-dependent endocytosis.^20,21^ CD147 was found to regulate the expression of cytokines and chemokines by “spike protein-CD147-CyPA signaling axis”, triggering cytokine storm.^22,23^ We also identified CD147 as a crucial regulator for fibroblast activation in the SARS-CoV-2-induced pulmonary fibrogenesis.^24^ We developed a humanized antibody, meplazumab, acting as a CD147 blocker to inhibit entry of the virus and its variants. Meplazumab is different from viral protein-targeted antibodies whose neutralizing activity is compromised by mutations in the spike protein. In the phase 1 clinical trial (NCT04369586), meplazumab showed a favorable safety profile in healthy volunteers.^25^ We then conducted a randomized, double-blind, placebo-controlled phase 2/3 seamless trial in hospitalized patients with severe COVID-19 (NCT04586153), and the meplazumab displayed clinical benefits, including decreased mortality and improved live discharge without oxygen supplement with a good safety profile.^26^

To validate these findings, we conducted a multicenter, double-blind, randomized, placebo-controlled phase 3 add-on trial to evaluate the efficacy and safety of meplazumab in patients with severe COVID-19 (NCT05679479). The 28-day all-cause mortality, as well as 14- and 56-day all-cause mortality, discharge rates, and the incidence of adverse reactions between meplazumab and placebo were compared, and the safety was assessed.

## Results

### Baseline characteristics

**Fig. 1:**
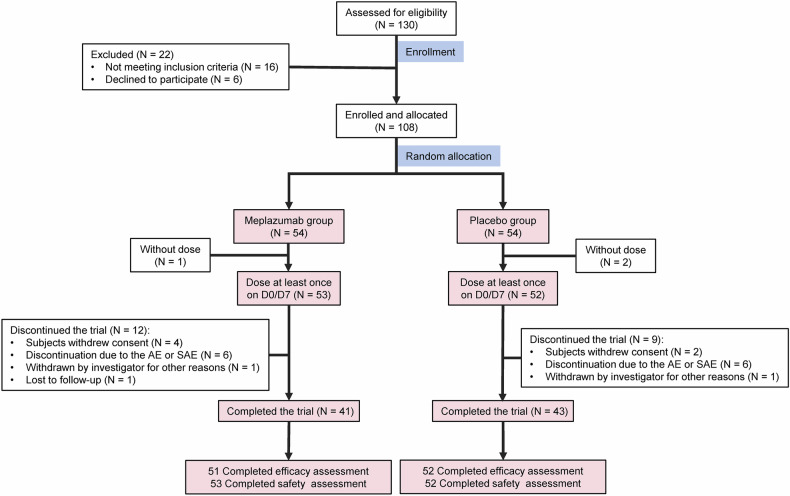
Enrollment and trial design. Flowchart depicting the enrollment, allocation, and exclusion process of study participants. AE adverse event, SAE serious adverse event

From February 2023 to November 2023, a total of 130 patients underwent screening at eight clinical research centers in China. Among them, 108 patients were randomized to the meplazumab group or placebo group in a 1:1 ratio. A total of 103 patients were included in the intention-to-treat population, and 105 were included in the safety analysis population. In the meplazumab group, 53 subjects received the first dose, among whom 27 subjects received the second dose. In the placebo group, 52 subjects received the first dose, among whom 29 subjects received the second dose. The ratio of received two doses in two groups was balanced (*P* = 0.56). The 28-day follow-up evaluation was completed in 105 patients (Fig. 1).

The median age of the meplazumab group and placebo group was 68.2 and 69.7 years old, respectively. Thirty-two patients (31.1%) were female. All patients had severe COVID-19. A total of 45 patients (43.7%) had received COVID-19 vaccination prior to enrollment. The baseline demographic and clinical characteristics were balanced between the two groups except for “smoking” (Table 1). The comorbidities and medical history were also balanced between the two groups except for “coronary atherosclerosis” and “renal failure” (supplementary Table 1). All patients received standard of care according to *the Diagnosis and Treatment Protocol for Novel Coronavirus Pneumonia (Tentative 10th Edition)*, and the concomitant medications, including the details of concomitant use of antiviral agents, glucocorticoids, anticoagulants, and oxygen therapy are listed in the Supplementary Tables 2–4.

**Table 1:** Baseline demographic and clinical characteristics^a^

	Meplazumab (*N* = 51)	Placebo (*N* = 52)	Statistics	*P*-value
Age				
<65 years, *n* (%)	15 (29.41)	15 (28.85)	Chi-square test	0.9496
≥65 years, *n* (%)	36 (70.59)	37 (71.15)		
Total (missing)	51 (0)	52 (0)		
Gender				
Male, *n* (%)	38 (74.51)	33 (63.46)	Chi-square test	0.2257
Female, *n* (%)	13 (25.49)	19 (36.54)		
Total (Missing)	51 (0)	52 (0)		
Baseline BMI ≥ 30 kg/m^2^				
Yes, *n* (%)	6 (13.04)	2 (4.65)	Fisher’s exact test	0.2684
No, *n* (%)	40 (86.96)	41 (95.35)		
Total (missing)	46 (5)	43 (9)		
Comorbidities				
Yes, *n* (%)	49 (96.08)	48 (92.31)	Fisher’s exact test	0.6781
No, *n* (%)	2 (3.92)	4 (7.69)		
Total (missing)		52 (0)		
SARS-CoV-2 vaccination status				
Vaccinated, *n* (%)	24 (48.98)	21 (41.18)	Chi-square test	0.4330
Unvaccinated, *n* (%)	25 (51.02)	30 (58.82)		
Total (missing)	49 (2)	51 (1)		
Duration of illness (days)^b^				
*N* (missing)	51 (0)	52 (0)		
Mean (SD)	6.1 (7.9)	5.8 (8.2)	Independent samples *t*-test	0.8173
Median	3.0	2.0		
Q1, Q3	1.0, 8.0	2.0, 7.0		
Min, Max	0, 40	0, 50		
Smoking				
Yes, *n* (%)	21 (42.00)	12 (23.53)	Chi-square test	0.0478
No, *n* (%)	29 (58.00)	39 (76.47)		
Total (missing)	50 (1)	51 (1)		
Concomitant antiviral medication				
Yes, *n* (%)	6 (11.76)	7 (13.46)	Chi-square test	0.7954
No, *n* (%)	45 (88.24)	45 (86.54)		
Total (missing)	51 (0)	52 (0)		

^a^Baseline is defined as the last non-missing observation before the first treatment

^b^Duration of illness (days) = Date of informed consent signed - Date of COVID-19 diagnosis + 1

### Clinical efficacy

On day 28, 1 patient (1.96%, 95% CI: 0.05, 10.45) in the meplazumab group, and 4 (7.69%, 95% CI: 2.14, 18.54) in the placebo group had died. A decreased all-cause mortality was observed in the meplazumab group compared to the placebo group, with the difference not being statistically significant (*P* = 0.170) and the rate difference at 5.72% (95% CI: −2.46, 13.90).

The supplementary analysis using composite strategy showed that on day 28, the all-cause mortality was 3.92% (95% CI: 0.48, 13.46) in the meplazumab group and 15.38% (95% CI: 6.88, 28.08) in the placebo group (*P* = 0.044), and the rate difference between the two groups was 11.44% (95% CI: 0.30, 22.58) with a relatively lower rate of 74.5% in the meplazumab (Table 2). Furthermore, subgroup analyses of the primary efficacy endpoints based on smoking showed that the all-cause mortality on day 28 was 4.76% (95% CI: 0.12, 23.82) in the meplazumab group and 33.33% (95% CI: 9.92, 65.11) in the placebo group (*P* = 0.047, Supplementary Table 5) with the rate difference of 28.57% (95% CI: 2.88, 57.59).

**Table 2:** All-cause mortality by day 56

Characteristics		Meplazumab (*N* = 51)		Placebo (*N* = 52)
All-cause mortality on day 28				
*n* (%)		1 (1.96)		4 (7.69)
95% CI^a^		0.05, 10.45		2.14, 18.54
Rate difference (%, 95% CI)^b^		5.72 (−2.46, 13.90)		
OR (95% CI)^c^		0.24 (0.03, 2.23)		
*P*-value		0.170		
Relatively decreased mortality by meplazumab^d^		75.4%		
All-cause mortality on day 28 (Supplementary analysis)^e^				
*n* (%)		2 (3.92)		8 (15.38)
95% CI^a^		0.48, 13.46		6.88, 28.08
Rate difference (%, 95% CI)^b^		11.44 (0.30, 22.58)		
OR (95% CI)^c^		0.22 (0.04, 1.11)		
*P*-value		0.044		
Relatively decreased mortality by meplazumab^d^		74.5%		
All-cause mortality on day 14				
*n* (%)		0 (0.00)	2 (3.85)	
95% CI^a^		0.00, 6.98	0.47, 13.21	
rate difference (%, 95% CI)^b^		3.83 (−1.39, 9.05)		
OR (95% CI)^c^				
*P*-value		0.150		
Relatively decreased mortality by meplazumab^d^		100%		
All-cause mortality on day 14 (Supplementary analysis)^e^				
*n* (%)		1 (1.96)	7 (13.46)	
95% CI^a^		0.05, 10.45	5.59, 25.79	
Rate difference (%, 95% CI)^b^		11.49 (1.46, 21.52)		
OR (95% CI)^c^		0.13 (0.02, 1.09)		
*P*-value		0.025		
Relatively decreased mortality by meplazumab^d^		85.4%		
All-cause mortality on day 56				
*n* (%)		1 (1.96)		6 (11.54)
95% CI^a^		0.05, 10.45		4.35, 23.44
Rate difference (%, 95% CI)^b^		9.58 (0.08, 19.07)		
OR (95% CI)^c^		0.15 (0.02, 1.32)		
*P*-value		0.048		
Relatively decreased mortality by meplazumab^d^		83.0%		
All-cause mortality on day 56 (Supplementary analysis)^e^				
*n* (%)		2 (3.92)		8 (15.38)
95% CI^a^		0.48, 13.46		6.88, 28.08
Rate difference (%, 95% CI)^b^		11.44 (0.30, 22.58)		
OR (95% CI)^c^		0.22 (0.04, 1.11)		
*P*-value		0.044		
Relatively decreased mortality by meplazumab^d^		74.5%		

^a^The 95% confidence interval (CI) is estimated using the Clopper–Pearson method

^b^The rate difference and its 95% CI are estimated using the Cochran–Mantel–Haenszel method, adjusting for stratification factors (age groups: <65 years vs ≥65 years)

^c^Odds ratio (OR) is estimated to use the logistic regression model, with the all-cause mortality being the dependent variable, and the treatment groups (meplazumab and placebo) and age groups (<65 years and ≥65 years) being the fixed effects

^d^The relatively decreased mortality by meplazumab = [Mortality of placebo group − Mortality of meplazumab group)/Mortality of placebo group] × 100%

^e^The supplementary analysis is defined as follows: based on FAS, efficacy evaluation is performed with intercurrent events “early discontinuation of treatment due to poor efficacy”, “concomitant use of prohibited medications/treatments affecting efficacy evaluation”, and “disease aggravation” analyzed as “death” according to the composite strategy

The short-term efficacy of the meplazumab was evaluated on day 14, where the all-cause mortality was 0% (95% CI: 0.00, 6.98) in the meplazumab group and 3.85% (95% CI: 0.47, 13.21) in the placebo group (*P* = 0.150) with rate difference 3.83% (95% CI: −1.39%, 9.05%). The supplementary analysis showed that all-cause mortality on day 14 was 1.96% (95% CI: 0.05, 10.45) in the meplazumab group and 13.46% (95% CI: 5.59, 25.79) in the placebo group, with a rate difference of 11.49% (95% CI: 1.46, 21.52), and the relatively decreased rate was 85.4% in the meplazumab group (*P* = 0.025, Table 2).

The long-term therapeutic effect was assessed using the all-cause mortality on day 56, which was 1.96% (95% CI: 0.05, 10.45) in the meplazumab group and 11.54% (95% CI: 4.35, 23.44) in the placebo group, with a rate difference of 9.58% (95% CI: 0.08, 19.07), and the relatively decreased rate was 83.0% in the meplazumab group (*P* = 0.048, Table 2). The supplementary analysis showed that all-cause mortality in the meplazumab group and placebo group was 3.92% (95% CI: 0.48, 13.46) and 15.38% (95% CI: 6.88, 28.08), respectively, with a rate difference of 11.44% (95% CI: 0.30, 22.58), and the relatively decreased rate was 74.5% in the meplazumab group (*P* = 0.044, Table 2).

The assessment of other secondary endpoints also indicated potential benefits for patients treated with meplazumab, including a 5.58% increase in the discharge rate on day 28 in the meplazumab group compared to the placebo group (90.20% vs 84.62%). Additionally, the rate of sustained clinical improvement by day 28 was increased by 7.47% in the meplazumab group compared to the placebo group (88.24% vs 80.77%).

The meplazumab group had 27 subjects (52.94%) admitted to the ICU, with a median ICU stay of 11 days, whereas the placebo group had 31 cases (59.62%) admitted to the ICU, with a median ICU stay of 12 days. This indicates that the meplazumab group had a 6.68% lower rate of ICU admission than the placebo group.

Viral load is associated with the body’s immune response, disease severity, and mortality in COVID-19.^27,28^ In this study, the nucleic acid-negative conversion rate and the nucleic acid-negative conversion time were analyzed using the Kaplan–Meier method. As shown in supplementary Table 6, from day 7 to day 56, the nucleic acid-negative conversion rates at various time points were consistently higher in the meplazumab group than in the placebo group. Particularly, the nucleic acid-negative conversion rate on day 7 was improved by 11.69% in the meplazumab group compared to the placebo group (57.14% vs 45.45%). The median time to nucleic acid-negative conversion in the meplazumab group was 7 days, compared to 9 days in the placebo group. The viral genomic copy number was evaluated using the cycle threshold values of the ORF1ab gene of SARS-CoV-2. As shown in Supplementary Table 7, the cycle threshold changes from baseline were higher in the meplazumab group than in the placebo group, indicating the viral load was reduced by meplazumab.

### Safety

In this study, 0.2 mg/kg meplazumab demonstrated good safety and tolerability in patients with severe COVID-19. Most adverse events were consistent with the reported complications in COVID-19, and the majority of adverse events were considered not associated with meplazumab by the investigators. Drug-related adverse events were observed in 6 patients (11.32%) in the meplazumab group and 5 patients (9.62%) in the placebo group. The meplazumab group had 1 patient reporting an adverse event (fever) leading to drug discontinuation (Table 3 and Supplementary Tables 8 and 9). No grade ≥ 3 adverse reactions were observed.

**Table 3:** Summary of treatment-emergent adverse events through day 56^*^

Adverse event	Meplazumab (*N* = 53)		Placebo (*N* = 52)		Total (*N* = 105)	
	Frequency	*N* (%)	Frequency	*N* (%)	Frequency	*N* (%)
**Total**	160	43 (81.13)	124	40 (76.92)	284	83 (79.05)
AE related to the investigation drug^§^	7	6 (11.32)	6	5 (9.62)	13	11 (10.48)
AE with grade ≥ 3	12	12 (22.64)	25	15 (28.85)	37	27 (25.71)
Serious adverse events	10	10 (18.87)	22	13 (25.00)	32	23 (21.90)
Significant adverse events	9	6 (11.32)	7	5 (9.62)	16	11 (10.48)
AE leading to withdrawal from the study	6	6 (11.32)	9	6 (11.54)	15	12 (11.43)
AE leading to death	3	3 (5.66)	6	6 (11.54)	9	9 (8.57)
AE leading to discontinuation of the medication	3	3 (5.66)	1	1 (1.92)	4	4 (3.81)
AE leading to discontinuation and related to the drug	1	1 (1.89)	0	0 (0.00)	1	1 (0.95)
AE leading to dose reduction	1	1 (1.89)	2	2 (3.85)	3	3 (2.86)

^*^Adverse events (AE) are coded using MedDRA version 27.0

^§^AE related to the investigation drug are classified as “related”, “likely related”, or “possibly related”

## Discussion

In this phase 3 clinical trial, we examined the efficacy of meplazumab plus standard of care (named “add-on”) in the treatment of patients with severe COVID-19 who might develop acute respiratory distress syndrome leading to death due to the lack of specific drugs. We assessed the effects of intervention with host cell receptor CD147-targeted antibody therapy on all-cause mortality, discharge rate, sustained clinical improvement, ICU stay time, and nucleic acid-negative conversion. Our study showed that meplazumab treatment significantly reduced the all-cause mortality on day 14, day 28, and day 56 post-treatment with relatively decreased mortality ranging from 74.5% to 85.4%. These results were consistent with our previous phase 2/3 seamless clinical trial, which evaluated meplazumab in hospitalized patients with severe COVID-19. Notably, meplazumab resulted in decreased mortality in the early stages of the treatment, which would alleviate the multi-organ lesion, and improve the prognosis of severe cases. A significant relation was observed between smoking and the severity of COVID-19.^29^ Smokers had an increased risk of more severe outcomes from the disease, including higher hospitalization rates, increased ICU admission, higher rates of mechanical ventilation, and increased mortality.^29^ In this study, we also observed a higher mortality among COVID-19 patients with smoking history and found that the treatment with meplazumab could reduce the mortality in smoking subjects significantly. Consistent with the reduced viral load observed in our two previous clinical trials, meplazumab resulted in rapid reductions in the SARS-CoV-2 viral load on day 7 with a relatively improved nucleic acid-negative conversion rate of 28.2%. The results reflect the pharmacological effects of meplazumab on inhibiting the viral infection through the blockage of CD147. Meplazumab, as a receptor blocker, exhibits favorable antiviral effects by depressing persistent infections and high viral loads, which are commonly responsible for multiple organ failure and immune system compromise. The incidence of drug-related adverse events was similar between the meplazumab group (11.32%) and the placebo group (9.62%); no serious TEAEs were observed in either group. This finding suggests that meplazumab has a favorable safety profile in severe COVID-19 populations. The overall safety profile was consistent with that observed in our previous reports.^26^ We found that meplazumab treatment can reduce the mortality rate in patients with severe COVID-19 and offer multiple potential benefits while maintaining a favorable safety profile.^25,26^ Our study reinforced the effectiveness of this novel strategy using host receptor-targeted antibody therapy for severe COVID-19.

In this study, a composite strategy was planned ahead and employed for the supplementary analysis to complement and expand the main analysis results.^30^ Several concomitant events, including “discontinuation of treatment due to poor efficacy”, “concomitant use of prohibited drugs/therapies that affect efficacy evaluation”, and “disease progression” were combined into a composite variable and counted as “death”. These concomitant events were considered indicative of the ineffectiveness of the investigational drug and classified as treatment failure. Since the primary efficacy endpoint in this study is all-cause mortality, treatment failure should be calculated as “death” according to the composite strategy. For instance, cases of “disease progression” suggest that the drug treatment may be ineffective. For severe cases of COVID-19, “disease progression” could potentially lead to death by 56 days. Therefore, the supplementary analysis in this study provided a more objective and comprehensive evaluation of the meplazumab’s efficacy.

The treatment of severe COVID-19 involves a multiple approach targeting the hyperinflammatory response and immune dysregulation characteristic of the disease. Immune response regulators, including tocilizumab and baricitinib, have emerged as key therapeutic agents for severe COVID-19 by targeting cytokine storm and hyperinflammation. Corticosteroids, such as dexamethasone and methylprednisolone, are widely used to suppress inflammation to reduce mortality in severe cases. However, their application in the treatment of severe COVID-19 is somewhat restricted due to several potential side effects. Antiviral treatment is the most essential for severe COVID-19, as the virus proliferation in severe cases can lead to increased viral load and multiple organ failure. The emerging viral variants continue to undermine therapeutic efficacy, highlighting the need for ongoing research and adaptation of treatment strategies.^31^ Several antiviral agents, including viral RNA polymerase inhibitors, convalescent plasma, and neutralizing monoclonal antibodies show promise in clinical trials. Convalescent plasma therapy, while initially promising, has shown mixed results, with some studies reporting reduced 7-day mortality but no long-term benefits.^32^ Neutralizing antibodies like casirivimab/imdevimab and sotrovimab have shown significant efficacy in reducing hospitalization and mortality. However, challenges remain, including the emergence of viral variants that evade neutralization, the need for early administration, and the risk of adverse effects such as antibody-dependent enhancement.^31^ Targeting host receptors is an ideal strategy for developing broad-spectrum antiviral antibodies. This approach leverages the stability of host receptors, reducing the risk of viral escape mutations. Antibodies like DAS181, which target host sialic acid receptors, have shown efficacy against multiple respiratory viruses, including influenza A.^33^ In COVID-19 treatment, this host-targeting strategy holds promise for overcoming viral resistance and providing durable therapeutic benefits. CD147, recognized as a universal receptor for the spike protein of SARS-CoV-2 and its variants, plays a pivotal role in facilitating viral infection.^20^ Meplazumab, an antibody targeting CD147, has demonstrated the capability to effectively block viral entry and replication, which is not compromised by the variants of SARS-CoV-2.^22^

The trial has several limitations. The number of patients enrolled in this trial was less than the estimated sample size due to the rapid shrink of COVID-19 cases during the study period. While several secondary outcomes, including discharge rate, sustained clinical improvement rate, ICU admission rate, and viral load displayed better trends in the meplazumab group than in the placebo group, no statistically significant difference was observed. Additionally, factors such as the timing of previous vaccination or prior infection and the variants of SARS-CoV-2 were not recorded in the analysis, which may have affected the balance between the two groups. In conclusion, meplazumab demonstrates promising results in reducing mortality and enhancing clinical benefits in patients with severe COVID-19. The incidence of adverse events in the meplazumab group is comparable to that of the placebo group, highlighting its good safety profile for severe COVID-19. Therefore, our study provides an effective and specific therapeutic strategy for managing severe COVID-19.

## Materials and methods

### Study design and participants

This was phase 3, a double-blind, randomized, placebo-controlled, add-on study. The objective was to assess the safety and efficacy of meplazumab in the treatment of severe COVID-19. Eligible participants were adults at least 18-years-old and diagnosed with severe COVID-19 according to *the Diagnosis and Treatment Protocol for Novel Coronavirus Pneumonia (Tentative 10th Edition)*.^34^

The investigators reviewed the medical history, symptoms, risk factors, and inclusion and exclusion criteria. Concomitant medications were recorded. Written informed consents were obtained from all patients or their legal representatives. The full list of inclusion and exclusion criteria is provided in the protocol (Supplementary Appendix).

The trial was conducted in accordance with ethical principles and local laws and regulations. The protocol and related documents were reviewed and approved by the local institutional review board or ethics committee at eight trial centers (approve numbers: YS20221086-1, 202301-02, 202302-PJ01, 2023-009-04, L2023-Y147, [2023]YW-056, X-XJTU1AF2023LSY-135, and 023-002-01). Additional details regarding the trial design are provided in the protocol (Supplementary Appendix).

### Randomization and masking

All investigation staff were blinded when making decisions related to the study until the trial was completed. The baseline characteristics were collected, including demographic characteristics, smoking history, medical history of COVID-19, vital signs, physical examination, and laboratory examination. Eligible hospitalized patients were randomized to the meplazumab group or placebo group in a 1:1 ratio using an interactive web response system (IWRS) by stratified blocked randomization (block size = 4). The stratification factor was age (age < 65-years-old or ≥65-years-old). All patients received standard of care according to *the Diagnosis and Treatment Protocol for Novel Coronavirus Pneumonia (Tentative 10th* *Edition)*.^34^ The solutions of meplazumab and placebo were indistinguishable, prepared by an unblinded third party, and administered by an authorized and blinded staff member at each site.

### Procedures

**Fig. 2:**
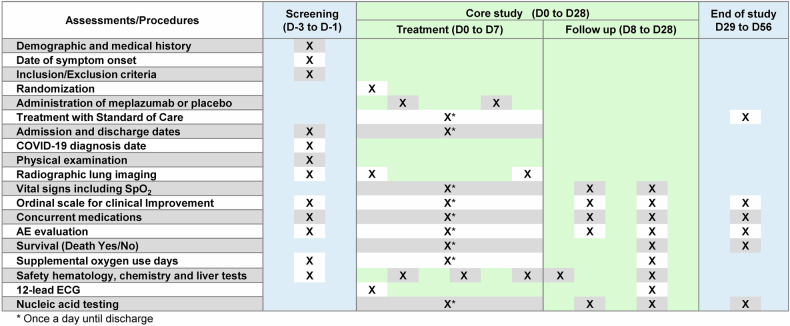
Timeline of study procedures and key protocol steps. Schematic of the study design representing the timeline and the key assessments

A double dose of 0.2 mg/kg meplazumab or placebo was administered on day 1 and day 7. On the day of infusion, meplazumab (diluted in 0.9% saline) or placebo (0.9% saline) was administered intravenously. The dose of meplazumab was determined based on the data from the phase 1 clinical trial in healthy patients (NCT04369586) and the phase 2/3 clinical trial in hospitalized patients with severe COVID-19 (NCT04586153).^25,26^ The safety assessments were performed between day -3 and day 56, and the efficacy assessments were performed between day -1 and day 56. The schedule of assessments is described in the protocol (Fig. 2, Supplementary Appendix).

### Outcomes

The primary objective of the trial was to compare all-cause mortality on day 28 with meplazumab vs placebo. The secondary endpoints included discharge rate on day 28, mortality on day 14 and day 56, time to sustained clinical improvement, duration of oxygen therapy (days), duration of mechanical ventilation (days), incidence and duration of reintubation within 24 h after extubation from mechanical ventilation (days), ICU and hospital stay duration (days), time to SARS-CoV-2 nucleic acid negativity, SARS-CoV-2 nucleic acid negativity rate, and change in SARS-CoV-2 viral load from baseline by day 7, day 14, and day 28. All efficacy endpoints were evaluated in the participants who were randomized into groups and received at least one dose of meplazumab or placebo.

To assess the safety profile of meplazumab, the incidence of adverse events that occurred during the treatment, including serious adverse events and adverse events that led to discontinuation of meplazumab or placebo, were collected by day 56. The safety data were evaluated in the safety analysis population, which included all participants who were randomized and received at least one dose of meplazumab or placebo.

### Statistical analysis

The sample size was calculated by assuming a mortality of 5% in the meplazumab group and 15% in the placebo group, with a significance level of α = 0.025 (one-sided) and a 20% dropout rate based on the results of exploratory phase 2 (NCT04275245) and phase 2/3 clinical trials (NCT04586153).^25,26^

The all-cause mortality on day 28 was calculated for the meplazumab group and the placebo group, respectively. The two-sided 95% confidence interval (CI) was identified using the Clopper–Pearson method. A stratified Cochran–Mantel–Haenszel (CMH) chi-square test was performed to assess the differences between the groups, adjusting for stratification factors (age groups: <65 years vs ≥65 years). The rate difference in all-cause mortality on day 28 was calculated and its two-sided 95% CI was determined using the CMH method, considering the stratification factors (age groups: <65 years vs ≥65 years). A supplementary analysis was performed using a multivariate strategy, where concomitant events including “discontinuation of treatment due to poor efficacy”, “concomitant use of prohibited drugs/therapies that affect efficacy evaluation”, and “disease progression” were combined into a composite variable and counted as “death”, as described in the study protocol and SAP (Supplementary Appendix).^30^

To perform a sensitivity analysis on missing data, the tipping point analysis (TPA) method was employed to evaluate the robustness influence of missing data on the study results. Additional details on sample size calculations and statistical analysis are provided in the Statistical Analysis Plan (Supplementary Appendix).

## Supplementary information

Supplemental Data

Protocol and SAP

## Data Availability

The protocol, consent form, statistical analysis plan, regulatory documents, and other relevant study materials are available online. The data that supports the findings of this study are available from the corresponding authors upon reasonable request. Participant data without names and identifiers will be made available after approval from all corresponding authors. After the publication of the study findings, the data will be available for others to request. The research team will provide an email address for communication once the data is approved to be shared with others. A proposal with a detailed description of study objectives and a statistical analysis plan will be needed for the evaluation of the reasonability to request for our data. The corresponding authors will make a decision based on these materials. Additional materials may also be required during the process.
