# Supplementary material for: Meplazumab, a CD147 antibody, for severe COVID-19: a double-blind, randomized, placebo-controlled, phase 3 clinical trial
Source: Signal Transduct Target Ther. 2025 Apr 14;10:119. doi: 10.1038/s41392-025-02208-9 (PMC11994814; doi:10.1038/s41392-025-02208-9)
Supplement: Supplementary file 1 — Supplemental Data [file 41392_2025_2208_MOESM1_ESM.docx]

**Supplementary Materials for**

**Meplazumab, a CD147 antibody, for severe COVID-19: a double-blind, randomized, placebo-controlled, phase 3 clinical trial**

Huijie Bian^*#^, Liang Chen^*^, Zheng Zhang^*^, Ai-Dong Wen^*^, Zhao-Hui Zheng^*^, Li-Qiang Song^*^ Meng-Ying Yao^*^, Ying-Xia Liu^*^, Xi-Jing Zhang^*^, Hong-Lin Dong^*^, Jian-Qi Lian^*^, Lei Pan^*^, Yu Liu^*^, Xing Gu^*^, Hui Zhao^*^, Jing-Wen Wang^*^, Qing-Yi Wang^*^, Kui Zhang, Jun-Feng Jia, Rong-Hua Xie, Xing Luo, Xiang-Hui Fu, Yan-Yan Jia, Jun-Na Hou, Qiu-Yue Tan, Xiao-Xia Chen, Liu-Qing Yang, Yuan-Long Lin, Xiao-Xia Wang, Lei Zhang, Qin-Jing Zeng, Wen-Jie Li, Rui-Xuan Wang, Yang Zhang, Xiu-Xuan Sun, Bin Wang, Xu Yang, Jian-Li Jiang, Ling Li, Jiao Wu, Xiang-Min Yang, Hai Zhang, Ying Shi, Xiao-Chun Chen, Hao Tang, Hong-Wei Shi, Shuang-Shuang Liu, Yong Yang, Tian-Yi Yang, Ding Wei^#^, Zhi-Nan Chen^#^, Ping Zhu^#^

Correspondence: Ping Zhu (zhuping@fmmu.edu.cn), Zhi-Nan Chen (znchen@fmmu.edu.cn), Huijie Bian (hjbian@fmmu.edu.cn), and Ding Wei (weidcq@fmmu.edu.cn)

**This file includes:**

Supplementary Tables 1 to 9

| **Table 1** **Comorbidities and medical history**§ | | | | | | | |
| --- | --- | --- | --- | --- | --- | --- | --- |
| **System organ class**  **Preferred term** | **Meplazumab (N=51)** | | | **Placebo (N=52)** | | | **P-value*** |
|  | **Frequency** | **n** | **Incidence (%)** | **Frequency** | **n** | **Incidence (%)** |  |
| **Total** | 116 | 46 | 90.20 | 81 | 41 | 78.85 | 0.1729 |
| **Vascular and lymphatic disorders** | 30 | 29 | 56.86 | 29 | 28 | 53.85 | 0.8436 |
| Hypertension | 30 | 29 | 56.86 | 29 | 28 | 53.85 | 0.8436 |
| **Cardiac disorders** | 34 | 24 | 47.06 | 15 | 13 | 25.00 | 0.0245 |
| Coronary atherosclerosis | 15 | 15 | 29.41 | 5 | 5 | 9.62 | 0.0133 |
| Heart failure | 8 | 8 | 15.69 | 3 | 3 | 5.77 | 0.1222 |
| Coronary heart disease | 7 | 7 | 13.73 | 6 | 6 | 11.54 | 0.7749 |
| Angina pectoris | 2 | 2 | 3.92 | 0 | 0 | 0.00 | 0.2427 |
| Myocardial infarction | 1 | 1 | 1.96 | 1 | 1 | 1.92 | 1.0000 |
| Pulmonary heart disease | 1 | 1 | 1.96 | 0 | 0 | 0.00 | 0.4951 |
| **Metabolic and nutritional disorders** | 21 | 21 | 41.18 | 17 | 17 | 32.69 | 0.4180 |
| Type 2 diabetes mellitus | 12 | 12 | 23.53 | 8 | 8 | 15.38 | 0.3287 |
| Diabetes mellitus | 9 | 9 | 17.65 | 9 | 9 | 17.31 | 1.0000 |
| **Respiratory, thoracic and mediastinal disorders** | 10 | 10 | 19.61 | 7 | 7 | 13.46 | 0.4375 |
| Chronic obstructive pulmonary disease (COPD) | 3 | 3 | 5.88 | 3 | 3 | 5.77 | 1.0000 |
| Emphysema | 3 | 3 | 5.88 | 2 | 2 | 3.85 | 0.6781 |
| Asthma | 1 | 1 | 1.96 | 1 | 1 | 1.92 | 1.0000 |
| Chronic bronchitis | 1 | 1 | 1.96 | 1 | 1 | 1.92 | 1.0000 |
| Bronchiectasis | 1 | 1 | 1.96 | 0 | 0 | 0.00 | 0.4951 |
| Pulmonary embolism | 1 | 1 | 1.96 | 0 | 0 | 0.00 | 0.4951 |
| **Nervous system disorders** | 7 | 7 | 13.73 | 8 | 7 | 13.46 | 1.0000 |
| Cerebral infarction | 4 | 4 | 7.84 | 5 | 5 | 9.62 | 1.0000 |
| Lacunar infarction | 2 | 2 | 3.92 | 1 | 1 | 1.92 | 0.6176 |
| Embolic cerebral infarction | 1 | 1 | 1.96 | 1 | 1 | 1.92 | 1.0000 |
| Cerebral hemorrhage | 0 | 0 | 0.00 | 1 | 1 | 1.92 | 1.0000 |
| **Renal and urinary disorders** | 7 | 7 | 13.73 | 0 | 0 | 0.00 | 0.0058 |
| Renal failure | 5 | 5 | 9.80 | 0 | 0 | 0.00 | 0.0268 |
| Chronic kidney disease | 1 | 1 | 1.96 | 0 | 0 | 0.00 | 0.4951 |
| Diabetic nephropathy | 1 | 1 | 1.96 | 0 | 0 | 0.00 | 0.4951 |
| Infections and infestations | 5 | 5 | 9.80 | 1 | 1 | 1.92 | 0.1123 |
| Hepatitis C | 3 | 3 | 5.88 | 0 | 0 | 0.00 | 0.1178 |
| Chronic hepatitis B | 2 | 2 | 3.92 | 1 | 1 | 1.92 | 0.6176 |
| **Neoplasms benign, malignant and unspecified (including cysts and polyps)** | 2 | 2 | 3.92 | 2 | 2 | 3.85 | 1.0000 |
| Plasma cell myeloma | 1 | 1 | 1.96 | 0 | 0 | 0.00 | 0.4951 |
| Malignant neoplasm of lung | 1 | 1 | 1.96 | 1 | 1 | 1.92 | 1.0000 |
| Bladder cancer | 0 | 0 | 0.00 | 1 | 1 | 1.92 | 1.0000 |
| **Congenital, familial and genetic disorders** | 0 | 0 | 0.00 | 1 | 1 | 1.92 | 1.0000 |
| Congenital heart disease | 0 | 0 | 0.00 | 1 | 1 | 1.92 | 1.0000 |
| **Hepatobiliary disorders** | 0 | 0 | 0.00 | 1 | 1 | 1.92 | 1.0000 |
| Liver cirrhosis | 0 | 0 | 0.00 | 1 | 1 | 1.92 | 1.0000 |
| § Comorbidities and medical history were coded using MedDRA version 27.0.  *P-values were calculated using Fisher's exact test. | | | | | | | |

| **Table 2 Concomitant medications*** | | | | | | |
| --- | --- | --- | --- | --- | --- | --- |
| **ATCII Names** | **Meplazumab (N=53)** | | | **Placebo (N=52)** | | |
|  | **Frequency** | **n** | **Incidence (%)** | **Frequency** | **n** | **Incidence (%)** |
| Cough and cold preparations | 101 | 44 | 83.02 | 93 | 41 | 78.85 |
| Antithrombotic agents | 98 | 44 | 83.02 | 103 | 49 | 94.23 |
| Drugs for obstructive airway disease | 132 | 43 | 81.13 | 120 | 41 | 78.85 |
| Corticosteroids for systemic use | 113 | 42 | 79.25 | 115 | 46 | 88.46 |
| Antibacterials for systemic use | 112 | 39 | 73.58 | 108 | 42 | 80.77 |
| Other respiratory system products | 64 | 39 | 73.58 | 67 | 41 | 78.85 |
| Blood substitutes and prefusion solutions | 115 | 39 | 73.58 | 101 | 35 | 67.31 |
| Drugs for acid related disorders | 72 | 35 | 66.04 | 75 | 38 | 73.08 |
| Mineral supplements | 185 | 35 | 66.04 | 153 | 41 | 78.85 |
| Drugs used in diabetes | 223 | 33 | 62.26 | 174 | 31 | 59.62 |
| Bile and liver therapy | 48 | 28 | 52.83 | 32 | 20 | 38.46 |
| Immunostimulants | 52 | 26 | 49.06 | 37 | 27 | 51.92 |
| Diuretics | 143 | 24 | 45.28 | 72 | 22 | 42.31 |
| Cardiac therapy | 90 | 23 | 43.40 | 71 | 21 | 40.38 |
| Drugs for constipation | 45 | 21 | 39.62 | 17 | 9 | 17.31 |
| Calcium channel blockers | 24 | 20 | 37.74 | 42 | 24 | 46.15 |
| Vitamins | 31 | 19 | 35.85 | 42 | 22 | 42.31 |
| Agents acting on the renin-angiotensin system | 22 | 18 | 33.96 | 18 | 12 | 23.08 |
| Antimycotics for systemic use | 38 | 16 | 30.19 | 34 | 15 | 28.85 |
| Lipid modifying agents | 24 | 16 | 30.19 | 18 | 14 | 26.92 |
| Beta blocking agents | 27 | 15 | 28.30 | 18 | 9 | 17.31 |
| Drugs for functional gastrointestinal disorders | 28 | 15 | 28.30 | 16 | 10 | 19.23 |
| Tonics | 19 | 15 | 28.30 | 10 | 6 | 11.54 |
| Psycholeptics | 60 | 15 | 28.30 | 53 | 17 | 32.69 |
| Antidiarrheals, intestinal anti-inflammatory/anti-infective agents | 24 | 13 | 24.53 | 12 | 9 | 17.31 |
| Anti-inflammatory and antirheumatic products | 15 | 12 | 22.64 | 13 | 8 | 15.38 |
| Antihemorrhagics | 22 | 12 | 22.64 | 10 | 8 | 15.38 |
| Other hematological agents | 14 | 11 | 20.75 | 11 | 5 | 9.62 |
| General nutrients | 27 | 10 | 18.87 | 19 | 12 | 23.08 |
| Analgesics | 18 | 10 | 18.87 | 16 | 9 | 17.31 |
| Immune sera and immunoglobulins | 18 | 9 | 16.98 | 11 | 11 | 21.15 |
| Antivirals for systemic use | 11 | 9 | 16.98 | 15 | 10 | 19.23 |
| Antianemic preparations | 20 | 9 | 16.98 | 4 | 3 | 5.77 |
| Immunosuppressants | 14 | 8 | 15.09 | 17 | 12 | 23.08 |
| Antineoplastic agents | 13 | 8 | 15.09 | 20 | 10 | 19.23 |
| Anesthetics | 12 | 6 | 11.32 | 38 | 6 | 11.54 |
| Antihistamines for systemic use | 8 | 5 | 9.43 | 2 | 2 | 3.85 |
| Other alimentary tract and metabolism products | 8 | 5 | 9.43 | 9 | 8 | 15.38 |
| Other nervous system drugs | 14 | 4 | 7.55 | 4 | 3 | 5.77 |
| Antipruritics, incl. antihistamines, anesthetics, etc. | 5 | 4 | 7.55 | 1 | 1 | 1.92 |
| Thyroid therapy | 5 | 4 | 7.55 | 3 | 3 | 5.77 |
| Digestives, incl. enzymes | 4 | 3 | 5.66 | 2 | 1 | 1.92 |
| Stomatological preparations | 4 | 3 | 5.66 | 4 | 4 | 7.69 |
| Throat preparations | 4 | 3 | 5.66 | 2 | 2 | 3.85 |
| Psychoanaleptics | 8 | 3 | 5.66 | 3 | 2 | 3.85 |
| Peripheral vasodilators | 2 | 2 | 3.77 | 0 | 0 | 0.00 |
| All other therapeutic products | 3 | 2 | 3.77 | 1 | 1 | 1.92 |
| All other nontherapeutic products | 2 | 2 | 3.77 | 2 | 1 | 1.92 |
| Antihypertensives | 5 | 2 | 3.77 | 8 | 5 | 9.62 |
| Preparations for treatment of wounds and ulcers | 2 | 2 | 3.77 | 0 | 0 | 0.00 |
| Corticosteroids, dermatological preparations | 2 | 2 | 3.77 | 1 | 1 | 1.92 |
| Muscle relaxantsd | 3 | 2 | 3.77 | 7 | 2 | 3.85 |
| Surgical dressing | 2 | 1 | 1.89 | 0 | 0 | 0.00 |
| Antimycobacterials | 4 | 1 | 1.89 | 0 | 0 | 0.00 |
| Anti-Parkinson drugs | 1 | 1 | 1.89 | 0 | 0 | 0.00 |
| Antigout preparations | 1 | 1 | 1.89 | 1 | 1 | 1.92 |
| Antiepileptics | 4 | 1 | 1.89 | 0 | 0 | 0.00 |
| Antidiarrheals, intestinal anti-inflammatory/anti-infective agents | 2 | 1 | 1.89 | 3 | 2 | 3.85 |
| Urologicals | 1 | 1 | 1.89 | 9 | 6 | 11.54 |
| Antifungals for dermatological use | 1 | 1 | 1.89 | 0 | 0 | 0.00 |
| Ophthalmologicals | 1 | 1 | 1.89 | 2 | 2 | 3.85 |
| Nasal preparations | 3 | 1 | 1.89 | 0 | 0 | 0.00 |
| Topical products for joint and muscular pain | 0 | 0 | 0.00 | 1 | 1 | 1.92 |
| * Concomitant medication is coded using WHODrug 2024-Mar-1. Concomitant medication refers to drugs that start before the initial administration and end after the initial administration, or drugs that start after the initial administration. If drugs cannot be determined, they are considered as concomitant medication. | | | | | | |

| **Table 3 Concomitant antiviral, corticosteroid, and anticoagulant medications*** | | | | | | |
| --- | --- | --- | --- | --- | --- | --- |
| **ATCII** | **Meplazumab (N=53)** | | | **Placebo (N=52)** | | |
| **Drug name** | **Frequency** | **n** | **Incidence (%)** | **Frequency** | **n** | **Incidence (%)** |
| **Antivirals for systemic use** | 11 | 9 | 16.98 | 15 | 10 | 19.23 |
| Penciclovir | 5 | 5 | 9.43 | 2 | 2 | 3.85 |
| Nirmatrelvir/Ritonavir | 1 | 1 | 1.89 | 0 | 0 | 0.00 |
| Oseltamivir | 1 | 1 | 1.89 | 0 | 0 | 0.00 |
| Tenofovir Alafenamide Fumarate | 1 | 1 | 1.89 | 0 | 0 | 0.00 |
| Ganciclovir | 1 | 1 | 1.89 | 3 | 2 | 3.85 |
| Tenofovir | 1 | 1 | 1.89 | 0 | 0 | 0.00 |
| Aciclovir Monophosphate | 0 | 0 | 0.00 | 1 | 1 | 1.92 |
| Peramivir | 0 | 0 | 0.00 | 2 | 2 | 3.85 |
| Dehydroandrographolide Succinate | 0 | 0 | 0.00 | 1 | 1 | 1.92 |
| Azvudine | 0 | 0 | 0.00 | 1 | 1 | 1.92 |
| **Corticosteroids for systemic use** | 113 | 42 | 79.25 | 115 | 46 | 88.46 |
| Methylprednisolone Sodium Succinate | 58 | 29 | 54.72 | 48 | 28 | 53.85 |
| Dexamethasone Sodium Phosphate | 9 | 7 | 13.21 | 13 | 9 | 17.31 |
| Hydrocortisone Sodium Succinate | 13 | 6 | 11.32 | 11 | 9 | 17.31 |
| Methylprednisolone | 12 | 6 | 11.32 | 6 | 6 | 11.54 |
| Prednisone Acetate | 12 | 6 | 11.32 | 21 | 9 | 17.31 |
| Dexamethasone | 6 | 4 | 7.55 | 5 | 3 | 5.77 |
| Dexamethasone Acetate | 2 | 2 | 3.77 | 3 | 3 | 5.77 |
| Prednisone | 1 | 1 | 1.89 | 7 | 3 | 5.77 |
| Prednisolone Acetate | 0 | 0 | 0.00 | 1 | 1 | 1.92 |
| **Antithrombotic agents** | **98** | **44** | **83.02** | **103** | **49** | **94.23** |
| Nadroparin Calcium | 28 | 18 | 33.96 | 26 | 16 | 30.77 |
| Enoxaparin Sodium | 30 | 17 | 32.08 | 29 | 20 | 38.46 |
| Aspirin | 10 | 8 | 15.09 | 11 | 9 | 17.31 |
| Rivaroxaban | 5 | 5 | 9.43 | 6 | 6 | 11.54 |
| Dalteparin Sodium | 5 | 5 | 9.43 | 3 | 2 | 3.85 |
| Clopidogrel Hydrogen Sulfate | 5 | 4 | 7.55 | 7 | 6 | 11.54 |
| Alprostadil | 4 | 3 | 5.66 | 0 | 0 | 0.00 |
| Low Molecular Weight Heparin Calcium | 2 | 2 | 3.77 | 4 | 4 | 7.69 |
| Low Molecular Weight Heparin Sodium | 3 | 2 | 3.77 | 8 | 6 | 11.54 |
| Ticagrelor | 1 | 1 | 1.89 | 2 | 1 | 1.92 |
| Fondaparinux Sodium | 1 | 1 | 1.89 | 2 | 1 | 1.92 |
| Heparin Sodium | 1 | 1 | 1.89 | 4 | 2 | 3.85 |
| Dabigatran Etexilate | 1 | 1 | 1.89 | 1 | 1 | 1.92 |
| * Concomitant medication is coded using WHODrug 2024-Mar-1. Concomitant medication refers to drugs that start before the initial administration and end after the initial administration, or drugs that start after the initial administration. If drugs cannot be determined, they are considered as concomitant medication. | | | | | | |

| **Table 4 Duration of** **oxygen therapy** | | | | |
| --- | --- | --- | --- | --- |
|  | **Meplazumab (N=51)** | **Placebo**  **(N=52)** | **Difference (95% CI)** | **P-value**¶ |
| Total oxygen therapy days (days)* |  |  |  |  |
| n (Missing) | 48 (3) | 51 (1) |  |  |
| Mean (SD) | 16.5 (9.7) | 16.0 (10.0) | -0.52 (-4.47,3.43) | 0.7946 |
| Median | 15.0 | 13.0 |  |  |
| Q1, Q3 | 9.5, 21.5 | 10.0, 18.0 |  |  |
| Min, Max | 3, 46 | 5, 52 |  |  |
| Low-flow oxygen therapy days (days) |  |  |  |  |
| n (Missing) | 44 (7) | 41 (11) |  |  |
| Mean (SD) | 11.6 (7.9) | 11.3 (7.8) | -0.32 (-3.71,3.07) | 0.8519 |
| Median | 11.0 | 10.0 |  |  |
| Q1, Q3 | 5.0, 15.5 | 7.0, 13.0 |  |  |
| Min, Max | 1, 39 | 3, 47 |  |  |
| High-flow oxygen therapy days (days) |  |  |  |  |
| n (Missing) | 18 (33) | 24 (28) |  |  |
| Mean (SD) | 15.7 (9.9) | 13.7 (7.7) | -2.01 (-7.50,3.47) | 0.4626 |
| Median | 12.0 | 11.5 |  |  |
| Q1, Q3 | 8.0, 21.0 | 9.0, 16.0 |  |  |
| Min, Max | 4, 34 | 5, 37 |  |  |
| Total mechanical ventilation days (days) § |  |  |  |  |
| n (Missing) | 2 (49) | 3 (49) |  |  |
| Mean (SD) | 26.5 (7.8) | 29.0 (12.1) | 2.50 (-29.08,34.08) | 0.8174 |
| Median | 26.5 | 31.0 |  |  |
| Q1, Q3 | 21.0, 32.0 | 16.0, 40.0 |  |  |
| Min, Max | 21, 32 | 16, 40 |  |  |

¶ The comparison between two groups utilizes the independent sample t - test.

* The duration of oxygen therapy (days)= The oxygen therapy end date - The oxygen therapy start date + 1

§ The duration of mechanical ventilation (days)= The mechanical ventilation end date - The mechanical ventilation start date + 1

| **Table 5 Subgroup analyses of the primary efficacy endpoints (supplementary analysis ※)** | | | | | |
| --- | --- | --- | --- | --- | --- |
|  | | **Meplazumab**  **(N=51)** | **Placebo**  **(N=52)** | **Rate difference** §  **(%, 95% CI)** § | **P-value¶** |
| Smoking | Yes |  |  |  |  |
|  | All-cause mortality, n (%) | 1 (4.76) | 4 (33.33) | 28.57 (2.88, 57.59) | 0.0471 |
|  | 95% CI* | 0.12, 23.82 | 9.92, 65.11 |  |  |
|  | Total (Missing) | 21 (0) | 12 (0) |  |  |
|  | No |  |  |  |  |
|  | All-cause mortality, n (%) | 1 (3.45) | 4 (10.26) | 6.81 (-8.24, 20.91) | 0.3843 |
|  | 95% CI* | 0.09, 17.76 | 2.87, 24.22 |  |  |
|  | Total (Missing) | 29 (0) | 39 (0) |  |  |
| Age | <65 years |  |  |  |  |
|  | All-cause mortality, n (%) | 0 (0.00) | 2 (13.33) | 13.33 (-9.08, 38.36) | 0.4828 |
|  | 95% CI* | 0.00, 21.80 | 1.66, 40.46 |  |  |
|  | Total (Missing) | 15 (0) | 15 (0) |  |  |
|  | ≥65 years |  |  |  |  |
|  | All-cause mortality, n (%) | 2 (5.56) | 6 (16.22) | 10.66 (-4.43, 26.67) | 0.2611 |
|  | 95% CI* | 0.68, 18.66 | 6.19, 32.01 |  |  |
|  | Total (Missing) | 36 (0) | 37 (0) |  |  |
| Gender | Male |  |  |  |  |
|  | All-cause mortality, n (%) | 2 (5.26) | 6 (18.18) | 12.92 (-2.18, 30.12) | 0.1335 |
|  | 95% CI* | 0.64, 17.75 | 6.98, 35.46 |  |  |
|  | Total (Missing) | 38 (0) | 33 (0) |  |  |
|  | Female |  |  |  |  |
|  | All-cause mortality, n (%) | 0 (0.00) | 2 (10.53) | 10.53 (-13.94, 31.80) | 0.5020 |
|  | 95% CI* | 0.00, 24.71 | 1.30, 33.14 |  |  |
|  | Total (Missing) | 13 (0) | 19 (0) |  |  |
| Concomitant Antiviral Medication | Yes |  |  |  |  |
|  | All-cause mortality, n (%) | 1 (16.67) | 3 (42.86) | 26.19 (-26.58, 66.16) | 0.5594 |
|  | 95% CI* | 0.42, 64.12 | 9.90, 81.59 |  |  |
|  | Total (Missing) | 6 (0) | 7 (0) |  |  |
|  | No |  |  |  |  |
|  | All-cause mortality, n (%) | 1 (2.22) | 5 (11.11) | 8.89 (-1.93, 21.71) | 0.2028 |
|  | 95% CI* | 0.06, 11.77 | 3.71, 24.05 |  |  |
|  | Total (Missing) | 45 (0) | 45 (0) |  |  |
| BMI≥30 (Kg/m^2^) | Yes |  |  |  |  |
|  | All-cause mortality, n (%) | 0 (0.00) | 0 (0.00) | 0.00 (-41.12, 41.12) | 1.0000 |
|  | 95% CI* | 0.00, 45.93 | 0.00, 84.19 |  |  |
|  | Total (Missing) | 6 (0) | 2 (0) |  |  |
|  | No |  |  |  |  |
|  | All-cause mortality, n (%) | 2 (5.00) | 7 (17.07) | 12.07 (-1.98, 27.22) | 0.1549 |
|  | 95% CI* | 0.61, 16.92 | 7.15, 32.06 |  |  |
|  | Total (Missing) | 40 (0) | 41 (0) |  |  |
| Comorbidities | Yes |  |  |  |  |
|  | All-cause mortality, n (%) | 2 (4.08) | 7 (14.58) | 10.50 (-1.29, 23.81) | 0.0913 |
|  | 95% CI* | 0.50, 13.98 | 6.07, 27.76 |  |  |
|  | Total (Missing) | 49 (0) | 48 (0) |  |  |
|  | No |  |  |  |  |
|  | All-cause mortality, n (%) | 0 (0.00) | 1 (25.00) | 25.00 (-54.56, 72.78) | 1.0000 |
|  | 95% CI* | 0.00, 84.19 | 0.63, 80.59 |  |  |
|  | Total (Missing) | 2 (0) | 4 (0) |  |  |
| SARS-CoV-2 vaccination status | Yes |  |  |  |  |
|  | All-cause mortality, n (%) | 0 (0.00) | 2 (9.52) | 9.52 (-5.28, 29.19) | 0.2121 |
|  | 95% CI* | 0.00, 14.25 | 1.17, 30.38 |  |  |
|  | Total (Missing) | 24 (0) | 21 (0) |  |  |
|  | No |  |  |  |  |
|  | All-cause mortality, n (%) | 2 (8.00) | 6 (20.00) | 12.00 (-8.09, 31.14) | 0.2689 |
|  | 95% CI* | 0.98, 26.03 | 7.71, 38.57 |  |  |
|  | Total (Missing) | 25 (0) | 30 (0) |  |  |

※ The supplementary analysis is defined as following: Based on FAS, efficacy evaluation is performed with intercurrent events "early discontinuation of treatment due to poor efficacy", "concomitant use of prohibited medications/treatments affecting efficacy evaluation", and "disease aggravation" analyzed as "death" according to the composite strategy.

* The confidence intervals (CI) were estimated using the Clopper-Pearson method.

§ The rate differences and their CI were estimated using the Miettinen-Nurminen method without considering stratification factors.

¶ The comparison between two groups utilizes the Fisher's exact test.

| **Table 6 SARS-CoV-2 nucleic acid-negative conversion^＊^** | | |
| --- | --- | --- |
| **Characteristic** | **Meplazumab**  **(N=51)** | **Placebo (N=52)** |
| **Number of subjects** § | 42 | 44 |
| Nucleic acid-negative conversion, n (%) | 35 (83.33) | 35 (79.55) |
| Censored, n (%) | 7 (16.67) | 9 (20.45) |
| **Nucleic acid-negative conversion rate (%)** ⸸ |  |  |
| Day 7 (95% CI) | 63.46 (48.47, 78.32) | 49.51 (35.60, 65.40) |
| Day 14 (95% CI) | 82.96 (68.40, 93.41) | 77.91 (63.94, 89.31) |
| Day 21 (95% CI) | 89.78 (76.38, 97.28) | 81.07 (67.23, 91.64) |
| Day 28 (95% CI) | 89.78 (76.38, 97.28) | 84.22 (70.67, 93.80) |
| Day 35 (95% CI) | 96.59 (85.34, 99.74) | 87.38 (74.26, 95.74) |
| Day 42 (95% CI) | 96.59 (85.34, 99.74) | 87.38 (74.26, 95.74) |
| Day 49 (95% CI) | 96.59 (85.34, 99.74) | 87.38 (74.26, 95.74) |
| Day 56 (95% CI) | 96.59 (85.34, 99.74) | 87.38 (74.26, 95.74) |
| **Nucleic acid-negative conversion time (days)** ¶ |  |  |
| Min, Max ✤ | 2.00, 62.00+ | 2.00, 61.00+ |
| Q1 (95% CI) | 3.00 (2.00, 5.00) | 3.50 (2.00, 6.00) |
| Median (95% CI) | 7.00 (5.00, 9.00) | 9.00 (5.00, 10.00) |
| Q3 (95% CI) | 14.00 (8.00, 18.00) | 12.00 (10.00, NR) |
| **Comparison between groups (meplazumab / placebo)** |  |  |
| Risk ratio (95% CI) ♠ | 1.15 (0.72, 1.84) |  |
| Log-Rank test statistic ♣ | 0.381 |  |
| P-value ♣ | 0.54 |  |
| **＊** SARS-CoV-2 nucleic acid-negative conversion is defined as two consecutive negative tests for the SARS-CoV2 nucleic acid with a sampling interval of at least 24 hours. NR = Not reached, indicating that the event was not reached or achieved.  § Subjects with positive baseline nucleic acid tests.  ⸸ Nucleic acid-negative conversion rate is calculated using the Kaplan-Meier method and the two-sided 95% confidence interval (CI) is estimated using the Greenwood formula.  ¶ The nucleic acid-negative conversion time is defined as the sampling time of the first negative nucleic acid test among the two consecutive negative tests. The nucleic acid-negative conversion time is calculated using the Kaplan-Meier method and the two-sided 95% CI is estimated with the Brookmeyer & Crowley method.  ✤ "Min, Max" includes censored times. "+" indicates that the subject has not yet tested negative at that time.  ♠ The risk ratio is calculated using the Cox proportional hazards model, with the time to test negative as the dependent variable and treatment groups (meplazumab and placebo) and stratification factors (age groups: < 65 years vs. ≥ 65 years) as the fixed effects.  ♣ The Log-Rank test and P-value are calculated using the stratified Log-Rank test. | | |

| **Table 7 Quantification of viral load*** | | | | | |
| --- | --- | --- | --- | --- | --- |
| **Characteristic** | **Meplazumab** | **Placebo** | **Statistics** | **Rate difference**  **(%, 95% CI)** | **P-value** |
| Baseline |  |  |  |  |  |
| n (Missing) | 33 (9) | 38 (6) |  |  |  |
| Mean (SD) | 33.702 (4.679) | 33.651 (5.273) | Grouped t-test | -0.05 (-2.43, 2.32) | 0.966 |
| Difference from baseline on the day 7 |  |  |  |  |  |
| n (Missing) | 11 (31) | 18 (26) |  |  |  |
| Mean (SD) | 3.649 (5.200) | 3.612 (4.479) | Grouped t-test | -0.04 (-3.77, 3.70) | 0.984 |
| Difference from baseline on the day 14 |  |  |  |  |  |
| n (Missing) | 4 (38) | 2 (42) |  |  |  |
| Mean (SD) | 4.340 (3.480) | 3.535 (2.171) | Grouped t-test | -0.81 (-8.51, 6.90) | 0.786 |
| Difference from baseline on the day 28 |  |  |  |  |  |
| n (Missing) | 2 (40) | 4 (40) |  |  |  |
| Mean (SD) | 7.010 (0.467) | 5.103 (6.528) | Grouped t-test | -1.91 (-15.51, 11.70) | 0.717 |
| * Analysis based on subjects with positive nucleic acid testing at baseline. Viral load is evaluated by the cycle threshold of ORF1ab using PCR method, and subjects with negative ORF1ab gene test for viral load are processed as the cutoff +1. The baseline is defined as the last non-null observation prior to the first treatment. | | | | | |

| **Table 8 Treatment emergent adverse events (TEAEs)*** | | | | | | | | | |  |
| --- | --- | --- | --- | --- | --- | --- | --- | --- | --- | --- |
| **System organ class** | **Meplazumab (N=53)** | | | **Placebo (N=52)** | | | **Total (N=105)** | | |  |
| **Preferred term** | **Frequency** | **n** | **Incidence (%)** | **Frequency** | **n** | **Incidence (%)** | **Frequency** | **n** | **Incidence (%)** | |
| **TEAE** | 160 | 43 | 81.13 | 124 | 40 | 76.92 | 284 | 83 | 79.05 | |
| **Metabolic and nutritional disorders** | 44 | 16 | 30.19 | 27 | 19 | 36.54 | 71 | 35 | 33.33 | |
| Hyponatremia | 9 | 6 | 11.32 | 0 | 0 | 0.00 | 9 | 6 | 5.71 | |
| Hypokalemia | 10 | 5 | 9.43 | 9 | 9 | 17.31 | 19 | 14 | 13.33 | |
| Hyperlipidemia | 4 | 4 | 7.55 | 5 | 5 | 9.62 | 9 | 9 | 8.57 | |
| Hyperuricemia | 4 | 3 | 5.66 | 1 | 1 | 1.92 | 5 | 4 | 3.81 | |
| Hyperkalemia | 3 | 3 | 5.66 | 0 | 0 | 0.00 | 3 | 3 | 2.86 | |
| Hypochloremia | 4 | 2 | 3.77 | 1 | 1 | 1.92 | 5 | 3 | 2.86 | |
| Hypophosphatemia | 2 | 2 | 3.77 | 1 | 1 | 1.92 | 3 | 3 | 2.86 | |
| Hypocalcemia | 2 | 2 | 3.77 | 1 | 1 | 1.92 | 3 | 3 | 2.86 | |
| Type 2 diabetes mellitus | 1 | 1 | 1.89 | 0 | 0 | 0.00 | 1 | 1 | 0.95 | |
| Hypoproteinemia | 1 | 1 | 1.89 | 4 | 4 | 7.69 | 5 | 5 | 4.76 | |
| Malnutrition | 1 | 1 | 1.89 | 1 | 1 | 1.92 | 2 | 2 | 1.90 | |
| Hypertriglyceridemia | 1 | 1 | 1.89 | 0 | 0 | 0.00 | 1 | 1 | 0.95 | |
| Hypercholesterolemia | 1 | 1 | 1.89 | 2 | 1 | 1.92 | 3 | 2 | 1.90 | |
| Hypernatremia | 1 | 1 | 1.89 | 0 | 0 | 0.00 | 1 | 1 | 0.95 | |
| Hypoglycemia | 0 | 0 | 0.00 | 1 | 1 | 1.92 | 1 | 1 | 0.95 | |
| Hyperchloremia | 0 | 0 | 0.00 | 1 | 1 | 1.92 | 1 | 1 | 0.95 | |
| **General disorders and administration site conditions** | 21 | 15 | 28.30 | 13 | 9 | 17.31 | 34 | 24 | 22.86 | |
| Pyrexia | 15 | 11 | 20.75 | 11 | 7 | 13.46 | 26 | 18 | 17.14 | |
| Chills | 2 | 2 | 3.77 | 0 | 0 | 0.00 | 2 | 2 | 1.90 | |
| Device-related thrombosis | 1 | 1 | 1.89 | 0 | 0 | 0.00 | 1 | 1 | 0.95 | |
| Multi-organ failure syndrome | 1 | 1 | 1.89 | 1 | 1 | 1.92 | 2 | 2 | 1.90 | |
| Intolerance to temperature changes | 1 | 1 | 1.89 | 0 | 0 | 0.00 | 1 | 1 | 0.95 | |
| Disease progression | 1 | 1 | 1.89 | 1 | 1 | 1.92 | 2 | 2 | 1.90 | |
| **Investigations** | 44 | 15 | 28.30 | 24 | 11 | 21.15 | 68 | 26 | 24.76 | |
| Alanine aminotransferase increased | 5 | 5 | 9.43 | 4 | 4 | 7.69 | 9 | 9 | 8.57 | |
| Aspartate aminotransferase increased | 5 | 4 | 7.55 | 3 | 3 | 5.77 | 8 | 7 | 6.67 | |
| Blood glucose increased | 4 | 4 | 7.55 | 0 | 0 | 0.00 | 4 | 4 | 3.81 | |
| Platelet count decreased | 3 | 3 | 5.66 | 2 | 2 | 3.85 | 5 | 5 | 4.76 | |
| Weight decreased | 2 | 2 | 3.77 | 2 | 2 | 3.85 | 4 | 4 | 3.81 | |
| Monocyte count increased | 3 | 2 | 3.77 | 0 | 0 | 0.00 | 3 | 2 | 1.90 | |
| Lymphocyte count decreased | 3 | 2 | 3.77 | 0 | 0 | 0.00 | 3 | 2 | 1.90 | |
| Serum albumin decreased | 2 | 2 | 3.77 | 0 | 0 | 0.00 | 2 | 2 | 1.90 | |
| Fibrinogen increased | 2 | 2 | 3.77 | 0 | 0 | 0.00 | 2 | 2 | 1.90 | |
| Fibrinogen decreased | 2 | 2 | 3.77 | 1 | 1 | 1.92 | 3 | 3 | 2.86 | |
| Gamma-glutamyl transferase increased | 1 | 1 | 1.89 | 2 | 2 | 3.85 | 3 | 3 | 2.86 | |
| Neutrophil count decreased | 1 | 1 | 1.89 | 0 | 0 | 0.00 | 1 | 1 | 0.95 | |
| Coagulation time prolonged | 1 | 1 | 1.89 | 0 | 0 | 0.00 | 1 | 1 | 0.95 | |
| Prothrombin time prolonged | 1 | 1 | 1.89 | 1 | 1 | 1.92 | 2 | 2 | 1.90 | |
| Heart rate increased | 1 | 1 | 1.89 | 0 | 0 | 0.00 | 1 | 1 | 0.95 | |
| Electrocardiogram QT interval prolonged | 1 | 1 | 1.89 | 0 | 0 | 0.00 | 1 | 1 | 0.95 | |
| White blood cell count decreased | 1 | 1 | 1.89 | 0 | 0 | 0.00 | 1 | 1 | 0.95 | |
| Fibrin D-dimer increased | 1 | 1 | 1.89 | 0 | 0 | 0.00 | 1 | 1 | 0.95 | |
| Blood lactate increased | 1 | 1 | 1.89 | 0 | 0 | 0.00 | 1 | 1 | 0.95 | |
| Lactate dehydrogenase increased | 1 | 1 | 1.89 | 1 | 1 | 1.92 | 2 | 2 | 1.90 | |
| Alkaline phosphatase increased | 1 | 1 | 1.89 | 0 | 0 | 0.00 | 1 | 1 | 0.95 | |
| Creatinine increased | 1 | 1 | 1.89 | 0 | 0 | 0.00 | 1 | 1 | 0.95 | |
| Bilirubin increased | 1 | 1 | 1.89 | 0 | 0 | 0.00 | 1 | 1 | 0.95 | |
| Neutrophil count increased | 0 | 0 | 0.00 | 2 | 2 | 3.85 | 2 | 2 | 1.90 | |
| Coagulation test abnormal | 0 | 0 | 0.00 | 1 | 1 | 1.92 | 1 | 1 | 0.95 | |
| Eosinophil count increased | 0 | 0 | 0.00 | 1 | 1 | 1.92 | 1 | 1 | 0.95 | |
| Urine glucose | 0 | 0 | 0.00 | 1 | 1 | 1.92 | 1 | 1 | 0.95 | |
| White blood cell count increased | 0 | 0 | 0.00 | 2 | 2 | 3.85 | 2 | 2 | 1.90 | |
| Troponin increased | 0 | 0 | 0.00 | 1 | 1 | 1.92 | 1 | 1 | 0.95 | |
| **Infections and infestations** | 8 | 7 | 13.21 | 9 | 8 | 15.38 | 17 | 15 | 14.29 | |
| Urinary tract infection | 2 | 2 | 3.77 | 1 | 1 | 1.92 | 3 | 3 | 2.86 | |
| COVID-19 pneumonia | 1 | 1 | 1.89 | 0 | 0 | 0.00 | 1 | 1 | 0.95 | |
| EB virus infection | 1 | 1 | 1.89 | 0 | 0 | 0.00 | 1 | 1 | 0.95 | |
| Legionella infection | 1 | 1 | 1.89 | 0 | 0 | 0.00 | 1 | 1 | 0.95 | |
| Candidiasis | 1 | 1 | 1.89 | 1 | 1 | 1.92 | 2 | 2 | 1.90 | |
| Infectious pneumonia | 1 | 1 | 1.89 | 3 | 3 | 5.77 | 4 | 4 | 3.81 | |
| Bacterial pneumonia | 1 | 1 | 1.89 | 0 | 0 | 0.00 | 1 | 1 | 0.95 | |
| Upper respiratory tract infection | 0 | 0 | 0.00 | 1 | 1 | 1.92 | 1 | 1 | 0.95 | |
| Oral candidiasis | 0 | 0 | 0.00 | 1 | 1 | 1.92 | 1 | 1 | 0.95 | |
| Sepsis | 0 | 0 | 0.00 | 2 | 2 | 3.85 | 2 | 2 | 1.90 | |
| **Gastrointestinal disorders** | 10 | 7 | 13.21 | 6 | 5 | 9.62 | 16 | 12 | 11.43 | |
| Constipation | 3 | 3 | 5.66 | 1 | 1 | 1.92 | 4 | 4 | 3.81 | |
| Diarrhea | 2 | 2 | 3.77 | 0 | 0 | 0.00 | 2 | 2 | 1.90 | |
| Belching | 1 | 1 | 1.89 | 0 | 0 | 0.00 | 1 | 1 | 0.95 | |
| Nausea | 1 | 1 | 1.89 | 0 | 0 | 0.00 | 1 | 1 | 0.95 | |
| Toothache | 1 | 1 | 1.89 | 0 | 0 | 0.00 | 1 | 1 | 0.95 | |
| Gastroesophageal reflux disease | 1 | 1 | 1.89 | 0 | 0 | 0.00 | 1 | 1 | 0.95 | |
| Abdominal distension | 1 | 1 | 1.89 | 0 | 0 | 0.00 | 1 | 1 | 0.95 | |
| Chronic gastritis | 0 | 0 | 0.00 | 1 | 1 | 1.92 | 1 | 1 | 0.95 | |
| Gastrointestinal hemorrhage | 0 | 0 | 0.00 | 1 | 1 | 1.92 | 1 | 1 | 0.95 | |
| Ascites | 0 | 0 | 0.00 | 1 | 1 | 1.92 | 1 | 1 | 0.95 | |
| Abdominal pain | 0 | 0 | 0.00 | 1 | 1 | 1.92 | 1 | 1 | 0.95 | |
| Tongue ulcer | 0 | 0 | 0.00 | 1 | 1 | 1.92 | 1 | 1 | 0.95 | |
| **Hepatobiliary disorders** | 6 | 6 | 11.32 | 4 | 4 | 7.69 | 10 | 10 | 9.52 | |
| Hepatic dysfunction | 5 | 5 | 9.43 | 2 | 2 | 3.85 | 7 | 7 | 6.67 | |
| Hepatic steatosis | 1 | 1 | 1.89 | 0 | 0 | 0.00 | 1 | 1 | 0.95 | |
| Primary biliary cholangitis | 0 | 0 | 0.00 | 1 | 1 | 1.92 | 1 | 1 | 0.95 | |
| Cirrhosis | 0 | 0 | 0.00 | 1 | 1 | 1.92 | 1 | 1 | 0.95 | |
| **Hematological and lymphatic system disorders** | 6 | 6 | 11.32 | 5 | 5 | 9.62 | 11 | 11 | 10.48 | |
| Anemia | 4 | 4 | 7.55 | 3 | 3 | 5.77 | 7 | 7 | 6.67 | |
| Megaloblastic anemia | 1 | 1 | 1.89 | 0 | 0 | 0.00 | 1 | 1 | 0.95 | |
| Thrombocytopenia | 1 | 1 | 1.89 | 0 | 0 | 0.00 | 1 | 1 | 0.95 | |
| Hypofibrinogenemia | 0 | 0 | 0.00 | 1 | 1 | 1.92 | 1 | 1 | 0.95 | |
| Hypercoagulable state | 0 | 0 | 0.00 | 1 | 1 | 1.92 | 1 | 1 | 0.95 | |
| **Respiratory, thoracic and mediastinal disorders** | 5 | 4 | 7.55 | 19 | 12 | 23.08 | 24 | 16 | 15.24 | |
| Respiratory failure | 1 | 1 | 1.89 | 3 | 3 | 5.77 | 4 | 4 | 3.81 | |
| Chronic obstructive pulmonary disease | 1 | 1 | 1.89 | 0 | 0 | 0.00 | 1 | 1 | 0.95 | |
| Hypoxia | 1 | 1 | 1.89 | 0 | 0 | 0.00 | 1 | 1 | 0.95 | |
| Pleural effusion | 1 | 1 | 1.89 | 3 | 3 | 5.77 | 4 | 4 | 3.81 | |
| Interstitial lung disease | 1 | 1 | 1.89 | 2 | 2 | 3.85 | 3 | 3 | 2.86 | |
| Oropharyngeal discomfort | 0 | 0 | 0.00 | 1 | 1 | 1.92 | 1 | 1 | 0.95 | |
| Oropharyngeal pain | 0 | 0 | 0.00 | 1 | 1 | 1.92 | 1 | 1 | 0.95 | |
| Respiratory acidosis | 0 | 0 | 0.00 | 1 | 1 | 1.92 | 1 | 1 | 0.95 | |
| Pneumothorax | 0 | 0 | 0.00 | 1 | 1 | 1.92 | 1 | 1 | 0.95 | |
| Pulmonary fibrosis | 0 | 0 | 0.00 | 1 | 1 | 1.92 | 1 | 1 | 0.95 | |
| Pulmonary inflammation | 0 | 0 | 0.00 | 5 | 5 | 9.62 | 5 | 5 | 4.76 | |
| Hypercapnia | 0 | 0 | 0.00 | 1 | 1 | 1.92 | 1 | 1 | 0.95 | |
| **Vascular and lymphatic disorders** | 5 | 3 | 5.66 | 2 | 2 | 3.85 | 7 | 5 | 4.76 | |
| Deep vein thrombosis of the limbs | 3 | 2 | 3.77 | 0 | 0 | 0.00 | 3 | 2 | 1.90 | |
| Head and arm vein thrombosis | 1 | 1 | 1.89 | 0 | 0 | 0.00 | 1 | 1 | 0.95 | |
| Deep vein thrombosis | 1 | 1 | 1.89 | 1 | 1 | 1.92 | 2 | 2 | 1.90 | |
| Thrombosis | 0 | 0 | 0.00 | 1 | 1 | 1.92 | 1 | 1 | 0.95 | |
| **Nervous system disorders** | 2 | 2 | 3.77 | 2 | 1 | 1.92 | 4 | 3 | 2.86 | |
| Cerebral infarction | 1 | 1 | 1.89 | 0 | 0 | 0.00 | 1 | 1 | 0.95 | |
| Petit mal seizure | 1 | 1 | 1.89 | 0 | 0 | 0.00 | 1 | 1 | 0.95 | |
| Somnolence | 0 | 0 | 0.00 | 1 | 1 | 1.92 | 1 | 1 | 0.95 | |
| Cerebrovascular disorder | 0 | 0 | 0.00 | 1 | 1 | 1.92 | 1 | 1 | 0.95 | |
| **Cardiac disorders** | 2 | 2 | 3.77 | 5 | 5 | 9.62 | 7 | 7 | 6.67 | |
| Heart failure | 1 | 1 | 1.89 | 2 | 2 | 3.85 | 3 | 3 | 2.86 | |
| Atrial flutter | 1 | 1 | 1.89 | 0 | 0 | 0.00 | 1 | 1 | 0.95 | |
| Myocardial injury | 0 | 0 | 0.00 | 1 | 1 | 1.92 | 1 | 1 | 0.95 | |
| Myocardial ischemia | 0 | 0 | 0.00 | 1 | 1 | 1.92 | 1 | 1 | 0.95 | |
| Acute coronary syndrome | 0 | 0 | 0.00 | 1 | 1 | 1.92 | 1 | 1 | 0.95 | |
| **Skin and subcutaneous tissue disorders** | 2 | 2 | 3.77 | 0 | 0 | 0.00 | 2 | 2 | 1.90 | |
| Eczema | 1 | 1 | 1.89 | 0 | 0 | 0.00 | 1 | 1 | 0.95 | |
| Pressure ulcer | 1 | 1 | 1.89 | 0 | 0 | 0.00 | 1 | 1 | 0.95 | |
| **Renal and urinary disorders** | 2 | 2 | 3.77 | 3 | 2 | 3.85 | 5 | 4 | 3.81 | |
| Renal failure | 1 | 1 | 1.89 | 1 | 1 | 1.92 | 2 | 2 | 1.90 | |
| Hematuria | 1 | 1 | 1.89 | 0 | 0 | 0.00 | 1 | 1 | 0.95 | |
| Urinary retention | 0 | 0 | 0.00 | 1 | 1 | 1.92 | 1 | 1 | 0.95 | |
| Renal cyst | 0 | 0 | 0.00 | 1 | 1 | 1.92 | 1 | 1 | 0.95 | |
| **Musculoskeletal and connective tissue disorders** | 2 | 1 | 1.89 | 2 | 2 | 3.85 | 4 | 3 | 2.86 | |
| Muscle hematoma | 1 | 1 | 1.89 | 0 | 0 | 0.00 | 1 | 1 | 0.95 | |
| Back pain | 1 | 1 | 1.89 | 0 | 0 | 0.00 | 1 | 1 | 0.95 | |
| Arthralgia | 0 | 0 | 0.00 | 1 | 1 | 1.92 | 1 | 1 | 0.95 | |
| Connective tissue disease | 0 | 0 | 0.00 | 1 | 1 | 1.92 | 1 | 1 | 0.95 | |
| **Ocular disorders** | 1 | 1 | 1.89 | 0 | 0 | 0.00 | 1 | 1 | 0.95 | |
| Abnormal eye movement | 1 | 1 | 1.89 | 0 | 0 | 0.00 | 1 | 1 | 0.95 | |
| **Psychiatric disorders** | 0 | 0 | 0.00 | 3 | 2 | 3.85 | 3 | 2 | 1.90 | |
| Insomnia | 0 | 0 | 0.00 | 1 | 1 | 1.92 | 1 | 1 | 0.95 | |
| Depression | 0 | 0 | 0.00 | 1 | 1 | 1.92 | 1 | 1 | 0.95 | |
| Anxiety | 0 | 0 | 0.00 | 1 | 1 | 1.92 | 1 | 1 | 0.95 | |
| *Adverse events were coded using MedDRA version 27.0. | | | | | | | | | |  |

| **Table 9. Treatment emergent adverse events (TEAEs) related to the investigation drug*** | | | | | | | | | |  |
| --- | --- | --- | --- | --- | --- | --- | --- | --- | --- | --- |
| **System organ class** | **Meplazumab (N=53)** | | | **Placebo (N=52)** | | | **Total (N=105)** | | |  |
| **Preferred term** | **Frequency** | **n** | **Incidence (%)** | **Frequency** | **n** | **Incidence (%)** | **Frequency** | **n** | **Incidence (%)** |  |
| **TEAE** | 7 | 6 | 11.32 | 6 | 5 | 9.62 | 13 | 11 | 10.48 |  |
| Grade 1 | 2 | 2 | 3.77 | 3 | 3 | 5.77 | 5 | 5 | 4.76 |  |
| Grade 2 | 5 | 4 | 7.55 | 3 | 3 | 5.77 | 8 | 7 | 6.67 |  |
| Grade 3 | 0 | 0 | 0.00 | 0 | 0 | 0.00 | 0 | 0 | 0.00 |  |
| Grade 4 | 0 | 0 | 0.00 | 0 | 0 | 0.00 | 0 | 0 | 0.00 |  |
| Grade 5 | 0 | 0 | 0.00 | 0 | 0 | 0.00 | 0 | 0 | 0.00 |  |
| ≥Grade 3 | 0 | 0 | 0.00 | 0 | 0 | 0.00 | 0 | 0 | 0.00 |  |
| **General disorders and administration site conditions** | 4 | 3 | 5.66 | 2 | 2 | 3.85 | 6 | 5 | 4.76 |  |
| Grade 1 | 1 | 1 | 1.89 | 2 | 2 | 3.85 | 3 | 3 | 2.86 |  |
| Grade 2 | 3 | 2 | 3.77 | 0 | 0 | 0.00 | 3 | 2 | 1.90 |  |
| ≥Grade 3 | 0 | 0 | 0.00 | 0 | 0 | 0.00 | 0 | 0 | 0.00 |  |
| Fever | 2 | 2 | 3.77 | 2 | 2 | 3.85 | 4 | 4 | 3.81 |  |
| Grade 1 | 1 | 1 | 1.89 | 2 | 2 | 3.85 | 3 | 3 | 2.86 |  |
| Grade 2 | 1 | 1 | 1.89 | 0 | 0 | 0.00 | 1 | 1 | 0.95 |  |
| ≥Grade 3 | 0 | 0 | 0.00 | 0 | 0 | 0.00 | 0 | 0 | 0.00 |  |
| Chills | 1 | 1 | 1.89 | 0 | 0 | 0.00 | 1 | 1 | 0.95 |  |
| Grade 1 | 0 | 0 | 0.00 | 0 | 0 | 0.00 | 0 | 0 | 0.00 |  |
| Grade 2 | 1 | 1 | 1.89 | 0 | 0 | 0.00 | 1 | 1 | 0.95 |  |
| ≥Grade 3 | 0 | 0 | 0.00 | 0 | 0 | 0.00 | 0 | 0 | 0.00 |  |
| Temperature intolerance | 1 | 1 | 1.89 | 0 | 0 | 0.00 | 1 | 1 | 0.95 |  |
| Grade 1 | 0 | 0 | 0.00 | 0 | 0 | 0.00 | 0 | 0 | 0.00 |  |
| Grade 2 | 1 | 1 | 1.89 | 0 | 0 | 0.00 | 1 | 1 | 0.95 |  |
| ≥Grade 3 | 0 | 0 | 0.00 | 0 | 0 | 0.00 | 0 | 0 | 0.00 |  |
| **Hepatobiliary disorders** | 2 | 2 | 3.77 | 2 | 2 | 3.85 | 4 | 4 | 3.81 |  |
| Grade 1 | 1 | 1 | 1.89 | 0 | 0 | 0.00 | 1 | 1 | 0.95 |  |
| Grade 2 | 1 | 1 | 1.89 | 2 | 2 | 3.85 | 3 | 3 | 2.86 |  |
| ≥Grade 3 | 0 | 0 | 0.00 | 0 | 0 | 0.00 | 0 | 0 | 0.00 |  |
| Liver function abnormality | 2 | 2 | 3.77 | 2 | 2 | 3.85 | 4 | 4 | 3.81 |  |
| Grade 1 | 1 | 1 | 1.89 | 0 | 0 | 0.00 | 1 | 1 | 0.95 |  |
| Grade 2 | 1 | 1 | 1.89 | 2 | 2 | 3.85 | 3 | 3 | 2.86 |  |
| ≥Grade 3 | 0 | 0 | 0.00 | 0 | 0 | 0.00 | 0 | 0 | 0.00 |  |
| **Investigations** | 1 | 1 | 1.89 | 2 | 1 | 1.92 | 3 | 2 | 1.90 |  |
| Grade 1 | 0 | 0 | 0.00 | 1 | 1 | 1.92 | 1 | 1 | 0.95 |  |
| Grade 2 | 1 | 1 | 1.89 | 1 | 1 | 1.92 | 2 | 2 | 1.90 |  |
| ≥Grade 3 | 0 | 0 | 0.00 | 0 | 0 | 0.00 | 0 | 0 | 0.00 |  |
| Increased bilirubin | 1 | 1 | 1.89 | 0 | 0 | 0.00 | 1 | 1 | 0.95 |  |
| Grade 1 | 0 | 0 | 0.00 | 0 | 0 | 0.00 | 0 | 0 | 0.00 |  |
| Grade 2 | 1 | 1 | 1.89 | 0 | 0 | 0.00 | 1 | 1 | 0.95 |  |
| ≥Grade 3 | 0 | 0 | 0.00 | 0 | 0 | 0.00 | 0 | 0 | 0.00 |  |
| Increased γ-glutamyl transferase | 0 | 0 | 0.00 | 1 | 1 | 1.92 | 1 | 1 | 0.95 |  |
| Grade 1 | 0 | 0 | 0.00 | 1 | 1 | 1.92 | 1 | 1 | 0.95 |  |
| Grade 2 | 0 | 0 | 0.00 | 0 | 0 | 0.00 | 0 | 0 | 0.00 |  |
| ≥Grade 3 | 0 | 0 | 0.00 | 0 | 0 | 0.00 | 0 | 0 | 0.00 |  |
| Increased alanine Aminotransferase | 0 | 0 | 0.00 | 1 | 1 | 1.92 | 1 | 1 | 0.95 |  |
| Grade 1 | 0 | 0 | 0.00 | 0 | 0 | 0.00 | 0 | 0 | 0.00 |  |
| Grade 2 | 0 | 0 | 0.00 | 1 | 1 | 1.92 | 1 | 1 | 0.95 |  |
| ≥Grade 3 | 0 | 0 | 0.00 | 0 | 0 | 0.00 | 0 | 0 | 0.00 |  |
| * Adverse events (AE) were coded using MedDRA version 27.0. AE related to the investigation drug are classified as "related", "likely related", or "possibly related". | | | | | | | | | | |
