# Supplementary material for: Meplazumab, a CD147 antibody, for severe COVID-19: a double-blind, randomized, placebo-controlled, phase 3 clinical trial
Source: Signal Transduct Target Ther. 2025 Apr 14;10:119. doi: 10.1038/s41392-025-02208-9 (PMC11994814; doi:10.1038/s41392-025-02208-9)
Supplement: Supplementary file 2 — Protocol and SAP [file 41392_2025_2208_MOESM2_ESM.pdf]

Meplazumab, a CD147 antibody, for severe Covid-19: a double-blind, randomized, placebo-controlled, phase 3 clinical trial

Protocol and Statistical Analysis Plan

This supplement contains the following items：

- Original protocol of the study (v1.0)
- Final protocol of the study (v1.6)
- Summary of amendments to the protocol
- Original statistical analysis plan (v1.0)

Clinical Trial Approval Letter No.:

Registration Classification of Therapeutic Biological Products: Class I

Clinical Study Protocol

A Multicenter, Double-blind, Randomized, Placebo-controlled, Add-on Phase III Clinical Study to Evaluate the Safety and Efficacy of Meplazumab for Injection in Patients with Severe SARS-CoV-2 Infection

Protocol No.:	MPZ-III-01-CN
Sponsor:	Jiangsu Pacific Meinuoke Bio-pharmaceutical Co., Ltd.<br>Fourth Military Medical University
Principal Director:	Zhi-Nan Chen
Contract Research Organizations:	Beijing Key Tech Statistical Technology Co., Ltd.<br>Beijing CTSmed Co., Ltd.
Version No.:	1.0
Version Date:	December 29, 2022

Confidentiality Statement

This document contains important confidential information, which is the property of the sponsor and shall not be disclosed unless required by current laws or regulations. All persons to whom this document is addressed should be informed of this confidentiality requirement. The requirement is also applicable to all the documents provided to you that are confidential in the future.

Compliance Statement

This study will be conducted in accordance with Good Clinical Practices (GCP)\ICH-GCP and the following regulations and guidelines applicable to clinical studies:

1) *Drug Administration Law of the People's Republic of China* (2019)

2) *Provisions for Drug Registration* (2020)

3) *Declaration of Helsinki* (2013)

4) *Guidelines for Laboratory Management of Biological Sample Analysis in Drug Clinical Trials* (Trial) (2011)

The principal investigator will ensure that the protocol remains unviolated and unchanged before obtaining permission from the sponsor and the Ethics Committee, except when necessary to prevent direct harm to subjects.

The study protocol, informed consent form (ICF), approval letter from the National Medical Products Administration (NMPA) of China, certificates of analysis (CoAs) and other relevant materials will be submitted to the Ethics Committee for review and approval. Before subject enrollment, an approval letter must be obtained from the Ethics Committee. Any amendments to the protocol must also be reviewed and approved by the Ethics Committee before implementation.

Table of Contents

Table of Contents 4
Synopsis 6
1 Introduction 16
1.1 SARS-CoV-2 17
1.2 CD147 Target 17
1.3 Meplazumab for Injection 19
1.4 Mechanism of Meplazumab for Injection in the Treatment of COVID-19 20
2 Study Objectives 22
3 Study Design and Principle 22
3.1 Overall Design 22
3.2 Overview of Study Design 22
3.3 Principle of Study Design 22
4 Study Population 24
4.1 Inclusion Criteria 24
4.2 Exclusion Criteria 24
5 Treatment Assignment and Blinding 25
5.1 Study Blindness 26
5.2 Emergency Unblinding 26
5.3 Blind Review and Unblinding 27
6 Dosage and Method of Administration 27
6.1 Rationale for Dose Selection 27
6.2 Method of Administration 29
6.3 End of Study 29
6.4 Treatment Regimen 29
7 Medication Compliance 30
8 Concomitant Therapy 30
8.1 Permitted Drug Therapies for Subjects During the Study 30
8.2 Contraindicated Medications for Subjects During the Study 30
9 Study Evaluation 31
9.1. Efficacy Measurements 31
9.2 Efficacy Evaluation 31
9.3 Safety Evaluation 32
10 Early Termination of Study 32
11 Subject Completion/Withdrawal 33
11.1 Completion 33
11.2 Early Termination/Withdrawal 33
11.3 Case Report Form (CRF) 34
11.4 Record Retention 34
12 Statistical Analysis 34
12.1 Statistical and Analytical Plans 34
12.2 Analysis Data Set 34
12.3 General Principles for Statistical Analysis 35

12.4 Efficacy Analysis 35
12.5 Safety Analysis 35
12.6 Interim Analysis 36
12.7 Determination of Sample Size 36
13 Adverse Event Reporting 36
13.1 Relevant Definitions 36
13.2 Additional Considerations for PTEs and AEs 38
13.3 Definition of Causality 40
13.4 Judgment Criteria for Severity 41
13.5 Actions with the Study Drug 42
13.6 Outcome of Adverse Events 42
13.7 Collection and Reporting Procedures 43
13.8 Death 46
13.9 Hospitalization 46
13.10 Pregnancy 46
13.11 Overdose 47
14 Information of Study Drug 47
14.1 Name and Strength of Therapeutic Drug 47
14.2 Drug Product Formulation 49
14.3 Drug Packaging and Label 49
14.4 Drug Management 49
15 Ethics 50
15.1 Investigator's Responsibility 50
15.2 Independent Ethics Committee (IEC)/Institutional Review Board (IRB) 50
15.3 Informed Consent 50
15.4 Protection of Subject Data 51
16 Management Requirements 51
16.1 Protocol Amendment 51
16.2 Data Management 51
16.3 Monitoring 53
16.4 Audits and Inspections 53
16.5 Verification of Original Records 53
16.6 Study Completion/Termination 54
16.7 Confidentiality Agreement and Patient Privacy 54
16.8 Use and Publication of Information 55
17 References 55

Synopsis

**Study Title**	A Multicenter, Double-blind, Randomized, Placebo-controlled, Add-on Phase III Clinical Study to Evaluate the Safety and Efficacy of Meplazumab for Injection in Patients with Severe SARS-CoV-2 Infection
**Protocol No.**	MPZ-III-01-CN
**Phase of Development**	III
**Indication**	Patients diagnosed with severe SARS-CoV-2 infection as per the *Diagnosis and Treatment Protocol for COVID-19 Patients (Tentative 9th Version)* issued by the National Health Commission (NHC) of China.
**Study Objectives**	To evaluate the safety and efficacy of Meplazumab for Injection when added to the standard of care, compared to the standard of care alone, in patients with severe SARS-CoV-2 infection.
**Number of Subjects**	Approximately 350 subjects are planned to be enrolled
**Population**	Patients with severe SARS-CoV-2 infection
**Study Design**	This is a multicenter, double-blind, randomized, placebo-controlled, add-on phase III clinical study.<br>A 0.2 mg/kg test group and a placebo group will be set up for the study based on the standard of care protocol (SoC) in the *Diagnosis and Treatment Protocol for COVID-19 Patients (Tentative 9th Version)* [1], the *Technical Guidelines for Clinical Trials of New Antiviral Drugs for COVID-19 (Trial)* [2] and previous clinical study results. Treatment regimen: a single intravenous (IV) infusion of 0.2 mg/kg Meplazumab or placebo on the first day (D0) and D7 after the first dose during the treatment period, calculated based on subjects' body weight. It is expected that 350 subjects will be randomized (1:1) into the Meplazumab and placebo groups. Each subject will be evaluated for short-term efficacy within 28 days after the first dose to determine the treatment effect and safety of Meplazumab. Each subject will undergo a long-term follow-up within 56 days after the first dose to determine the safety of Meplazumab.<br>Subjects will be diagnosed and treated as per the *Diagnosis and Treatment Protocol for COVID-19 Patients (Tentative 9th Version)* issued by NHC and relevant clinical diagnosis and treatment guidelines. Any adverse events (AEs) that occur will be managed and treated based on their type and severity.<br>Subjects who discontinue treatment for any reason (including those who withdraw due to intolerable AEs, or those who are unable to continue treatment due to disease progression) will be followed up for short-term safety and efficacy within 28 days after the first dose.

	The study is deemed complete when the last visit is completed for the last subject or the last data point is collected for statistical analysis, whichever occurs later. The last subject refers to the last case planned to be enrolled, or the last case actually enrolled if the COVID-19 epidemic has ended and no additional cases meet the enrollment criteria and do not meet the exclusion criteria.
**Inclusion/Exclusion Criteria**	**Inclusion Criteria (subjects must meet all of the following criteria):**<br>(1) Males or females aged 18–80 (both inclusive);<br>(2) Subjects diagnosed with SARS-CoV-2 infection in the laboratory by PCR nucleic acid amplification test (within 72 h before the first dose);<br>(3) Patients diagnosed with severe COVID-19 as per the *Diagnosis and Treatment Protocol for COVID-19 Patients (Tentative 9th Version)* issued by NHC;<br>(4) Subjects who agree to take effective non-drug contraceptive measures from signing the ICF to 3 months after the end of the study;<br>(5) Subjects who are capable of comprehending the study, willing to participate in the study and sign the ICF (for incapable subjects whose participation is deemed beneficial by the investigator, their legal guardians may sign the ICF on their behalf, with explanations recorded in relevant documents such as original medical records).<br>**Exclusion Criteria (subjects who meet any of the following criteria will be excluded):**<br>(1) Subjects with any abnormal physical examination findings, abnormal laboratory tests and/or any medical history that, in the judgment of the investigator, may compromise their safety by participating in the study;<br>(2) Patients diagnosed with critical SARS-CoV-2 infection as per the *Diagnosis and Treatment Protocol for COVID-19 Patients (Tentative 9th Version)* issued by NHC;<br>(3) Subjects with stage 4 severe chronic kidney disease or requiring dialysis (estimated glomerular filtration rate [eGFR] <30 mL/min/1.73 m^2), or an increase in serum creatinine of 44.2 μmol/L within 7 days, or oliguria (<400 mL/24 h) or anuria (<100 mL/24 h);<br>(4) Pregnant or lactating women;<br>(5) Subjects who will be transferred to a hospital that is not a participating site of the study within 72 hours;

	(6) Patients known to be allergic to the study drug and its components;<br>(7) Subjects who have used anti-cancer drugs, anti-rejection drugs after a transplant, or immunomodulatory biologics (within 30 days or 5 half-lives [whichever is longer] before enrollment);<br>(8) Long-term use of glucocorticoids at a daily dose equivalent to > 10 mg oral prednisone for more than 3 months (10 mg oral prednisone every other day is allowed);<br>(9) Subjects who have used live vaccines (live attenuated vaccines) within 2 weeks prior to randomization or during the study treatment and safety follow-up period;<br>(10) Subjects who are participating in another clinical study. They must undergo a washout period of 5 half-lives (depending on the investigational product used or 30 days from their participation in the previous study, whichever is longer);<br>(11) Subjects with total bilirubin (TBL) > 2 × upper limit of normal (ULN), or alanine aminotransferase (ALT) > 5 × ULN, or aspartate aminotransferase (AST) > 5 × ULN, or alkaline phosphatase > 5 × ULN;<br>(12) Subjects with platelet count < 50 × 10^9/L, or hemoglobin < 70 g/L;<br>(13) Patients with other factors deemed by investigators as not suitable for the trial.<br>*Note: The investigator should ensure that subjects meet all inclusion criteria and do not meet any exclusion criteria at screening. Subjects will be excluded from the study if their status (including laboratory results) changes after screening but before the first dose, causing them to meet any exclusion criteria.*
**Study Drug**	**Name of Study Drug:**<br>Meplazumab for Injection<br>**Method of Administration:**<br>Dissolve Meplazumab for Injection in 1 mL of sterile water for injection and add the resulting solution to 100 mL of 0.9% sodium chloride for IV drip over 30–60 min. (Placebo: 100 mL of 0.9% sodium chloride, IV drip.)<br>**Dosage Design:**<br>A placebo group and a test group will be set up for the clinical study, following the treatment regimen below: a single IV infusion of 0.2 mg/kg Meplazumab or placebo on the first day (D0) and D7 after the first dose during the treatment period, calculated based on subjects' body weight.
**Rationale for Dose**	The dose for the clinical study is determined as 0.2 mg/kg according to the

**Selection**	stage 1 clinical study report of an international multicenter phase II/III study (final report dated July 15, 2022) and the recommendations made by the Independent Data Monitoring Committee (IDMC) on December 19, 2021 based on interim analysis results (January 26, 2022)
**Efficacy Evaluation**	**Primary Measurements**<br>1) All-cause mortality on D28<br>**Secondary Measurements**<br>2) Discharge rate on D28<br>3) Mortality on D28 and D56<br>4) Time to sustained recovery (days; time range: D0 to D28), defined as the first day at which one of the following is achieved: 1. discharge from hospital; 2. hospitalization without oxygen supplementation requirement<br>5) Number of days with oxygen supplementation (days)<br>6) Number of days with mechanical ventilation (days)<br>7) Incidence and duration of re-ventilation 24 hours after withdrawal (days)<br>8) ICU and hospitalization time (days)<br>9) SARS-CoV-2 nucleic acid negative conversion rate on D7, D14, and D28<br>10) Proportion of subjects with improvement in lung imaging (defined as any of the following changes from baseline in lung imaging during the study: 1) reduction in lesion diameter; 2) decrease in the number of lesions; 3) decrease in the number of lung lobes involved by lesions [3, 4])<br>11) Changes from D0 (pre-dose) in the levels of cytokines and chemokines associated with inflammatory and immune states on D1, D4, D7 (before the second dose), D8, D10 and D28, including CRP, IL-2, IL-4, IL-6, INF-α, IL-8, IL-10, IL-12p70, IL-17, IL-1β, TNF-α and IFN-γ<br>12) Changes from D0 (pre-dose) in lymphocyte subsets CD3+, CD4+ and CD8+ on D1, D4, D7 (before the second dose), D8, D10 and D28
**Evaluation of Safety and Immunogenicity**	All subjects will be observed and recorded for any AEs/serious adverse events (SAEs) occurring during the study, including clinical symptoms, clinically relevant abnormal physical signs, abnormal laboratory tests and abnormal electrocardiogram (ECG) findings. The correlation between these events and the study drug will be judged. AEs will be followed up until they are recovered/returned to normal, returned to baseline levels, stable or

	deemed no longer necessary for follow-up based on clinical judgment. Besides, AEs will be evaluated as per CTCAEv5.0.<br>Infusion safety will be monitored before and after each dose by testing blood pressure (systolic and diastolic), pulse rate and blood oxygen saturation SpO_2 or oxygenation index, respiration rate and body temperature before each dose (within 30 min) and at 1 h ± 10 min, 6 h ± 1 h and 12 h ± 1 h after each dose. Hematology, blood chemistry, urinalysis, chest imaging, ECG and other indicators will be measured post-dose in the treatment period.
**Statistical Methods**	**Analysis Population Set:**<br>Intent-to-Treat (ITT) Set: All subjects who underwent randomization.<br>Full Analysis Set (FAS): The population of all subjects dosed after enrollment and randomization.<br>Modified Intention-to-Treat (mITT) Set: All subjects who are randomized and receive at least 1 dose of the study drug (Meplazumab or placebo) will be included in the mITT group.<br>Safety Set: All subjects who are randomized and receive at least 1 dose of the study drug (Meplazumab or placebo) will be included in the SS.<br>**General Principles for Statistical Analysis:**<br>The statistical analysis will be performed using SAS 9.4 or higher version.<br>The baseline result is defined as the last non-missing test result prior to the first dose of Meplazumab.<br>Statistical description will be performed using continuous variables, including number of cases (non-missing), mean, standard deviation (SD), median, maximum, and minimum. The number and percentage of patients in each category will be calculated as categorical variables. Unless otherwise specified, the number of missing subjects will not be included in the calculation of percentage.<br>**Efficacy Analysis:**<br>The analysis of all-cause mortality at Day 28 will be performed between the 0.2 mg/kg dose group and the control group using Cochran-Mantel-Haenszel (CMH) statistics, with stratification by age group (<65 years vs. ≥65 years). The *P* values associated with the CMH statistics will be compared at an alpha level of 0.05 for a 2-sided test.<br>Sensitivity analyses will be performed on all-cause mortality at D28 using logistic regression that includes treatment, baseline, interaction between

	baseline and treatment, and age group (<65 years vs. ≥65 years), with the interaction between age group and treatment as a fixed effect. The treatment efficacy, 95% confidence interval (CI), and *P* value will be evaluated based on the model.<br>Subgroup analysis can be performed by age group, gender, and concomitant antiviral drugs.<br>**Safety Analysis:**<br>Pooled analyses will be performed for treatment-emergent AEs (TEAEs). TEAEs are defined as AEs occurring after the first dose. TEAEs will be summarized by SOC and PT to calculate the number of subjects and incidence under each category.<br>Safety evaluation parameters such as laboratory tests, vital signs and ECG findings will be summarized with descriptive statistics. Changes from baseline in these parameters will be summarized and classified as normal or abnormal (with or without clinical significance). The number and proportion of subjects under each category will be calculated.

Table 1 Study Schedule

Assessments/Procedures	*Screening*	*Treatment*					End of Treatment
**Visit Time**	**D-3 to D-1**	**First Dose (D0)**	**QD Until Discharge**	**D7 Post-Dose**	**D14[a] ± 3 Days Post-Dose**	**D28[a] ± 3 Days Post-Dose**	**D56[a] ± 7 Days Post-Dose**
**Enrollment**							
Informed consent	X						
Demographic and medical history [b]	X						
Date of Symptom Onset	X						
Inclusion/Exclusion Criteria	X						
SARS-CoV-2 result review; positive confirmation	X						
**Study Intervention**							
Randomization		X					
Dosing with Meplazumab or placebo; subjects will be weighed prior to each dose on each dosing day to determine accurate dosage		X		X[c]			

Assessments/Procedures	*Screening*	*Treatment*					End of Treatment
**Visit Time**	**D-3 to D-1**	**First Dose (D0)**	**QD Until Discharge**	**D7 Post-Dose**	**D14[a] ± 3 Days Post-Dose**	**D28[a] ± 3 Days Post-Dose**	**D56[a] ± 7 Days Post-Dose**
SoC until discharge at the discretion of treating physician		X					
**Study Procedures**							
Admission and discharge dates [d]	X	X	X				
COVID-19 diagnosis date	X						
Physical examination [e]	X						
Lung imaging (CT scan)[f]	X	X (pre-dose)		X (before the second dose)	X		
Vital signs, including SpO_2/oxygenation index [g]		X[i]	X	X	X	X	
- Concurrent medications	X	X[i]	X	X	X	X	X
AE evaluation	X	X	X	X	X	X	X
Drug infusion safety monitoring [h]		X		X			
Survival status (dead or not)		X	X			X	X
Number of days with low-flow oxygen supplementation Nasal cannulas and simple masks	X	X	X			X	
Number of days with high-flow oxygen supplementation Venturi masks and high-flow nasal cannulas	X	X	X			X	
Number of days with invasive mechanical ventilation		X	X			X	
Number of days without invasive mechanical ventilation		X	X			X	
**Safety Laboratory**							
Urinalysis [j]	X		X (within 3 days before discharge)				

Assessments/Procedures	*Screening*	*Treatment*					End of Treatment
**Visit Time**	**D-3 to D-1**	**First Dose (D0)**	**QD Until Discharge**	**D7 Post-Dose**	**D14[a] ± 3 Days Post-Dose**	**D28[a] ± 3 Days Post-Dose**	**D56[a] ± 7 Days Post-Dose**
Safety hematology, blood chemistry and coagulation [j]	X[k]	X[i,]	If hospitalized, on D2, D4, D7, D10 (± 1 d) and the day of discharge (± 1 d)			X	
12-lead ECG		X[i]				X	
Blood pregnancy test for women of childbearing potential	X						X
Hepatitis and serological tests (HIV, HBV, HCV)	X						
**Study Laboratory**							
Blood collection for pharmacodynamic (PD) assessment [l]		X	X[l]	X[l]		X	
ADA assessment		X[i]				X	X
Nasopharyngeal swab collection for nucleic acid testing [m]		X[i]	On D2, D4, D7 (before the second dose), D9, and thereafter every 48 h until discharge [m]		X[m]	X[m]	X[m]

Abbreviations: CT = computed tomography; ECG = electrocardiogram; HBV = hepatitis B virus; HCV = hepatitis C virus; HIV = human immunodeficiency virus.

a. Subjects are required to undergo scheduled visits on site. However, if a subject is unable to return to the study site due to epidemic quarantine or other factors, post-discharge remote or home visits are acceptable.

b. Medical history records: COVID-19 history and other past medical histories, such as chronic lung disease (including asthma), chronic kidney disease, diabetes mellitus, heart disease, hypertension, autoimmune diseases, tumors and organ transplantation.

c. If a subject has been discharged before dosing on D7, no more dosing is required on D7.

d. Dates to be recorded: The dates of admission to and discharge from ICU, the dates of re-admission to and re-discharge from ICU (if available), and the dates of admission to and discharge from hospital.

e. Physical examination: Appearance, eyes, ears, nose, throat, thyroid/neck, lymph nodes, heart, lungs, gastrointestinal tract, abdomen, skeletal muscle/extremities, skin, and neurology/psychiatry.

f. Chest radiography (CT scan) will be performed at screening or D0 (predose), D7 (before the second dose), and D14. Additional CT scans may be performed as needed depending on the changes in the subject's conditions.

g. Body temperature, pulse rate, respiratory rate, blood pressure (systolic and diastolic) and blood oxygen saturation (SpO_2) or oxygenation index.

h. Drug infusion safety monitoring: Vital signs, including blood pressure (systolic and diastolic), pulse rate, blood oxygen saturation SpO_2 or oxygenation index, respiration rate and body temperature, will be measured before each dose (within 30 min) and 1 h ± 10 min, 6 h ± 1 h and 12 h ± 1 h after each dose during the treatment period.

i. Baseline assessments should be performed prior to dosing of study drug.

j. Safety hematology, blood chemistry, coagulation and urinalysis:

- Urinalysis: White blood cells (WBC), red blood cells (RBC), pH and protein;
- Hematology: WBC, RBC, hemoglobin (HGB), platelet (PLT) count, absolute lymphocytes, absolute neutrophils, absolute monocytes, absolute eosinophils, absolute basophils, hematocrit (HCT), mean corpuscular volume (MCV), mean corpuscular hemoglobin (MCH), and mean corpuscular hemoglobin concentration (MCHC);
- Blood chemistry: Liver function (total protein, albumin, total bilirubin [TBL], direct bilirubin, indirect bilirubin, alanine aminotransferase [ALT], aspartate aminotransferase [AST], alkaline phosphatase and gamma-glutamyl transferase); renal function (urea, creatinine, uric acid, cystatin C); lactate dehydrogenase, cardiac troponin, D-dimer and ferritin; blood lipids (triglyceride, high-density lipoprotein, low-density lipoprotein and total cholesterol); postprandial glucose; electrolytes (sodium, potassium, chloride and calcium);
- Coagulation: Prothrombin time, activated partial thromboplastin time, fibrinogen and thrombin time;

k. Laboratory tests will be performed 72 h before the first dose to determine enrollment eligibility.

l. PD endpoints: CRP, IL-2, IL-4, IL-6, INF-α, IL-8, IL-10, IL-12p70, IL-17, IL-1β, TNF-α and IFN-γ, as well as lymphocyte subsets CD3+, CD4+ and CD8+. Blood samples will be collected at the following time points, when available: pre-dose and on D1, D4, D7 (before the second dose), D8, D10 and D28 (optional).

m. If the first nucleic acid test is negative, a re-test is required at least 24 hours later. If a subject is tested negative for nucleic acid before discharge (two consecutive negative results, at least 24 hours apart), no more nucleic acid testing is required at subsequent visits; if a subject does not achieve negative conversion before discharge, nucleic acid testing must continue at subsequent visits.

1 Introduction

Coronaviruses are a group of enveloped single-stranded positive-sense RNA viruses that can infect both humans and a variety of vertebrates. They are important pathogens responsible for common colds and upper respiratory tract infections in humans. In recent years, several highly contagious and pathogenic coronaviruses have emerged successively, including SARS-CoV in 2002, MERS-CoV in 2012 and SARS-CoV-2 at the end of 2019. The emergence of these novel coronaviruses poses a serious threat to public health, leading to a progressive decrease in respiratory function and even death in some subjects. The coronavirus disease 2019 (COVID-19) has become a major public health event worldwide. As the global pandemic is still spreading and has the potential to persist for a long time, especially four variants (N501Y, N501Y-D614G, B.1.1.7 (UK), B.1.351 (South Africa)) have recently been prevalent worldwide. To date, the B.1.1.7 variant has spread globally and the E484K mutation has also emerged in the United Kingdom and the United States. It is estimated that the sensitivity of this E484K mutant virus to immune sera from individuals vaccinated with Pfizer Biotechnology's genetic vaccine is reduced by about 6-fold, and its susceptibility to convalescent sera is reduced by about 11-fold; in addition, the B.1.1.7 strain has become widespread in 33 states of the United States, spreading at an unexpected speed. The B.1.351 and P.1 variants, also known as 501Y.V2 and 501Y.V3, are present in South Africa and Brazil, respectively, with three important mutation sites N501Y, E484K and K417N/T resulting in significantly reduced susceptibility to vaccine-induced and convalescent sera. Concerningly, there is evidence of convergent evolution with mutations such as N501Y and E484K that occur not only in these mutants but also in other variants of concern such as P3 in the Philippines, B.1.526 in the United States, and B.1.525 in the United Kingdom and West Africa. India has seen its second wave of COVID-19 since March 2021, setting a world record for more than 400,000 new confirmed cases in a single day. Gene sequencing was performed for 13,000 samples in India, of which 3,532 had "mutations of concern". The risk of transmission and spreading of COVID-19 in China will also persist. At present, the treatment of coronavirus disease 2019 (COVID-19) is mainly supportive symptomatic treatment and antiviral treatment. Specific drugs are still not available. There is an urgent need to develop new treatment methods and drugs for coronavirus infection. In this study, on the basis of previous work, a clinical treatment

study on COVID-19 will be carried out. CD147 molecule with independent intellectual property rights will be used as the target, and humanized Meplazumab targeting CD147 molecule will be used to block the binding between SARS-CoV-2 and target cells, so as to obtain innovative treatment means and new strategies for pneumonia caused by infection with the virus.

1.1 SARS-CoV-2

Coronaviruses are a class of single-stranded positive-sense RNA viruses that can infect humans and many vertebrates, and are important pathogens causing the common cold. Up to now, there are seven known human coronaviruses (including the newly discovered SARS-CoV-2), three of which have high transmission rates and high case fatality rates, namely the Severe Acute Respiratory Syndrome Coronavirus (SARS-CoV-1) that caused the outbreak in Guangdong Province, China in 2002, the Middle East Respiratory Syndrome Coronavirus (MERS-CoV) that caused the outbreak in Saudi Arabia in 2012, and the novel coronavirus (SARS-CoV-2) that caused the outbreak in Wuhan, China at the end of 2019. They are all β coronaviruses. Symptoms of MERS typically include pyrexia, cough and tachypnoea, or even pneumonia, with a case fatality rate of about 34.4%. Symptoms of SARS typically include pyrexia, chills and body pain, or even pneumonia, with a case fatality rate of about 9.6%. Coronaviruses contain at least four structural proteins: S (spike), E (envelope), M (membrane), and N (nucleocapsid) proteins. S protein promotes host attachment and virus fusion with cell membrane during viral infection, while N protein is mainly involved in maintaining the stability of the genome and the formation of nucleocapsid.

1.2 CD147 Target

CD147 was first discovered by Biswas in 1982 and named as tumor cell collagenase stimulatory factor. With the progress of studies, it was successively named extracellular matrix metalloproteinase inducer (EMMPRIN), M6, HAb18G (obtained by the study team in liver cancer tissue screening), etc. The 6th International Workshop and Conference on Human Leukocyte Differentiation Antigens gave different names from various laboratories a new number, CD147. CD147 is a member of the immunoglobulin superfamily, a type I transmembrane glycoprotein highly expressed in cancer cells and in various tumor tissues, including liver cancer, glioblastoma, lung cancer, breast cancer, lymphoma, squamous cell carcinoma of the oral cavity, melanoma, bladder cancer and kidney cancer. It is a

broad-spectrum tumor-associated antigen [5]. Also, CD147 expressed by host cells is an important receptor or coreceptor for various pathogens to invade target cells. It has been reported in the literature that CD147 can mediate infection of hosts by human cytomegalovirus [6], HIV [6], measles virus [7] etc. Our previous studies have shown that the S protein of SARS-CoV-2 can interact with CD147. The S protein recognizes the CD147 receptor on host cells and the virus invades host cells by endocytosis, suggesting that CD147-SP is a new pathway for COVID-19 to invade host cells [8]. In addition, CD147-CyPA is also a receptor-ligand interaction molecule that binds to CyPA through extracellular Pro180 and Gly181 sites, mediating chemotaxis of inflammatory cells and cytokine storm. We also identified the CD147-CyPA interaction, which played an important role in coronavirus infection of host cells [9].

Based on the CD147 overexpression, knockdown/knockout cell models, and the humanized CD147 (hCD147) transgenic mouse model with SARS-CoV-2 infection, we demonstrated *in vitro* and *in vivo* that CD147 mediated SARS-CoV-2 and its variants to infect host cells. CD147 is one of the routes of SARS-CoV-2 infection [8].

After stably interfering with CD147 expression in Vero E6 and BEAS-2B cells, the ability of SARS-CoV-2 to infect them was significantly reduced by 2.08 and 5.17 folds, respectively; however, overexpression of CD147 in BEAS-2B and BHK-21 cells enhanced the viral infection capacity by 3.99 and 3.96 folds respectively.

Compared with wild-type Vero E6 cells, the content of SARS-CoV-2 wild strain (Wuhan strain) and its variants B.1.1.7 strain (501Y.V1) and B.1.351 strain (501Y.V2) in CD147 knockout Vero E6 cells was significantly down-regulated by 3.79, 2.07 and 3.99 folds, respectively.

The drug R&D team found for the first time that the host cell receptor CD147 interacts with SARS-CoV-2 S protein and is a new receptor for SARS-CoV-2 to invade host cells. SARS-CoV-2 invades host cells through CD147-dependent receptor-mediated endocytosis, which is closely related to the regulation of Arf6. CD147 provides a key target for the development of specific drugs for COVID-19.

Based on the previous work, we will carry out a clinical study on the treatment of pneumonia caused by infection with SARS-CoV-2 and its variants. The receptor CD147 molecule found by our team for the first time will be used as the target, and humanized Meplazumab targeting CD147 molecule will be used to block SARS-CoV-2 epidemic strains and Alpha, Beta, Gamma,

Delta and Omicron strains from infecting the cell, without being affected by viral mutations. The treatment is expected to have strong inhibition on infection invasion and viral replication for all these strains. The clinical trials of this product were conducted in healthy volunteers and patients with severe and moderate COVID-19. Five clinical trials, including Phase I clinical trials [10], exploratory Phase II clinical trial [10], international multicenter Phase II/III clinical trial, and China multicenter Phase II/III clinical trial, have confirmed that the drug has good safety, can effectively reduce the mortality rate of severe/critical patients and increase the discharge of severe/critical patients, improve the disease improvement rate (reduce the clinical severity by two levels), inhibit viral replication, shorten the time to virus negative conversion, improve the early virus negative conversion rate and early discharge rate of patients with mild and moderate disease. Also, studies have confirmed that this antibody can also block the interaction between CD147 and the pro-inflammatory factor CyPA, and reduce the occurrence of cytokine storm in the body [11], with clear pharmacological effects. On December 8, 2022, "Meplazumab for Injection", an anti-COVID-19 specific receptor blocking antibody drug targeting CD147, was approved for production, becoming the first receptor blocking drug approved for the treatment of severe and critical COVID-19 in the world.

1.3 Meplazumab for Injection

Meplazumab for Injection is a new humanized monoclonal antibody drug jointly developed by the National Translational Science Center for Molecular Medicine, Fourth Military Medical University and Jiangsu Pacific Meinuoke Bio-pharmaceutical Co., Ltd. Meplazumab is a recombinant humanized IgG2 antibody expressed in CHO cells. We replaced the FR sequences in the variable regions of light and heavy chains of the antibody with human-derived FR sequences using bioinformatics method and recombinant technology, and more than 2/3 of the entire molecule was human-derived. The variable region had the function of binding to antigens, while the constant region had the antibody effector function, immunogenicity, and species characteristics. The Fc segment of the chimeric antibody could prolong the half-life of the antibody in serum, theoretically reducing the immunogenicity of heterologous antibodies. The equilibrium dissociation constant of the humanized antibody did not change significantly compared with its parental non-humanized monoclonal antibody, and the affinity constant (K_D) was 1.7×10^{-10} M, indicating that Meplazumab had similar affinity to its parental non-humanized monoclonal antibody 6H8 (affinity constant $K_D = 4.48 \times 10^{-10}$ M), ensuring the

ability of this monoclonal antibody to bind to target molecules *in vitro* and *in vivo*.

1.4 Mechanism of Meplazumab for Injection in the Treatment of COVID-19

Coronaviruses have at least four structural proteins: S, E, M and N proteins. S protein is the main component of nucleocapsid. In recent years, it has been found that CyPA can bind to the N protein of coronavirus and play an important role in viral replication, assembly, release and infection. In particular, CyPA is not only directly involved in the budding and release of progeny viruses, but also presents on the surface of the viral membrane after viral release, and participates in the recognition and binding process between coronaviruses and target cells[9, 12].

Our recent studies have shown that the S protein of SARS-CoV-2 can interact with CD147. The S protein recognizes the CD147 receptor on host cells and the virus invades host cells by membrane fusion, suggesting that CD147-SP is a new pathway for COVID-19 to invade host cells [8]. In addition, CD147-CyPA is also a receptor-ligand interaction molecule that binds to CyPA through extracellular Pro180 and Gly181 sites, mediating chemotaxis of inflammatory cells and cytokine storm. In the previous work, we also identified the CD147-CyPA interaction, which played an important role in coronavirus infection of host cells [11]. Several domestic and international patents have been filed for relevant contents [13-18].

Studies showed [19, 20] that the expression of CD147 in bronchoalveolar lavage fluid or lung tissue was elevated in patients with pulmonary interstitial fibrosis; in patients with UIP-IPF, the expression of CD147 in macrophages and alveolar epithelial cells in fibrotic foci areas was elevated, suggesting that CD147 played an important role in the occurrence and development of pulmonary fibrosis. Our previous study showed [21] that anti-CD147 antibody significantly reduced the collagen score and improved pulmonary interstitial fibrosis in a mouse model of bleomycin-induced pulmonary interstitial fibrosis. The team's latest study found that CD147 is a key regulator of fibroblast activation during pulmonary fibrosis, in addition to mediating SARS-CoV-2 invasion and inducing cytokine storm. Meplazumab inhibited the accumulation of activated fibroblasts and the production of extracellular matrix proteins, reducing the progression of pulmonary fibrosis caused by SARS-CoV-2 [22].

CD147 is expressed in T cells, mononuclear macrophages, dendritic cells, B cells, and other cells associated with various inflammatory diseases, and the degree of progression of inflammatory diseases is closely related to the expression level of CD147. Our previous studies

showed that, in patients with rheumatoid arthritis, the expression of CD147 on synovial fibroblasts, neutrophils and mononuclear macrophages was significantly increased. When chemotaxis of these cells reached the inflammatory site, CD147 promoted the expression of a large number of matrix metalloproteinases in the diseased site, which ultimately increased the rate of cartilage destruction in rheumatoid arthritis [23-25]. Treatment of rheumatoid arthritis mouse model with CD147 monoclonal antibody significantly inhibited the secretion of various inflammatory-related factors and MMPs by synovial fibroblasts, thus attenuating their destruction to implanted synovial cartilage.

In summary, the main pharmacological mechanism of this product is as follows:

CD147 is a universal receptor for SARS-CoV-2 to infect humans, mediating the infection with SARS-CoV-2 epidemic strains and variants Alpha, Beta, Gamma, Delta, and Omicron (BA.1 and BA.2) in humans. Meplazumab can directly bind to CD147 and block the interaction between CD147 and SARS-CoV-2 S protein, thus inhibiting SARS-CoV-2 infection. In the model of human CD147 transgenic mice infected with SARS-CoV-2 epidemic strains and variants Alpha, Beta and Omicron BA.1, Meplazumab reduced viral load and inhibited the pathological characteristics of exudative alveolitis; in international multicenter clinical trials for severe COVID-19, Meplazumab had antiviral effects of reducing viral load and increasing negative rate and negative conversion rate.

CD147 plays a core regulatory role in the cytokine storm induced by SARS-CoV-2 and mediates the cytokine storm (inflammatory storm) caused by CyPA upregulation. Meplazumab blocks the interaction between CD147 and CyPA by binding to CD147, and has an anti-inflammatory effect of inhibiting various cytokine storms caused by SARS-CoV-2 infection.

2 Study Objectives

To evaluate the safety and efficacy of Meplazumab for Injection when added to the standard of care, compared to the standard of care alone, in severe COVID-19 patients.

3 Study Design and Principle

3.1 Overall Design

This is a multicenter, double-blind, randomized, placebo-controlled add-on Phase III clinical trial to evaluate the safety and efficacy of Meplazumab for Injection in severe COVID-19 patients. Neither the subject nor the investigator will know what is used (study drug or placebo), as the study drug/placebo will be prepared by an unblinded third party (e.g., pharmacist or nurse), and will be given by authorized blinded study site personnel.

Hospitalized patients with laboratory-confirmed SARS-CoV-2 infection will be included in the study. Subjects will be randomly stratified by age group (age <65 years or ≥ 65 years).

Subject recruitment will continue throughout the study until the total number of subjects planned to be randomized into the study is reached. Subjects will be randomized into either the treatment group or control group.

During hospitalization, all subjects will receive SoC matching COVID-19 hospitalization based on the medical judgment of the investigator.

A total of 350 subjects will be randomized in a 1:1 ratio to receive Meplazumab at 0.2 mg/kg or placebo.

3.2 Overview of Study Design

This study is a multicenter, double-blind, randomized, placebo-controlled add-on clinical trial to evaluate the safety and efficacy of Meplazumab + SoC vs. SoC by intravenous infusion in severe COVID-19 patients.

Approximately 350 severe COVID-19 patients will be enrolled.

3.3 Principle of Study Design

Given the rapidly evolving SoC for COVID-19, the primary treatment is supportive treatment and based on the medical judgment of the physician treating the subject. At present, there are few reports of positive study results on COVID-19; however, based on early analysis of adaptive COVID-19 treatment trial (ACTT) data, Remdesivir received emergency use authorization from the United States Food and Drug Administration (FDA) for treating subjects

with suspected or laboratory-confirmed SARS-CoV-2 infection and severe COVID-19. The reported results showed that subjects with "moderate" infection recovered faster after 5 days of Remdesivir treatment.

The United Kingdom RECOVERY trial evaluated 28-day mortality in hospitalized patients with COVID-19 who received a low dose of dexamethasone (6 mg) daily orally or IV for 10 days in addition to their conventional treatment. In the dexamethasone group, mortality was lower in patients who received invasive mechanical ventilation (29.3% vs. 41.4%) and patients with oxygen supplementation who did not receive invasive mechanical ventilation (23.3% vs. 26.2%) than in the standard care group. Results from the RECOVERY trial and other similar studies suggested that low-dose glucocorticoids may be beneficial in treating hospitalized COVID-19 patients, and clinicians considered this therapy.

Following the discovery of SARS-CoV in 2002, which caused a large global outbreak, there has been growing interest in developing specific treatment drugs. SARS-CoV subjects were treated with corticosteroids, interferon type 1 (IFN) preparations, convalescent patient plasma, ribavirin, lopinavir or ritonavir, and, except for ribavirin, many drugs were provided with in vitro pre-clinical data supporting their efficacy. Since the outbreak of SARS, novel therapeutic drugs (targeting viral access proteins, proteases, polymerases and methyltransferases) have passed tests, however, they have not yet demonstrated efficacy in clinical trials.

As this study is a multicenter, randomized, third-party blinded study, rigorous data on the safety and efficacy of Meplazumab in the treatment of COVID-19 will be obtained in addition to detailed information on an important subpopulation of COVID-19 that develops into respiratory disease before requiring mechanical ventilation.

Randomization and inclusion of the control group are essential to control unexpectedly introduced biases in study results, and are necessary to determine the safety and efficacy of Meplazumab for COVID-19 treatment. The use of a 1:1 drug-control randomization ratio is more conducive to obtaining potentially effective treatment outcomes. Clinical and virological data from enrolled subjects collected using standardized timelines and collection tools will provide valuable information on the clinical course and incidence of severe COVID-19 in different cohorts of hospitalized adult subjects.

All study personnel responsible for decisions related to the study will be blinded by third parties in this study. A third-party blinding design is important to provide an objective assessment of

the pharmacological effects of the study drug and minimize the impact of bias in the assessment.

4 Study Population

Patients diagnosed with severe COVID-19 as per the *Diagnosis and Treatment Protocol for COVID-19 Patients (Tentative 9th Version)* issued by NHC.

4.1 Inclusion Criteria

(1) Males or females aged 18–80 (both inclusive);

(2) Subjects diagnosed with SARS-CoV-2 infection in the laboratory by PCR nucleic acid amplification test (the test results must be obtained within 72 h before the first dose);

(3) Patients diagnosed with severe COVID-19 as per the Diagnosis and Treatment Protocol for COVID-19 Patients (Tentative 9th Version) issued by NHC;

(4) Subjects who agree to take effective non-drug contraceptive measures from signing the ICF to 3 months after the end of the study;

(5) Subjects who are capable of comprehending the study, willing to participate in the study and sign the ICF (for incapable subjects whose participation is deemed beneficial by the investigator, their legal guardians may sign the ICF on their behalf, with explanations recorded in relevant documents such as original medical records).

4.2 Exclusion Criteria

Those who meet one or more of the following conditions will not be included in the study:

(1) Subjects with any abnormal physical examination findings, abnormal laboratory tests and/or any medical history that, in the judgment of the investigator, may compromise their safety by participating in the study;

(2) Patients diagnosed with severe COVID-19 as per the *Diagnosis and Treatment Protocol for COVID-19 Patients (Tentative 9th Version)* issued by NHC;

(3) Subjects with stage 4 severe chronic kidney disease or requiring dialysis (estimated glomerular filtration rate [eGFR] <30 mL/min/1.73 m^2), or an increase in serum creatinine of 44.2 μmol/L within 7 days, or oliguria (<400 mL/24 h) or anuria (<100 mL/24 h);

(4) Pregnant or lactating women;

(5) Subjects who will be transferred to a hospital that is not a participating site of the study within 72 hours;

(6) Patients known to be allergic to the study drug and its components;

(7) Subjects who have used anti-cancer drugs, anti-rejection drugs after a transplant, or immunomodulatory biologics (within 30 days or 5 half-lives [whichever is longer] before enrollment);

(8) Long-term use of glucocorticoids at a daily dose equivalent to > 10 mg oral prednisone for more than 3 months (10 mg oral prednisone every other day is allowed);

(9) Subjects who have used live vaccines (live attenuated vaccines) within 2 weeks prior to randomization or during the study treatment and safety follow-up period;

(10) Subjects who are participating in another clinical study. They must undergo a washout period of 5 half-lives (depending on the investigational product used or 30 days from their participation in the previous study, whichever is longer);

(11) Subjects with total bilirubin (TBL) > 2 × upper limit of normal (ULN), or alanine aminotransferase (ALT) > 5 × ULN, or aspartate aminotransferase (AST) > 5 × ULN, or alkaline phosphatase > 5 × ULN;

(12) Subjects with platelet count < 50 × 109/L, or hemoglobin < 70 g/L;

(13) Patients with other factors deemed by investigators as not suitable for the trial.

Note: The investigator should ensure that subjects meet all inclusion criteria and do not meet any exclusion criteria at screening. Subjects will be excluded from the study if their status (including laboratory results) changes after screening but before the first dose, causing them to meet any exclusion criteria.

5 Treatment Assignment and Blinding

Eligible subjects will be randomized to the Meplazumab group or placebo group in a 1:1 ratio according to the principle of randomization, and subject randomization will be completed using an interactive web response system (IWRS). The randomization statistician will use SAS9.4 or higher version software to generate the Subject Randomization Schedule by stratified blocked randomization. The stratification factor is age (age < 65 years old or ≥ 65 years old), and the schedule will be imported into the IWRS by the system engineer. After the subjects are successfully screened, the authorized investigators will log in to the IWRS to randomize the subjects and generate a randomization number, obtain the treatment group assignment information of the subjects, and collect the drugs of the corresponding groups for

the subjects according to the system prompt to complete drug preparation and injection; in case of drug damage and other conditions, the unblinded investigator may re-collect the drugs for the same group to complete drug preparation and injection for the subjects.

5.1 Study Blindness

In this study, the study drug should be dissolved with sterile water for injection and then mixed with 100 mL of 0.9% normal saline, while the control drug will be a placebo, and it is planned to directly use 0.9% normal saline. Double-blind in the strict sense cannot be achieved due to the different appearances of the two drugs. In this study, the traditional packaging blinding of the investigational product cannot be performed before the trial. To avoid the bias on the study results caused by people obtaining specific grouping information, the blindness assurance work plan in this study is mainly carried out as follows:

1) It is planned to set a blinded team and an unblinded team at the site level to conduct the study. The unblinded study personnel include drug management personnel, personnel logging into the IWRS to obtain treatment grouping information, and drug preparation personnel. The remaining study personnel will remain blinded.
2) The project management and study monitoring team will have blinded and unblinded monitors. The unblinded monitors will perform site-specific drug accountability and monitor whether the subjects' drug preparation and administration process meet the protocol requirements. The remaining study personnel will remain blinded.
3) Drug preparation: Unblinded personnel will log in to the IWRS to obtain treatment grouping information and complete solution preparation before administration. After preparation, the appearance of drugs in both the test group and control group will be consistent to maintain blindness. The drug preparation personnel cannot disclose relevant information about therapeutic drugs to subjects and any other persons, nor participate in all subsequent study evaluation processes.
4) After the start of the trial, both study members in the blinded group and unblinded group should ensure that they strictly follow the protocol requirements to carry out trial operations within their respective responsibilities.

5.2 Emergency Unblinding

When the specific treatment group information of a subject needs to be obtained to treat the subject due to serious adverse events or other emergencies, an authorized investigator can

perform emergency unblinding for the subject through the emergency unblinding module of the IWRS to obtain the specific grouping information of the subject. If possible, prior to unblinding the investigational product, the authorized investigator must notify the Principal Investigator and relevant personnel of the sponsor first, and obtain approval from the Principal Investigator before obtaining specific subject grouping information. If the sponsor is not contacted before emergency unblinding, the investigator must contact the sponsor within 24 hours after unblinding. The investigator should record the date and reason for the unblinding of the subject, as well as the operation process of unblinding in the source documents.

5.3 Blind Review and Unblinding

After all data are entered into the database and confirmed to be correct, the data manager will write a blind data review report and hold a data blind review meeting. At the data blind review meeting, the principal investigator, data manager, statistician, and monitor should confirm any protocol violations in the trial and their impact on the statistical analysis set, make decisions on the problems raised in the data blind review report, and finalize the data blind review report. The database will also be locked according to the process. Once the database is locked, the unblinding procedure can be started. The independent statistician for randomization will release the Subject Randomization Schedule to the project statistician for unblinded data statistical analysis according to the process.

6 Dosage and Method of Administration

6.1 Rationale for Dose Selection

Based on the Topline report of stage I clinical data from an international multicenter Phase II/III clinical study (final report dated July 15, 2022), and the recommendations made by the Independent Data Monitoring Committee (IDMC) based on the interim analysis results dated December 19, 2021 (January 26, 2022), the protocol for determining the clinical efficacy and safety evaluation in hospitalized subjects with COVID-19 infection compared with placebo plus SoC has been studied.

The following experimental study results will be used to justify the doses in this clinical study:

Evaluation of key clinically significant functional efficacy endpoints:

Mortality (D29)

1 (2.4%) in the 0.12 mg/kg group, 6 (14.6%) in the 0.2 mg/kg group, 4 (9.1%) in the 0.3 mg/kg

group, and 6 (14.6%) in the placebo group. Mortality in the 0.3 mg/kg group was reduced by 37.7% compared with the placebo group; mortality in the 0.2 mg/kg group was equal to that in the placebo group; and mortality in the 0.12 mg/kg group was statistically significantly reduced by 83.6% compared with the placebo group (*p*=0.0150).

Proportion of subjects who are alive and not receiving oxygen supplementation at discharge (D29)

34 (82.9%) in the 0.12 mg/kg group, 27 (65.9%) in the 0.2 mg/kg group, 31 (70.5%) in the 0.3 mg/kg group, and 29 (70.7%) in the placebo group. The survival rate of the 0.3 mg/kg group at discharge was comparable to that of the placebo group, and the 0.12 mg/kg group had a statistically significant improvement of 17.3% compared with the placebo group (*p*=0.0337).

Safety Evaluation

The incidence of adverse events in the three dose groups (0.12 mg/kg, 0.2 mg/kg, and 0.3 mg/kg) of Meplazumab was basically comparable to that in the placebo group. No new or unexpected safety findings were found, indicating that this drug had good safety and tolerability in human treatment.

Based on clinical evaluation of the clinical efficacy and safety results, no obvious dose response was observed at the 3 dose levels. Most likely, the sample sizes for all 4 treatment groups were small, resulting in a lack of obvious dose response; however, the reasons may depend on many factors, such as study population, the geographic location of most subjects, and inherent characteristics of the study drug.

While clinical and functional tests and mortality indicated that the 0.12 mg/kg dose performed best with respect to relevant clinical endpoints (D29 mortality and proportion of subjects discharged alive at D29 without oxygen supplementation requirement), this result likely reflected limitations in study design, including the small sample size per treatment group. Therefore, given the potential for inherent variability in data, the variability of subject characteristics between recruited study populations, and IDMC recommendations, 0.2 mg/kg is selected for testing and will be compared with placebo plus SoC.

To sum up, according to the available clinical data reviewed in the topline report, Meplazumab has shown a good clinical benefit in the treatment of severe COVID-19, providing a basis for progression to phase III at the dose of 0.2 mg/kg to further evaluate the clinical efficacy of Meplazumab in the treatment of COVID-19.

6.2 Method of Administration

Dissolve the antibody with 1 mL of sterile water for injection, calculate the dose required based on the dose determined in the previous clinical study (0.2 mg/kg) and the body weight of the patient, add to 100 mL of 0.9% sodium chloride for intravenous drip (use only 100 mL of 0.9% sodium chloride for intravenous drip in the placebo control group), and complete intravenous drip in 30–60 min.

Normal saline is selected as the placebo in this clinical study because there is no approved active control for severe COVID-19 and the study is a controlled study with SoC added.

6.3 End of Study

The study is deemed complete when the last visit is completed for the last subject or the last data point is collected for statistical analysis, whichever occurs later. The last subject refers to the last case planned to be enrolled, or the last case actually enrolled if the COVID-19 epidemic has ended and no additional cases meet the enrollment criteria and do not meet the exclusion criteria.

6.4 Treatment Regimen

The treatment plan includes a screening phase, a treatment phase, and an end of treatment phase. Treatment-related contents in each phase are as follows:

6.4.1 Screening Phase

After the ICF is signed, relevant items will be screened according to the study protocol (refer to the Study Schedule). Subjects who pass the screening will proceed to the next phase.

6.4.2 Treatment Phase

Administration (D0, D7)

- Physical examination: Pre-dose;
- Vital signs: Pre-dose and at 1 hr ± 10 min, 6 ± 1 hr and 12 ± 1 hr postdose;
- Dissolve the antibody with 1 mL of sterile water for injection, calculate the dose required based on the dose determined in the previous clinical study (0.2 mg/kg) and the body weight of the patient, and add to 100 mL of 0.9% sodium chloride for intravenous drip (use only 100 mL of 0.9% sodium chloride for intravenous drip in the placebo control group).

6.4.3 End of Treatment

Subjects who develop a fatal allergic reaction during treatment should be treated

symptomatically and managed in accordance with the emergency plan. Oxygen supplementation, bronchodilators, epinephrine, antihistamines or glucocorticoids should be given for symptomatic treatment. During the treatment, the vital signs of patients should be closely observed and actively evaluated until all indicators of the subject are stable.

7 Medication Compliance

Meplazumab should be used under the supervision of the Principal Investigator or other study personnel. The administration of all study drugs should be recorded in the CRF. The reason(s) for medication interruption, dose reduction, or missing doses will also be recorded in the CRF. Such information and drug accountability of all study drugs in each period will be used to assess treatment compliance.

8 Concomitant Therapy

Concomitant therapies include all drug therapies and obvious non-drug therapies (including physiotherapy and blood or platelet transfusion) used after admission to the end of this clinical study. Drugs include not only those prescribed by doctors, but also all OTC drugs, vitamins, TCM decoctions, Chinese patent medicines and so on. All should be recorded in CRF.

8.1 Permitted Drug Therapies for Subjects During the Study

On the premise of protecting the interests and safety of subjects, the investigators should continue to provide standardized treatment for subjects in accordance with the requirements of the *Diagnosis and Treatment Protocol for COVID-19 Patients (Tentative 9th Version)* issued by the National Health Commission during the study. Necessary drugs and necessary therapies can also be used according to the patient's condition. The information on concomitant medications received by subjects during this study and symptomatic treatment should be recorded in the CRF.

8.2 Contraindicated Medications for Subjects During the Study

The investigators should continue to provide standardized treatment for subjects in accordance with the requirements of the *Diagnosis and Treatment Protocol for COVID-19 Patients (Tentative 9th Version)* issued by the National Health Commission during the study. If serious adverse reactions occur during treatment, they will be handled according to relevant clinical guidelines.

9 Study Evaluation

9.1. Efficacy Measurements

Primary Measurement

(1) All-cause mortality at D28

Secondary Measurements

(2) Discharge rate on D28 postdose

(3) Mortality on Days 14 and 56 postdose

(4) Time to sustained recovery (days; time range: D0 to D28), defined as the first day at which one of the following is achieved: 1. discharge from hospital; 2. hospitalization without oxygen supplementation requirement

(5) Duration of oxygen supplementation (days)

(6) Duration of mechanical ventilation (days)

(7) Incidence and duration of re-ventilation 24 hours after withdrawal (days)

(8) Length of stay in ICU and hospitalization time (days)

(9) SARS-CoV-2 nucleic acid negative conversion rate on D7, D14, and D28

(10) Proportion of subjects with improvement in lung imaging (defined as any of the following changes from baseline in lung imaging during the study: 1) reduction in lesion diameter; 2) decrease in the number of lesions; 3) decrease in the number of lung lobes involved by lesions [3, 4])

(11) Changes from D0 (predose) in the levels of cytokines and chemokines associated with inflammatory and immune states on D1, D4, D7 (before the second dose), D8, D10 and D28, including CRP, L-2, IL-4, IL-6, INF-α, IL-8, IL-10, IL-12p70, IL-17, IL-1β, TNF-α and IFN-γ

(12) Changes from D0 (predose) in lymphocyte subsets CD3+, CD4+ and CD8+ on D1, D4, D7 (before the second dose), D8, D10 and D28

9.2 Efficacy Evaluation

The following clinical support indicators should be assessed on each study day during hospitalization:

• Hospitalization

• Survival status

• Oxygen demand - Days of low-flow oxygen supplementation (< 40%)/days of high-flow oxygen supplementation (> 40%)

• Non-invasive mechanical ventilation (via mask)

• Invasive mechanical ventilation (via endotracheal intubation or tracheotomy)

• Requirement for extracorporeal membrane oxygenation (ECMO)

Definition of negative nucleic acid conversion: Negative nucleic acid conversion is judged by two consecutive negative nucleic acid tests (sampling time should be at least 24 hours apart), and the sampling time of the first negative nucleic acid test is taken as the time to negative nucleic acid conversion.

9.3 Safety Evaluation

All subjects will be observed and recorded for any AEs/serious adverse events (SAEs) occurring during the study, including clinical symptoms, clinically relevant abnormal physical signs, abnormal laboratory tests and abnormal electrocardiogram (ECG) findings. The correlation between these events and the study drug will be judged. AEs will be followed up until they are recovered/returned to normal, returned to baseline levels, stable or deemed no longer necessary for follow-up based on clinical judgment. Besides, AEs will be evaluated as per CTCAE v5.0.

Infusion safety will be monitored before and after each dose by testing blood pressure (systolic and diastolic), pulse rate and blood oxygen saturation SpO_2 or oxygenation index, respiration rate and body temperature before each dose (within 30 min) and at 1 h ± 10 min, 6 h ± 1 h and 12 h ± 1 h after each dose. Hematology, blood chemistry, urinalysis, chest imaging, ECG and other indicators will be measured post-dose in the treatment period.

10 Early Termination of Study

The sponsor may terminate the study at any time. The termination of the study must be reported to the Independent Ethics Committee (IEC) and Institutional Review Board (IRB). Reasons for early termination of the study include, but are not limited to, the following:

(1) Drug regulatory authority, ethics committee, sponsor, or investigator believes that the therapeutic drug has significant safety risks;

(2) The sponsor may terminate the study for any scientific, medical, or ethical reason, but the rights, safety, and health of subjects must be fully considered;

(3) Other reasons judged by the sponsor or investigator to be unsuitable for continuing the study.

11 Subject Completion/Withdrawal

11.1 Completion

If a subject completes all evaluations within 28 days after the first dose according to the study protocol or dies, the subject will be considered as a subject who has completed the study.

11.2 Early Termination/Withdrawal

Withdrawal Decided by Investigator

Withdrawal from the study refers to a situation where an enrolled subject is deemed unsuitable to continue participation during the course of the study, and the investigator decides to withdraw the subject from the study.

(1) In the clinical trial, the subject experiences some comorbidities, complications or deterioration of the condition and is not suitable for further participation in the study;

(2) Subjects do not comply with the medical advice to use other treatments, which affects evaluation;

(3) The subject experiences adverse events or serious adverse events and is not suitable for further participation in the study;

(4) Poor compliance of subjects, affecting efficacy and safety judgment;

(5) Subject pregnancy;

(6) Other reasons based on which the investigator judges that the subject is not suitable for further participation in the study.

Withdrawal Decided by Subject

According to the informed consent form, if subjects are not willing to continue to participate in the clinical study, they have the right to withdraw at any stage of the study, or if subjects are lost to follow-up due to no further administration and tests, although they do not explicitly request to withdraw, it is also considered as "withdrawal" (or "drop-out"). The reasons for withdrawal should be investigated as much as possible and recorded. For example: subjects are intolerant of some adverse reactions, unable to further participate in the clinical study for other reasons, or lost to follow-up without any explanations.

Handling of Withdrawals

The investigator must fill in the reason for withdrawal in the case report form, contact the patient if possible, complete all assessment items that can be completed, fill in the end-of-treatment follow-up record form, and record the time of the last dose if possible. Those

who withdraw due to adverse events that are finally judged to be related to the therapeutic drug after follow-up must be recorded in the case report form, and the sponsor should be informed.

All existing study-related toxicities and SAEs at the time of withdrawal from the study must be followed up until they are resolved or corresponding indicators return to baseline levels, unless in the opinion of the investigator, remission is unlikely due to the patient's disease.

After the patient terminates the study treatment, the investigator should try to follow up all existing or new AEs that occur within 28 days after the last infusion of Meplazumab. All new AEs and SAEs occurring within this time frame should be reported. SAEs must be reported to the sponsor within 24 hours and followed up until they are resolved or corresponding indicators return to the baseline levels as described above. The Investigator should notify the sponsor immediately after a patient terminates the study. For any SAE, the sponsor should be contacted in time according to the corresponding SAE reporting procedure.

11.3 Case Report Form (CRF)

All CRFs must be completed, modified and replaced by the investigator or his/her authorized personnel. Queries will be generated by the EDC system. The investigator or his/her authorized personnel must answer or correct the queries sent.

11.4 Record Retention

According to ICH-GCP guidelines, the investigator/study site should maintain all CRFs, source documents of data collected from each subject, and all study documents specified in Section 8 of ICH-GCP and current regulations. The investigator/study institution should take measures to prevent these documents from being accidentally or prematurely destroyed.

12 Statistical Analysis

12.1 Statistical and Analytical Plans

All efficacy and safety data of each dose group will be tabulated and summarized according to the statistical analysis plan.

Data will be presented by treatment group.

12.2 Analysis Data Set

FAS: The population of all subjects dosed after enrollment and randomization.

Modified Intention-to-Treat (mITT) Set: All subjects who are randomized and receive at least 1 dose of the study drug (Meplazumab or placebo) will be included in the mITT group.

Intent-to-Treat (ITT) Set: All subjects who underwent randomization.

Safety Set: All subjects who are randomized and receive at least 1 dose of the study drug (Meplazumab or comparator) will be included in the SS.

12.3 General Principles for Statistical Analysis

The statistical analysis will be performed using SAS 9.4 or higher version.

The baseline result is defined as the last non-missing test result prior to the first dose of Meplazumab.

Statistical description will be performed using continuous variables, including number of cases (non-missing), mean, standard deviation (SD), median, maximum, and minimum. Categorical variables will be statistically described using the summary of frequency and percentage. Unless otherwise specified, the number of missing subjects will not be included in the calculation of percentage.

Demographic data and baseline indicators will be analyzed in the FAS population. All demographic variables and baseline characteristics (e.g., gender, date of birth, weight, disease category, concomitant medications, prior therapy history, vital signs, etc.) are summarized by dose group.

12.4 Efficacy Analysis

The analysis of all-cause mortality at Day 28 postdose will be performed between the selected dose group and the control group using Cochran-Mantel-Haenszel (CMH) statistics, with stratification by age group (<65 years vs. ≥65 years). The P values associated with the CMH statistics will be compared at an alpha level of 0.05 for a 2-sided test.

Sensitivity analyses will be performed on all-cause mortality at D28 postdose using logistic regression that includes treatment, baseline, interaction between baseline and treatment, and age group (<65 years vs. ≥65 years), with the interaction between age group and treatment as a fixed effect. The treatment efficacy, 95% confidence interval (CI), and P value will be evaluated based on the model.

Subgroup analysis can be performed by age group, gender, and concomitant antiviral drugs.

12.5 Safety Analysis

Safety analysis will be performed based on the SS.

Pooled analyses will be performed for treatment-emergent AEs (TEAEs). TEAEs are defined as AEs that occur after the first dose of Meplazumab. TEAEs will be summarized by SOC and PT to calculate the number of subjects and incidence under each category.

Safety evaluation parameters such as laboratory tests, vital signs and ECG findings will be summarized with descriptive statistics. Changes from baseline in these parameters will be summarized and classified as normal or abnormal (with or without clinical significance). The number and proportion of subjects under each category will be calculated.

12.6 Interim Analysis

Not considered.

12.7 Determination of Sample Size

Based on the available clinical study results and dose justification in Section 6.1, subjects in the 0.2 mg/kg dose group will enter Phase III clinical trial to compare with placebo + SoC. Previous results showed a mortality of 2.4%, 14.6%, 9.1%, and 14.6% in the 0.12 mg/kg, 0.2 mg/kg, 0.3 mg/kg, and placebo groups, respectively. Given the potential for inherent variability in data, the mortality rate of the placebo group will be estimated at 15%. The mortality rate of the 0.2 mg/kg group is expected to have a 10% reduction compared with the placebo group. The alpha level of the two-sided test will be set at 0.05 and the power at 0.8. nQuery (Version 9.1) will be used for calculation. At least 141 subjects will be required for each group. Considering a dropout rate of 20%, 175 subjects will be required for each group, totaling 350 subjects.

13 Adverse Event Reporting

Timely, accurate and complete reporting and analysis of safety information from clinical studies is critical to the protection of subjects, investigators, and the sponsor, and is also a mandatory requirement for regulatory authorities. The sponsor has established standard operating procedures (SOPs) according to the requirements of *Good Clinical Practice* (No. 57, 2020), *Provisions for Adverse Drug Reaction Reporting and Monitoring* (Ministry of Health Order No. 81), and *Provisions for Drug Registration* (SAMR Order No. 27) to ensure appropriate reporting of safety information.

All clinical studies initiated by the sponsor are required to report AEs in accordance with these SOPs.

13.1 Relevant Definitions

13.1.1 Pretreatment Event

Pretreatment event (PTE): Any untoward medical event in a clinical study subject who signs the ICF to participate in the study, but which occurs before the administration of any study drug; the event does not necessarily have causality with participation in the study.

13.1.2 Definition and Classification of Adverse Events

- **Adverse Events (AEs)**

An adverse event (AE) refers to any adverse medical event, experienced by a subject administered an investigational product, which may present with symptoms, signs, diseases, or laboratory test abnormalities but does not necessarily have a causal relationship with the investigational product. The investigator must report all AEs in the eCRF. All AEs occurring during the study should be recorded in the CRF.

- **Serious Adverse Events (SAEs)**

A serious adverse event (SAE) is any untoward medical event in a subject after administration of the investigational product that meets one or more of the following criteria:

(1) Resulting in death;

(2) Result in life-threatening consequences;

(3) Resulting in hospitalization or prolongation of existing hospitalization;

(4) Resulting in significant or permanent disability/incapacity;

(5) Resulting in congenital anomaly or birth defect;

(6) Resulting in other important medical events: For example, important medical events that are not immediately life-threatening, or fatal, or require hospitalization but may jeopardize the patient, or require medical intervention to prevent the outcomes listed above, scientific medical judgment should be made immediately. These should also be considered SAEs.

The serious adverse event is further explained as follows:

(1) Death due to any AE occurring during the study. If a subject dies during the study with an autopsy performed, the autopsy results should be included in the subject's CRF.

(2) The occurrence of an AE will immediately place the subject at risk of death. AEs that may result in death after serious progression (e.g., drug-induced hepatitis without hepatic failure) are excluded.

(3) AEs that result in hospitalization or prolongation of hospitalization (prolongation of hospitalization is defined as a delay in the planned or expected date of discharge, usually at least 1 day overnight in the hospital). This does not include elective

surgeries or admission examinations decided before the trial, and the unchanged treatment course during the study.

(4) Any AE that results in the impairment, damage or disruption of the subject's function, physiological structure, or both, affecting physical activity or quality of life.

(5) It is suspected that exposure of either parent to the study drug will result in adverse outcomes in the offspring.

- **Significant AEs**

Significant adverse events refer to any adverse events other than serious adverse events that result in the use of targeted medical measures (e.g., discontinuation, dose reduction, and symptomatic treatment) and other significant abnormalities in hematology or other laboratory tests.

- **Adverse Drug Reactions**

Adverse drug reaction (ADR) refers to reactions harmful to the human body or unintended reactions that are considered to be related to the investigational product in a clinical trial. The investigational product and the AE are at least in one possibly reasonable causality, i.e., the correlation cannot be ruled out. .

13.2 Additional Considerations for PTEs and AEs

Generally, for the unfavorable results:

(1) Indication of a new diagnosis or an unexpected aggravation of a pre-existing condition. An intercurrent event due to pre-existing underlying conditions should not be considered a PTE or AE;

(2) Therapeutic intervention is required;

(3) Invasive diagnostic procedures are required;

(4) Discontinuation or change in the dose of study drug or concomitant medication is required;

(5) Considered unfavorable by the investigator for any reason;

(6) A PTE/AE caused by study procedures (e.g., bruises after blood collection) should be recorded as a PTE/AE. Signs and symptoms before and after the study procedure should be compared:

(7) Each event should be recorded as an individual diagnosis. Concomitant signs (including abnormal laboratory test values or ECG findings) or symptoms should not

be recorded as additional AEs. If the diagnosis is unknown, the signs or symptoms may be recorded as PTE or AE accordingly.

Laboratory Values and ECG Results:

(1) A change in laboratory value or ECG parameter will be considered a PTE or AE only if it is judged as a clinically significant abnormality (i.e., if certain actions or interventions need to be taken, or if the investigator concludes that the change is outside of the normal range of physiological fluctuation). Laboratory retests and/or ongoing monitoring of abnormal values are not considered interventions. In addition, repeated or additional non-invasive tests performed to validate, evaluate or monitor an abnormality are not considered as interventions.

(2) If an abnormal laboratory test value or ECG finding is a pathological finding of an overall diagnosis (e.g., an increase in creatinine in kidney failure), only this diagnosis should be reported as an AE or a PTE accordingly.

Pre-existing Conditions:

(1) Pre-existing conditions (present at the time of ICF signing) will be considered concomitant conditions, and should not be recorded as a PTEs or AEs. Baseline evaluations (e.g., laboratory tests, ECGs, and x-ray) should not be recorded as PTEs unless associated with study procedures. However, if a subject experiences aggravation of such concomitant condition or develops a complication, the aggravation or complication should be recorded as either a PTE (if the aggravation or complication occurs before the start of the study drug) or an AE (if the aggravation or complication occurs after the start of study drug), as appropriate. The Investigator should ensure that the event term recorded reflects a change in the condition (e.g., "aggravation of ...").

(2) If a subject has a pre-existing intercurrent condition (e.g., asthma and epilepsy), only an episode that becomes more frequent, more severe, or aggravates should be recorded as a PTE/AE, i.e., the Investigator should ensure that the recorded AE term can describe the change from baseline in the condition (e.g., "aggravation of ...").

(3) If a subject has a degenerative concomitant condition (e.g., cataract and rheumatoid arthritis), aggravation of the condition should be recorded as a PTE/AE only if it

aggravates more than expected. In addition, the investigator should ensure that the recorded AE term describes a change in the condition (e.g., "aggravation of ...").

Aggravation of PTE or AE:

(1) If a subject experiences an aggravation or complication of PTE after commencing study drug administration, the aggravation or complication should be recorded as an AE accordingly. The investigator should ensure that the recorded AE term describes a change in the condition (e.g., "aggravation of ...").

(2) If a subject experiences an aggravation or complication of an AE following any change in study drug, the aggravation or complication should be recorded as a new AE. The investigator should ensure that the recorded AE term describes a change in the condition (e.g., "aggravation of ...").

Change in AE/PTE Severity:

If a subject experiences a change in the severity of an AE/PTE, the event should be recorded once at its maximum severity.

Pre-planned Surgery or Procedure:

(1) A pre-planned procedure (surgery or therapy) scheduled before the signing of the ICF will not be considered a PTE or AE. However, if the pre-planned procedure is performed earlier (e.g., as an emergency) due to the aggravation of a pre-existing condition, then the aggravation of the condition should be recorded as a PTE or AE accordingly. Complications resulting from any planned surgery should be reported as AEs.

(2) Elective surgery or procedure: An elective surgery performed in the absence of a change in the subject's condition should not be reported as a PTE or AE, but should be recorded in the subject's source document. Complications due to elective surgeries should be reported as AEs.

(3) Insufficient clinical response (lack of efficacy): An insufficient clinical response, efficacy, or pharmacological effect should not be recorded as an AE. The Principal Investigator must distinguish between aggravation of a pre-existing condition and lack of therapeutic efficacy.

13.3 Definition of Causality

The causality between the investigational product and AEs will be judged according to the

following criteria.

(1) Unrelated: The AE is unrelated to the investigational product; the timing of the reaction does not align with the administration of the drug, the reaction is consistent with the known reaction types of non-investigational products, the patient's clinical condition or other treatments may also cause the reaction, the reaction resolves with the improvement of the disease or the cessation of other treatments, the reaction recurs with the repeated use of other treatments, and there is a close correlation to other risk factors.

(2) Unlikely related: The AE is more likely explained by other factors; the timing of the reaction does not align with the administration of the drug, the reaction does not match the known reaction types of the investigational product, and the patient's clinical condition or other treatment may also cause the reaction. The relationship with administration cannot be ruled out.

(3) Possibly related: The AE may be related to the investigational product, and other explanations are not persuasive; the time of occurrence of the reaction is in accordance with the temporal sequence of administration, the reaction conforms to the known reaction type of the investigational product, and the clinical status or other treatment methods of patients may also produce this reaction.

(4) Probably related: The AE is probably related to the investigational product, possible correlation in time is suggested, and the possibility of other explanations is low;

(5) Related: The AE has been listed as a possible adverse drug reaction and there is no reason to use other explanations. The timing of the reaction aligns with the administration of the drug, the reaction matches the known reaction types of the study drug, the reaction improves upon dose reduction or discontinuation, and reoccurs upon re-administration.

13.4 Judgment Criteria for Severity

Severity of AEs will be assessed according to CTCAE v5.0.

Grade 1: Mild; asymptomatic or mild symptoms; clinical or diagnostic observations only; intervention not indicated;

Grade 2: Moderate; minimal, local or non-invasive intervention; limiting age-appropriate instrumental activities of daily living (ADL) (instrumental ADL refers to cooking,

buying groceries or clothes, using the telephone, managing money, etc.);

Grade 3: Severe or with important medical significance, but not immediately life-threatening; hospitalization or prolongation of existing hospitalization; disabling; limiting self-care ADL (self-care ADL refers to bathing, dressing and undressing, feeding one-self, using the toilet, taking medications, but not bedridden);

Grade 4: Life-threatening consequences; urgent intervention indicated;

Grade 5: Death related to AE.

13.5 Actions with the Study Drug

(1) Discontinuation: Discontinue the study drug due to a specific AE;

(2) Dose unchanged: No need to discontinue the study drug due to a specific AE;

(3) Unknown: Used only when the actions to be taken cannot be determined;

(4) Not applicable: Discontinuation of the study drug due to reasons other than specific AEs, e.g., termination of the study, death of the subject, or the study drug being discontinued prior to the occurrence of the AE;

(5) Dose reduction: Reduce the dose due to a specific AE;

(6) Dose interruption: Temporarily interrupt (suspend) use of the study drug due to a specific AE, including voluntary interruption by the subject, and then resume the use.

13.6 Outcome of Adverse Events

(1) Recovered/resolved: The "end date of (serious) AE" should be indicated;

(2) Recovering/Resolving: The event is still not fully resolved, but the subject is in the recovery phase. Follow-up required;

(3) Not recovered/not resolved: The event is ongoing;

(4) Recovered/Resolved with Sequelae: Only if the subject has long-lasting or lifelong sequelae, such as blindness due to diabetes mellitus and hemiplegia after stroke. "End date of (serious) adverse event" should be noted;

(5) Death: "End date of (serious) AE" should be indicated for the death caused by an AE, and "end date of (serious) AE" is not required for death not caused by an AE. If the subject dies, the time of death should be recorded;

(6) Unknown: The Investigator is unable to know the AE, e.g., subject lost to follow-up.

If the outcome of an AE is rated as "recovering/resolving", or "not recovered/not resolved" or "unknown", the end date of the AE may not be recorded tentatively.

When the outcome of an AE is rated as "recovered/resolved" or "recovered/resolved with sequelae", the end date of the AE must be recorded.

All AEs must be followed up to determine the final outcome or until the stable status is reached or the subject is lost to follow-up.

After a subject completes the clinical study, the investigator should follow up on the outcome of AEs that may be related to the study drug or cannot be determined, or until the stable status is reached.

13.7 Collection and Reporting Procedures

13.7.1 Collection and Reporting Procedures of Pretreatment Events and All Adverse Events

Collection Period

PTEs will be collected from the time a subject signs the ICF to participate in the study to the time when the subject receives the first dose of the study drug or until screening failure. For a subject who discontinues the study before the reinfusion of the study drug, PTEs will be collected until the subject discontinues the study.

AE collection will begin when a subject receives the first dose of the study drug through the 28-day follow-up period after the subject's first dose. After the follow-up period, only spontaneous reports will be collected.

The end date of an AE/PTE is the date when the subject recovers, the event resolves with sequelae, or the subject dies due to the AE.

Reporting

The investigator will assess the occurrence of subjective AEs at each study visit. A neutral question such as "How have you been feeling since last visit?" may be asked. Subjects may report AEs occurring at any other time during the study. Subjects who experience PTEs must be monitored until the symptoms resolve or any clinically relevant changes in laboratory tests have returned to baseline values, or a satisfactory explanation for the changes can be made. Non-serious PTEs related or unrelated to study procedures do not require follow-up according to the objectives of the study protocol. All AEs experienced by subjects, regardless of their correlation to the investigational product, must be monitored until symptoms subside and any clinically relevant changes in laboratory test values return to baseline or are satisfactorily explained. All clinically significant laboratory abnormalities confirmed by repeat tests should be followed until they return to an acceptable level or are satisfactorily explained. All PTEs and

AEs will be recorded on the PTE/AE page of the CRF, regardless of the relationship to the therapeutic drug judged by the investigator. The following information will be documented for each event:

(1) Event terms;

(2) Start and end dates and times;

(3) Severity;

(4) Investigator's judgment of causality between the event and administration of investigational product (related or not related) (not required for PTE);

(5) Investigator's judgment of causality between the event and study procedures, including details of suspected procedures;

(6) Actions taken with the investigational product (not applicable for PTE);

(7) Event results;

(8) Severity.

The subject diary and questionnaire will not be used as the primary means for collecting AEs. However, if the information gathered by the investigator from these documents identifies a potential AE, the patient should be followed up appropriately for medical evaluation. If, as a result of this visit, an AE that has not been previously reported is identified, it should be reported in accordance with the normal reporting requirements.

13.7.2 Collection and Reporting of SAEs

For any SAE occurring in the trial, regardless of whether it is related to the investigational drug or not, the investigator should give timely rescue treatment. The investigator should fill in the *SAE Report Form* formulated for this trial as detailed as possible, sign and date. The investigator should report the SAE to the sponsor (or CRO designated by the sponsor) by email within 24 hours after becoming aware of it. The sponsor (or the CRO designated by the sponsor) will conduct an assessment, and then report the assessed suspected unexpected serious adverse reactions (SUSARs) to the principal investigator and project manager as well as their clinical trial institution and ethics committee in a rapid manner, and report to the national drug regulatory authority and health authorities. The investigator should sign and read the relevant safety information of the clinical study provided by the sponsor (or CRO designated by the sponsor) in a timely manner after receiving it, and report the SUSAR report provided by the sponsor (or CRO designated by the sponsor) to the ethics committee.

For SAEs, the description of symptoms, severity, occurrence time, treatment time, actions taken, follow-up time and mode, and outcome should be recorded in detail.

The investigator must report SAEs together with relevant assessment of causality. If the investigator's assessment of causality is lost or unavailable, the sponsor will judge the event until the investigator's assessment is finally available.

If the investigator cannot determine whether an AE is an SAE, the AE will be considered an SAE until its nature is proven otherwise. Such events will be notified in writing to local authorities and the relevant Investigators according to local requirements.

For all SAEs (including those that are still in the development stage after the end of the study and occur within 28 days after the end of the study), the investigator should follow up until there is a definite result to ensure that all problems are resolved. Detailed follow-up information should be provided (e.g., whether special treatment or hospitalization is required after the study).

The investigator will submit a follow-up report to the CRO until the resolution of the AE. In case of permanent impairment, follow-up is required until the event is considered stable.

Time limit requirements for expedited reporting:

(I) For SUSARs that are fatal or life-threatening, the sponsor (or the CRO designated by the sponsor) should report them as soon as possible, but not more than 7 days after initial awareness and submit a complete follow-up report within 8 days after the first report.

(II) For SUSARs that are not fatal or life-threatening, the sponsor (or the CRO designated by the sponsor) should report them as soon as possible, but not more than 15 days after initial awareness. After report submission, serious adverse reactions should continue to be followed up. Any new information or changes to the previous report should be promptly submitted in the form of a follow-up report, within 15 days of receiving the new information.

Reporting route:

Center for Drug Evaluation, NMPA	
Transmission Mode	Transmit by Gateway of the PV system
National Health Authority	
E-mail	saefax@163.com
Jiangsu Pacific Meinuoke Bio-pharmaceutical Co., Ltd.	
E-mail	lss@pmbp.cn
Fourth Military Medical University	

E-mail	znchen@fmmu.edu.cn
Pharmacovigilance Department, Beijing CTSmed Co., Ltd.	
E-mail	pv@ctsmed.com

13.8 Death

All deaths occurring during the study or within 28 days after withdrawal from the trial (last visit) must be notified to the sponsor within 24 hours, and if the criteria for expedited reporting are met, the regulatory authority should be notified within 7 days, and relevant follow-up information (cause of death, autopsy report, and hospital report) should be reported within the next 8 days (15 days in total).

In case of withdrawal due to death, the event should be reported as a progressive disease (PD) or AE, and the cause of death should be recorded in the CRF. If death is caused by PD combined with other causes, the investigator must determine the primary cause of death and appropriately classify the reason for withdrawal.

13.9 Hospitalization

AEs that required hospitalization are considered SAEs. In general, if admission procedures are handled and treatment is given, the AE will be considered as an SAE.

Hospitalization for elective surgery, routine clinical procedures, annual physical examination, admission observation, or protocol requirements rather than AEs will not be considered an AE but should be recorded on the Clinical Assessment Form and the CRF. If an unexpected event occurs in this process, it will be reported as a "serious" or "non-serious" AE according to conventional criteria.

Note: Hospitalization or prolongation of hospitalization for non-medical reasons/convenience or purely for clinical trial purposes does not meet the criteria for a medical event and therefore cannot be considered an SAE.

13.10 Pregnancy

Pregnancy will be determined by serum pregnancy test. Subjects found pregnant at screening will be excluded from the study; subjects who become pregnant during the treatment period must withdraw from the study.

All pregnancy events must be reported. The investigator should fill in the *Pregnancy Event Report Form* of the clinical trial and submit it to the sponsor within 30 days after being informed of the pregnancy event. The investigator must follow up and record processes and outcomes of all pregnancies, even if the subject has withdrawn from the study or the study has ended.

The outcomes of all pregnancies must be reported. Pregnancy events in the trial should be tracked to the end of pregnancy or one month after the fetus's birth if possible, and follow-up information should be reported in the form of the *Pregnancy Event Report Form*.

Pregnancy alone will not be considered an AE unless it is suspected that the study drug may have affected the efficacy of contraceptives; elective abortion without complications should not be handled as an AE, except for therapeutic abortion. Hospitalization for a normal birth of a healthy newborn should not be considered an SAE.

Any SAE that occurs during pregnancy (including those occurring after the last dose of the study drug) must be recorded on the SAE Report Form (e.g., maternal serious complications, spontaneous or therapeutic abortion, ectopic pregnancy, stillbirth, death neonatal, congenital anomaly or birth defect) and reported within 24 hours according to the procedures for reporting SAEs.

13.11 Overdose

Any therapeutic dose in excess of that specified in the protocol should be recorded on the CRF as an overdose and whether there is any AE related to the overdose should be recorded. Symptomatic supportive treatment should be given for AEs related to overdose.

14 Information of Study Drug

14.1 Name and Strength of Therapeutic Drug

Meplazumab for Injection is a new humanized IgG2 monoclonal antibody drug jointly developed by Jiangsu Pacific Meinuoke Bio-pharmaceutical Co., Ltd. and Fourth Military Medical University. Meplazumab for Injection is a recombinant humanized IgG2 antibody expressed in CHO cells (CHO DG44, a mutated Chinese hamster ovary cell). This molecule consists of 2 heavy chains containing 442 amino acids and 2 light chains containing 214 amino acids linked by disulfide bonds. Each light chain contains 2 intrachain disulfide bonds and each heavy chain contains 4 intrachain disulfide bonds; there are 4 interchain disulfide bonds between heavy chain and heavy chain, 2 interchain disulfide bonds between light chain and heavy chain, and a total of 18 disulfide bonds in the intact protein. Asparagine at position 292 in the Fc region of the heavy chain of this molecule is the only glycosylation site, and the glycosylation modifications are mainly G0F, G1Fa, G1Fb, and G2F glycoforms. The theoretical amino acid molecular weight of the molecule is 144,094 Da, and the theoretical isoelectric point is 7.45. The affinity constant (K_D) of Meplazumab for Injection to CD147 molecule is 1.7 ×

10^{-10} M. The molecular formula is $C_{6406}H_{9866}N_{1692}O_{2009}S_{50}$.

Biological Characteristics

1) Antibody Humanization

We used bioinformatics, recombinant DNA technology and other means to amplify the genes in variable regions of antibody light and heavy chains from hybridoma cell lines expressing anti-CD147 antibody 6H8, and replaced the FR sequences in the variable regions of antibody light and heavy chains with human-derived FR sequences by using bioinformatics method and recombinant technology. The human-derived FR sequences were inserted into expression vectors containing human IgG2 antibody constant region gene respectively, and then corresponding host cells were transfected for expression. The light and heavy chain CDR regions of the expressed antibody molecule were murine, while the FR region and constant region were human-derived. More than 2/3 of the entire molecule was human-derived. The variable region had the function of binding to antigens, while the constant region had the antibody effector function, immunogenicity, and species characteristics. The Fc segment of the chimeric antibody can prolong the half-life of the antibody in serum and theoretically reduce the immunogenicity of heterologous antibodies. This technology retains the complete murine monoclonal antibody variable region sequence, ensuring the affinity and specificity of the antibody. Although theoretically speaking, the constructed chimeric antibody still retains the heterology of murine variable region and may induce a HAMA response, the results of animal studies showed that humanized Meplazumab did not cause abnormal clinical symptoms in the tested animals, and had no significant effect on spontaneous activity, body weight, food consumption, body temperature, blood pressure, ECG, hematological indicators, serum biochemical indicators, urine indicators, CD3+/CD4+, CD3+/CD8+ lymphocytes in the blood, bone marrow cells, histopathology, injection site, etc.

2) High Affinity

The intensity of antibody-antigen interactions is mainly determined by the affinity between them. These interactions result from non-covalent bonding. Because some amino acid sites in the framework region of parental non-humanized monoclonal antibody may be involved in antigen binding or play an important role in maintaining the conformation of antigen binding region, the affinity of humanized antibody is reduced to a certain extent compared with that of its parental non-humanized monoclonal antibody. However, the affinity of Meplazumab to

CD147 extracellular segment molecules was determined by the SPR system. Kinetic-Langmuir model analysis showed that the equilibrium dissociation constant of Meplazumab did not change significantly compared with its parental non-humanized monoclonal antibody 6H8, with an affinity constant (K_D) of 1.7×10^{-10} M, indicating that Meplazumab had a similar affinity compared with its parental mouse monoclonal antibody 6H8 (affinity constant $K_D = 4.48 \times 10^{-10}$ M), ensuring the ability of this monoclonal antibody to bind to target molecules *in vivo* and *in vitro*.

14.2 Drug Product Formulation

Meplazumab will be supplied in lyophilized powder packed in vials, 10 mg/vial, including 1.60 mg of histidine, 3.08 mg of histidine hydrochloride, 50.0 mg of sucrose, 70.0 mg of mannitol and 1.0 mg of polysorbate 80.

The matching control drug is sterile normal saline (0.9%) for IV infusion.

Meplazumab will be provided by the sponsor. The control drug will be provided by the clinical study site.

14.3 Drug Packaging and Label

The production, packaging and labeling of the investigational product will be carried out in accordance with the Good Manufacturing Practice (GMP) and applicable laws and regulations. The investigational product will be appropriately packaged to protect it from contamination during transportation and storage.

The investigational product will be appropriately labeled in accordance with the GCP and GMP, and the label will include instructions that meet regulatory requirements. The drug label will at least include the following contents: clinical trial applicant, name of investigational product, usage, strength, batch number, protocol number or other unique code used in the clinical trial, drug number, shelf life, storage conditions, and the words "For Clinical Study Only".

14.4 Drug Management

14.4.1 Transfer and Storage

The study drug will be received, handled and stored by a specially-assigned person at the study site. Before administration, the drug will be kept under designated conditions, and can be accessed and checked only by the relevant responsible person of the study, which will be recorded in the CRF. The investigator is responsible for returning all unused therapeutic drugs to the sponsor.

14.4.2 Waste Drug Handling

Discarded study drug (including those with wrong labels, not used by subjects, and refused to be used by subjects) should be collected centrally and disposed of reasonably. All supplies used for reinfusion should be recovered and disposed of, including infusion tubes. The recovered antibody drugs and wastes after infusion should be kept by a specially-assigned person and registered for filing in time. They should be disposed of within one week in accordance with the medical waste disposal standards.

15 Ethics

15.1 Investigator’s Responsibility

The investigator is responsible for ensuring that the clinical study is conducted in accordance with the protocol, current ICH-GCP and relevant regulations of the National Medical Products Administration (NMPA) of China.

ICH-GCP is an internationally recognized standard of ethical and scientific quality for designing, conducting, recording, and reporting studies involving human subjects. Studies that adhere to this standard are considered consistent with the principles enunciated in the Declaration of Helsinki in terms of protecting the rights, safety, and interests of subjects and are trustworthy with respect to the quality of study data.

15.2 Independent Ethics Committee (IEC)/Institutional Review Board (IRB)

Before initiation of the study, the investigator must provide the IEC/IRB with the following documents:

Investigator's Brochure, study protocol, CRF, ICF, etc.

The trial cannot begin until the IEC/IRB has given their full approval for the study protocol, ICF, materials to assist in subject enrollment, compensation measures for subjects and the sponsor has received copies of the IEC/IRB approval documents. The approval document must indicate the approved study title (protocol number), study document name (including version number), and approval date.

At the end of the study, the investigator should inform the IEC/IRB that the trial has been completed.

15.3 Informed Consent

The investigator is responsible for explaining the objectives, methods, benefits and potential risks of the clinical trial to each subject. An ICF signed by the subject must be obtained before

any procedure related to the clinical trial. ICFs should be provided both orally and in writing. The ICF must be dated and signed by the subject, or by their parents, legal guardians or protectors for those subjects who are unable to sign the ICF by themselves for any reason. A copy of the signed ICF and information sheet should be kept by the subject.

The ICF must be approved by both the sponsor and the IEC/IRB. The ICF should comply with the Declaration of Helsinki, current GCP guidelines, applicable regulations, and sponsor's regulations.

By signing the ICF, the subject/patient must also agree to allow the sponsor, drug approval authority, auditors, and/or monitors to review the original data related to the clinical study, with the reviewer adhering to confidentiality statement.

15.4 Protection of Subject Data

Data from subjects that are essential to study the efficacy, safety, quality, and application of drugs are collected and processed in this study.

The confidentiality of these data will be fully ensured when they are collected and used, and relevant laws and regulations for protecting the privacy of subjects will be followed.

The investigator must take appropriate technical steps and management measures to protect the personal information of subjects from access and disclosure by other unauthorized personnel, accidental and illegal destruction, and accidental loss and alteration. The sponsor who has access to the subject's personal data will keep it confidential throughout the study.

16 Management Requirements

16.1 Protocol Amendment

All protocol amendments must be signed and dated by the sponsor before release. They should not be implemented without IEC/IRB approval, except when necessary to avoid an immediate hazard to the subjects, or when changes are made to only logistical or administrative aspects of the study (e.g. typographical errors, inconsistencies).

16.2 Data Management

16.2.1 Completion and Transfer of Original Data and Case Report Form (CRF)

The Data Department of the CRO will be responsible for the management of the study data to ensure the authenticity, integrity, privacy, and traceability of the clinical trial data.

The data in the eCRFs are all from the original medical records, which are filled in by the investigator or the investigator's designee, and the integrity and accuracy of the information

should be ensured. In case of any error that needs to be corrected, the modification should be carried out in accordance with the CRF filling instructions, and the name of the data modifier and the modification date will be automatically recorded in the CRF system.

The completed CRF should be submitted to the CRF system through the network in a timely manner. After SDV, review by DM, query, and so on of the data in the CRF system have been completed without any doubt, the investigator should confirm by electronic signature before the data locking.

16.2.2 Design and Establishment of Database

The database should be established by the Data Department of the CRO, and should meet the requirements of the *Guidelines for Biostatistics in Drug Clinical Trials*. Data traces such as system login, data entry, modification, and deletion in the database should be managed, and the database should be established using the CDISC standard if possible.

16.2.3 Data Entry

Data will be entered into the EDC database by authorized personnel. After data entry is completed, the EDC system will check through programmed study-specific logical checks to ensure the integrity and accuracy of information.

16.2.4 Query Handling

After data are entered and stored in the EDC system, system verification will be started to trigger queries, which need to be reviewed and answered by investigators. The data manager would assess the investigator's response and close the query if the response was acceptable. The data manager will also manually check the entered data to ensure the logic, consistency and accuracy of the data.

The subject data listings/reports will be generated by programming to support the manual data verification throughout the study. Manual queries can be added in the EDC system when the data requiring the clarification/verification/confirmation by the investigator arises. Before locking, the data manager should confirm that all queries have been resolved.

16.2.5 Data Quality Assurance

The clinical study will be subject to quality assurance audits by the sponsor or a person authorized by the sponsor. GCP audits may also be conducted by drug approval authorities. QA auditor is allowed to review all medical records, study-related documents and correspondence, and ICFs.

16.3 Monitoring

The sponsor will designate a monitor to conduct on-site monitoring. The monitor will be from the sponsor or a CRO authorized by the sponsor, and will operate according to the SOP of the CRO. The monitor should visit regularly from the beginning to the end of the study.

The monitor will have access to the relevant original data of this clinical study and will review the CRFs in accordance with the SOP to determine that the information is complete, accurate, and consistent with the original data.

The CRFs, copies of laboratory data and medical test results must be readily available for inspection by the Clinical Research Associate (CRA), auditors, and health authorities. The monitor should review all CRFs and ICFs.

16.4 Audits and Inspections

In accordance with GCP and the sponsor's audit plan, the sponsor's representative may choose to audit this trial to evaluate the conduct of the trial and compliance with the protocol, GCP and relevant regulatory requirements, and will review the facilities (such as drug storage sites and laboratories) and trial-related records at the site.

Government regulatory authorities may also inspect the facilities of the sponsor and/or the study site. The sponsor will inform the relevant Investigator immediately upon receipt of a notification to inspect the study site. Similarly, the investigator should inform the sponsor of any upcoming inspections.

The investigator must allow representatives of government regulatory authorities and personnel in charge of audit to perform the following activities:

- Inspect facilities of the study site;
- Meet all team members involved in the trial;
- Directly view test data and original documents;
- Consult all other documents related to the study.

Audits and inspections may be conducted at any time during or after the trial to ensure the validity and integrity of the trial data.

16.5 Verification of Original Records

The Investigator should properly handle all data obtained during the clinical study to guarantee the rights and privacy of patients. The investigator should allow the study monitor/auditor/inspector to review and inspect the required clinical study data, to verify the

accuracy of the original data and understand the study progress. If the original records cannot be verified, the investigator should assist the monitor/auditor/inspector in further verification of data quality control.

16.6 Study Completion/Termination

End of Study

After the last visit of the last subject in the study is completed, the study site should notify the sponsor, and the study can be considered completed. The sponsor will notify all study sites of the end time of the trial. Continuation of the trial after this time must be agreed upon by the sponsor and may be implemented without supplement to the protocol.

Study Termination

The sponsor reserves the right to terminate the trial at any time. Reasons for the sponsor to terminate the trial or stop the trial at a study center may include, but are not limited to:

(1) The investigator fails to comply with the study protocol or GCP guidelines;

(2) Safety considerations;

(3) There is sufficient evidence suggesting lack of efficacy;

(4) Inadequate subjects recruited by the investigator.

16.7 Confidentiality Agreement and Patient Privacy

The investigator should undertake to keep confidential to a third party any confidential information obtained from the sponsor, or provided or disclosed in connection with the present contractual relationship, and to use such information to the extent agreed herein.

As long as the sponsor has reasonable and justifiable reasons to require the investigator to maintain a confidentiality agreement, this agreement should be independent and valid during the contractual relationship between both parties.

The investigator should ensure the privacy of subjects. In all documents submitted to the sponsor, only the subject identification codes can be used to identify subjects in the study, and their names and hospitalization numbers should not be indicated. The investigator must maintain the name and address of the clinical trial subjects concerned and the enrollment forms corresponding to the clinical study subject codes. These enrollment forms should be kept in strict confidence by the investigator and cannot be submitted to the sponsor.

16.8 Use and Publication of Information

As the sponsors, Jiangsu Pacific Meinuoke Bio-pharmaceutical Co., Ltd. and Fourth Military Medical University have exclusive rights to this study. The authors and the manuscript will reflect collaboration between several investigators as well as collaboration between the sites and sponsor staff. Authors should be identified before the manuscript is written. Because there are many study sites participating in this study, individual articles are not allowed to be published before the final report of the multicenter study is completed, unless agreed by the sponsor. The sponsor has the final decision on the drafts and publications.

17 References

[1]. Diagnosis and Treatment Protocol for COVID-19 Patients (Tentative 9th Version). 2022.
[2]. Technical Guidelines for Clinical Trials of New Antiviral Drugs for COVID-19, 2022.
[3]. Rong Yan, Analysis on Clinical, Imaging and Serum Antibody Characteristics of Mild and Moderate COVID-19, 2021, Southern Medical University.
[4]. Fang Xu, et al. Clinical features and chest CT findings in moderate and severe COVID-19 patients: an analysis of 506 cases from Wuhan Huoshenshan Hospital. Chinese Journal of Clinical Infectious Diseases, 2020. 03(13): p161-166.
[5]. Vanarsdall, A.L., et al., CD147 Promotes Entry of Pentamer-Expressing Human Cytomegalovirus into Epithelial and Endothelial Cells. mBio, 2018. 9(3).
[6]. Pushkarsky, T., et al., CD147 facilitates HIV-1 infection by interacting with virus-associated cyclophilin A. Proc Natl Acad Sci U S A, 2001. 98(11): p. 6360-5.
[7]. Watanabe, A., et al., CD147/EMMPRIN acts as a functional entry receptor for measles virus on epithelial cells. J Virol, 2010. 84(9): p. 4183-93.
[8]. Wang, K., et al., CD147-spike protein is a novel route for SARS-CoV-2 infection to host cells. Signal Transduct Target Ther, 2020. 5(1): p. 283.
[9]. Chen, Z., et al., Function of HAb18G/CD147 in invasion of host cells by severe acute respiratory syndrome coronavirus. J Infect Dis, 2005. 191(5): p. 755-60.
[10]. Bian, H., et al., Safety and efficacy of meplazumab in healthy volunteers and COVID-19 patients: a randomized phase 1 and an exploratory phase 2 trial. Signal Transduct Target Ther, 2021. 6(1): p. 194.
[11]. Geng, J., et al., CD147 antibody specifically and effectively inhibits infection and cytokine storm of SARS-CoV-2 and its variants delta, alpha, beta, and gamma. Signal Transduct Target Ther, 2021. 6(1): p. 347.
[12]. Saphire, A.C., M.D. Bobardt and P.A. Gallay, Human immunodeficiency virus type 1 hijacks host cyclophilin A for its attachment to target cells. Immunol Res, 2000. 21(2-3): p. 211-7.
[13]. Zhinan, C., Use of hab18g/cd147 molecule as target for antiviral antagonists and thus obtained antiviral antagonist. 2007. p. 0.
[14]. Chen Zhinan et al., Application of anti-BASIGIN humanized antibody in the preparation of drugs for treatment of COVID-19, 2020. p18.
[15]. Chen Zhinan, Antagonists of CD147 receptor target for SARS coronavirus and AIDS virus (HIV-1), 2003. p12.
[16]. CHEN, Z.C., Use of hab18g/cd147 molecule as target for antiviral antagonists and thus

obtained antiviral antagonist. 2007. p. 0.
[17]. Chen Zhinan et al., Humanized anti-BASIGIN antibodies and the use thereof, 2019. p28.
[18]. CHEN, Z.N.B.C., et al., HAb18G/CD147, ITS ANTAGONIST AND APPLICATION. 2008. p. 0.
[19]. Yurchenko, V., S. Constant and M. Bukrinsky, Dealing with the family: CD147 interactions with cyclophilins. Immunology, 2006. 117(3): p. 301-9.
[20]. Schmidt, R., et al., Extracellular matrix metalloproteinase inducer (CD147) is a novel receptor on platelets, activates platelets, and augments nuclear factor kappaB-dependent inflammation in monocytes. Circ Res, 2008. 102(3): p. 302-9.
[21]. Geng, J.J., et al., Enhancement of CD147 on M1 macrophages induces differentiation of Th17 cells in the lung interstitial fibrosis. Biochim Biophys Acta, 2014. 1842(9): p. 1770-82.
[22]. Wu, J., et al., CD147 contributes to SARS-CoV-2-induced pulmonary fibrosis. Signal Transduct Target Ther, 2022. 7(1): p. 382.
[23]. Zhu, P., et al., CD147 overexpression on synoviocytes in rheumatoid arthritis enhances matrix metalloproteinase production and invasiveness of synoviocytes. Arthritis Res Ther, 2006. 8(2): p. R44.
[24]. Zhu, P., et al., Expression of CD147 on monocytes/macrophages in rheumatoid arthritis: its potential role in monocyte accumulation and matrix metalloproteinase production. Arthritis Res Ther, 2005. 7(5): p. R1023-33.
[25]. Wang, C.H., et al., Expression of CD147 (EMMPRIN) on neutrophils in rheumatoid arthritis enhances chemotaxis, matrix metalloproteinase production and invasiveness of synoviocytes. J Cell Mol Med, 2011. 15(4): p. 850-60.

-Clinical Trial Approval Letter No.:
Registration Classification of Therapeutic Biological Products: Class I

Clinical Study Protocol

A Multicenter, Double-blind, Randomized, Placebo-controlled, Add-on Phase III Clinical Study to Evaluate the Safety and Efficacy of Meplazumab for Injection in Patients with Severe SARS-CoV-2 Infection

Protocol No.:	MPZ-III-01-CN
Sponsor:	Jiangsu Pacific Meinuoke Bio-pharmaceutical Co., Ltd.<br>Fourth Military Medical University
Principal Director:	Zhi-Nan Chen
Contract Research Organizations:	Beijing Key Tech Statistical Technology Co., Ltd.<br>Beijing CTSmed Co., Ltd.
Version No.:	1.6
Version Date:	November 13, 2023

Confidentiality Statement

This document contains important confidential information, which is the property of the sponsor and shall not be disclosed unless required by current laws or regulations. All persons to whom this document is addressed should be informed of this confidentiality requirement. The requirement is also applicable to all the documents provided to you that are confidential in the future.

Compliance Statement

This study will be conducted in accordance with Good Clinical Practices (GCP)\ICH-GCP and the following regulations and guidelines applicable to clinical studies:

1) *Drug Administration Law of the People's Republic of China* (2019)

2) *Provisions for Drug Registration* (2020)

3) *Declaration of Helsinki* (2013)

4) *Guidelines for Laboratory Management of Biological Sample Analysis in Drug Clinical Trials* (Trial) (2011)

The principal investigator will ensure that the protocol remains unviolated and unchanged before obtaining permission from the sponsor and the Ethics Committee, except when necessary to prevent direct harm to subjects.

The study protocol, informed consent form (ICF), approval letter from the National Medical Products Administration (NMPA) of China, certificates of analysis (CoAs) and other relevant materials will be submitted to the Ethics Committee for review and approval. Before subject enrollment, an approval letter must be obtained from the Ethics Committee. Any amendments to the protocol must also be reviewed and approved by the Ethics Committee before implementation.

Table of Contents

Table of Contents 4
Synopsis 7
Study Schedule 13
1 Introduction 17
1.1 SARS-CoV-2 18
1.2 CD147 Target 18
1.3 Meplazumab for Injection 20
1.4 Mechanism of Meplazumab for Injection in the Treatment of COVID-19 21
2 Study Objectives, Endpoints, and Estimands 23
2.1 Study Objectives and Endpoints 23
2.2 Estimands 24
3 Study Design and Principle 27
3.1 Overall Design 27
3.2 Overview of Study Design 27
3.3 Principle of Study Design 27
4 Study Population 29
4.1 Inclusion Criteria 29
4.2 Exclusion Criteria 29
5 Treatment Assignment and Blinding 31
5.1 Study Blindness 31
5.2 Emergency Unblinding 32
5.3 Blind Review and Unblinding 32
6 Dosage and Method of Administration 33
6.1 Rationale for Dose Selection 33
6.2 Method of Administration 36
6.3 End of Study 36
6.4 Treatment Regimen 36
7 Medication Compliance 37
8 Concomitant Therapy 37
8.1 Permitted Drug Therapies for Subjects During the Study 37
8.2 Contraindicated Medications for Subjects During the Study 37
9 Study Evaluation 38
9.1 Efficacy Evaluation 38

9.2 PD Evaluation 39
9.3 Evaluation of Safety and Immunogenicity 39

10 Early Termination of Study 39

11 Subject Completion/Withdrawal 40

11.1 Completion 40
11.2 Early Termination/Withdrawal 40
11.3 Case Report Form (CRF) 41
11.4 Record Retention 41

12 Statistical Analysis 41

12.1 Analysis Data Set 41
12.2 Hypothesis Testing 42
12.3 General Principles for Statistical Analysis 42
12.4 Efficacy Analysis 42
12.5 Pharmacodynamic Analysis 45
12.6 Immunogenicity Analysis 46
12.7 Safety Analysis 46
12.8 Interim Analysis 48
12.9 Subgroup Analysis 48
12.10 Multiplicity Handling 48
12.11 Handling of Missing Data 48
12.12 Determination of Sample Size 48

13 Adverse Event Reporting 48

13.1 Relevant Definitions 49
13.2 Additional Considerations for PTEs and AEs 50
13.3 Definition of Causality 53
13.4 Judgment Criteria for Severity 54
13.5 Actions with the Study Drug 54
13.6 Outcome of Adverse Events 55
13.7 Collection and Reporting Procedures 55
13.8 Death 59
13.9 Hospitalization 59
13.10 Pregnancy 60
13.11 Overdose 60

14 Information of Study Drug 60

14.1 Name and Strength of Therapeutic Drug 60
14.2 Drug Product Formulation 62
14.3 Drug Packaging and Label 62
14.4 Drug Management 63

15 Ethics 63

15.1 Investigator's Responsibility 63

15.2 Independent Ethics Committee (IEC)/Institutional Review Board (IRB) 63
15.3 Informed Consent 64
15.4 Protection of Subject Data 64

16 Management Requirements 65

16.1 Protocol Amendment 65
16.2 Data Management 65
16.3 Monitoring 66
16.4 Audits and Inspections 66
16.5 Verification of Original Records 67
16.6 Study Completion/Termination 67
16.7 Confidentiality Agreement and Patient Privacy 68
16.8 Use and Publication of Information 68

17 References 68
18 Appendix 1 Ordinal Scale for Clinical Improvement [26-27] 70

Synopsis

**Study Title**	A Multicenter, Double-blind, Randomized, Placebo-controlled, Add-on Phase III Clinical Study to Evaluate the Safety and Efficacy of Meplazumab for Injection in Patients with Severe SARS-CoV-2 Infection
**Protocol No.**	MPZ-III-01-CN
**Phase of Development**	III
**Indication**	Patients diagnosed with severe SARS-CoV-2 infection as per the *Diagnosis and Treatment Protocol for COVID-19 Patients (Tentative 10th Version)* issued by the National Health Commission (NHC) of China.
**Study Objectives**	Primary Objective:<br>• To validate that Meplazumab is superior to placebo when added to standard of care in reducing all-cause mortality on D28 in patients with severe SARS-CoV-2 infection;<br>Secondary Objectives:<br>• To evaluate the efficacy of Meplazumab compared to placebo when added to the standard of care in increasing the discharge rate, reducing mortality, shortening the time to sustained clinical improvement, reducing the number of days with oxygen supplementation, mechanical ventilation, length of intensive care unit (ICU) stay and hospital stay, shortening the time to SARS-CoV-2 nucleic acid negative conversion, increasing the SARS-CoV-2 nucleic acid negative conversion rate, reducing SARS-CoV-2 viral load and improving the proportion of subjects with improvement in lung imaging in patients with severe SARS-CoV-2 infection;<br>• To evaluate the changes from baseline in levels of cytokines and chemokines associated with inflammatory and immune states as well as lymphocyte subsets in patients with severe SARS-CoV-2 infection after dosing with Meplazumab;<br>• To evaluate the safety of Meplazumab in patients with severe SARS-CoV-2 infection;<br>• To evaluate the immunogenicity of Meplazumab in patients with severe SARS-CoV-2 infection.
**Number of Subjects**	Approximately 352 subjects are planned to be enrolled
**Population**	Patients with severe SARS-CoV-2 infection
**Study Design**	This is a multicenter, double-blind, randomized, placebo-controlled, add-on phase III clinical study.<br>A 0.2 mg/kg test group and a placebo group will be set up for the study based on the standard of care protocol (SoC) in the *Diagnosis and Treatment*

	Protocol for COVID-19 Patients (Tentative 10th Version) [1], the *Technical Guidelines for Clinical Trials of New Antiviral Drugs for COVID-19 (Trial)* [2] and previous clinical study results. Treatment regimen: a single intravenous (IV) infusion of 0.2 mg/kg Meplazumab or placebo on the first day (D0) and D7 after the first dose during the treatment period, calculated based on subjects' body weight. It is expected that 352 subjects will be randomized (1:1) into the Meplazumab and placebo groups. Each subject will be evaluated for short-term efficacy within 28 days after the first dose to determine the treatment effect and safety of Meplazumab. Each subject will undergo a long-term follow-up within 56 days after the first dose to determine the safety of Meplazumab.<br>Subjects will be diagnosed and treated as per the *Diagnosis and Treatment Protocol for COVID-19 Patients (Tentative 10th Version)* issued by NHC and relevant clinical diagnosis and treatment guidelines. Any adverse events (AEs) that occur will be managed and treated based on their type and severity.<br>Subjects who discontinue treatment for any reason (including those who withdraw due to intolerable AEs, or those who are unable to continue treatment due to disease progression) will be followed up for short-term safety and efficacy within 28 days after the first dose.<br>The study is deemed complete when the last visit is completed for the last subject or the last data point is collected for statistical analysis, whichever occurs later. The last subject refers to the last case planned to be enrolled, or the last case actually enrolled if the COVID-19 epidemic has ended and no additional cases meet the enrollment criteria and do not meet the exclusion criteria.
**Inclusion/Exclusion Criteria**	**Inclusion Criteria (subjects must meet all of the following criteria):**<br>(1) Male or female patients with age ≥ 18 years old;<br>(2) Patients diagnosed with severe SARS-CoV-2 infection as per the *Diagnosis and Treatment Protocol for COVID-19 Patients (Tentative 10th Version)* issued by NHC;<br>(3) Subjects who agree to take effective non-drug contraceptive measures from signing the ICF to 3 months after the end of the study;<br>(4) Subjects who are capable of comprehending the study, willing to participate in the study and sign the ICF (for incapable subjects whose participation is deemed beneficial by the investigator, their legal guardians may sign the ICF on their behalf, with explanations recorded in relevant documents such as original medical records).

	**Exclusion Criteria (subjects who meet any of the following criteria will be excluded):**<br>(1) Subjects with any abnormal physical examination findings, abnormal laboratory tests and/or any medical history that, in the judgment of the investigator, may compromise their safety by participating in the study;<br>(2) Subjects who use anti-SARS-CoV-2 drugs within 3 half-lives prior to the first dose or during the study, such as Paxlovid (Nirmatrelvir Tablets/Ritonavir Tablets [co-packaged], $t_{1/2}$: approx. 6.05 h), Azvudine Tablets ($t_{1/2}$: approx. 9 h), Molnupiravir Capsules ($t_{1/2}$: approx. 3.3 h), Amubarvimab/Romlusevimab Injection ($t_{1/2}$: approx. 45 d/75 d), human COVID-19 immunoglobulins ($t_{1/2}$: approx. 3–4 w) or convalescent plasma ($t_{1/2}$: approx. 21 d), Deuremidevir Hydrobromide Tablets ($t_{1/2}$: approx. 4.80–6.95 h), Simnotrelvir Tablets/Ritonavir Tablets (co-packaged) ($t_{1/2}$: approx. 4.14 h), Baricitinib ($t_{1/2}$: approx. 12.5 h), Tocilizumab ($t_{1/2}$: approx. 21.5 d), Remdesivir ($t_{1/2}$ approx. 27 h), Leritrelvir ($t_{1/2}$: approx. 14.9 h), Favipiravir ($t_{1/2}$: approx. 4.5 h) and 2-deoxy-D-glucose ($t_{1/2}$: approx. 50 min);<br>(3) Patients diagnosed with critical SARS-CoV-2 infection as per the *Diagnosis and Treatment Protocol for COVID-19 Patients (Tentative 10th Version)* issued by NHC;<br>(4) Subjects with stage 4 severe chronic kidney disease or requiring dialysis (estimated glomerular filtration rate [eGFR] <30 mL/min/1.73 m^2), or an increase in serum creatinine of 44.2 μmol/L within 7 days, or oliguria (<400 mL/24 h) or anuria (<100 mL/24 h);<br>(5) Pregnant or lactating women;<br>(6) Subjects who will be transferred to a hospital that is not a participating site of the study within 72 hours;<br>(7) Patients known to be allergic to the study drug and its components;<br>(8) Subjects who have used live vaccines (live attenuated vaccines) within 2 weeks prior to randomization or during the study treatment and safety follow-up period;<br>(9) Subjects who are participating in another clinical study. They must undergo a washout period of 5 half-lives (depending on the investigational product used or 30 days from their participation in the previous study, whichever is longer);<br>(10) Subjects with total bilirubin (TBL) > 2 × upper limit of normal

	(ULN), or alanine aminotransferase (ALT) > 5 × ULN, or aspartate aminotransferase (AST) > 5 × ULN, or alkaline phosphatase > 5 × ULN;<br>(11) Subjects with platelet count < 50 × 10^9/L, or hemoglobin < 70 g/L;<br>(12) Patients with other factors deemed by investigators as not suitable for the trial.<br>*Note: The investigator should ensure that subjects meet all inclusion criteria and do not meet any exclusion criteria at screening. Subjects will be excluded from the study if their status (including laboratory results) changes after screening but before the first dose, causing them to meet any exclusion criteria.*
**Study Drug**	**Name of Study Drug:**<br>Meplazumab for Injection<br>**Method of Administration:**<br>Dissolve Meplazumab for Injection in 1 mL of sterile water for injection and add the resulting solution to 100 mL of 0.9% sodium chloride for IV drip over 30–60 min. (Placebo: 100 mL of 0.9% sodium chloride, IV drip.)<br>**Dosage Design:**<br>A placebo group and a test group will be set up for the clinical study, following the treatment regimen below: a single IV infusion of 0.2 mg/kg Meplazumab or placebo on the first day (D0) and D7 after the first dose during the treatment period, calculated based on subjects' body weight.
**Rationale for Dose Selection**	The dose for the clinical study is determined as 0.2 mg/kg according to the stage 1 clinical study report of an international multicenter phase II/III study (final report dated July 15, 2022) and the recommendations made by the Independent Data Monitoring Committee (IDMC) on December 19, 2021 based on interim analysis results (January 26, 2022)
**Efficacy Evaluation**	**Efficacy Evaluation**<br>**Primary Endpoint**<br>1) All-cause mortality on D28<br>**Secondary Endpoints**<br>2) Discharge rate on D28<br>3) Mortality on D28 and D56<br>4) Time to sustained clinical improvement (days; range: D0–D28), defined as patient discharge (discharge criteria: body temperature returning to normal for at least 3 days, significant improvement in respiratory symptoms without oxygen support, and two consecutive negative nucleic acid test results at least 24 hours apart) or a reduction of at least 2 points on the Ordinal Scale for Clinical

	Improvement (see Appendix 1)<br>5) Number of days with oxygen supplementation (days)<br>6) Number of days with mechanical ventilation (days)<br>7) Incidence and duration of re-ventilation 24 hours after withdrawal (days)<br>8) ICU and hospitalization time (days)<br>9) Time to SARS-CoV-2 nucleic acid negative conversion<br>10) Changes from baseline in SARS-CoV-2 nucleic acid negative conversion rate and SARS-CoV-2 viral load on D7, D14 and D28<br>11) Proportion of subjects with improvement in lung imaging (defined as any of the following changes from baseline in lung imaging during the study: 1) reduction in lesion diameter; 2) decrease in the number of lesions; 3) decrease in the number of lung lobes involved by lesions [3, 4])<br>**Pharmacodynamic Evaluation**<br>1) Changes from D0 (pre-dose) in the levels of cytokines and chemokines associated with inflammatory and immune states on D1, D4, D7 (before the second dose), D8, D10 and D28, including CRP, IL-2, IL-4, IL-6, INF-α, IL-8, IL-10, IL-12p70, IL-17, IL-1β, TNF-α and IFN-γ<br>2) Changes from D0 (pre-dose) in lymphocyte subsets CD3+, CD4+ and CD8+ on D1, D4, D7 (before the second dose), D8, D10 and D28
**Evaluation of Safety and Immunogenicity**	All subjects will be observed and recorded for any AEs/serious adverse events (SAEs) occurring during the study, including clinical symptoms, clinically relevant abnormal physical signs, abnormal laboratory tests and abnormal electrocardiogram (ECG) findings. The correlation between these events and the study drug will be judged. AEs will be followed up until they are recovered/returned to normal, returned to baseline levels, stable or deemed no longer necessary for follow-up based on clinical judgment. Besides, AEs will be evaluated as per CTCAEv5.0.<br>Infusion safety will be monitored before and after each dose by testing blood pressure (systolic and diastolic), pulse rate and blood oxygen saturation SpO_2 or oxygenation index, respiration rate and body temperature before each dose (within 30 min) and at 1 h ± 10 min, 6 h ± 1 h and 12 h ± 1 h after each dose. Hematology, blood chemistry, urinalysis, chest imaging, ECG and other indicators will be measured post-dose in the treatment period.<br>Blood samples will be collected pre-dose and post-dose (on D28 and D56) and tested for anti-drug antibodies (ADAs) to evaluate immunogenicity.

**Statistical Methods**	**General Principles for Statistical Analysis:**<br>The statistical analysis will be performed using SAS 9.4 or higher version.<br>The baseline result is defined as the last non-missing test result prior to the first dose of Meplazumab.<br>Statistical description will be performed using continuous variables, including number of cases (non-missing), mean, standard deviation (SD), median, maximum, and minimum. The number and percentage of patients in each category will be calculated as categorical variables. Unless otherwise specified, the number of missing subjects will not be included in the calculation of percentage.<br>**Analysis of Primary Estimand:**<br>For the Meplazumab and placebo groups, the all-cause mortality on D28 post-dose will be calculated. The Clopper-Pearson method will be used to calculate its two-sided 95% confidence interval (CI). CMH Chi-square test will be used for the statistical test of inter-group difference, with age (< 65 years or ≥ 65 years) as the stratification factor. Meanwhile, the difference in the all-cause mortality on D28 post-dose (test group-control group) and its two-sided 95% CIs will be calculated using the CMH method, with age (< 65 years or ≥65 years) as the stratification factor. Subgroup analysis can be performed by age (<65 years or ≥65 years), gender (M or F), concomitant use of antiviral drugs (Yes or No), SARS-CoV-2 vaccination status (Yes or No), underlying disease (Yes or No), BMI ≥30 (kg/m^2) (Yes or No) and smoking (Yes or No).<br>**Safety Analysis:**<br>Pooled analyses will be performed for treatment-emergent AEs (TEAEs). TEAEs are defined as AEs occurring after the first dose. TEAEs will be summarized by SOC and PT to calculate the number of subjects and incidence under each category.<br>Safety evaluation parameters such as laboratory tests, vital signs and ECG findings will be summarized with descriptive statistics. Changes from baseline in these parameters will be summarized and classified as normal or abnormal (with or without clinical significance). The number and proportion of subjects under each category will be calculated.

Study Schedule

Assessments/Procedures	*Screening*	*Treatment*					End of Treatment
**Visit Time**	**D-3 to D-1**	**First Dose (D0)**	**QD Until Discharge**	**D7 Post-Dose**	**D14[a] ± 3 Days Post-Dose**	**D28[a] ± 3 Days Post-Dose**	**D56[a] ± 7 Days Post-Dose**
**Enrollment**							
Informed consent	X						
Demographic and medical history [b]	X						
Date of Symptom Onset	X						
Inclusion/Exclusion Criteria	X						
**Study Intervention**							
Randomization		X					
Dosing with Meplazumab or placebo; subjects will be weighed prior to each dose on each dosing day to determine accurate dosage		X		X[c]			

Assessments/Procedures	*Screening*	*Treatment*					End of Treatment
**Visit Time**	**D-3 to D-1**	**First Dose (D0)**	**QD Until Discharge**	**D7 Post-Dose**	**D14[a] ± 3 Days Post-Dose**	**D28[a] ± 3 Days Post-Dose**	**D56[a] ± 7 Days Post-Dose**
SoC until discharge at the discretion of treating physician		X					
**Study Procedures**							
Admission and discharge dates [d]	X	X	X				
COVID-19 diagnosis date	X						
Physical examination [e]	X						
Lung imaging (CT scan)[f]	X	X (pre-dose)		X (before the second dose)	X		
Vital signs, including SpO_2/oxygenation index [g]		X[i]	X	X	X	X	
Ordinal Scale for Clinical Improvement	X	X[i]	X	X	X	X	X
- Concurrent medications	X	X[i]	X	X	X	X	X
AE evaluation	X	X	X	X	X	X	X
Drug infusion safety monitoring [h]		X		X			
Survival status (dead or not)		X	X			X	X
Number of days with low-flow oxygen supplementation Nasal cannulas and simple masks	X	X	X			X	
Number of days with high-flow oxygen supplementation Venturi masks and high-flow nasal cannulas	X	X	X			X	
Number of days with invasive mechanical ventilation		X	X			X	
Number of days without invasive mechanical ventilation		X	X			X	
**Safety Laboratory**							

Assessments/Procedures	*Screening*	*Treatment*					End of Treatment
**Visit Time**	**D-3 to D-1**	**First Dose (D0)**	**QD Until Discharge**	**D7 Post-Dose**	**D14[a] ± 3 Days Post-Dose**	**D28[a] ± 3 Days Post-Dose**	**D56[a] ± 7 Days Post-Dose**
Urinalysis [j]	X[k]		X (within 3 days before discharge)				
Safety hematology, blood chemistry and coagulation [j]	X[k]	X[i,]	If hospitalized, on D2, D4, D7, D10 (± 1 d) and the day of discharge (± 1 d)			X	
12-lead ECG		X[i]				X	
Blood pregnancy test for women of childbearing potential	X						X
Hepatitis and serological tests (HIV, HBV, HCV)	X[k]						
**Study Laboratory**							
Blood collection for pharmacodynamic (PD) assessment [l]		X	X[l]	X[l]		X	
ADA assessment		X[i]				X	X
Nasopharyngeal swab collection for nucleic acid testing [m]		X[i]	X[m]	X[m]	X[m]	X[m]	X[m]

Abbreviations: CT = computed tomography; ECG = electrocardiogram; HBV = hepatitis B virus; HCV = hepatitis C virus; HIV = human immunodeficiency virus.

a. Subjects are required to undergo scheduled visits on site. However, if a subject is unable to return to the study site due to epidemic quarantine or other factors, post-discharge remote or home visits are acceptable. If a patient has been discharged before D14, a telephone follow-up on D14 is acceptable. For those who do not achieve negative conversion, nucleic acid test results obtained from another hospital are acceptable.

b. Medical history records: COVID-19 history and other past medical histories, such as chronic lung disease (including asthma), chronic kidney disease, diabetes mellitus, heart disease, hypertension, autoimmune diseases, tumors and organ transplantation.

c. If a subject has been discharged before dosing on D7, no more dosing is required on D7.

d. Dates to be recorded: The dates of admission to and discharge from ICU, the dates of re-admission to and re-discharge from ICU (if available), and the dates of admission to and discharge from hospital.

e. Physical examination: Appearance, eyes, ears, nose, throat, thyroid/neck, lymph nodes, heart, lungs, gastrointestinal tract, abdomen, skeletal muscle/extremities, skin, and neurology/psychiatry.

f. Lung imaging (CT scan) will be performed before the first dose (results obtained within 72 h before the first dose are acceptable), on D7 (± 1d) and D14 (± 1d). If a patient is discharged before D14 or meets COVID-19 discharge criteria, a CT scan is not required in subsequent visits; however, at least one CT scan should be performed at discharge. Additional CT scans may be performed as needed depending on the changes in the subject's conditions.

g. Body temperature, pulse rate, respiratory rate, blood pressure (systolic and diastolic) and blood oxygen saturation (SpO_2) or oxygenation index.

h. Drug infusion safety monitoring: Vital signs, including blood pressure (systolic and diastolic), pulse rate, blood oxygen saturation SpO_2 or oxygenation index, respiration rate and body temperature, will be measured before each dose (within 30 min) and 1 h ± 10 min, 6 h ± 1 h and 12 h ± 1 h after each dose during the treatment period.

i. Baseline assessments should be performed prior to dosing of study drug.

j. Safety hematology, blood chemistry, coagulation and urinalysis:

- Urinalysis: White blood cells (WBC), red blood cells (RBC), pH and protein;
- Hematology: WBC, RBC, hemoglobin (HGB), platelet (PLT) count, absolute lymphocytes, absolute neutrophils, absolute monocytes, absolute eosinophils, absolute basophils, hematocrit (HCT), mean corpuscular volume (MCV), mean corpuscular hemoglobin (MCH), and mean corpuscular hemoglobin concentration (MCHC);
- Blood chemistry: Liver function (total protein, albumin, total bilirubin [TBL], direct bilirubin, indirect bilirubin, alanine aminotransferase [ALT], aspartate aminotransferase [AST], alkaline phosphatase and gamma-glutamyl transferase); renal function (urea, creatinine, uric acid, cystatin C); lactate dehydrogenase, cardiac troponin, D-dimer and ferritin; blood lipids (triglyceride, high-density lipoprotein, low-density lipoprotein and total cholesterol); postprandial glucose; electrolytes (sodium, potassium, chloride and calcium);
- Coagulation: Prothrombin time, activated partial thromboplastin time, fibrinogen and thrombin time;

k. Test results obtained within 72 h prior to the first dose are acceptable.

l. PD endpoints: CRP, IL-2, IL-4, IL-6, INF-α, IL-8, IL-10, IL-12p70, IL-17, IL-1β, TNF-α and IFN-γ, as well as lymphocyte subsets CD3+, CD4+ and CD8+. Blood samples will be collected at the following time points, when available: pre-dose and on D1, D4, D7 (before the second dose), D8, D10 and D28 (optional). If the second dose is not administered, no sample will be collected on D7, D8 and D10.

m. If a subject is tested negative for nucleic acid before the first dose, no more nucleic acid testing is required during the study. For those who are still positive for nucleic acid before the first dose, nucleic acid testing will be performed during the study. If a negative nucleic acid test result is obtained, a re-test is required at least 24 hours later. If a subject is tested negative for nucleic acid before discharge (two consecutive negative results, at least 24 hours apart), no more nucleic acid testing is required at subsequent visits; if a subject does not achieve negative conversion before discharge, nucleic acid testing must continue at subsequent visits.

1 Introduction

Coronaviruses are a group of enveloped single-stranded positive-sense RNA viruses that can infect both humans and a variety of vertebrates. They are important pathogens responsible for common colds and upper respiratory tract infections in humans. In recent years, several highly contagious and pathogenic coronaviruses have emerged successively, including SARS-CoV in 2002, MERS-CoV in 2012 and SARS-CoV-2 at the end of 2019. The emergence of these novel coronaviruses poses a serious threat to public health, leading to a progressive decrease in respiratory function and even death in some subjects. The coronavirus disease 2019 (COVID-19) has become a major public health event worldwide. As the global pandemic is still spreading and has the potential to persist for a long time, especially four variants (N501Y, N501Y-D614G, B.1.1.7 (UK), B.1.351 (South Africa)) have recently been prevalent worldwide. To date, the B.1.1.7 variant has spread globally and the E484K mutation has also emerged in the United Kingdom and the United States. It is estimated that the sensitivity of this E484K mutant virus to immune sera from individuals vaccinated with Pfizer Biotechnology's genetic vaccine is reduced by about 6-fold, and its susceptibility to convalescent sera is reduced by about 11-fold; in addition, the B.1.1.7 strain has become widespread in 33 states of the United States, spreading at an unexpected speed. The B.1.351 and P.1 variants, also known as 501Y.V2 and 501Y.V3, are present in South Africa and Brazil, respectively, with three important mutation sites N501Y, E484K and K417N/T resulting in significantly reduced susceptibility to vaccine-induced and convalescent sera. Concerningly, there is evidence of convergent evolution with mutations such as N501Y and E484K that occur not only in these mutants but also in other variants of concern such as P3 in the Philippines, B.1.526 in the United States, and B.1.525 in the United Kingdom and West Africa. India has seen its second wave of COVID-19 since March 2021, setting a world record for more than 400,000 new confirmed cases in a single day. Gene sequencing was performed for 13,000 samples in India, of which 3,532 had "mutations of concern". The risk of transmission and spreading of COVID-19 in China will also persist. At present, the treatment of coronavirus disease 2019 (COVID-19) is mainly supportive symptomatic treatment and antiviral treatment. Specific drugs are still not available. There is an urgent need to develop new treatment methods and drugs for coronavirus infection. In this study, on the basis of previous work, a clinical treatment

study on COVID-19 will be carried out. CD147 molecule with independent intellectual property rights will be used as the target, and humanized Meplazumab targeting CD147 molecule will be used to block the binding between SARS-CoV-2 and target cells, so as to obtain innovative treatment means and new strategies for pneumonia caused by infection with the virus.

1.1 SARS-CoV-2

Coronaviruses are a class of single-stranded positive-sense RNA viruses that can infect humans and many vertebrates, and are important pathogens causing the common cold. Up to now, there are seven known human coronaviruses (including the newly discovered SARS-CoV-2), three of which have high transmission rates and high case fatality rates, namely the Severe Acute Respiratory Syndrome Coronavirus (SARS-CoV-1) that caused the outbreak in Guangdong Province, China in 2002, the Middle East Respiratory Syndrome Coronavirus (MERS-CoV) that caused the outbreak in Saudi Arabia in 2012, and the novel coronavirus (SARS-CoV-2) that caused the outbreak in Wuhan, China at the end of 2019. They are all β coronaviruses. Symptoms of MERS typically include pyrexia, cough and tachypnoea, or even pneumonia, with a case fatality rate of about 34.4%. Symptoms of SARS typically include pyrexia, chills and body pain, or even pneumonia, with a case fatality rate of about 9.6%. Coronaviruses contain at least four structural proteins: S (spike), E (envelope), M (membrane), and N (nucleocapsid) proteins. S protein promotes host attachment and virus fusion with cell membrane during viral infection, while N protein is mainly involved in maintaining the stability of the genome and the formation of nucleocapsid.

1.2 CD147 Target

CD147 was first discovered by Biswas in 1982 and named as tumor cell collagenase stimulatory factor. With the progress of studies, it was successively named extracellular matrix metalloproteinase inducer (EMMPRIN), M6, HAb18G (obtained by the study team in liver cancer tissue screening), etc. The 6th International Workshop and Conference on Human Leukocyte Differentiation Antigens gave different names from various laboratories a new number, CD147. CD147 is a member of the immunoglobulin superfamily, a type I transmembrane glycoprotein highly expressed in cancer cells and in various tumor tissues, including liver cancer, glioblastoma, lung cancer, breast cancer, lymphoma, squamous cell carcinoma of the oral cavity, melanoma, bladder cancer and kidney cancer. It is a

broad-spectrum tumor-associated antigen [5]. Also, CD147 expressed by host cells is an important receptor or coreceptor for various pathogens to invade target cells. It has been reported in the literature that CD147 can mediate infection of hosts by human cytomegalovirus [6], HIV [6], measles virus [7] etc. Our previous studies have shown that the S protein of SARS-CoV-2 can interact with CD147. The S protein recognizes the CD147 receptor on host cells and the virus invades host cells by endocytosis, suggesting that CD147-SP is a new pathway for COVID-19 to invade host cells [8]. In addition, CD147-CyPA is also a receptor-ligand interaction molecule that binds to CyPA through extracellular Pro180 and Gly181 sites, mediating chemotaxis of inflammatory cells and cytokine storm. We also identified the CD147-CyPA interaction, which played an important role in coronavirus infection of host cells [9].

Based on the CD147 overexpression, knockdown/knockout cell models, and the humanized CD147 (hCD147) transgenic mouse model with SARS-CoV-2 infection, we demonstrated *in vitro* and *in vivo* that CD147 mediated SARS-CoV-2 and its variants to infect host cells. CD147 is one of the routes of SARS-CoV-2 infection [8].

After stably interfering with CD147 expression in Vero E6 and BEAS-2B cells, the ability of SARS-CoV-2 to infect them was significantly reduced by 2.08 and 5.17 folds, respectively; however, overexpression of CD147 in BEAS-2B and BHK-21 cells enhanced the viral infection capacity by 3.99 and 3.96 folds respectively.

Compared with wild-type Vero E6 cells, the content of SARS-CoV-2 wild strain (Wuhan strain) and its variants B.1.1.7 strain (501Y.V1) and B.1.351 strain (501Y.V2) in CD147 knockout Vero E6 cells was significantly down-regulated by 3.79, 2.07 and 3.99 folds, respectively.

The drug R&D team found for the first time that the host cell receptor CD147 interacts with SARS-CoV-2 S protein and is a new receptor for SARS-CoV-2 to invade host cells. SARS-CoV-2 invades host cells through CD147-dependent receptor-mediated endocytosis, which is closely related to the regulation of Arf6. CD147 provides a key target for the development of specific drugs for COVID-19.

Based on the previous work, we will carry out a clinical study on the treatment of pneumonia caused by infection with SARS-CoV-2 and its variants. The receptor CD147 molecule found by our team for the first time will be used as the target, and humanized Meplazumab targeting CD147 molecule will be used to block SARS-CoV-2 epidemic strains and Alpha, Beta, Gamma,

Delta and Omicron strains from infecting the cell, without being affected by viral mutations. The treatment is expected to have strong inhibition on infection invasion and viral replication for all these strains. The clinical trials of this product were conducted in healthy volunteers and patients with severe and moderate COVID-19. Five clinical trials, including Phase I clinical trials [10], exploratory Phase II clinical trial [10], international multicenter Phase II/III clinical trial, and China multicenter Phase II/III clinical trial, have confirmed that the drug has good safety, can effectively reduce the mortality rate of severe/critical patients and increase the discharge of severe/critical patients, improve the disease improvement rate (reduce the clinical severity by two levels), inhibit viral replication, shorten the time to virus negative conversion, improve the early virus negative conversion rate and early discharge rate of patients with mild and moderate disease. Also, studies have confirmed that this antibody can also block the interaction between CD147 and the pro-inflammatory factor CyPA, and reduce the occurrence of cytokine storm in the body [11], with clear pharmacological effects. On December 8, 2022, "Meplazumab for Injection", an anti-COVID-19 specific receptor blocking antibody drug targeting CD147, was approved for production, becoming the first receptor blocking drug approved for the treatment of severe and critical COVID-19 in the world.

1.3 Meplazumab for Injection

Meplazumab for Injection is a new humanized monoclonal antibody drug jointly developed by the National Translational Science Center for Molecular Medicine, Fourth Military Medical University and Jiangsu Pacific Meinuoke Bio-pharmaceutical Co., Ltd. Meplazumab is a recombinant humanized IgG2 antibody expressed in CHO cells. We replaced the FR sequences in the variable regions of light and heavy chains of the antibody with human-derived FR sequences using bioinformatics method and recombinant technology, and more than 2/3 of the entire molecule was human-derived. The variable region had the function of binding to antigens, while the constant region had the antibody effector function, immunogenicity, and species characteristics. The Fc segment of the chimeric antibody could prolong the half-life of the antibody in serum, theoretically reducing the immunogenicity of heterologous antibodies. The equilibrium dissociation constant of the humanized antibody did not change significantly compared with its parental non-humanized monoclonal antibody, and the affinity constant (K_D) was 1.7×10^{-10} M, indicating that Meplazumab had similar affinity to its parental non-humanized monoclonal antibody 6H8 (affinity constant $K_D = 4.48 \times 10^{-10}$ M), ensuring the

ability of this monoclonal antibody to bind to target molecules *in vitro* and *in vivo*.

1.4 Mechanism of Meplazumab for Injection in the Treatment of COVID-19

Coronaviruses have at least four structural proteins: S, E, M and N proteins. S protein is the main component of nucleocapsid. In recent years, it has been found that CyPA can bind to the N protein of coronavirus and play an important role in viral replication, assembly, release and infection. In particular, CyPA is not only directly involved in the budding and release of progeny viruses, but also presents on the surface of the viral membrane after viral release, and participates in the recognition and binding process between coronaviruses and target cells[9, 12].

Our recent studies have shown that the S protein of SARS-CoV-2 can interact with CD147. The S protein recognizes the CD147 receptor on host cells and the virus invades host cells by membrane fusion, suggesting that CD147-SP is a new pathway for COVID-19 to invade host cells [8]. In addition, CD147-CyPA is also a receptor-ligand interaction molecule that binds to CyPA through extracellular Pro180 and Gly181 sites, mediating chemotaxis of inflammatory cells and cytokine storm. In the previous work, we also identified the CD147-CyPA interaction, which played an important role in coronavirus infection of host cells [11]. Several domestic and international patents have been filed for relevant contents [13-18].

Studies showed [19, 20] that the expression of CD147 in bronchoalveolar lavage fluid or lung tissue was elevated in patients with pulmonary interstitial fibrosis; in patients with UIP-IPF, the expression of CD147 in macrophages and alveolar epithelial cells in fibrotic foci areas was elevated, suggesting that CD147 played an important role in the occurrence and development of pulmonary fibrosis. Our previous study showed [21] that anti-CD147 antibody significantly reduced the collagen score and improved pulmonary interstitial fibrosis in a mouse model of bleomycin-induced pulmonary interstitial fibrosis. The team's latest study found that CD147 is a key regulator of fibroblast activation during pulmonary fibrosis, in addition to mediating SARS-CoV-2 invasion and inducing cytokine storm. Meplazumab inhibited the accumulation of activated fibroblasts and the production of extracellular matrix proteins, reducing the progression of pulmonary fibrosis caused by SARS-CoV-2 [22].

CD147 is expressed in T cells, mononuclear macrophages, dendritic cells, B cells, and other cells associated with various inflammatory diseases, and the degree of progression of inflammatory diseases is closely related to the expression level of CD147. Our previous studies

showed that, in patients with rheumatoid arthritis, the expression of CD147 on synovial fibroblasts, neutrophils and mononuclear macrophages was significantly increased. When chemotaxis of these cells reached the inflammatory site, CD147 promoted the expression of a large number of matrix metalloproteinases in the diseased site, which ultimately increased the rate of cartilage destruction in rheumatoid arthritis [23-25]. Treatment of rheumatoid arthritis mouse model with CD147 monoclonal antibody significantly inhibited the secretion of various inflammatory-related factors and MMPs by synovial fibroblasts, thus attenuating their destruction to implanted synovial cartilage.

In summary, the main pharmacological mechanism of this product is as follows:

CD147 is a universal receptor for SARS-CoV-2 to infect humans, mediating the infection with SARS-CoV-2 epidemic strains and variants Alpha, Beta, Gamma, Delta, and Omicron (BA.1 and BA.2) in humans. Meplazumab can directly bind to CD147 and block the interaction between CD147 and SARS-CoV-2 S protein, thus inhibiting SARS-CoV-2 infection. In the model of human CD147 transgenic mice infected with SARS-CoV-2 epidemic strains and variants Alpha, Beta and Omicron BA.1, Meplazumab reduced viral load and inhibited the pathological characteristics of exudative alveolitis; in international multicenter clinical trials for severe COVID-19, Meplazumab had antiviral effects of reducing viral load and increasing negative rate and negative conversion rate.

CD147 plays a core regulatory role in the cytokine storm induced by SARS-CoV-2 and mediates the cytokine storm (inflammatory storm) caused by CyPA upregulation. Meplazumab blocks the interaction between CD147 and CyPA by binding to CD147, and has an anti-inflammatory effect of inhibiting various cytokine storms caused by SARS-CoV-2 infection.

2 Study Objectives, Endpoints, and Estimands

2.1 Study Objectives and Endpoints

Table 1 Study Objectives and Endpoints

**Primary Objective**	**Primary Endpoint**
To validate that Meplazumab is superior to placebo when added to standard of care in reducing all-cause mortality on D28 in patients with severe SARS-CoV-2 infection.	• All-cause mortality on D28
**Secondary Objective - Efficacy**	**Secondary Efficacy Endpoints**
To evaluate the efficacy of Meplazumab compared to placebo when added to the standard of care in increasing the discharge rate, reducing mortality, shortening the time to sustained clinical improvement, reducing the number of days with oxygen supplementation, mechanical ventilation, length of intensive care unit (ICU) stay and hospital stay, shortening the time to SARS-CoV-2 nucleic acid negative conversion, increasing the SARS-CoV-2 nucleic acid negative conversion rate, reducing SARS-CoV-2 viral load and improving the proportion of subjects with improvement in lung imaging in patients with severe SARS-CoV-2 infection	• Discharge rate on D28<br>• Mortality on D14 and D56<br>• Time to sustained clinical improvement (days, range: D0–D28)<br>• Number of days with oxygen supplementation (days)<br>• Number of days with mechanical ventilation (days)<br>• Incidence and duration of re-ventilation 24 hours after withdrawal (days)<br>• ICU and hospitalization time (days)<br>• Time to SARS-CoV-2 nucleic acid negative conversion<br>• Changes from baseline in SARS-CoV-2 nucleic acid negative conversion rate and SARS-CoV-2 viral load on D7, D14 and D28<br>• Proportion of subjects with improvement in lung imaging
**Secondary Objective - Pharmacodynamics**	**Pharmacodynamic Evaluation**
To evaluate the changes from baseline in levels of cytokines and chemokines associated with inflammatory and immune states as well as lymphocyte subsets in patients with severe SARS-CoV-2 infection after dosing with Meplazumab	• Changes from D0 (pre-dose) in the levels of cytokines and chemokines associated with inflammatory and immune states on D1, D4, D7 (before the second dose), D8, D10 and D28, including CRP, IL-2, IL-4, IL-6, INF-α, IL-8, IL-10, IL-12p70, IL-17, IL-1β, TNF-α and IFN-γ<br>• Changes from D0 (pre-dose) in lymphocyte subsets CD3+, CD4+ and CD8+ on D1, D4, D7 (before the second dose), D8, D10 and D28
**Secondary Objectives - Safety**	**Safety Endpoints**
To evaluate the safety of Meplazumab in patients with severe SARS-CoV-2 infection	• Incidence of adverse events (AEs) and serious adverse events (SAEs);<br>• Vital signs (blood pressure, pulse, blood oxygen saturation SpO_2 or oxygenation index, respiration, and body temperature);<br>• Laboratory tests (hematology, urinalysis, blood chemistry and coagulation);<br>• 12-lead ECG;<br>• Chest imaging.
**Secondary Objectives - Immunogenicity**	**Immunogenicity Evaluation**
To evaluate the immunogenicity of Meplazumab in patients with severe SARS-CoV-2 infection	• Anti-drug antibody

2.2 Estimands

2.2.1 Primary Estimand

The primary estimand of this trial is the difference between the test group and control group in the all-cause mortality on D28 after treatment in patients with severe SARS-CoV-2 infection. Specifically:

Population: The target population of this trial is patients with severe SARS-CoV-2 infection. Refer to Section 4 "Study Population" for specific inclusion requirements.

Variable: Whether the subject dies on D28;

Treatment: Subjects in the test group will receive Meplazumab and standard of care (SoC), while subjects in the control group will receive placebo and SoC. Refer to Section 6 "Dosage and Method of Administration" for the specific treatment regimen.

Intercurrent Events and Handling Strategies: The main intercurrent events and handling strategies in the study are defined as follows:

Table 2 Intercurrent Events of Primary Estimand and Handling Strategies

Intercurrent Events	Handling Strategies	Remarks
Early discontinuation of treatment due to poor efficacy	**Therapeutic Strategy** Continue to collect and use data despite the intercurrent event	Reflect actual clinical condition
Early discontinuation of treatment due to adverse events	**Therapeutic Strategy** Continue to collect and use data despite the intercurrent event	Reflect actual clinical condition
Concomitant use of prohibited medications/treatments affecting efficacy evaluation	**Therapeutic Strategy** Continue to collect and use data despite the intercurrent event	Reflect actual clinical condition
Disease aggravation (progression to critical illness)	**Therapeutic Strategy** Continue to collect and use data despite the intercurrent event	

Population-level Summary: Difference between the test group and control group in all-cause mortality on D28.

2.2.2 Secondary Estimands

Population: The target population of this trial is patients with severe SARS-CoV-2 infection. Refer to Section 4 "Study Population" for specific inclusion requirements. The target population of estimand 6 is severe SARS-CoV-2 infected patients with mechanical ventilation withdrawal

during the trial; the target population of estimands 8–10 is severe SARS-CoV-2 infected patients with positive baseline nucleic acid testing.

Variable:

1) Whether the subject is discharged on D28;
2) Whether the subject dies on D14 and D56;
3) Time to sustained clinical improvement in subjects (days; range: D0–D28); sustained clinical improvement is defined as patient discharge (discharge criteria [26]: body temperature returning to normal for at least 3 days, significant improvement in respiratory symptoms without oxygen support, and two consecutive negative nucleic acid test results at least 24 hours apart) or a reduction of at least 2 points on the Ordinal Scale for Clinical Improvement (see Appendix 1);
4) Number of days with oxygen supplementation for the subject (days);
5) Number of days with mechanical ventilation for the subject (days);
6) Whether mechanical ventilation is provided again 24 hours after withdrawal of mechanical ventilation for the subject, and the duration of the second mechanical ventilation (days);
7) Length of stay in ICU (days) and hospitalization time of the subject (days);
8) Time to SARS-CoV-2 nucleic acid negative conversion of subjects;
9) Whether the subject's SARS-CoV-2 nucleic acid test result converts to negative on d7, d14 and d28;
10) Change from baseline in SARS-CoV-2 viral load on d7, d14 and d28;
11) Whether the subject's lung imaging is improved; Improvement in lung imaging is defined as any of the following changes from baseline in lung imaging during the study: 1) reduction in lesion diameter; 2) decrease in the number of lesions; 3) decrease in the number of lung lobes involved by lesions

Treatment: Subjects in the test group will receive Meplazumab and standard of care (SoC), while subjects in the control group will receive placebo and SoC. Refer to Section 6 "Dosage and Method of Administration" for the specific treatment regimen.

Intercurrent Events and Handling Strategies: The main intercurrent events and handling strategies in the study are defined as follows:

Table 3 Intercurrent Events of Secondary Estimand and Handling Strategies

Intercurrent Events	Handling Strategies	Remarks
Early discontinuation of treatment due to poor efficacy	**Therapeutic Strategy**<br>Continue to collect and use data despite the intercurrent event	Reflect actual clinical condition
Early discontinuation of treatment due to adverse events	**Therapeutic Strategy**<br>Continue to collect and use data despite the intercurrent event	Reflect actual clinical condition
Concomitant use of prohibited medications/treatments affecting efficacy evaluation	**Therapeutic Strategy**<br>Continue to collect and use data despite the intercurrent event	Reflect actual clinical condition
Death	Secondary estimands 1: Composite strategy, handle as "not discharged".<br>Secondary estimands 3 and 8: Hypothetical strategy, perform "censoring" according to the time of the last follow-up.<br>Secondary estimands 4–7: Therapeutic strategy, handle according to the actual observation days.<br>Secondary estimands 9: Composite strategy, handle as "no negative conversion".<br>Secondary estimand 10: Therapeutic strategy, handle according to the actual observed value.<br>Secondary estimand 11: Composite strategy, handle as "not improved".	

Population-level Summary:

1) Difference between the test group and control group in discharge rate on D28;
2) Difference between the test group and control group in mortality on D14 and D56;
3) Hazard ratio of time to sustained clinical improvement between the test group and control group;
4) Difference between the test group and control group in days of oxygen supplementation;
5) Difference between the test group and control group in days of mechanical ventilation;
6) Difference between the test group and control group in the incidence of the second mechanical ventilation 24 hours after withdrawal of mechanical ventilation and the duration of the second mechanical ventilation;
7) Difference between the test group and control group in ICU days and hospitalization days;

8) Hazard ratio of time to SARS-CoV-2 nucleic acid negative conversion between the test group and control group;
9) Difference between the test group and control group in negative conversion rate of SARS-CoV-2 nucleic acid test on D7, D14, and D28;
10) Difference between the test group and control group in the change from baseline in SARS-CoV-2 viral load on D7, D14, and D28;
11) Difference between the test group and control group in the proportion of subjects with improvement in lung imaging.

3 Study Design and Principle

3.1 Overall Design

This is a multicenter, double-blind, randomized, placebo-controlled add-on Phase III clinical trial to evaluate the safety and efficacy of Meplazumab for Injection in patients with severe SARS-CoV-2 infection. Neither the subject nor the investigator will know what is used (study drug or placebo), as the study drug/placebo will be prepared by an unblinded third party (e.g., pharmacist or nurse), and will be given by authorized blinded study site personnel.

Hospitalized patients with laboratory-confirmed SARS-CoV-2 infection will be included in the study. Subjects will be randomly stratified by age group (age <65 years or ≥ 65 years).

Subject recruitment will continue throughout the study until the total number of subjects planned to be randomized into the study is reached. Subjects will be randomized into either the treatment group or control group.

During hospitalization, all subjects will receive SoC matching COVID-19 hospitalization based on the medical judgment of the investigator.

A total of 352 subjects will be randomized in a 1:1 ratio to receive Meplazumab at 0.2 mg/kg or placebo.

3.2 Overview of Study Design

This study is a multicenter, double-blind, randomized, placebo-controlled add-on clinical trial to evaluate the safety and efficacy of Meplazumab + SoC vs. SoC by intravenous infusion in patients with severe SARS-CoV-2 infection.

Approximately 352 subjects will be enrolled.

3.3 Principle of Study Design

Given the rapidly evolving SoC for COVID-19, the primary treatment is supportive treatment

and based on the medical judgment of the physician treating the subject. At present, there are few reports of positive study results on COVID-19; however, based on early analysis of adaptive COVID-19 treatment trial (ACTT) data, Remdesivir received emergency use authorization from the United States Food and Drug Administration (FDA) for treating subjects with suspected or laboratory-confirmed SARS-CoV-2 infection and severe COVID-19. The reported results showed that subjects with "moderate" infection recovered faster after 5 days of Remdesivir treatment.

The United Kingdom RECOVERY trial evaluated 28-day mortality in hospitalized patients with COVID-19 who received a low dose of dexamethasone (6 mg) daily orally or IV for 10 days in addition to their conventional treatment. In the dexamethasone group, mortality was lower in patients who received invasive mechanical ventilation (29.3% vs. 41.4%) and patients with oxygen supplementation who did not receive invasive mechanical ventilation (23.3% vs. 26.2%) than in the standard care group. Results from the RECOVERY trial and other similar studies suggested that low-dose glucocorticoids may be beneficial in treating hospitalized COVID-19 patients, and clinicians considered this therapy.

Following the discovery of SARS-CoV in 2002, which caused a large global outbreak, there has been growing interest in developing specific treatment drugs. SARS-CoV subjects were treated with corticosteroids, interferon type 1 (IFN) preparations, convalescent patient plasma, ribavirin, lopinavir or ritonavir, and, except for ribavirin, many drugs were provided with in vitro pre-clinical data supporting their efficacy. Since the outbreak of SARS, novel therapeutic drugs (targeting viral access proteins, proteases, polymerases and methyltransferases) have passed tests, however, they have not yet demonstrated efficacy in clinical trials.

As this study is a multicenter, randomized, third-party blinded study, rigorous data on the safety and efficacy of Meplazumab in the treatment of COVID-19 will be obtained in addition to detailed information on an important subpopulation of COVID-19 that develops into respiratory disease before requiring mechanical ventilation.

Randomization and inclusion of the control group are essential to control unexpectedly introduced biases in study results, and are necessary to determine the safety and efficacy of Meplazumab for COVID-19 treatment. The use of a 1:1 drug-control randomization ratio is more conducive to obtaining potentially effective treatment outcomes. Clinical and virological data from enrolled subjects collected using standardized timelines and collection tools will

provide valuable information on the clinical course and incidence of severe COVID-19 in different cohorts of hospitalized adult subjects.

All study personnel responsible for decisions related to the study will be blinded by third parties in this study. A third-party blinding design is important to provide an objective assessment of the pharmacological effects of the study drug and minimize the impact of bias in the assessment.

4 Study Population

Patients diagnosed with severe SARS-CoV-2 infection as per the *Diagnosis and Treatment Protocol for COVID-19 Patients (Tentative 10th Version)* issued by the National Health Commission (NHC) of China.

4.1 Inclusion Criteria

Eligible subjects must meet all the following criteria:

(1) Male or female patients with age ≥ 18 years old;

(2) Patients diagnosed with severe SARS-CoV-2 infection as per the Diagnosis and Treatment Protocol for COVID-19 Patients (Tentative 10th Version) issued by NHC;

(3) Subjects who agree to take effective non-drug contraceptive measures from signing the ICF to 3 months after the end of the study;

(4) Subjects who are capable of comprehending the study, willing to participate in the study and sign the ICF (for incapable subjects whose participation is deemed beneficial by the investigator, their legal guardians may sign the ICF on their behalf, with explanations recorded in relevant documents such as original medical records).

4.2 Exclusion Criteria

Those who meet one or more of the following conditions will not be included in the study:

(1) Subjects with any abnormal physical examination findings, abnormal laboratory tests and/or any medical history that, in the judgment of the investigator, may compromise their safety by participating in the study;

(2) Subjects who use anti-SARS-CoV-2 drugs within 3 half-lives prior to the first dose or during the study, such as Paxlovid (Nirmatrelvir Tablets/Ritonavir Tablets [co-packaged], $t_{1/2}$: approx. 6.05 h), Azvudine Tablets ($t_{1/2}$: approx. 9 h), Molnupiravir Capsules ($t_{1/2}$: approx. 3.3 h), Amubarvimab/Romlusevimab Injection ($t_{1/2}$: approx. 45 d/75 d), human COVID-19 immunoglobulins ($t_{1/2}$: approx. 3–4 w) or convalescent

plasma ($t_{1/2}$: approx. 21 d), Deuremidevir Hydrobromide Tablets ($t_{1/2}$: approx. 4.80–6.95 h), Simnotrelvir Tablets/Ritonavir Tablets (co-packaged) ($t_{1/2}$: approx. 4.14 h), Baricitinib ($t_{1/2}$: approx. 12.5 h), Tocilizumab ($t_{1/2}$: approx. 21.5 d), Remdesivir ($t_{1/2}$ approx. 27 h), Leritrelvir ($t_{1/2}$: approx. 14.9 h), Favipiravir ($t_{1/2}$: approx. 4.5 h) and 2-deoxy-D-glucose ($t_{1/2}$: approx. 50 min);

(3) Patients diagnosed with critical SARS-CoV-2 infection as per the Diagnosis and Treatment Protocol for COVID-19 Patients (Tentative 10th Version) issued by NHC;

(4) Subjects with stage 4 severe chronic kidney disease or requiring dialysis (estimated glomerular filtration rate [eGFR] <30 mL/min/1.73 m^2), or an increase in serum creatinine of 44.2 μmol/L within 7 days, or oliguria (<400 mL/24 h) or anuria (<100 mL/24 h);

(5) Pregnant or lactating women;

(6) Subjects who will be transferred to a hospital that is not a participating site of the study within 72 hours;

(7) Patients known to be allergic to the study drug and its components;

(8) Subjects who have used live vaccines (live attenuated vaccines) within 2 weeks prior to randomization or during the study treatment and safety follow-up period;

(9) Subjects who are participating in another clinical study. They must undergo a washout period of 5 half-lives (depending on the investigational product used or 30 days from their participation in the previous study, whichever is longer);

(10) Subjects with total bilirubin (TBL) > 2 × upper limit of normal (ULN), or alanine aminotransferase (ALT) > 5 × ULN, or aspartate aminotransferase (AST) > 5 × ULN, or alkaline phosphatase > 5 × ULN;

(11) Subjects with platelet count < 50 × 109/L, or hemoglobin < 70 g/L;

(12) Patients with other factors deemed by investigators as not suitable for the trial.

Note: The investigator should ensure that subjects meet all inclusion criteria and do not meet any exclusion criteria at screening. Subjects will be excluded from the study if their status (including laboratory results) changes after screening but before the first dose, causing them to meet any exclusion criteria.

5 Treatment Assignment and Blinding

Eligible subjects will be randomized to the Meplazumab group or placebo group in a 1:1 ratio according to the principle of randomization, and subject randomization will be completed using an interactive web response system (IWRS). The randomization statistician will use SAS9.4 or higher version software to generate the Subject Randomization Schedule by stratified blocked randomization. The stratification factor is age (age < 65 years old or ≥ 65 years old), and the schedule will be imported into the IWRS by the system engineer. After the subjects are successfully screened, the authorized investigators will log in to the IWRS to randomize the subjects and generate a randomization number, obtain the treatment group assignment information of the subjects, and collect the drugs of the corresponding groups for the subjects according to the system prompt to complete drug preparation and injection; in case of drug damage and other conditions, the unblinded investigator may re-collect the drugs for the same group to complete drug preparation and injection for the subjects.

5.1 Study Blindness

In this study, the study drug should be dissolved with sterile water for injection and then mixed with 100 mL of 0.9% normal saline, while the control drug will be a placebo, and it is planned to directly use 0.9% normal saline. Double-blind in the strict sense cannot be achieved due to the different appearances of the two drugs. In this study, the traditional packaging blinding of the investigational product cannot be performed before the trial. To avoid the bias on the study results caused by people obtaining specific grouping information, the blindness assurance work plan in this study is mainly carried out as follows:

1) It is planned to set a blinded team and an unblinded team at the site level to conduct the study. The unblinded study personnel include drug management personnel, personnel logging into the IWRS to obtain treatment grouping information, and drug preparation personnel. The remaining study personnel will remain blinded.
2) The project management and study monitoring team will have blinded and unblinded monitors. The unblinded monitors will perform site-specific drug accountability and monitor whether the subjects' drug preparation and administration process meet the protocol requirements. The remaining study personnel will remain blinded.
3) Drug preparation: Unblinded personnel will log in to the IWRS to obtain treatment

grouping information and complete solution preparation before administration. After preparation, the appearance of drugs in both the test group and control group will be consistent to maintain blindness. The drug preparation personnel cannot disclose relevant information about therapeutic drugs to subjects and any other persons, nor participate in all subsequent study evaluation processes.

4) After the start of the trial, both study members in the blinded group and unblinded group should ensure that they strictly follow the protocol requirements to carry out trial operations within their respective responsibilities.

5.2 Emergency Unblinding

When the specific treatment group information of a subject needs to be obtained to treat the subject due to serious adverse events or other emergencies, an authorized investigator can perform emergency unblinding for the subject through the emergency unblinding module of the IWRS to obtain the specific grouping information of the subject. If possible, prior to unblinding the investigational product, the authorized investigator must notify the Principal Investigator and relevant personnel of the sponsor first, and obtain approval from the Principal Investigator before obtaining specific subject grouping information. If the sponsor is not contacted before emergency unblinding, the investigator must contact the sponsor within 24 hours after unblinding. The investigator should record the date and reason for the unblinding of the subject, as well as the operation process of unblinding in the source documents.

5.3 Blind Review and Unblinding

After all data are entered into the database and confirmed to be correct, the data manager will write a blind data review report and hold a data blind review meeting. At the data blind review meeting, the principal investigator, data manager, statistician, and monitor should confirm any protocol violations in the trial and their impact on the statistical analysis set, make decisions on the problems raised in the data blind review report, and finalize the data blind review report. The database will also be locked according to the process. Once the database is locked, the unblinding procedure can be started. The independent statistician for randomization will release the Subject Randomization Schedule to the project statistician for unblinded data statistical analysis according to the process.

6 Dosage and Method of Administration

6.1 Rationale for Dose Selection

Based on the previous Phase I-III clinical studies and clinical applications completed at home and abroad, combined with the mode of administration and mechanism of action of this product, and considering the safety of human administration, the single dose in this study is determined to be 0.2 mg/kg through comprehensive analysis. This dose is expected to have high efficacy and low risk of adverse reactions based on the following:

In the international multicenter clinical trial "A Multicenter, Seamless, Randomized, Third-Party-Blind, Clinical Trial to Evaluate the Safety and Efficacy of Meplazumab in Addition to Standard of Care for the Treatment of COVID-19 in Hospitalized Adults" completed in the United States, Brazil, Pakistan and Mexico, 167 (100.0%) subjects were randomized to 4 treatment groups at a ratio of 1:1:1:1 to receive either Meplazumab at 0.12 mg/kg, 0.2 mg/kg and 0.3 mg/kg or placebo. The trial results showed that compared with the placebo group, 0.12 mg/kg group and 0.3 mg/kg group, the 0.2 mg/kg group showed the highest clinical response rate, the most significant antiviral and anti-cytokine storm effects, the reduction of mortality and sustained clinical improvement were comparable to those in the placebo group, and the safety and tolerability were good. Therefore, the optimal dose of Meplazumab for the treatment of severe COVID-19 was comprehensively evaluated as 0.2 mg/kg. Detailed results are as follows:

(1) Efficacy

a) Response rate (D29): 46.3% (19/41) in the 0.12 mg/kg group on D29, 53.7% (22/41) in the 0.2 mg/kg group on D29, 50.0% (22/44) in the 0.3 mg/kg group on D29 and 46.3% (19/41) in the placebo group on D29. Compared with the placebo group, the response rate was comparable in the 0.12 mg/kg group and increased by 16.0% in the 0.2 mg/kg dose group and 8.0% in the 0.3 mg/kg dose group.

b) Antiviral effect: Viral load was effectively reduced and negative rate and negative conversion rate increased in all 3 dose groups of Meplazumab. In the early period after treatment, the viral load was reduced more significantly in the 0.2 mg/kg group than in the 0.3 mg/kg group. Compared with the placebo group, the negative rate was increased by 24.1% in the 0.2 mg/kg group and 40.6% in the 0.3 mg/kg group, which was a significant difference (p = 0.0363). On D29, the negative rate was

significantly increased in the 0.2 and 0.3 mg/kg groups compared with the placebo group; by D10, the 0.12 mg/kg group showed an increased negative conversion rate compared with the placebo group; by D29, the negative conversion rate was increased in both the 0.2 and 0.3 mg/kg groups by 36.9% and 29.5%, respectively, compared with the placebo group. The result in the 0.2 mg/kg group was superior to that in the 0.3 mg/kg group. Overall, the effect was significant in the 0.2 mg/kg group.

c) Anti-cytokine storm effect: Cytokine levels were reduced in all 3 dose groups of Meplazumab, with a more significant reduction on D8 and D9, and the most significant reduction was observed in the 0.2 mg/kg dose group. Compared with the placebo group, the levels of 12 cytokines (12/16) were reduced in the 3 dose groups of Meplazumab at multiple visit points, 6 in the 0.12 mg/kg group, 10 in the 0.2 mg/kg group and 10 in the 0.3 mg/kg group. From D2 to D29, 6 cytokines in the 0.12 mg/kg group, 9 cytokines in the 0.2 mg/kg group, and 6 cytokines in the 0.3 mg/kg group tended to decrease compared with the placebo group. The decrease in cytokines was statistically significant on D2 (IL-4, 0.2 mg/kg group, $p = 0.0365$), D8 (IL-8, 0.3 mg/kg group, $p = 0.0460$), and D9 (IL-8, 0.2 mg/kg group, $p = 0.0484$). Compared with baseline, the levels of 5 cytokines (IL-8, IL-15, IL-2Rα, IFN-γ and MCP-1) were significantly decreased in the 3 dose groups of Meplazumab mainly on D8 and D9 ($p < 0.05$), 3 in the 0.12 mg/kg dose group, 5 in the 0.2 mg/kg dose group and 4 in the 0.3 mg/kg dose group.

d) Mortality reduction: Compared with the placebo group, the mortality was reduced by 83.6% in the 0.12 mg/kg group ($p = 0.0150$), comparable in the 0.2 mg/kg group, and reduced by 37.7% in the 0.3 mg/kg group

e) Time to sustained clinical improvement (to D29): 17 (41.5%, 17/41) in the 0.12 mg/kg group, 13 (31.7%, 13/41) in the 0.2 mg/kg group, 13 (29.5%, 13/44) in the 0.3 mg/kg group, and 13 (31.7%, 13/41) in the placebo group achieved sustained clinical improvement. Compared with the placebo group, the sustained clinical improvement was comparable in the 0.2 mg/kg group, and increased by 30.9% in the 0.12 mg/kg group.

(2) Safety: The incidence of adverse events in the three dose groups (0.12 mg/kg, 0.2 mg/kg and 0.3 mg/kg) of Meplazumab was basically comparable to that in the placebo group. No new or unexpected safety findings were found. This drug had good safety and tolerability in human treatment.

In the multicenter II/III clinical trial "A Multicenter, Double-blind, Randomized, Placebo-controlled, Add-on Phase II/III Clinical Study on the Safety and Efficacy of Meplazumab for Injection in Patients with COVID-19" completed by Shanghai Public Health Clinical Center, Shenzhen Third People's Hospital and Public Health Clinical Center of Chengdu, 150 patients were randomized into Meplazumab group or placebo group at a ratio of 2:1. Subjects were dosed with Meplazumab at 0.2 mg/kg or placebo. The results showed that the negative conversion rate of the SARS-CoV-2 test was higher in the Meplazumab group than in the placebo group by D5 postdose (from D3 to D5 postdose, the negative conversion rate in the Meplazumab group increased by about 22.5%–96.1% compared with the placebo group); by D4–D8 postdose, the discharge rate in the Meplazumab group was higher than that in the placebo group (34.8%–145.1% higher than that in the placebo group on D4–D8 postdose), indicating that Meplazumab at 0.2 mg/kg improved the early negative conversion rate and discharge rate of COVID-19 patients.

On December 8, 2022, Meplazumab was approved by the Health Bureau of the Logistics Support Department of the Central Military Commission for military special drugs (approval letter No.: JYZZ No. S2022001), the clinical data of 12 patients with severe COVID-19 using Meplazumab (single dose of 0.2 mg/kg, 3 subjects received 1 dose, 8 subjects received 2 doses (at an interval of 7 days) and 1 subject received 3 doses (at an interval of 7 days)) were collected and analyzed. The results showed that after treatment with Meplazumab, the body temperature of 12 patients returned to normal from 38–40°C before treatment. Symptoms such as pyrexia, headache, sore throat, chest pain, dyspnoea, cough, and fatigue were significantly improved. Blood oxygen saturation was recovered from 60–80% to 93–99%. CT images showed that lung shadows shrank or disappeared. Symptoms abated and oxygen saturation recovered to more than 95% in 5 of the patients within 12 hours postdose.

To sum up, optimal efficacy, high safety and good tolerability are obtained when the single dose of Meplazumab is 0.2 mg/kg. Therefore, the single dose in this study is determined as 0.2 mg/kg.

6.2 Method of Administration

Dissolve the antibody with 1 mL of sterile water for injection, calculate the dose required based on the dose determined in the previous clinical study (0.2 mg/kg) and the body weight of the patient, add to 100 mL of 0.9% sodium chloride for intravenous drip (use only 100 mL of 0.9% sodium chloride for intravenous drip in the placebo control group), and complete intravenous drip in 30–60 min.

Normal saline is selected as the placebo in this clinical study because there is no approved active control for severe SARS-CoV-2 infection and the study is a controlled study with SoC added.

6.3 End of Study

The study is deemed complete when the last visit is completed for the last subject or the last data point is collected for statistical analysis, whichever occurs later. The last subject refers to the last case planned to be enrolled, or the last case actually enrolled if the COVID-19 epidemic has ended and no additional cases meet the enrollment criteria and do not meet the exclusion criteria.

6.4 Treatment Regimen

The treatment plan includes a screening phase, a treatment phase, and an end of treatment phase. Treatment-related contents in each phase are as follows:

6.4.1 Screening Phase

After the ICF is signed, relevant items will be screened according to the study protocol (refer to the Study Schedule). Subjects who pass the screening will proceed to the next phase.

6.4.2 Treatment Phase

Administration (D0, D7)

- Physical examination: Pre-dose;
- Vital signs: Pre-dose and at 1 hr ± 10 min, 6 ± 1 hr and 12 ± 1 hr postdose;
- Dissolve the antibody with 1 mL of sterile water for injection, calculate the dose required based on the dose determined in the previous clinical study (0.2 mg/kg) and the body weight of the patient, and add to 100 mL of 0.9% sodium chloride for intravenous drip (use only 100 mL of 0.9% sodium chloride for intravenous drip in the placebo control group).

6.4.3 End of Treatment

Subjects who develop a fatal allergic reaction during treatment should be treated symptomatically and managed in accordance with the emergency plan. Oxygen supplementation, bronchodilators, epinephrine, antihistamines or glucocorticoids should be given for symptomatic treatment. During the treatment, the vital signs of patients should be closely observed and actively evaluated until all indicators of the subject are stable.

7 Medication Compliance

Meplazumab should be used under the supervision of the Principal Investigator or other study personnel. The administration of all study drugs should be recorded in the CRF. The reason(s) for medication interruption, dose reduction, or missing doses will also be recorded in the CRF. Such information and drug accountability of all study drugs in each period will be used to assess treatment compliance.

8 Concomitant Therapy

Concomitant therapies include all drug therapies and obvious non-drug therapies (including physiotherapy and blood or platelet transfusion) used after admission to the end of this clinical study. Drugs include not only those prescribed by doctors, but also all OTC drugs, vitamins, TCM decoctions, Chinese patent medicines and so on. All should be recorded in CRF.

8.1 Permitted Drug Therapies for Subjects During the Study

On the premise of protecting the interests and safety of subjects, the investigators should continue to provide standardized treatment for subjects in accordance with the requirements of the *Diagnosis and Treatment Protocol for COVID-19 Patients (Tentative 10th Version)* issued by the National Health Commission during the study. Necessary drugs and necessary therapies can also be used according to the patient's condition. The information on concomitant medications received by subjects during this study and symptomatic treatment should be recorded in the CRF.

8.2 Contraindicated Medications for Subjects During the Study

The investigators should continue to provide standardized treatment for subjects in accordance with the requirements of the *Diagnosis and Treatment Protocol for COVID-19 Patients (Tentative 10th Version)* issued by the National Health Commission during the study. If serious adverse reactions occur during treatment, they will be handled according to relevant clinical

guidelines.

Contraindicated medications for subjects during the study: anti-SARS-CoV-2 drugs (such as Paxlovid (Nematvir Tablets/Ritonavir Tablets co-package), Azvudine Tablets, Molnupiravir Capsules, Amubarvimab/Romlusevimab Injection, COVID-19 human immunoglobulin or convalescent patient plasma, Deuremidevir Hydrobromide Tablets, Simnotrelvir Tablets/Ritonavir Tablets (Co-packaged), **baricitinib, tocilizumab, remdesivir,** leritrelvir, favipiravir, and 2-deoxy-D-glucose).

9 Study Evaluation

9.1 Efficacy Evaluation

The following clinical support indicators should be assessed on each study day during hospitalization:

- Hospitalization
- Survival status
- Oxygen demand - Days of low-flow oxygen supplementation (< 40%)/days of high-flow oxygen supplementation (> 40%)
- Non-invasive mechanical ventilation (via mask)
- Invasive mechanical ventilation (via endotracheal intubation or tracheotomy)
- Requirement for extracorporeal membrane oxygenation (ECMO)

Definition of negative nucleic acid conversion: For patients with positive baseline SARS-CoV-2 nucleic acid testing, negative nucleic acid conversion is judged by two consecutive negative nucleic acid tests (sampling time should be at least 24 hours apart), and the sampling time of the first negative nucleic acid test is taken as the time to negative nucleic acid conversion.

Definition of time to sustained clinical improvement in the study: The patient is discharged (discharge criteria [26]: body temperature returns to normal for at least 3 days; significant improvement in respiratory symptoms without oxygen support; two consecutive negative PCR tests at least 24 hours apart) or a reduction of at least 2 points on the Ordinal Scale for Clinical Improvement (see Appendix 1).

Improvement in lung imaging is defined as any of the following changes from baseline in lung imaging during the study: 1) reduction in lesion diameter; 2) decrease in the number of lesions; 3) decrease in the number of lung lobes involved by lesions [3, 4]

9.2 PD Evaluation

In the study, blood samples for PD will be collected pre-dose, on D1 and D4, before the second infusion (D7), on D8, D10, and D28 (optional). If the second dose is not administered, no sample will be collected on D7, D8 and D10. Collected blood samples will be sent to the laboratory for PD analysis, so as to evaluate changes in levels of cytokines and chemokines associated with inflammatory and immune states after treatment with Meplazumab compared with D0 (pre-dose), including CRP, IL-2, IL-4, IL-6, INF-α, IL-8, IL-10, IL-12p70, IL-17, IL-1β, TNF-α and IFN-γ. The changes in lymphocyte subsets CD3+, CD4+ and CD8+ compared with D0 (pre-dose) will also be tested.

9.3 Evaluation of Safety and Immunogenicity

All subjects will be observed and recorded for any AEs/serious adverse events (SAEs) occurring during the study, including clinical symptoms, clinically relevant abnormal physical signs, abnormal laboratory tests and abnormal electrocardiogram (ECG) findings. The correlation between these events and the study drug will be judged. AEs will be followed up until they are recovered/returned to normal, returned to baseline levels, stable or deemed no longer necessary for follow-up based on clinical judgment. Besides, AEs will be evaluated as per CTCAE v5.0.

Infusion safety will be monitored before and after each dose by testing blood pressure (systolic and diastolic), pulse rate and blood oxygen saturation SpO_2 or oxygenation index, respiration rate and body temperature before each dose (within 30 min) and at 1 h ± 10 min, 6 h ± 1 h and 12 h ± 1 h after each dose. Hematology, blood chemistry, urinalysis, chest imaging, ECG and other indicators will be measured post-dose in the treatment period.

Blood samples will be collected pre-dose and post-dose (on D28 and D56) and tested for anti-drug antibodies (ADAs) to evaluate immunogenicity.

10 Early Termination of Study

The sponsor may terminate the study at any time. The termination of the study must be reported to the Independent Ethics Committee (IEC) and Institutional Review Board (IRB). Reasons for early termination of the study include, but are not limited to, the following:

(1) Drug regulatory authority, ethics committee, sponsor, or investigator believes that the therapeutic drug has significant safety risks;

(2) The sponsor may terminate the study for any scientific, medical, or ethical reason, but the rights, safety, and health of subjects must be fully considered;

(3) Other reasons judged by the sponsor or investigator to be unsuitable for continuing the study.

11 Subject Completion/Withdrawal

11.1 Completion

If a subject completes all evaluations within 28 days after the first dose according to the study protocol or dies, the subject will be considered as a subject who has completed the study.

11.2 Early Termination/Withdrawal

Withdrawal Decided by Investigator

Withdrawal from the study refers to a situation where an enrolled subject is deemed unsuitable to continue participation during the course of the study, and the investigator decides to withdraw the subject from the study.

(1) In the clinical trial, the subject experiences some comorbidities, complications or deterioration of the condition and is not suitable for further participation in the study;

(2) Subjects do not comply with the medical advice to use other treatments, which affects evaluation;

(3) The subject experiences adverse events or serious adverse events and is not suitable for further participation in the study;

(4) Poor compliance of subjects, affecting efficacy and safety judgment;

(5) Subject pregnancy;

(6) Other reasons based on which the investigator judges that the subject is not suitable for further participation in the study.

Withdrawal Decided by Subject

According to the informed consent form, if subjects are not willing to continue to participate in the clinical study, they have the right to withdraw at any stage of the study, or if subjects are lost to follow-up due to no further administration and tests, although they do not explicitly request to withdraw, it is also considered as "withdrawal" (or "drop-out"). The reasons for withdrawal should be investigated as much as possible and recorded. For example: subjects are intolerant of some adverse reactions, unable to further participate in the clinical study for other reasons, or lost to follow-up without any explanations.

Handling of Withdrawals

The investigator must fill in the reason for withdrawal in the case report form, contact the

patient if possible, complete all assessment items that can be completed, fill in the end-of-treatment follow-up record form, and record the time of the last dose if possible. Those who withdraw due to adverse events that are finally judged to be related to the therapeutic drug after follow-up must be recorded in the case report form, and the sponsor should be informed.

All existing study-related toxicities and SAEs at the time of withdrawal from the study must be followed up until they are resolved or corresponding indicators return to baseline levels, unless in the opinion of the investigator, remission is unlikely due to the patient's disease.

After the patient terminates the study treatment, the investigator should try to follow up all existing or new AEs that occur within 28 days after the last infusion of Meplazumab. All new AEs and SAEs occurring within this time frame should be reported. SAEs must be reported to the sponsor within 24 hours and followed up until they are resolved or corresponding indicators return to the baseline levels as described above. The Investigator should notify the sponsor immediately after a patient terminates the study. For any SAE, the sponsor should be contacted in time according to the corresponding SAE reporting procedure.

11.3 Case Report Form (CRF)

All CRFs must be completed, modified and replaced by the investigator or his/her authorized personnel. Queries will be generated by the EDC system. The investigator or his/her authorized personnel must answer or correct the queries sent.

11.4 Record Retention

According to ICH-GCP guidelines, the investigator/study site should maintain all CRFs, source documents of data collected from each subject, and all study documents specified in Section 8 of ICH-GCP and current regulations. The investigator/study institution should take measures to prevent these documents from being accidentally or prematurely destroyed.

12 Statistical Analysis

12.1 Analysis Data Set

The primary estimand will be analyzed mainly based on the Full Analysis Set (FAS). FAS includes all randomized subjects who receive at least one dose of the investigational product, as per the intention-to-treat principle. In addition, it will be analyzed based on the Per-Protocol Set (PPS), which will exclude subjects who do not complete treatment according to the protocol or have major protocol violations from the FAS.

Secondary estimands will be analyzed based on the FAS and PPS.

Safety analysis will be conducted based on the Safety Set (SS), which includes all randomized subjects who receive at least one dose of the investigational product.

All the above analysis sets will be jointly discussed and decided by the sponsor, principal investigator, statistician, and data manager during the data blinding review meeting prior to database lock.

12.2 Hypothesis Testing

- Hypothesis testing will be performed on all-cause mortality on D28 postdose in subjects with severe SARS-CoV-2 infection, i.e.,

Null hypothesis H_0:$\pi_C \le \pi_T$,

Alternative hypothesis H_1:$\pi_C > \pi_T$

Where, π_T,π_C represent the all-cause mortality in the Meplazumab group and placebo group, respectively, with one-sided α=0.025 used for the test level.

12.3 General Principles for Statistical Analysis

The statistical analysis will be performed using SAS 9.4 or higher version.

The baseline result is defined as the last non-missing test result prior to the first dose of Meplazumab.

Statistical description will be performed using continuous variables, including number of cases (non-missing), mean, standard deviation (SD), median, maximum, and minimum. Categorical variables will be statistically described using the summary of frequency and percentage. Unless otherwise specified, the number of missing subjects will not be included in the calculation of percentage.

Demographic data and baseline indicators will be analyzed in the FAS population. All demographic variables and baseline characteristics (e.g., gender, date of birth, weight, disease category, concomitant medications, prior therapy history, vital signs, etc.) are summarized by dose group.

12.4 Efficacy Analysis

12.4.1 Analysis of Primary Estimand

For the Meplazumab and placebo groups, the all-cause mortality on D28 post-dose will be calculated. The Clopper-Pearson method will be used to calculate its two-sided 95% confidence interval (CI). CMH Chi-square test will be used for the statistical test of inter-group difference, with age (< 65 years or ≥ 65 years) as the stratification factor. Meanwhile, the difference in the

all-cause mortality on D28 post-dose (placebo group-Meplazumab group) and its two-sided 95% CIs will be calculated using the CMH method, with age (< 65 years or ≥65 years) as the stratification factor. Also, Miettinen & Nurminen's method without considering stratification factors will be used to calculate the difference between the groups (placebo group-Meplazumab group) and its two-sided 95% confidence interval (CI). The chi-square test/Fisher's exact test without considering stratification factors will be used for statistical testing of the difference between the two groups.

Logistic regression model will be fitted to calculate the odds ratio (OR) between the Meplazumab group and the placebo group as well as the 95% CI. The model will include all-cause mortality on D28 postdose as the dependent variable and group and age group (< 65 years vs. ≥ 65 years) as fixed effects.

Also, TPA (tipping point analysis) will be used to perform sensitivity analysis on missing data and evaluate the impact of missing data on the robustness of test results.

Supplementary analysis: Efficacy evaluation will be performed with intercurrent events "early discontinuation of treatment due to poor efficacy", "concomitant use of prohibited medications/treatments affecting efficacy evaluation", and "disease aggravation" analyzed as "death" according to the composite strategy.

12.4.2 Analysis of Secondary Estimands

(1)Discharge rate on D28 after the first dose

The discharge rate on D28 after the first dose will be calculated for the Meplazumab group and placebo group, respectively. The two-sided 95% CIs will be calculated using the Clopper-Pearson method. The difference in discharge rate on D28 postdose (placebo group-Meplazumab group) and the two-sided 95% CIs will be calculated using the CMH method considering stratification factor (age group (< 65 years vs. ≥ 65 years)).

(2) All-cause mortality on D14 and D56 after the first dose

The all-cause mortality on D14 and D56 after the first dose will be calculated for the Meplazumab group and placebo group, respectively. The two-sided 95% CIs will be calculated using the Clopper-Pearson method. The difference in all-cause mortality on D14 and D56 postdose (placebo group-Meplazumab group) and the two-sided 95% CIs will be calculated using the CMH method considering stratification factor (age group (< 65 years vs. ≥ 65 years)).

(3) Time to sustained clinical improvement (days)

Kaplan-Meier method will be used to calculate the improvement rates at different time points and the lower quartile, median, and upper quartile of time to sustained clinical improvement and their two-sided 95% CIs (Greenwood method: log-log transformation) in the Meplazumab group and placebo group, respectively, and stratified log-rank test will be used for statistical testing of differences between the groups. Also, the Cox model with the time to sustained clinical improvement as the dependent variable, and group and stratification factor age group (< 65 years old vs. ≥ 65 years old) as the fixed effects will be used to estimate the hazard ratio between the groups (Meplazumab group-placebo group) as well as the two-sided 95% CI. Kaplan-Meier curves will be plotted for the time to sustained clinical improvement in the Meplazumab group and placebo group.

(4) Number of days with oxygen supplementation (days)

Days of oxygen supplementation (including cumulative days of oxygen supplementation, days of low-flow oxygen supplementation and days of high-flow oxygen supplementation) in the Meplazumab group and placebo group will be statistically described, respectively, and the difference between the groups (placebo group-Meplazumab group) and its two-sided 95% CIs will be calculated.

(5) Number of days with mechanical ventilation (days)

The days of mechanical ventilation in the Meplazumab treatment group and placebo group will be statistically described, respectively, and the difference between the groups (placebo group-Meplazumab group) and its two-sided 95% CIs will be calculated.

(6) Incidence and duration of re-ventilation 24 hours after withdrawal (days)

The incidence and duration of the second mechanical ventilation 24 hours after withdrawal of mechanical ventilation in the Meplazumab group and placebo group will be statistically described, respectively, and the difference between the groups (placebo group-Meplazumab group) and its two-sided 95% CIs will be calculated.

(7) Length of stay in ICU and hospitalization time (days)

The ICU time and hospitalization time in the Meplazumab group and placebo group will be statistically described, respectively, and the difference between the groups (placebo group-Meplazumab group) and its two-sided 95% CIs will be calculated.

(8) Time to negative nucleic acid conversion

Kaplan-Meier method will be used to calculate the lower quartile, median and upper quartile of

time to negative conversion as well as their two-sided 95% CIs (Greenwood method: log-log transformation) in the Meplazumab group and placebo group, respectively, and stratified log-rank test will be used for statistical testing of differences between the groups. Also, the Cox model with the time to negative conversion as the dependent variable, and group and stratification factor age group (< 65 years old vs. ≥ 65 years old) as the fixed effects will be used to estimate the hazard ratio between the groups (Meplazumab group-placebo group) as well as the two-sided 95% CI. Kaplan-Meier curves will be plotted for the time to negative conversion in the Meplazumab group and placebo group.

(9) SARS-Cov-2 nucleic acid negative conversion rate on D7, D14 and D28

The SARS-CoV-2 nucleic acid negative conversion rate on D7, D14 and D28 in the Meplazumab group and placebo group will be statistically described, respectively, and the difference between the groups (placebo group-Meplazumab group) and its two-sided 95% CIs will be calculated.

(10) Change from baseline in SARS-CoV-2 viral load on d7, d14 and d28

The changes from baseline in SARS-CoV-2 viral load (log 10) on D7, D14 and D28 in the Meplazumab group and placebo group will be statistically described, respectively, and the difference between the groups (placebo group-Meplazumab group) and its two-sided 95% CIs will be calculated.

(11) Lung imaging improvement rate

The lung imaging improvement rate on D7 and D14 after the first dose in the Meplazumab group and placebo group will be calculated, respectively, the 95% CIs will be calculated by the Clopper-Pearson method, and the difference between the groups (placebo group-Meplazumab group) and its two-sided 95% CIs will be calculated.

12.5 Pharmacodynamic Analysis

(1) Changes from baseline in cytokines and chemokines

Cytokines and chemokines on D1, D4, D7 (before the second dose), D8, D10 and D28 after the first dose and their changes from baseline in the Meplazumab group and placebo group will be statistically described, respectively.

(2) Change from baseline in lymphocyte subsets

Lymphocyte subsets on D1, D4, D7 (before the second dose), D8, D10 and D28 after the first dose and their changes from baseline in the Meplazumab group and placebo group will be

statistically described, respectively.

12.6 Immunogenicity Analysis

The positive rate and cumulative positive rate of anti-drug antibody (ADA) in the Meplazumab group and placebo group at each time point before and after treatment will be calculated, respectively, and the two-sided 95% CIs will be calculated by the Clopper-Pearson method.

12.7 Safety Analysis

12.7.1 Adverse Events (AEs)

AEs will be medically coded using MedDRA 26.0 or the latest version. Classified statistics will be performed by SOC and PT. In this trial, the treatment-emergent adverse events (TEAEs) will mainly be statistically analyzed; pretreatment AEs will be presented in a list. Unless otherwise specified, adverse events below are TEAEs.

The number of events, number of subjects and incidence of the following AEs will be calculated for subjects in the Meplazumab group and placebo group, respectively:

- **All TEAEs;**
  - Treatment-related AEs (TRAEs);
- **AEs of different severities;**
  - TRAEs of different severities;
- **AEs with Incidence ≥ 1% in Any Group;**
  - TRAEs with incidence ≥ 1% in any group;
- **AEs with incidence ≥ 5% in any group;**
  - TRAEs with incidence ≥ 5% in any group;
- **Serious Adverse Events (SAEs);**
  - Treatment-related SAEs;
- **AEs leading to withdrawal from the trial;**
  - TRAEs leading to withdrawal from the trial;
- **AEs leading to death;**
  - TRAEs leading to death;
- **AEs leading to discontinuation;**
  - TRAEs leading to discontinuation;
- **AEs leading to dose reduction;**
  - TRAEs leading to dose reduction;

- **AEs leading to dose interruption;**
  - ✧ TRAEs leading to dose interruption

The severity and correlation of adverse events will be tabulated.

When calculating the incidence of AEs in each group, count multiple occurrences of the same adverse event in a single subject as one event. When calculating the number of cases, count multiple occurrences of the same adverse event in a single subject as multiple events. For the analysis of the severity and drug correlation of adverse events, if a subject experiences the same adverse event multiple times, the most severe occurrence or the one most related to the drug should be used for analysis.

AEs will be listed separately.

12.7.2 Laboratory Tests

Baseline values of laboratory test indicators (including hematology, blood chemistry, urinalysis, and coagulation) and changes from baseline to the worst grade will be described in a shift table (based on the normal range and investigator's judgment of clinical significance) by Meplazumab group and placebo group.

The test results and abnormalities of all laboratory tests during the study will be listed.

12.7.3 Physical Examination

Baseline values of physical examination indicators and changes from baseline to the worst grade will be described in a shift table (based on the normal range and investigator's judgment of clinical significance) by Meplazumab group and placebo group.

All physical examination results and abnormalities during the study will be listed.

12.7.4 Vital Signs

The vital signs in the Meplazumab group and placebo group will be statistically described, respectively.

Baseline values of vital signs examination indicators and changes from baseline to the worst grade will be described in a shift table (based on the normal range and investigator's judgment of clinical significance) by Meplazumab group and placebo group.

The test results and abnormalities of vital signs during the study will be listed.

12.7.5 12-lead ECG

Baseline values of 12-ECG indicators and changes from baseline to the worst grade will be described in a shift table (based on the normal range and investigator's judgment of clinical

significance) by Meplazumab group and placebo group.

The test results and abnormalities of all 12-lead ECG examinations during the study will be listed.

12.8 Interim Analysis

Interim analysis will not be performed in this study.

12.9 Subgroup Analysis

If supported by data, subgroup analysis of the primary efficacy endpoint by age (< 65 years or ≥ 65 years), sex (M or F), concomitant use of antiviral drugs (Yes or No), SARS-CoV-2 vaccination status (Yes or No), underlying disease (Yes or No), BMI ≥ 30 (kg/m^2) (Yes or No), and smoking (Yes or No) will be performed, with a forest plot plotted.

12.10 Multiplicity Handling

There is only one primary endpoint for this trial; therefore, type I error correction is not required.

12.11 Handling of Missing Data

In this study, missing data for the primary efficacy endpoint on D28 after administration will be imputed according to the "status of the last follow-up". The handling of missing data for secondary efficacy endpoints will be detailed in the SAP.

12.12 Determination of Sample Size

Based on the available clinical study results and dose justification in Section 6.1, subjects in the 0.2 mg/kg dose group will enter Phase III clinical trial to compare with placebo + SoC. Previous results showed a mortality of 2.4%, 14.6%, 9.1%, and 14.6% in the 0.12 mg/kg, 0.2 mg/kg, 0.3 mg/kg, and placebo groups, respectively. Considering the mortality of the 3 dose groups in the previous clinical studies and the possibility of inherent variability in the data. Estimating the mortality in the placebo group as 15%, the mortality in the 0.2 mg/kg group is expected to decrease by 10% compared with the placebo group. The one-sided test α is 0.025, and the power is set at 0.8. It is calculated using the "Tests for Two Proportions" module in PASS 21 that at least 141 subjects are required for each group. Considering a 20% dropout rate, 176 subjects per group are required, for a total of 352 subjects.

13 Adverse Event Reporting

Timely, accurate and complete reporting and analysis of safety information from clinical studies is critical to the protection of subjects, investigators, and the sponsor, and is also a mandatory

requirement for regulatory authorities. The sponsor has established standard operating procedures (SOPs) according to the requirements of *Good Clinical Practice* (No. 57, 2020), *Provisions for Adverse Drug Reaction Reporting and Monitoring* (Ministry of Health Order No. 81), and *Provisions for Drug Registration* (SAMR Order No. 27) to ensure appropriate reporting of safety information.

All clinical studies initiated by the sponsor are required to report AEs in accordance with these SOPs.

13.1 Relevant Definitions

13.1.1 Pretreatment Event

Pretreatment event (PTE): Any untoward medical event in a clinical study subject who signs the ICF to participate in the study, but which occurs before the administration of any study drug; the event does not necessarily have a causality with participation in the study.

13.1.2 Definition and Classification of Adverse Events

- **Adverse Events (AEs)**

An adverse event (AE) refers to any adverse medical event, experienced by a subject administered an investigational product, which may present with symptoms, signs, diseases, or laboratory test abnormalities but does not necessarily have a causal relationship with the investigational product. The investigator must report all AEs in the eCRF. All AEs occurring during the study should be recorded in the CRF.

- **Serious Adverse Events (SAEs)**

A serious adverse event (SAE) is any untoward medical event in a subject after administration of the investigational product that meets one or more of the following criteria:

(1) Resulting in death;
(2) Result in life-threatening consequences;
(3) Resulting in hospitalization or prolongation of existing hospitalization;
(4) Resulting in significant or permanent disability/incapacity;
(5) Resulting in congenital anomaly or birth defect;
(6) Resulting in other important medical events: For example, important medical events that are not immediately life-threatening, or fatal, or require hospitalization but may jeopardize the patient, or require medical intervention to prevent the outcomes listed

above, scientific medical judgment should be made immediately. These should also be considered SAEs.

The serious adverse event is further explained as follows:

(1) Death due to any AE occurring during the study. If a subject dies during the study with an autopsy performed, the autopsy results should be included in the subject's CRF.

(2) The occurrence of an AE will immediately place the subject at risk of death. AEs that may result in death after serious progression (e.g., drug-induced hepatitis without hepatic failure) are excluded.

(3) AEs that result in hospitalization or prolongation of hospitalization (prolongation of hospitalization is defined as a delay in the planned or expected date of discharge, usually at least 1 day overnight in the hospital). This does not include elective surgeries or admission examinations decided before the trial, and the unchanged treatment course during the study.

(4) Any AE that results in the impairment, damage or disruption of the subject's function, physiological structure, or both, affecting physical activity or quality of life.

(5) It is suspected that exposure of either parent to the study drug will result in adverse outcomes in the offspring.

- **Significant AEs**

Significant adverse events refer to any adverse events other than serious adverse events that result in the use of targeted medical measures (e.g., discontinuation, dose reduction, and symptomatic treatment) and other significant abnormalities in hematology or other laboratory tests.

- **Adverse Drug Reactions**

Adverse drug reaction (ADR) refers to reactions harmful to the human body or unintended reactions that are considered to be related to the investigational product in a clinical trial. The investigational product and the AE are at least in one possibly reasonable causality, i.e., the correlation cannot be ruled out. .

13.2 Additional Considerations for PTEs and AEs

Generally, for the unfavorable results:

(1) Indication of a new diagnosis or an unexpected aggravation of a pre-existing condition. An intercurrent event due to pre-existing underlying conditions should not be considered a PTE or AE;
(2) Therapeutic intervention is required;
(3) Invasive diagnostic procedures are required;
(4) Discontinuation or change in the dose of study drug or concomitant medication is required;
(5) Considered unfavorable by the investigator for any reason;
(6) A PTE/AE caused by study procedures (e.g., bruises after blood collection) should be recorded as a PTE/AE. Signs and symptoms before and after the study procedure should be compared:
(7) Each event should be recorded as an individual diagnosis. Concomitant signs (including abnormal laboratory test values or ECG findings) or symptoms should not be recorded as additional AEs. If the diagnosis is unknown, the signs or symptoms may be recorded as PTE or AE accordingly.

Laboratory Values and ECG Results:

(1) A change in laboratory value or ECG parameter will be considered a PTE or AE only if it is judged as a clinically significant abnormality (i.e., if certain actions or interventions need to be taken, or if the investigator concludes that the change is outside of the normal range of physiological fluctuation). Laboratory retests and/or ongoing monitoring of abnormal values are not considered interventions. In addition, repeated or additional non-invasive tests performed to validate, evaluate or monitor an abnormality are not considered as interventions.
(2) If an abnormal laboratory test value or ECG finding is a pathological finding of an overall diagnosis (e.g., an increase in creatinine in kidney failure), only this diagnosis should be reported as an AE or a PTE accordingly.

Pre-existing Conditions:

(1) Pre-existing conditions (present at the time of ICF signing) will be considered concomitant conditions, and should not be recorded as a PTEs or AEs. Baseline evaluations (e.g., laboratory tests, ECGs, and x-ray) should not be recorded as PTEs unless associated with study procedures. However, if a subject experiences

aggravation of such concomitant condition or develops a complication, the aggravation or complication should be recorded as either a PTE (if the aggravation or complication occurs before the start of the study drug) or an AE (if the aggravation or complication occurs after the start of study drug), as appropriate. The Investigator should ensure that the event term recorded reflects a change in the condition (e.g., "aggravation of ...").

(2) If a subject has a pre-existing intercurrent condition (e.g., asthma and epilepsy), only an episode that becomes more frequent, more severe, or aggravates should be recorded as a PTE/AE, i.e., the Investigator should ensure that the recorded AE term can describe the change from baseline in the condition (e.g., "aggravation of ...").

(3) If a subject has a degenerative concomitant condition (e.g., cataract and rheumatoid arthritis), aggravation of the condition should be recorded as a PTE/AE only if it aggravates more than expected. In addition, the investigator should ensure that the recorded AE term describes a change in the condition (e.g., "aggravation of ...").

Aggravation of PTE or AE:

(1) If a subject experiences an aggravation or complication of PTE after commencing study drug administration, the aggravation or complication should be recorded as an AE accordingly. The investigator should ensure that the recorded AE term describes a change in the condition (e.g., "aggravation of ...").

(2) If a subject experiences an aggravation or complication of an AE following any change in study drug, the aggravation or complication should be recorded as a new AE. The investigator should ensure that the recorded AE term describes a change in the condition (e.g., "aggravation of ...").

Change in AE/PTE Severity:

If a subject experiences a change in the severity of an AE/PTE, the event should be recorded once at its maximum severity.

Pre-planned Surgery or Procedure:

(1) A pre-planned procedure (surgery or therapy) scheduled before the signing of the ICF will not be considered a PTE or AE. However, if the pre-planned procedure is performed earlier (e.g., as an emergency) due to the aggravation of a pre-existing condition, then the aggravation of the condition should be recorded as a PTE or AE

accordingly. Complications resulting from any planned surgery should be reported as AEs.

(2) Elective surgery or procedure: An elective surgery performed in the absence of a change in the subject's condition should not be reported as a PTE or AE, but should be recorded in the subject's source document. Complications due to elective surgeries should be reported as AEs.

(3) Insufficient clinical response (lack of efficacy): An insufficient clinical response, efficacy, or pharmacological effect should not be recorded as an AE. The Principal Investigator must distinguish between aggravation of a pre-existing condition and lack of therapeutic efficacy.

13.3 Definition of Causality

The causality between the investigational product and AEs will be judged according to the following criteria.

(1) Unrelated: The AE is unrelated to the investigational product; the timing of the reaction does not align with the administration of the drug, the reaction is consistent with the known reaction types of non-investigational products, the patient's clinical condition or other treatments may also cause the reaction, the reaction resolves with the improvement of the disease or the cessation of other treatments, the reaction recurs with the repeated use of other treatments, and there is a close correlation to other risk factors.

(2) Unlikely related: The AE is more likely explained by other factors; the timing of the reaction does not align with the administration of the drug, the reaction does not match the known reaction types of the investigational product, and the patient's clinical condition or other treatment may also cause the reaction. The relationship with administration cannot be ruled out.

(3) Possibly related: The AE may be related to the investigational product, and other explanations are not persuasive; the time of occurrence of the reaction is in accordance with the temporal sequence of administration, the reaction conforms to the known reaction type of the investigational product, and the clinical status or other treatment methods of patients may also produce this reaction.

(4) Probably related: The AE is probably related to the investigational product, possible correlation in time is suggested, and the possibility of other explanations is low;

(5) Related: The AE has been listed as a possible adverse drug reaction and there is no reason to use other explanations. The timing of the reaction aligns with the administration of the drug, the reaction matches the known reaction types of the study drug, the reaction improves upon dose reduction or discontinuation, and reoccurs upon re-administration.

13.4 Judgment Criteria for Severity

Severity of AEs will be assessed according to CTCAE v5.0.

Grade 1: Mild; asymptomatic or mild symptoms; clinical or diagnostic observations only; intervention not indicated;

Grade 2: Moderate; minimal, local or non-invasive intervention; limiting age-appropriate instrumental activities of daily living (ADL) (instrumental ADL refers to cooking, buying groceries or clothes, using the telephone, managing money, etc.);

Grade 3: Severe or with important medical significance, but not immediately life-threatening; hospitalization or prolongation of existing hospitalization; disabling; limiting self-care ADL (self-care ADL refers to bathing, dressing and undressing, feeding one-self, using the toilet, taking medications, but not bedridden);

Grade 4: Life-threatening consequences; urgent intervention indicated;

Grade 5: Death related to AE.

13.5 Actions with the Study Drug

(1) Discontinuation: Discontinue the study drug due to a specific AE;

(2) Dose unchanged: No need to discontinue the study drug due to a specific AE;

(3) Unknown: Used only when the actions to be taken cannot be determined;

(4) Not applicable: Discontinuation of the study drug due to reasons other than specific AEs, e.g., termination of the study, death of the subject, or the study drug being discontinued prior to the occurrence of the AE;

(5) Dose reduction: Reduce the dose due to a specific AE;

(6) Dose interruption: Temporarily interrupt (suspend) use of the study drug due to a specific AE, including voluntary interruption by the subject, and then resume the use.

13.6 Outcome of Adverse Events

(1) Recovered/resolved: The "end date of (serious) AE" should be indicated;

(2) Recovering/Resolving: The event is still not fully resolved, but the subject is in the recovery phase. Follow-up required;

(3) Not recovered/not resolved: The event is ongoing;

(4) Recovered/Resolved with Sequelae: Only if the subject has long-lasting or lifelong sequelae, such as blindness due to diabetes mellitus and hemiplegia after stroke. "End date of (serious) adverse event" should be noted;

(5) Death: "End date of (serious) AE" should be indicated for the death caused by an AE, and "end date of (serious) AE" is not required for death not caused by an AE. If the subject dies, the time of death should be recorded;

(6) Unknown: The Investigator is unable to know the AE, e.g., subject lost to follow-up.

If the outcome of an AE is rated as "recovering/resolving", or "not recovered/not resolved" or "unknown", the end date of the AE may not be recorded tentatively.

When the outcome of an AE is rated as "recovered/resolved" or "recovered/resolved with sequelae", the end date of the AE must be recorded.

All AEs must be followed up to determine the final outcome or until the stable status is reached or the subject is lost to follow-up.

After a subject completes the clinical study, the investigator should follow up on the outcome of AEs that may be related to the study drug or cannot be determined, or until the stable status is reached.

13.7 Collection and Reporting Procedures

13.7.1 Collection and Reporting Procedures of Pretreatment Events and All Adverse Events

Collection Period

PTEs will be collected from the time a subject signs the ICF to participate in the study to the time when the subject receives the first dose of the study drug or until screening failure. For a subject who discontinues the study before the reinfusion of the study drug, PTEs will be collected until the subject discontinues the study.

AE collection will begin when a subject receives the first dose of the study drug through the 28-day follow-up period after the subject's first dose. After the follow-up period, only spontaneous reports will be collected.

The end date of an AE/PTE is the date when the subject recovers, the event resolves with sequelae, or the subject dies due to the AE.

Reporting

The investigator will assess the occurrence of subjective AEs at each study visit. A neutral question such as "How have you been feeling since last visit?" may be asked. Subjects may report AEs occurring at any other time during the study. Subjects who experience PTEs must be monitored until the symptoms resolve or any clinically relevant changes in laboratory tests have returned to baseline values, or a satisfactory explanation for the changes can be made. Non-serious PTEs related or unrelated to study procedures do not require follow-up according to the objectives of the study protocol. All AEs experienced by subjects, regardless of their correlation to the investigational product, must be monitored until symptoms subside and any clinically relevant changes in laboratory test values return to baseline or are satisfactorily explained. All clinically significant laboratory abnormalities confirmed by repeat tests should be followed until they return to an acceptable level or are satisfactorily explained. All PTEs and AEs will be recorded on the PTE/AE page of the CRF, regardless of the relationship to the therapeutic drug judged by the investigator. The following information will be documented for each event:

(1) Event terms;
(2) Start and end dates and times;
(3) Severity;
(4) Investigator's judgment of causality between the event and administration of investigational product (related or not related) (not required for PTE);
(5) Investigator's judgment of causality between the event and study procedures, including details of suspected procedures;
(6) Actions taken with the investigational product (not applicable for PTE);
(7) Event results;
(8) Severity.

The subject diary and questionnaire will not be used as the primary means for collecting AEs. However, if the information gathered by the investigator from these documents identifies a potential AE, the patient should be followed up appropriately for medical evaluation. If, as a result of this visit, an AE that has not been previously reported is identified, it should be

reported in accordance with the normal reporting requirements.

13.7.2 Collection and Reporting of SAEs

For any SAE occurring in the trial, regardless of whether it is related to the investigational drug or not, the investigator should give timely rescue treatment. The investigator should fill in the *SAE Report Form* formulated for this trial as detailed as possible, sign and date. The investigator should report the SAE to the sponsor (or CRO designated by the sponsor) by email within 24 hours after becoming aware of it. The sponsor (or the CRO designated by the sponsor) will conduct an assessment, and then report the assessed suspected unexpected serious adverse reactions (SUSARs) to the principal investigator and project manager as well as their clinical trial institution and ethics committee in a rapid manner, and report to the national drug regulatory authority and health authorities. The investigator should sign and read the relevant safety information of the clinical study provided by the sponsor (or CRO designated by the sponsor) in a timely manner after receiving it, and report the SUSAR report provided by the sponsor (or CRO designated by the sponsor) to the ethics committee.

For SAEs, the description of symptoms, severity, occurrence time, treatment time, actions taken, follow-up time and mode, and outcome should be recorded in detail.

The investigator must report SAEs together with relevant assessment of causality. If the investigator's assessment of causality is lost or unavailable, the sponsor will judge the event until the investigator's assessment is finally available.

If the investigator cannot determine whether an AE is an SAE, the AE will be considered an SAE until its nature is proven otherwise. Such events will be notified in writing to local authorities and the relevant Investigators according to local requirements.

For all SAEs (including those that are still in the development stage after the end of the study and occur within 28 days after the end of the study), the investigator should follow up until there is a definite result to ensure that all problems are resolved. Detailed follow-up information should be provided (e.g., whether special treatment or hospitalization is required after the study).

The investigator will submit a follow-up report to the CRO until the resolution of the AE. In case of permanent impairment, follow-up is required until the event is considered stable.

Time limit requirements for expedited reporting:

(I) For SUSARs that are fatal or life-threatening, the sponsor (or the CRO designated by the sponsor) should report them as soon as possible, but not more than 7 days after initial awareness and submit a complete follow-up report within 8 days after the first report.

(II) For SUSARs that are not fatal or life-threatening, the sponsor (or the CRO designated by the sponsor) should report them as soon as possible, but not more than 15 days after initial awareness. After report submission, serious adverse reactions should continue to be followed up. Any new information or changes to the previous report should be promptly submitted in the form of a follow-up report, within 15 days of receiving the new information.

Reporting Route:

Center for Drug Evaluation, NMPA	
Transmission Mode	Transmit by Gateway of the PV system
National Health Authority	
E-mail	saefax@163.com
Jiangsu Pacific Meinuoke Bio-pharmaceutical Co., Ltd.	
E-mail	lss@pmbp.cn
Fourth Military Medical University	
E-mail	znchen@fmmu.edu.cn
Pharmacovigilance Department, Beijing CTSmed Co., Ltd.	
E-mail	pv@ctsmed.com

13.8 Death

All deaths occurring during the study or within 28 days after withdrawal from the trial (last visit) must be notified to the sponsor within 24 hours, and if the criteria for expedited reporting are met, the regulatory authority should be notified within 7 days, and relevant follow-up information (cause of death, autopsy report, and hospital report) should be reported within the next 8 days (15 days in total).

In case of withdrawal due to death, the event should be reported as a progressive disease (PD) or AE, and the cause of death should be recorded in the CRF. If death is caused by PD combined with other causes, the investigator must determine the primary cause of death and appropriately classify the reason for withdrawal.

13.9 Hospitalization

AEs that required hospitalization are considered SAEs. In general, if admission procedures are handled and treatment is given, the AE will be considered as an SAE.

Hospitalization for elective surgery, routine clinical procedures, annual physical examination, admission observation, or protocol requirements rather than AEs will not be considered an AE but should be recorded on the Clinical Assessment Form and the CRF. If an unexpected event occurs in this process, it will be reported as a "serious" or "non-serious" AE according to conventional criteria.

Note: Hospitalization or prolongation of hospitalization for non-medical reasons/convenience or purely for clinical trial purposes does not meet the criteria for a medical event and therefore cannot be considered an SAE.

13.10 Pregnancy

Pregnancy will be determined by serum pregnancy test. Subjects found pregnant at screening will be excluded from the study; subjects who become pregnant during the treatment period must withdraw from the study.

All pregnancy events must be reported. The investigator should fill in the *Pregnancy Event Report Form* of the clinical trial and submit it to the sponsor within 30 days after being informed of the pregnancy event. The investigator must follow up and record processes and outcomes of all pregnancies, even if the subject has withdrawn from the study or the study has ended.

The outcomes of all pregnancies must be reported. Pregnancy events in the trial should be tracked to the end of pregnancy or one month after the fetus's birth if possible, and follow-up information should be reported in the form of the *Pregnancy Event Report Form*.

Pregnancy alone will not be considered an AE unless it is suspected that the study drug may have affected the efficacy of contraceptives; elective abortion without complications should not be handled as an AE, except for therapeutic abortion. Hospitalization for a normal birth of a healthy newborn should not be considered an SAE.

Any SAE that occurs during pregnancy (including those occurring after the last dose of the study drug) must be recorded on the SAE Report Form (e.g., maternal serious complications, spontaneous or therapeutic abortion, ectopic pregnancy, stillbirth, death neonatal, congenital anomaly or birth defect) and reported within 24 hours according to the procedures for reporting SAEs.

13.11 Overdose

Any therapeutic dose in excess of that specified in the protocol should be recorded on the CRF as an overdose and whether there is any AE related to the overdose should be recorded. Symptomatic supportive treatment should be given for AEs related to overdose.

14 Information of Study Drug

14.1 Name and Strength of Therapeutic Drug

Meplazumab for Injection is a new humanized IgG2 monoclonal antibody drug jointly developed by Jiangsu Pacific Meinuoke Bio-pharmaceutical Co., Ltd. and Fourth Military Medical University. Meplazumab for Injection is a recombinant humanized IgG2 antibody expressed in CHO cells (CHO DG44, a mutated Chinese hamster ovary cell). This molecule consists of 2 heavy chains containing 442 amino acids and 2 light chains containing 214 amino

acids linked by disulfide bonds. Each light chain contains 2 intrachain disulfide bonds and each heavy chain contains 4 intrachain disulfide bonds; there are 4 interchain disulfide bonds between heavy chain and heavy chain, 2 interchain disulfide bonds between light chain and heavy chain, and a total of 18 disulfide bonds in the intact protein. Asparagine at position 292 in the Fc region of the heavy chain of this molecule is the only glycosylation site, and the glycosylation modifications are mainly G0F, G1Fa, G1Fb, and G2F glycoforms. The theoretical amino acid molecular weight of the molecule is 144,094 Da, and the theoretical isoelectric point is 7.45. The affinity constant (K_D) of Meplazumab for Injection to CD147 molecule is 1.7 × 10^{-10} M. The molecular formula is $C_{64}0_6H_{9866}N1 6_{92}O_{2009}S_{50}$.

Biological Characteristics

1) Antibody Humanization

We used bioinformatics, recombinant DNA technology and other means to amplify the genes in variable regions of antibody light and heavy chains from hybridoma cell lines expressing anti-CD147 antibody 6H8, and replaced the FR sequences in the variable regions of antibody light and heavy chains with human-derived FR sequences by using bioinformatics method and recombinant technology. The human-derived FR sequences were inserted into expression vectors containing human IgG2 antibody constant region gene respectively, and then corresponding host cells were transfected for expression. The light and heavy chain CDR regions of the expressed antibody molecule were murine, while the FR region and constant region were human-derived. More than 2/3 of the entire molecule was human-derived. The variable region had the function of binding to antigens, while the constant region had the antibody effector function, immunogenicity, and species characteristics. The Fc segment of the chimeric antibody can prolong the half-life of the antibody in serum and theoretically reduce the immunogenicity of heterologous antibodies. This technology retains the complete murine monoclonal antibody variable region sequence, ensuring the affinity and specificity of the antibody. Although theoretically speaking, the constructed chimeric antibody still retains the heterology of murine variable region and may induce a HAMA response, the results of animal studies showed that humanized Meplazumab did not cause abnormal clinical symptoms in the tested animals, and had no significant effect on spontaneous activity, body weight, food consumption, body temperature, blood pressure, ECG, hematological indicators, serum biochemical indicators, urine indicators, CD3+/CD4+, CD3+/CD8+ lymphocytes in the blood,

bone marrow cells, histopathology, injection site, etc.

2) High Affinity

The intensity of antibody-antigen interactions is mainly determined by the affinity between them. These interactions result from non-covalent bonding. Because some amino acid sites in the framework region of parental non-humanized monoclonal antibody may be involved in antigen binding or play an important role in maintaining the conformation of antigen binding region, the affinity of humanized antibody is reduced to a certain extent compared with that of its parental non-humanized monoclonal antibody. However, the affinity of Meplazumab to CD147 extracellular segment molecules was determined by the SPR system. Kinetic-Langmuir model analysis showed that the equilibrium dissociation constant of Meplazumab did not change significantly compared with its parental non-humanized monoclonal antibody 6H8, with an affinity constant (K_D) of 1.7×10^{-10} M, indicating that Meplazumab had a similar affinity compared with its parental mouse monoclonal antibody 6H8 (affinity constant $K_D = 4.48 \times 10^{-10}$ M), ensuring the ability of this monoclonal antibody to bind to target molecules *in vivo* and *in vitro*.

14.2 Drug Product Formulation

Meplazumab will be supplied in lyophilized powder packed in vials, 10 mg/vial, including 1.60 mg of histidine, 3.08 mg of histidine hydrochloride, 50.0 mg of sucrose, 70.0 mg of mannitol and 1.0 mg of polysorbate 80.

The matching control drug is sterile normal saline (0.9%) for IV infusion.

Meplazumab will be provided by the sponsor. The control drug will be provided by the clinical study site.

14.3 Drug Packaging and Label

The production, packaging and labeling of the investigational product will be carried out in accordance with the Good Manufacturing Practice (GMP) and applicable laws and regulations. The investigational product will be appropriately packaged to protect it from contamination during transportation and storage.

The investigational product will be appropriately labeled in accordance with the GCP and GMP, and the label will include instructions that meet regulatory requirements. The drug label will at least include the following contents: clinical trial applicant, name of investigational product,

usage, strength, batch number, protocol number or other unique code used in the clinical trial, drug number, shelf life, storage conditions, and the words "For Clinical Study Only".

14.4 Drug Management

14.4.1 Transfer and Storage

The study drug will be received, handled and stored by a specially-assigned person at the study site. Before administration, the drug will be kept under designated conditions, and can be accessed and checked only by the relevant responsible person of the study, which will be recorded in the CRF. The investigator is responsible for returning all unused therapeutic drugs to the sponsor.

14.4.2 Waste Drug Handling

Discarded study drug (including those with wrong labels, not used by subjects, and refused to be used by subjects) should be collected centrally and disposed of reasonably. All supplies used for reinfusion should be recovered and disposed of, including infusion tubes. The recovered antibody drugs and wastes after infusion should be kept by a specially-assigned person and registered for filing in time. They should be disposed of within one week in accordance with the medical waste disposal standards.

15 Ethics

15.1 Investigator's Responsibility

The investigator is responsible for ensuring that the clinical study is conducted in accordance with the protocol, current ICH-GCP and relevant regulations of the National Medical Products Administration (NMPA) of China.

ICH-GCP is an internationally recognized standard of ethical and scientific quality for designing, conducting, recording, and reporting studies involving human subjects. Studies that adhere to this standard are considered consistent with the principles enunciated in the Declaration of Helsinki in terms of protecting the rights, safety, and interests of subjects and are trustworthy with respect to the quality of study data.

15.2 Independent Ethics Committee (IEC)/Institutional Review Board (IRB)

Before initiation of the study, the investigator must provide the IEC/IRB with the following documents:

Investigator's Brochure, study protocol, CRF, ICF, etc.

The trial cannot begin until the IEC/IRB has given their full approval for the study protocol, ICF, materials to assist in subject enrollment, compensation measures for subjects and the sponsor has received copies of the IEC/IRB approval documents. The approval document must indicate the approved study title (protocol number), study document name (including version number), and approval date.

At the end of the study, the investigator should inform the IEC/IRB that the trial has been completed.

15.3 Informed Consent

The investigator is responsible for explaining the objectives, methods, benefits and potential risks of the clinical trial to each subject. An ICF signed by the subject must be obtained before any procedure related to the clinical trial. ICFs should be provided both orally and in writing. The ICF must be dated and signed by the subject, or by their parents, legal guardians or protectors for those subjects who are unable to sign the ICF by themselves for any reason. A copy of the signed ICF and information sheet should be kept by the subject.

The ICF must be approved by both the sponsor and the IEC/IRB. The ICF should comply with the Declaration of Helsinki, current GCP guidelines, applicable regulations, and sponsor's regulations.

By signing the ICF, the subject/patient must also agree to allow the sponsor, drug approval authority, auditors, and/or monitors to review the original data related to the clinical study, with the reviewer adhering to confidentiality statement.

15.4 Protection of Subject Data

Data from subjects that are essential to study the efficacy, safety, quality, and application of drugs are collected and processed in this study.

The confidentiality of these data will be fully ensured when they are collected and used, and relevant laws and regulations for protecting the privacy of subjects will be followed.

The investigator must take appropriate technical steps and management measures to protect the personal information of subjects from access and disclosure by other unauthorized personnel, accidental and illegal destruction, and accidental loss and alteration. The sponsor who has access to the subject's personal data will keep it confidential throughout the study.

16 Management Requirements

16.1 Protocol Amendment

All protocol amendments must be signed and dated by the sponsor before release. They should not be implemented without IEC/IRB approval, except when necessary to avoid an immediate hazard to the subjects, or when changes are made to only logistical or administrative aspects of the study (e.g. typographical errors, inconsistencies).

16.2 Data Management

16.2.1 Completion and Transfer of Original Data and Case Report Form (CRF)

The Data Department of the CRO will be responsible for the management of the study data to ensure the authenticity, integrity, privacy, and traceability of the clinical trial data.

The data in the eCRFs are all from the original medical records, which are filled in by the investigator or the investigator's designee, and the integrity and accuracy of the information should be ensured. In case of any error that needs to be corrected, the modification should be carried out in accordance with the CRF filling instructions, and the name of the data modifier and the modification date will be automatically recorded in the CRF system.

The completed CRF should be submitted to the CRF system through the network in a timely manner. After SDV, review by DM, query, and so on of the data in the CRF system have been completed without any doubt, the investigator should confirm by electronic signature before the data locking.

16.2.2 Design and Establishment of Database

The database should be established by the Data Department of the CRO, and should meet the requirements of the *Guidelines for Biostatistics in Drug Clinical Trials*. Data traces such as system login, data entry, modification, and deletion in the database should be managed, and the database should be established using the CDISC standard if possible.

16.2.3 Data Entry

Data will be entered into the EDC database by authorized personnel. After data entry is completed, the EDC system will check through programmed study-specific logical checks to ensure the integrity and accuracy of information.

16.2.4 Query Handling

After data are entered and stored in the EDC system, system verification will be started to trigger queries, which need to be reviewed and answered by investigators. The data manager

would assess the investigator's response and close the query if the response was acceptable. The data manager will also manually check the entered data to ensure the logic, consistency and accuracy of the data.

The subject data listings/reports will be generated by programming to support the manual data verification throughout the study. Manual queries can be added in the EDC system when the data requiring the clarification/verification/confirmation by the investigator arises. Before locking, the data manager should confirm that all queries have been resolved.

16.2.5 Data Quality Assurance

The clinical study will be subject to quality assurance audits by the sponsor or a person authorized by the sponsor. GCP audits may also be conducted by drug approval authorities. QA auditor is allowed to review all medical records, study-related documents and correspondence, and ICFs.

16.3 Monitoring

The sponsor will designate a monitor to conduct on-site monitoring. The monitor will be from the sponsor or a CRO authorized by the sponsor, and will operate according to the SOP of the CRO. The monitor should visit regularly from the beginning to the end of the study.

The monitor will have access to the relevant original data of this clinical study and will review the CRFs in accordance with the SOP to determine that the information is complete, accurate, and consistent with the original data.

The CRFs, copies of laboratory data and medical test results must be readily available for inspection by the Clinical Research Associate (CRA), auditors, and health authorities. The monitor should review all CRFs and ICFs.

16.4 Audits and Inspections

In accordance with GCP and the sponsor's audit plan, the sponsor's representative may choose to audit this trial to evaluate the conduct of the trial and compliance with the protocol, GCP and relevant regulatory requirements, and will review the facilities (such as drug storage sites and laboratories) and trial-related records at the site.

Government regulatory authorities may also inspect the facilities of the sponsor and/or the study site. The sponsor will inform the relevant Investigator immediately upon receipt of a notification to inspect the study site. Similarly, the investigator should inform the sponsor of any upcoming inspections.

The investigator must allow representatives of government regulatory authorities and personnel in charge of audit to perform the following activities:

- Inspect facilities of the study site;
- Meet all team members involved in the trial;
- Directly view test data and original documents;
- Consult all other documents related to the study.

Audits and inspections may be conducted at any time during or after the trial to ensure the validity and integrity of the trial data.

16.5 Verification of Original Records

The Investigator should properly handle all data obtained during the clinical study to guarantee the rights and privacy of patients. The investigator should allow the study monitor/auditor/inspector to review and inspect the required clinical study data, to verify the accuracy of the original data and understand the study progress. If the original records cannot be verified, the investigator should assist the monitor/auditor/inspector in further verification of data quality control.

16.6 Study Completion/Termination

End of Study

After the last visit of the last subject in the study is completed, the study site should notify the sponsor, and the study can be considered completed. The sponsor will notify all study sites of the end time of the trial. Continuation of the trial after this time must be agreed upon by the sponsor and may be implemented without supplement to the protocol.

Study Termination

The sponsor reserves the right to terminate the trial at any time. Reasons for the sponsor to terminate the trial or stop the trial at a study center may include, but are not limited to:

(1) The investigator fails to comply with the study protocol or GCP guidelines;

(2) Safety considerations;

(3) There is sufficient evidence suggesting lack of efficacy;

(4) Inadequate subjects recruited by the investigator.

16.7 Confidentiality Agreement and Patient Privacy

The investigator should undertake to keep confidential to a third party any confidential information obtained from the sponsor, or provided or disclosed in connection with the present contractual relationship, and to use such information to the extent agreed herein.

As long as the sponsor has reasonable and justifiable reasons to require the investigator to maintain a confidentiality agreement, this agreement should be independent and valid during the contractual relationship between both parties.

The investigator should ensure the privacy of subjects. In all documents submitted to the sponsor, only the subject identification codes can be used to identify subjects in the study, and their names and hospitalization numbers should not be indicated. The investigator must maintain the name and address of the clinical trial subjects concerned and the enrollment forms corresponding to the clinical study subject codes. These enrollment forms should be kept in strict confidence by the investigator and cannot be submitted to the sponsor.

16.8 Use and Publication of Information

As the sponsors, Jiangsu Pacific Meinuoke Bio-pharmaceutical Co., Ltd. and Fourth Military Medical University have exclusive rights to this study. The authors and the manuscript will reflect collaboration between several investigators as well as collaboration between the sites and sponsor staff. Authors should be identified before the manuscript is written. Because there are many study sites participating in this study, individual articles are not allowed to be published before the final report of the multicenter study is completed, unless agreed by the sponsor. The sponsor has the final decision on the drafts and publications.

17 References

[1]. Diagnosis and Treatment Protocol for COVID-19 Patients (Tentative 10th Version), 2023.
[2]. Technical Guidelines for Clinical Trials of New Antiviral Drugs for COVID-19, 2022.
[3]. Rong Yan, Analysis on Clinical, Imaging and Serum Antibody Characteristics of Mild and Moderate COVID-19, 2021, Southern Medical University.
[4]. Fang Xu, et al. Clinical features and chest CT findings in moderate and severe COVID-19 patients: an analysis of 506 cases from Wuhan Huoshenshan Hospital. Chinese Journal of Clinical Infectious Diseases, 2020. 03(13): p161-166.
[5]. Vanarsdall, A.L., et al., CD147 Promotes Entry of Pentamer-Expressing Human Cytomegalovirus into Epithelial and Endothelial Cells. mBio, 2018. 9(3).
[6]. Pushkarsky, T., et al., CD147 facilitates HIV-1 infection by interacting with virus-associated cyclophilin A. Proc Natl Acad Sci U S A, 2001. 98(11): p. 6360-5.
[7]. Watanabe, A., et al., CD147/EMMPRIN acts as a functional entry receptor for measles virus on epithelial cells. J Virol, 2010. 84(9): p. 4183-93.

[8]. Wang, K., et al., CD147-spike protein is a novel route for SARS-CoV-2 infection to host cells. Signal Transduct Target Ther, 2020. 5(1): p. 283.
[9]. Chen, Z., et al., Function of HAb18G/CD147 in invasion of host cells by severe acute respiratory syndrome coronavirus. J Infect Dis, 2005. 191(5): p. 755-60.
[10]. Bian, H., et al., Safety and efficacy of meplazumab in healthy volunteers and COVID-19 patients: a randomized phase 1 and an exploratory phase 2 trial. Signal Transduct Target Ther, 2021. 6(1): p. 194.
[11]. Geng, J., et al., CD147 antibody specifically and effectively inhibits infection and cytokine storm of SARS-CoV-2 and its variants delta, alpha, beta, and gamma. Signal Transduct Target Ther, 2021. 6(1): p. 347.
[12]. Saphire, A.C., M.D. Bobardt and P.A. Gallay, Human immunodeficiency virus type 1 hijacks host cyclophilin A for its attachment to target cells. Immunol Res, 2000. 21(2-3): p. 211-7.
[13]. Zhinan, C., Use of hab18g/cd147 molecule as target for antiviral antagonists and thus obtained antiviral antagonist. 2007. p. 0.
[14]. Chen Zhinan et al., Application of anti-BASIGIN humanized antibody in the preparation of drugs for treatment of COVID-19, 2020. p18.
[15]. Chen Zhinan, Antagonists of CD147 receptor target for SARS coronavirus and AIDS virus (HIV-1), 2003. p12.
[16]. CHEN, Z.C., Use of hab18g/cd147 molecule as target for antiviral antagonists and thus obtained antiviral antagonist. 2007. p. 0.
[17]. Chen Zhinan et al., Humanized anti-BASIGIN antibodies and the use thereof, 2019. p28.
[18]. CHEN, Z.N.B.C., et al., HAb18G/CD147, ITS ANTAGONIST AND APPLICATION. 2008. p. 0.
[19]. Yurchenko, V., S. Constant and M. Bukrinsky, Dealing with the family: CD147 interactions with cyclophilins. Immunology, 2006. 117(3): p. 301-9.
[20]. Schmidt, R., et al., Extracellular matrix metalloproteinase inducer (CD147) is a novel receptor on platelets, activates platelets, and augments nuclear factor kappaB-dependent inflammation in monocytes. Circ Res, 2008. 102(3): p. 302-9.
[21]. Geng, J.J., et al., Enhancement of CD147 on M1 macrophages induces differentiation of Th17 cells in the lung interstitial fibrosis. Biochim Biophys Acta, 2014. 1842(9): p. 1770-82.
[22]. Wu, J., et al., CD147 contributes to SARS-CoV-2-induced pulmonary fibrosis. Signal Transduct Target Ther, 2022. 7(1): p. 382.
[23]. Zhu, P., et al., CD147 overexpression on synoviocytes in rheumatoid arthritis enhances matrix metalloproteinase production and invasiveness of synoviocytes. Arthritis Res Ther, 2006. 8(2): p. R44.
[24]. Zhu, P., et al., Expression of CD147 on monocytes/macrophages in rheumatoid arthritis: its potential role in monocyte accumulation and matrix metalloproteinase production. Arthritis Res Ther, 2005. 7(5): p. R1023-33.
[25]. Wang, C.H., et al., Expression of CD147 (EMMPRIN) on neutrophils in rheumatoid arthritis enhances chemotaxis, matrix metalloproteinase production and invasiveness of synoviocytes. J Cell Mol Med, 2011. 15(4): p. 850-60.
[26]. Ling L, Wei Z, Yu H, et al. Effect of Convalescent Plasma Therapy on Time to Clinical Improvement in Patients With Severe and Life-threatening COVID-19: A Randomized Clinical Trial, JAMA, 2020. 324(5): p. 460-470.
[27]. Wang Y, Zhang D, Du G, et al. Remdesivir in adults with severe COVID-19: a randomised, double-blind, placebo-controlled, multicentre trial. Lancet, 2020. 395(10236): p. 1569-78.

18 Appendix 1 Ordinal Scale for Clinical Improvement [26-27]

Grade	Clinical Manifestations	Score
1	Died	6
2	Hospitalized, invasive mechanical ventilation, or extracorporeal membrane oxygenation (ECMO)	5
3	Hospitalized, non-invasive mechanical ventilation or high flow oxygen supplementation	4
4	Hospitalized, oxygen supplementation (non-high flow or noninvasive mechanical ventilation)	3
5	Hospitalized, no oxygen supplementation	2
6	Discharged	1

Change Summary of Protocol Modification Description

Table 1 Document History

Document	Date
Original Study Protocol	December 29, 2022
Study Protocol V1.1	January 06, 2023
Study Protocol V1.2	February 13, 2023
Study Protocol V1.3	April 17, 2023
Study Protocol V1.4	July 03, 2023
Study Protocol V1.5	July 18, 2023
Study Protocol V1.6	November 13, 2023

Overall Reason for Modification in Protocol Modification Description 6

The protocol was revised to primarily adjust for intercurrent events and treatment strategies based on the review recommendations from the CDE, as well as to update the statistical analysis methods.

Table 2 Description of Changes in Protocol Modification Description 6

<table>
<tr><th>Section Number and Title</th><th>Description of Change</th><th>Reason for Revision</th></tr>
<tr><td>Cover, Header
Version No. and date</td><td>Updated to:
Version No.: V1.6. Version date: November 13, 2023</td><td>Updated version No. and version date.</td></tr>
<tr><td>2.2.1 Primary Estimand - Table 2 Intercurrent Events of Primary Estimand and Handling Strategies</td><td>Updated intercurrent events:
Updated the following text as follows (new text in bold):
Early discontinuation of treatment due to poor efficacy ~~or adverse events~~
Early discontinuation of treatment due to adverse events</td><td>Modified according to CDE's review comments on the protocol</td></tr>
<tr><td>2.2.2 Secondary Estimands - Table 3 Intercurrent Events</td><td>Updated the following text as follows (new text in bold):<table><tr><th>Intercurrent Events</th><th>Handling Strategy</th><th>Remarks</th></tr></table></td><td>Modified according to CDE's review comments on the</td></tr>
</table>

of Secondary Estimand and Handling Strategies		Early discontinuation of treatment **due to poor efficacy**	**Therapeutic Strategy** Continue to collect and use data despite the intercurrent event	Reflect actual clinical condition		protocol
		**Early discontinuation of treatment due to adverse events**	**Therapeutic Strategy Continue to collect and use data despite the intercurrent event**	**Reflect actual clinical condition**		
		Concomitant use of prohibited medications/treatments affecting efficacy evaluation	**Therapeutic Strategy** Continue to collect and use data despite the intercurrent event	Reflect actual clinical condition		
		Death	~~**Composite variable strategy** (for Secondary Estimands 1, 3, 4, 5, 6, 7, 8, 9, 10, 11)~~<br>**Secondary estimand 1: Composite strategy, handle as "not discharged".**<br>**Secondary estimands 3 and 8: Hypothetical strategy, perform "censoring" according to the time of the last follow-up.**<br>**Secondary estimands 4-7: Therapeutic strategy, handle according to the actual observation days.**<br>**Secondary estimand 9:**			

	**Composite strategy, handle as "no negative conversion".**<br>**Secondary estimand 10: Therapeutic strategy, handle according to the actual observed value.**<br>**Secondary estimand 11: Composite strategy, handle as "not improved".**	
2.2.2 Secondary Estimands - Population-level Summary	Modified Article 8 to (new text in bold):<br>**Hazard ratio** ~~intergroup difference~~ of time to SARS-CoV-2 nucleic acid negative conversion between the test group and control group	Modified the population-level summary of this indicator due to adjustment of statistical analysis methods
12.4.1 Analysis of Primary Estimand	Added the following:<br>Supplementary analysis: Efficacy evaluation will be performed with intercurrent events "early discontinuation of treatment due to poor efficacy", "concomitant use of prohibited medications/treatments affecting efficacy evaluation", and "disease aggravation" analyzed as "death" according to the composite variable strategy.	Added supplementary analysis
12.4.2 Analysis of Secondary Estimands - (3) Time to sustained clinical improvement (days)	Modified the Secondary Estimand 3 "A competitive risk model will be used, with death as a competing risk event. The lower quartile, median and upper quartile of recovery rate and time to sustained clinical improvement at different time points and their two-sided 95% CIs in the Meplazumab group and placebo group, respectively, will be calculated. Also, hazard ratio between the groups (Meplazumab group-placebo group) as well as the two-sided 95% CI will be estimated. Curves will be plotted for the time to sustained clinical improvement in the Meplazumab group and placebo group." to:<br>"Kaplan-Meier method will be used to calculate the improvement rate at different time points	Adjustment of statistical analysis methods

	and the lower quartile, median, and upper quartile of time to sustained clinical improvement and their two-sided 95% CIs (Greenwood method: log-log transformation) in the Meplazumab group and placebo group, respectively, and stratified log-rank test will be used for statistical testing of differences between the groups. Also, the Cox model with the time to sustained clinical improvement as the dependent variable, and group and stratification factor age group (< 65 years old vs. ≥ 65 years old) as the fixed effects will be used to estimate the hazard ratio between the groups (Meplazumab group-placebo group) as well as the two-sided 95% CI. Kaplan-Meier curves will be plotted for the time to sustained clinical improvement in the Meplazumab group and placebo group."	
12.4.2 Analysis of Secondary Estimands - (4) Number of days with oxygen supplementation (days)	Modified Secondary Estimand 4 to (new text in bold):<br>Days of oxygen supplementation (including cumulative days of oxygen supplementation, days of low-flow oxygen supplementation and days of high-flow oxygen supplementation) in the Meplazumab group and placebo group will be statistically described, respectively, **and the difference between the groups (placebo group-Meplazumab group) and its two-sided 95% CI will be calculated.**	Adjustment of statistical analysis methods
12.4.2 Analysis of Secondary Estimands - (5) Number of days with mechanical ventilation (days)	Modified Secondary Estimand 5 to (new text in bold):<br>The days of mechanical ventilation in the Meplazumab treatment group and placebo group will be statistically described, respectively, **and the difference between the groups (placebo group-Meplazumab group) and its two-sided 95% CI will be calculated.**	Adjustment of statistical analysis methods
12.4.2 Analysis of Secondary Estimands - (6) Incidence and duration of re-ventilation 24 hours after withdrawal (days)	Modified Secondary Estimand 6 to (new text in bold):<br>The incidence and duration of the second mechanical ventilation 24 hours after withdrawal of mechanical ventilation in the Meplazumab group and placebo group will be statistically described, respectively, **and the difference between the groups (placebo group-Meplazumab group) and its two-sided 95% CI will be calculated.**	Adjustment of statistical analysis methods
12.4.2 Analysis of Secondary Estimands - (7) Length of	Modified Secondary Estimand 7 to (new text in bold):<br>The ICU time and hospitalization time in the Meplazumab group and placebo group will be	Adjustment of statistical analysis methods

stay in ICU and hospitalization time (days)	statistically described, respectively, **and the difference between the groups (placebo group-Meplazumab group) and its two-sided 95% CI will be calculated.**	
12.4.2 Analysis of Secondary Estimands - (8) Time to negative nucleic acid conversion	Modified the Secondary Estimand 8 "The time to negative nucleic acid conversion in Meplazumab group and placebo group will be statistically described, respectively." to:<br>"Kaplan-Meier method will be used to calculate the lower quartile, median and upper quartile of time to negative conversion as well as their two-sided 95% CIs (Greenwood method: log-log transformation) in the Meplazumab group and placebo group, respectively, and stratified log-rank test will be used for statistical testing of differences between the groups. Also, the Cox model with the time to negative conversion as the dependent variable, and group and stratification factor age group (< 65 years old vs. ≥ 65 years old) as the fixed effects will be used to estimate the hazard ratio between the groups (Meplazumab group-placebo group) as well as the two-sided 95% CI. Kaplan-Meier curves will be plotted for the time to negative conversion in the Meplazumab group and placebo group."	Adjustment of statistical analysis methods
12.4.2 Analysis of Secondary Estimands - (9) SARS-Cov-2 nucleic acid negative conversion rate on D7, D14 and D28	Modified Secondary Estimand 9 to (new text in bold):<br>The SARS-CoV-2 nucleic acid negative conversion rate on D7, D14 and D28 in the Meplazumab group and placebo group will be statistically described, respectively, **and the difference between the groups (placebo group-Meplazumab group) and its two-sided 95% CI will be calculated.**	Adjustment of statistical analysis methods
12.4.2 Analysis of Secondary Estimands - (10) Change from baseline in SARS-CoV-2 viral load on d7, d14 and d28	Modified Secondary Estimand 10 to (new text in bold):<br>The changes from baseline in SARS-CoV-2 viral load **(log 10)** on D7, D14 and D28 in the Meplazumab group and placebo group will be statistically described, respectively, **and the difference between the groups (placebo group-Meplazumab group) and its two-sided 95% CI will be calculated.**	Adjustment of statistical analysis methods
12.4.2 Analysis of Secondary Estimands - (11) Lung imaging improvement rate	Modified Secondary Estimand 11 to:<br>The lung imaging improvement rate on D7 and D14 after the first dose in the Meplazumab group and placebo group will be calculated, respectively, the 95% CI will be calculated by	Adjustment of statistical analysis methods

	the Clopper-Pearson method, **and the difference between the groups (placebo group-Meplazumab group) and its two-sided 95% CI will be calculated.**	
12.10 Multiplicity Handling	Added the following sections and contents:<br>12.10 Multiplicity Handling<br>There is only one primary endpoint for this trial; therefore, type I error correction is not required.	Added a section - Multiplicity Handling
12.11 Handling of Missing Data	Added the following sections and contents:<br>12.11 Handling of Missing Data<br>In this study, missing data for the primary efficacy endpoint on D28 after administration will be imputed according to the "status of the last follow-up". The handling of missing data for secondary efficacy endpoints will be detailed in the SAP.	Added a section - Handling of Missing Data
12.12 Determination of Sample Size	Changed to Section 12.12 and modified as follows:<br>Modified "2-sided test α is 0.05, and the power is set at 0.8. It is calculated using the nQuery (Version 9.1)" to "The one-sided test α is 0.025, and the power is set at 0.8. It is calculated using the "Tests for Two Proportions" module in PASS 21".	Modification of sample size calculation software

Overall Reason for Modification in Protocol Modification Description 5

The protocol was revised to primarily adjust for intercurrent events and subgroup analyses based on the review recommendations from the CDE, add missing information, and correct errors and inconsistencies.

Table 3 Description of Changes in Protocol Modification Description 5

Section Number and Title	Description of Change	Reason for Revision
Cover, Header<br>Version No. and date	Updated to:<br>Version No.: V1.5. Version date: July 18, 2023	Updated version No. and version date.
Synopsis: Secondary	Updated the following text as follows (new text in bold):	Error correction: Unification

Objectives, 2.1 Study Objectives and Endpoints	Time to Sustained ~~Recovery~~ **Clinical Improvement**	of the title of clinical improvement in the full text.
Synopsis: Analysis of Primary Estimand, 12.9 Subgroup Analysis	Updated the following text as follows (new text in bold):<br>Subgroup analysis can be performed by age (<65 years or ≥65 years), gender and whether or not **(M or F)**, concomitant use of antiviral drugs **(Yes or No)**, **SARS-CoV-2 vaccination status (Yes or No), pre-existing disease (Yes or No), BMI ≥ 30 (kg/m^2) (Yes or No) and smoking (Yes or No)**	Modified according to CDE's modification suggestions on study protocol
Flowchart Description "m"	Updated the following text as follows (new text in bold):<br>**If a subject is tested negative for nucleic acid before the first dose, no more nucleic acid testing is required during the study. For those who are still positive for nucleic acid before the first dose, nucleic acid testing will be performed during the study.** If the ~~first~~ nucleic acid test result is negative, a re-test is required at least 24 hours later.	Note here as enrollment of patients with negative baseline nucleic acid test was allowed
2.2.1 Primary Estimand: Table 2 Intercurrent Events of Primary Estimand and Handling Strategies	Modified contents of Table 2:<br>"Early discontinuation of treatment" for intercurrent events was revised to "Early discontinuation of treatment due to poor efficacy or adverse events";<br>Modified intercurrent event "death" and handling strategy "**composite variable strategy**" to "disease aggravation (progression to critical illness)" and "**Therapeutic Strategy** Continue to collect and use data despite the intercurrent event"	Modified according to CDE's modification suggestions on study protocol
2.2.2 Secondary estimands	Added the following under Population:<br>the target population of estimands 8–10 is severe SARS-CoV-2 infected patients with positive baseline nucleic acid testing.	Since the baseline nucleic acid testing of enrolled patients may be negative, the target population for modified variables 8 – 10 is only severe SARS-CoV-2 infected patients with positive baseline nucleic acid testing.

2.2.2 Secondary estimands	Modified and updated Secondary Estimand 8 "Negative conversion rate of SARS-CoV-2 nucleic acid test on D7, D14, and D28" to:<br>8） Time to SARS-CoV-2 nucleic acid negative conversion of subjects;<br>9） Whether the subject's SARS-CoV-2 nucleic acid test result converts to negative on d7, d14 and d28;<br>10） Change from baseline in SARS-CoV-2 viral load on d7, d14 and d28;	Concurrent revision of secondary estimand variables based on revised secondary objectives and endpoints
2.2.2 Secondary estimands	Handling strategies for the intercurrent event "death" in Table 3:<br>Deleted the text "Therapeutic strategy (applies to secondary estimands 1, 4, 5, 6, 7, 8, 9)" and "Treatment strategy (applies to estimand 3) Death is treated as a competing risk event for events with sustained resolution";<br>In the composite variable strategy, (applicable to secondary estimands 2) was changed to (applicable to secondary estimands 1, 3, 4, 5, 6, 7, 8, 9, 10, 11)	Modified according to CDE's modification suggestions on study protocol
9.1 Efficacy Evaluation	Definition of nucleic acid negative conversion added: for patients with positive baseline SARS-CoV-2 nucleic acid testing	Modified the definition of nucleic acid conversion as enrollment of patients with negative baseline nucleic acid test was allowed
12.10 Determination of Sample Size	Changed 175 subjects to 176 subjects	Corrected the errors
Modified format in the full text	NA	Unified format

Overall Reason for Modification in Protocol Modification Description 4

The protocol was revised primarily to revise the inclusion criteria, add missing information, and correct errors and inconsistencies.

Table 4 Description of Changes in Protocol Modification Description 4

Section Number and Title	Description of Change	Reason for Revision
Cover, Header<br>Version No. and date	Updated to:<br>Version No.: V1.4. Version date: July 3, 2023	Updated version No. and version date.
Synopsis, 4.1 Inclusion Criteria (1)	Updated the following text as follows (new text in bold):<br>Males or females aged ~~18–84 (both inclusive)~~ **≥ 18**;	Available clinical data showed that Meplazumab had good safety in subjects aged ≥ 85 years, so the age range of subjects was expanded
Synopsis, 4.1 Inclusion Criteria (2)	Delete "Subjects diagnosed with SARS-CoV-2 infection in the laboratory by PCR nucleic acid amplification test (within 72 h before the first dose)"	Meplazumab has antiviral and anti-inflammatory effects. For patients with nucleic acid negative conversion, this drug can exert a therapeutic effect by inhibiting inflammatory storms, so subjects who have turned negative can be included.
Synopsis, 4.2 Exclusion Criteria (2)	The following text was updated as follows (new text in bold):<br>"Paxlovid (Nirmatrelvir Tablets/Ritonavir Tablets [co-packaged], $t_{1/2}$: approx. ~~18 h~~ **6.05 h**)"	Corrected the writing error. The $t_{1/2}$ of Paxlovid (Nirmatrelvir Tablets/Ritonavir Tablets [co-packaged]) should be 6.05 h.
Flow Chart	Delete "SARS-CoV-2 result review; positive confirmation"	Meplazumab has antiviral and anti-inflammatory effects. For patients with nucleic acid negative conversion, this drug can exert a therapeutic effect by inhibiting inflammatory storms, so subjects who have turned negative can be included.

Overall Reason for Modification in Protocol Modification Description 3

The protocol was revised primarily to modify the study objectives and endpoints according to the review suggestions from CDE, so as to statistically increase the sample size by 2 cases and refine the drugs that may have an impact on the investigational drug in the exclusion criteria.

Table 5 Description of Changes in Protocol Modification Description 3

Section Number and	Description of Change	Reason for

Title		Revision
Cover, Header Version No. and date	Updated to:<br>Version No.: V1.3. Version date: April 17, 2023	Updated version No. and version date.
Synopsis, 2 Study Objectives, Endpoints, and Estimands	Delete "To evaluate the safety and efficacy of Meplazumab for Injection when added to the standard of care, compared to the standard of care alone, in patients with severe SARS-CoV-2 infection." Updated to:<br>Primary objectives:<br>• To validate that Meplazumab is superior to placebo when added to the standard of care in reducing all-cause mortality on D28 in patients with severe SARS-CoV-2 infection;<br>Secondary objectives:<br>• To evaluate the efficacy of Meplazumab compared to placebo when added to the standard of care in increasing the discharge rate, reducing mortality, shortening the time to sustained recovery, reducing the number of days with oxygen supplementation, mechanical ventilation, length of intensive care unit (ICU) stay and hospital stay, shortening the time to SARS-CoV-2 nucleic acid negative conversion, increasing the SARS-CoV-2 nucleic acid negative conversion rate, reducing SARS-CoV-2 viral load and improving the proportion of subjects with improvement in lung imaging in patients with severe SARS-CoV-2 infection;<br>• To evaluate the changes from baseline in levels of cytokines and chemokines associated with inflammatory and immune states as well as lymphocyte subsets in patients with severe SARS-CoV-2 infection after dosing with Meplazumab;<br>• To evaluate the safety of Meplazumab in patients with severe SARS-CoV-2 infection;<br>• To evaluate the immunogenicity of Meplazumab in patients with severe SARS-CoV-2 infection.<br>According to the requirements of estimands, the study objectives were refined and corresponded to the study endpoints one by one. Section 2.1 Study Objectives and Endpoints was added, and "Table 1 Study Objectives and Endpoints" was added:<br>Added Section 2.2 Estimands, including the definition and description of estimands.	Modified according to CDE reply
Synopsis, 4.2 Exclusion Criteria (2)	Added Exclusion Criteria 2:<br>Subjects who use anti-SARS-CoV-2 drugs within 3 half-lives prior to the first dose or during the study, such as	Modified according to CDE reply

	Paxlovid (Nirmatrelvir Tablets/Ritonavir Tablets [co-packaged], t1/2: approx. 18 h), Azvudine Tablets (t1/2: approx. 9 h), Molnupiravir Capsules (t1/2: approx. 3.3 h), Amubarvimab/Romlusevimab Injection (t1/2: approx. 45 d/75 d), human COVID-19 immunoglobulins (t1/2: approx. 3–4 w) or convalescent plasma (t1/2: approx. 21 d), Deuremidevir Hydrobromide Tablets (t1/2: approx. 4.80–-6.95 h), Simnotrelvir Tablets/Ritonavir Tablets (co-packaged) (t1/2: approx. 4.14 h), **Baricitinib** (t1/2: approx. 12.5 h), **Tocilizumab** (t1/2: approx. 21.5 d), **Remdesivir** (t1/2 approx. 27 h), Leritrelvir (t1/2: approx. 14.9 h), Favipiravir (t1/2: approx. 4.5 h) and 2-deoxy-D-glucose (t1/2: approx. 50 min);	
Synopsis: Secondary indicators/endpoints for efficacy evaluation	Updated the following text as follows (new text in bold):<br>(4) Time to sustained clinical improvement (days; range: D0–D28), defined as **patient discharge (discharge criteria: body temperature returning to normal for at least 3 days, significant improvement in respiratory symptoms without oxygen support, and two consecutive negative nucleic acid test results at least 24 hours apart) or a reduction of at least 2 points on the Ordinal Scale for Clinical Improvement (see Appendix 1)** ~~to achieve one of the following: 1. Discharge; 2. Hospitalization. No need for oxygen inhalation~~<br>(9) **Time to SARS-CoV-2 nucleic acid negative conversion**<br>**(10)** SARS-CoV-2 nucleic acid negative conversion rate and **SARS-CoV-2 viral load** on D7, D14 and D28	Modified according to CDE reply
Synopsis; Study Evaluation	Deleted secondary efficacy endpoints (11) and (12), added new pharmacodynamic evaluation indicator (1) Changes from D0 (pre-dose) in the levels of cytokines and chemokines associated with inflammatory and immune states on D1, D4, D7 (before the second dose), including CRP, IL-2, IL-4, IL-6, INF-α, IL-8, IL-10, IL-12p70, IL-17, IL-1β, TNF-α and IFN-γ; (2) Changes from D0 (pre-dose) in lymphocyte subsets CD3+, CD4+ and CD8+ on D1, D4, D7 (before the second dose), D8, D10 and D28.<br>"Safety Evaluation" was revised to "Evaluation of Safety and Immunogenicity", and the relevant description was added: "Blood samples will be collected pre-dose and post-dose (on D28 and D56) and tested for anti-drug antibodies (ADAs) to evaluate immunogenicity."	According to the format framework of "Estimands", deleted repeated descriptions and added/detailed evaluation contents
Flow Chart	Added the requirement that investigators should assess subjects' clinical status according to "Ordinal Scale for Clinical Improvement" on a daily basis	Modified according to CDE reply

<table>
<tr><td>Flow Chart</td><td>"On d2, d4, d7 (before the second dose), d9, and thereafter every 48 h until discharge [m]" of nasopharyngeal swab collection for nucleic acid testing was changed to "Nucleic acid testing is performed daily until discharge [m]".</td><td>Modified according to CDE reply</td></tr>
<tr><td>2.1 Study Objectives and Endpoints</td><td>Section 2.1 Study Objectives and Endpoints was added as follows:
Table 1 Study Objectives and Endpoints
<table>
<tr><th>Primary Objectives</th><th>Primary Endpoints</th></tr>
<tr><td>To validate that Meplazumab is superior to placebo when added to standard of care in reducing all-cause mortality on D28 in patients with severe SARS-CoV-2 infection</td><td>• All-cause mortality at D28</td></tr>
<tr><th>Secondary Objectives - Efficacy</th><th>Secondary Efficacy Endpoints</th></tr>
<tr><td>To evaluate the efficacy of Meplazumab compared to placebo when added to the standard of care in increasing the discharge rate, reducing mortality, shortening the time to sustained clinical improvement, reducing the number of days with oxygen supplementation, mechanical ventilation, length of intensive care unit (ICU) stay and hospital stay, shortening the time to SARS-CoV-2 nucleic acid negative conversion, increasing the SARS-CoV-2 nucleic acid negative conversion rate, reducing SARS-CoV-2 viral load and improving the proportion of subjects with improvement in lung imaging in patients with severe SARS-CoV-2 infection</td><td>• Discharge rate on D28
• Mortality on D14 and D56
• Time to sustained clinical improvement (days, range: D0–D28)
• Duration of oxygen supplementation (days)
• Duration of mechanical ventilation (days)
• Incidence and duration of re-ventilation 24 hours after withdrawal (days)
• Length of stay in ICU and hospitalization time (days)
• Time to SARS-CoV-2 nucleic acid negative conversion
• SARS-CoV-2 nucleic acid negative conversion rate and SARS-CoV-2 viral load on D7, D14 and D28
• Proportion of subjects with improvement in lung imaging</td></tr>
<tr><th>Secondary Objectives - Pharmacodynamics</th><th>PD Evaluation</th></tr>
</table>
</td><td>Modified according to CDE reply</td></tr>
</table>

<table>
<tr><td></td><td>
<table>
<tr><td>To evaluate the changes from baseline in levels of cytokines and chemokines associated with inflammatory and immune states as well as lymphocyte subsets in patients with severe SARS-CoV-2 infection after dosing with Meplazumab</td><td>• Changes from D0 (pre-dose) in the levels of cytokines and chemokines associated with inflammatory and immune states on D1, D4, D7 (before the second dose), D8, D10 and D28, including CRP, IL-2, IL-4, IL-6, INF-α, IL-8, IL-10, IL-12p70, IL-17, IL-1β, TNF-α and IFN-γ
• Changes from D0 (pre-dose) in lymphocyte subsets CD3+, CD4+ and CD8+ on D1, D4, D7 (before the second dose), D8, D10 and D28</td></tr>
<tr><td>Secondary Objectives - Safety</td><td>Safety Endpoints</td></tr>
<tr><td>To evaluate the safety of Meplazumab in patients with severe SARS-CoV-2 infection</td><td>• Incidence of adverse events (AEs) and serious adverse events (SAEs);
• Vital signs (blood pressure, pulse, blood oxygen saturation SpO2 or oxygenation index, respiration, and body temperature);
• Laboratory tests (hematology, urinalysis, blood chemistry and coagulation);
• 12-lead ECG;
• Chest imaging.</td></tr>
<tr><td>Secondary Objectives - Immunogenicity</td><td>Immunogenicity Evaluation</td></tr>
<tr><td>To evaluate the immunogenicity of Meplazumab in patients with severe SARS-CoV-2 infection</td><td>• Anti-drug antibody</td></tr>
</table>
</td><td></td></tr>
<tr><td>2.2 Estimands</td><td>Added Section 2.2 Estimands as follows:
2.2.1 Primary estimands
The primary estimand of this trial is the difference between the test group and control group in the all-cause</td><td>Modified according to CDE reply</td></tr>
</table>

mortality on D28 after treatment in patients with severe SARS-CoV-2 infection. Specifically:

Population: The target population of this trial is patients with severe SARS-CoV-2 infection. Refer to Section 4 "Study Population" for specific inclusion requirements.

Variable: Whether the subject dies on D28;

Treatment: Subjects in the test group will receive Meplazumab and standard of care (SoC), while subjects in the control group will receive placebo and SoC. Refer to Section 6 "Dosage and Method of Administration" for the specific treatment regimen.

Intercurrent events and handling strategies: The main intercurrent events and handling strategies in the study are defined as follows:

Table 2 Intercurrent Events of Primary Estimands and Handling Strategies

Intercurrent Events	Handling Strategy	Remarks
Early discontinuation of treatment	**Therapeutic Strategy**<br>Continue to collect and use data despite the intercurrent event	Reflect actual clinical condition
Concomitant use of prohibited medications/treatments affecting efficacy evaluation	**Therapeutic Strategy**<br>Continue to collect and use data despite the intercurrent event	Reflect actual clinical condition
Death	**Composite variable strategy**	

Population-level Summary: Difference between the test group and control group in all-cause mortality on D28.

2.2.2 Secondary Estimands

Population: The target population of this trial is patients with severe SARS-CoV-2 infection. Refer to Section 4 "Study Population" for specific inclusion requirements. The target population of estimand 6 is severe SARS-CoV-2 infected patients with mechanical ventilation withdrawal during the trial.

Variable:

1） Whether the subject is discharged on D28;

2） Whether the subject dies on D14 and D56;

3） Time to sustained clinical improvement in subjects (days; range: D0–D28); sustained clinical improvement is defined as patient discharge (discharge definition[26]: body temperature returning to normal for at least 3 days, significant improvement in respiratory symptoms without oxygen support, and two consecutive negative nucleic acid test results at least 24 hours apart) or a reduction of at least 2 points on the Ordinal Scale for Clinical Improvement (see Appendix 1);

4） Duration of oxygen supplementation for the subject (days);

5） Duration of mechanical ventilation for the subject (days);

6） Whether mechanical ventilation is provided again 24 hours after withdrawal of mechanical ventilation for the subject, and the duration of the second mechanical ventilation (days);

7） length of stay in ICU (days) and hospitalization time of the subject (days);

8） Whether the subject's SARS-CoV-2 nucleic acid test result converts to negative on d7, d14 and d28;

9） Whether the subject's lung imaging is improved; Improvement in lung imaging is defined as any of the following changes from baseline in lung imaging during the study: 1) reduction in lesion diameter; 2) decrease in the number of lesions; 3) decrease in the number of lung lobes involved by lesions.

Treatment: Subjects in the test group will receive Meplazumab and standard of care (SoC), while subjects in the control group will receive placebo and SoC. Refer to Section 6 "Dosage and Method of Administration" for the specific treatment regimen.

Intercurrent events and handling strategies: The main intercurrent events and handling strategies in the study are defined as follows:

Table 3 Intercurrent Events of Secondary Estimand and Handling Strategies

Intercurrent Events	Handling Strategy	Remarks
Early discontinuation of treatment	**Therapeutic Strategy**<br>Continue to collect and use data despite the intercurrent event	Reflect actual clinical condition
Concomitant use of	**Therapeutic Strategy**	Reflect actual clinical

prohibited medications/treatments affecting efficacy evaluation	Continue to collect and use data despite the intercurrent event	condition
Death	**Therapeutic strategy** (for Secondary Estimands 1, 4, 5, 6, 7, 8, 9)<br>**Composite variable strategy (applicable to secondary estimands 2) Treatment strategy (applicable to estimands 3)**<br>Death is treated as a competing risk event for events with sustained resolution	

Population-level Summary:

1） Difference between the test group and control group in discharge rate on D28;

2） Difference between the test group and control group in mortality on D14 and D56;

3） Hazard ratio of time to sustained clinical improvement between the test group and control group;

4） Difference between the test group and control group in days of oxygen supplementation;

5） Difference between the test group and control group in days of mechanical ventilation;

6） Difference between the test group and control group in the incidence of the second mechanical ventilation 24 hours after withdrawal of mechanical ventilation and the duration of the second mechanical ventilation;

7） Difference between the test group and control group in ICU days and hospitalization days;

8） Difference of time to SARS-CoV-2 nucleic acid negative conversion between the test group and control group;

9） Difference between the test group and control group in SARS-CoV-2 nucleic acid negative conversion rate on D7, D14, and D28;

10） Difference between the test group and control group in SARS-CoV-2 viral load on D7, D14, and D28;

11） Difference between the test group and control group in the proportion of subjects with improvement in lung

	imaging.	
12 Statistical Analysis	**Section 12.1** was revised to "Statistical and Analytical Plan" with the content revised as "The primary estimands will be based on the Full Analysis Set (FAS) for the primary analysis. FAS includes all randomized subjects who receive at least one dose of the investigational product, as per the intent-to-treat (ITT) principle. In addition, it will be analyzed based on the Per-Protocol Set (PPS), which will exclude subjects who do not complete treatment according to the protocol or have major protocol violations from the FAS. Secondary estimands will be analyzed based on the FAS and PPS. Safety analysis will be conducted based on the Safety Set (SS), which includes all randomized subjects who receive at least one dose of the investigational product. All the above analysis sets will be jointly discussed and decided by the sponsor, principal investigator, statistician, and data manager during the data blinding review meeting prior to database lock."<br>**Section 12.2** was revised as "Hypothesis Testing" and the content was revised to "Hypothesis testing will be performed on all-cause mortality on D28 postdose in subjects with severe SARS-CoV-2 infection, *i.e.*: Null hypothesis $H_0:\pi_C \leq \pi_T$ and alternative hypothesis $H_1:\pi_C > \pi_T$, where π_T,π_C represent the all-cause mortality in the Meplazumab group and placebo group, respectively, with one-sided α=0.025 used for the test level."<br>**Section 12.4** was modified to "Efficacy Analyses" and the content was modified to:<br>"12.4.1 Analysis of Primary Estimand<br>For the Meplazumab and placebo groups, the all-cause mortality on D28 post-dose will be calculated. The Clopper-Pearson method will be used to calculate its two-sided 95% confidence interval (CI). CMH Chi-square test will be used for the statistical test of inter-group difference, with age (< 65 years or ≥ 65 years) as the stratification factor. Meanwhile, the difference in the all-cause mortality on D28 post-dose (placebo group-Meplazumab group) and its two-sided 95% CI will be calculated using the CMH method, with age (< 65 years or ≥65 years) as the stratification factor. Also, Miettinen & Nurminen's method without considering stratification factors will be used to calculate the difference between the groups (placebo group-Meplazumab group) and its two-sided 95% confidence interval (CI). The chi-square test/Fisher's exact test without considering stratification factors will be used for statistical testing of the difference between the two groups. Logistic regression model will be fitted to calculate the odds ratio (OR) between the Meplazumab group and the placebo	Modified according to CDE's reply, and carried out statistical analysis according to the latest requirements of estimands

	group as well as the 95% CI. The model will include all-cause mortality on D28 postdose as the dependent variable and group and age group (< 65 years vs ≥ 65 years) as fixed effects. Also, TPA (tipping point analysis) will be used to perform sensitivity analysis on missing data and evaluate the impact of missing data on the robustness of test results.<br>12.4.2 Analysis of Secondary Estimands<br>(1) Discharge rate on d28 after the first dose<br>The discharge rate on D28 after the first dose will be calculated for the Meplazumab group and placebo group, respectively. The two-sided 95% CI will be calculated using the Clopper-Pearson method. The difference in discharge rate on D28 postdose (placebo group-Meplazumab group) and the two-sided 95% CI will be calculated using the CMH method considering stratification factor (age group (< 65 years vs. ≥ 65 years)).<br>(2) All-cause mortality on d14 and d56 after the first dose<br>The all-cause mortality on D14 and D56 after the first dose will be calculated for the Meplazumab group and placebo group, respectively. The two-sided 95% CI will be calculated using the Clopper-Pearson method. The difference in all-cause mortality on D14 and D56 postdose (placebo group-Meplazumab group) and the two-sided 95% CI will be calculated using the CMH method considering stratification factor (age group (< 65 years vs. ≥ 65 years)).<br>(3) Time to sustained clinical improvement (days)<br>"A competitive risk model will be used, with death as a competing risk event. The lower quartile, median and upper quartile of recovery rate and time to sustained clinical improvement at different time points and their two-sided 95% CIs in the Meplazumab group and placebo group, respectively, will be calculated. Also, hazard ratio between the groups (Meplazumab group-placebo group) as well as the two-sided 95% CI will be estimated. Kaplan-Meier curves will be plotted for the time to sustained clinical improvement in the Meplazumab group and placebo group.<br>(4) Duration of oxygen supplementation (days)<br>Days of oxygen supplementation (including cumulative days of oxygen supplementation, days of low-flow oxygen supplementation and days of high-flow oxygen supplementation) in the Meplazumab group and placebo	

	group will be statistically described.<br>(5) Number of days with mechanical ventilation (days)<br>The days of mechanical ventilation in the Meplazumab treatment group and placebo group will be statistically described, respectively.<br>(6) Incidence and duration of re-ventilation 24 hours after withdrawal (days)<br>The incidence and duration of the second mechanical ventilation 24 hours after withdrawal of mechanical ventilation in the Meplazumab group and placebo group will be statistically described, respectively.<br>(7) Length of stay in ICU and hospitalization time (days)<br>The ICU time and hospitalization time in the Meplazumab group and placebo group will be statistically described, respectively.<br>(8) Time to negative nucleic acid conversion<br>The time to negative nucleic acid conversion in Meplazumab group and placebo group will be statistically described, respectively.<br>(9) SARS-CoV-2 nucleic acid negative conversion rate on D7, D14, and D28<br>The SARS-CoV-2 nucleic acid negative conversion rate on D7, D14 and D28 in the Meplazumab group and placebo group will be statistically described, respectively.<br>(10) SARS-CoV-2 viral load to D7, D14 and D28<br>The SARS-CoV-2 viral load on D7, D14 and D28 in the Meplazumab group and placebo group will be statistically described, respectively.<br>(11) Lung imaging improvement rate<br>The lung imaging improvement rate on D7 and D14 after the first dose in the Meplazumab group and placebo group will be calculated, respectively, the 95% CIs will be calculated by the Clopper-Pearson method."<br>**Section 12.5** was modified to "Pharmacodynamic Analysis" and the content was modified to:<br>"(1) Changes from baseline in cytokines and chemokines<br>Cytokines and chemokines on D1, D4, D7 (before the second dose), D8, D10 and D28 after the first dose and their changes from baseline in the Meplazumab group and placebo group will be statistically described,	

	respectively.<br>(2) Change from baseline in lymphocyte subsets<br>Lymphocyte subsets on D1, D4, D7 (before the second dose), D8, D10 and D28 after the first dose and their changes from baseline in the Meplazumab group and placebo group will be statistically described, respectively.<br>**Section 12.6** was modified to "Immunogenicity Analysis" and the content was changed to:<br>"The positive rate and cumulative positive rate of anti-drug antibody (ADA) in the Meplazumab group and placebo group at each time point before and after treatment will be calculated, respectively, and the two-sided 95% CI will be calculated by the Clopper-Pearson method."<br>**Section 12.7** was modified to "Safety Analysis" and the content was changed to:<br>"12.7.1 Adverse events<br>AEs will be coded as per MedDRA (26.0 or the latest version). Classified statistics will be performed by SOC and PT. In this trial, the treatment-emergent adverse events (TEAEs) will mainly be statistically analyzed; pretreatment AEs will be presented in a list. Unless otherwise specified, adverse events below are TEAEs.<br>The number of events, number of subjects and incidence of the following AEs will be calculated for subjects in the Meplazumab group and placebo group, respectively:<br>All TEAEs;<br>Treatment-related AEs (TRAEs);<br>AEs of different severity;<br>TRAEs of different severity;<br>AEs with Incidence ≥ 1% in Any Group;<br>TRAEs with incidence ≥ 1% in any group;<br>AEs with Incidence ≥ 5% in Any Group;<br>TRAEs with incidence ≥ 5% in any group;<br>Serious Adverse Events (SAEs);<br>Treatment-related SAEs;<br>AEs leading to withdrawal from the trial;	

TRAEs leading to withdrawal from the trial;
AEs leading to death;
TRAEs leading to death;
AEs leading to discontinuation;
TRAEs leading to discontinuation;
AEs leading to dose reduction;
TRAEs leading to dose reduction;
AEs leading to dose interruption;
TRAEs leading to dose interruption.

The severity and correlation of adverse events will be tabulated.

When calculating the incidence of AEs in each group, count multiple occurrences of the same adverse event in a single subject as one event. When calculating the number of cases, count multiple occurrences of the same adverse event in a single subject as multiple events. For the analysis of the severity and drug correlation of adverse events, if a subject experiences the same adverse event multiple times, the most severe occurrence or the one most related to the drug should be used for analysis.

AEs will be listed separately.

12.7.2 Laboratory tests

Baseline values of laboratory test indicators (including hematology, blood chemistry, urinalysis, and coagulation function) and changes from baseline to the worst grade will be described in a shift table (based on the normal range and investigator's judgment of clinical significance) by Meplazumab group and placebo group.

The test results and abnormalities of all laboratory tests during the study will be listed.

12.7.3 Physical examination

Baseline values of physical examination indicators and changes from baseline to the worst grade will be described in a shift table (based on the normal range and investigator's judgment of clinical significance) by Meplazumab group and placebo group.

All physical examination results and abnormalities during the study will be listed.

	12.7.4 Vital signs<br>The vital signs in the Meplazumab group and placebo group will be statistically described, respectively.<br>Baseline values of vital signs examination indicators and changes from baseline to the worst grade will be described in a shift table (based on the normal range and investigator's judgment of clinical significance) by Meplazumab group and placebo group.<br>The test results and abnormalities of vital signs during the study will be listed.<br>12.7.5 12-Lead ECG<br>Baseline values of 12-ECG indicators and changes from baseline to the worst grade will be described in a shift table (based on the normal range and investigator's judgment of clinical significance) by the Meplazumab group and placebo group.<br>The test results and abnormalities of all 12-lead ECG examinations during the study will be listed.<br>**Section 12.8** "Interim Analysis" was added and the content was revised to: "Interim analysis will not be performed in this study."<br>**Section 12.9** "Subgroup Analysis" was added and the text was revised to: "If supported by data, subgroup analysis of the primary efficacy endpoint by age (< 65 years or ≥ 65 years), sex, and concomitant use of antiviral drugs will be performed, with a forest plot plotted."	
12.10 Determination of Sample Size	Section 12.7 was updated to 12.10 and the following text was updated as follows (new text in bold):<br>The possibility of combining the **mortality rates in the 3 dose groups in previous clinical studies** and considering the variability inherent in the data<br>Total: ~~350~~**352** subjects	Modified the description, and increased the sample size by 2 cases considering the convenience of later statistical operation
8. Concomitant Therapy: 8.2	Added the following contents:<br>The investigators should continue to provide standardized treatment for subjects in accordance with the	Exclusion of drugs that may have an

<table>
<tr><td>Contraindicated Medications for Subjects During the Study</td><td>requirements of the Diagnosis and Treatment Protocol for COVID-19 Patients (Tentative 10th Version) issued by the National Health Commission during the study. If serious adverse reactions occur during treatment, they will be handled according to relevant clinical guidelines.
Contraindicated medications for subjects during the study: anti-SARS-CoV-2 drugs (such as Paxlovid (Nematvir Tablets/Ritonavir Tablets co-package), Azvudine Tablets, Molnupiravir Capsules, Amubarvimab/Romlusevimab Injection, COVID-19 human immunoglobulin or convalescent patient plasma, Deuremidevir Hydrobromide Tablets, Simnotrelvir Tablets/Ritonavir Tablets (Co-packaged), baricitinib, tocilizumab, remdesivir, leritrelvir, favipiravir, and 2-deoxy-D-glucose).</td><td>impact on the efficacy or safety of the investigational drug</td></tr>
<tr><td>9.1 Efficacy Evaluation</td><td>Updated the following text as follows (new text in bold):
Definition of time to sustained clinical improvement in the study: The patient is discharged (discharge definition[26]: body temperature returns to normal for at least 3 days; significant improvement in respiratory symptoms without oxygen support; two consecutive negative PCR tests at least 24 hours apart) or a reduction of at least 2 points on the Ordinal Scale for Clinical Improvement (see Appendix 1). <s>Achieve one of the following: 1. Discharge; 2. Hospitalization. No need for oxygen inhalation.</s></td><td>Modified according to CDE reply</td></tr>
<tr><td>17 References</td><td>Added the following references:
[26] Ling L , Wei Z , Yu H, et al. Effect of Convalescent Plasma Therapy on Time to Clinical Improvement in Patients With Severe and Life-threatening COVID-19: A Randomized Clinical Trial， JAMA, 2020. 324(5): p. 460-470.
[27]. Wang Y, Zhang D, Du G, et al. Remdesivir in adults with severe COVID-19: a randomised, double-blind, placebo-controlled, multicentre trial. Lancet, 2020. 395(10236): p. 1569-78.</td><td>References were added</td></tr>
<tr><td>Appendix 1</td><td>ADD CONTENTS:
APPENDIX 1 ORDINAL SCALE FOR CLINICAL IMPROVEMENT [26-27]
<table><tr><th>GRADE</th><th>CLINICAL MANIFESTATIONS</th><th>SCORE</th></tr><tr><td>1</td><td>DEATH</td><td>6</td></tr></table></td><td>Modified according to CDE reply</td></tr>
</table>

		2	HOSPITALIZED, INVASIVE MECHANICAL VENTILATION, OR EXTRACORPOREAL MEMBRANE OXYGENATION (ECMO)	5		
		3	HOSPITALIZED, NON-INVASIVE MECHANICAL VENTILATION OR HIGH FLOW OXYGEN SUPPLEMENTATION	4		
		4	HOSPITALIZED, OXYGEN SUPPLEMENTATION (NON-HIGH FLOW OR NONINVASIVE MECHANICAL VENTILATION)	3		
		5	HOSPITALIZED, NO OXYGEN SUPPLEMENTATION	2		
		6	DISCHARGE	1		
Modified format in the full text	NA					Unified format

Overall Reason for Modification in Protocol Modification Description 2

The protocol was revised primarily to modify the inclusion and exclusion criteria according to the diagnosis and treatment guidelines and clinical practice, and some modifications were explained to facilitate clinical implementation.

Table 6 Description of Changes in Protocol Modification Description 2

Section Number and Title	Description of Change	Reason for Revision
Cover, Header<br>Version No. and date	Updated to:<br>Version No.: V1.2. Version date: February 13, 2023	Updated version No. and version date.
Synopsis, 4.1<br>Inclusion Criteria<br>(1)	(1) Males or females aged 18~~–80~~84 (both inclusive)	According to the newly issued *Diagnosis and Treatment Protocol for COVID-19 Patients* (Tentative 10th Version), advanced age (over 65 years old) is a high-risk factor for severe/critical cases. Based on clinical practice, expanding the upper age limit is beneficial to more elderly patients.
Synopsis, 4.2<br>Exclusion Criteria<br>(7) and (8)	Delete "(7) Use of anti-cancer drugs, anti-rejection drugs after a transplant, or immunomodulatory biologics (within 30 days or 5 half-lives [whichever is longer] before enrollment);<br>(8) Long-term use of glucocorticoids at a daily dose equivalent to > 10 mg oral prednisone for more than 3 months (10 mg oral prednisone every other day is allowed);"	According to the actual clinical analysis, the above drugs were not limited, which was beneficial for patients with immunodeficiency or patients requiring long-term use of the above drugs
Study Schedule	Updated the following text as follows (new text in bold):<br>Urinalysis [j] | X[**k**]<br><br>Hepatitis and serological tests (HIV, HBV, and HCV) | X[**k**]	Limited the acceptable time frame for urinalysis, hepatitis and serological test results
Study Schedule -	Added the following contents:	Patients discharged within D14 without

Remark "a"	If a patient has been discharged before D14, a telephone follow-up on D14 is acceptable. For those who do not achieve negative conversion, nucleic acid test results obtained from another hospital are acceptable.	special examination can meet the requirements by telephone visit
Study Schedule - Remark "f"	Updated the following text as follows (new text in bold):<br>Lung imaging (CT scan) will be performed ~~at screening or on D0 (pre-dose)~~ **before the first dose (results obtained within 72 h before the first dose are acceptable)**, on D7 **(± 1 d** ~~before the second dose~~) and D14 **(± 1 d)**. **If a patient is discharged before D14 or meets COVID-19 discharge criteria, a CT scan is not required in subsequent visits; however, at least one CT scan should be performed at discharge.** Additional CT scans may be performed as needed depending on the changes in the subject's conditions.	For patients discharged within D14, whether to receive CT examination was explained in more detail for execution
Study Schedule - Remark "k"	Updated the following text as follows (new text in bold):<br>~~Laboratory tests will be performed~~ **Test results** within 72 h before the first dose ~~to determine enrollment eligibility~~ **is acceptable**	More specific description for execution
Study Schedule - Remark "I"	Added the following contents:<br>If the second dose is not administered, no sample will be collected on D7, D8 and D10.	Specific instructions for not administering the 2nd dose

Overall Reason for Modification in Protocol Modification Description 1

The protocol was revised, mainly to revise the corresponding contents according to the latest guidelines and regulations, improve the usage of the study drug, clarify the definition of some secondary evaluation indicators, modify the assignment method of subjects, improve the blind content of the study, add the blinding methods and processes, as well as the division of labor between blinded and unblinded personnel and their respective workflows, and improve the definitions of adverse event, serious adverse event and adverse reaction; update and improve the recording method for death events caused by adverse reactions; add updates to SUSAR reporting and the process for collecting and reporting SAEs; add the details of time limit requirements for expedited reporting, and add the reporting channels for expedited reporting; update the method for determining pregnancy and process for reporting pregnancy events, add missing information and correct errors and inconsistencies.

Table 7 Description of Changes in Protocol Modification Description 1

Section Number and Title	Description of Change	Reason for Revision

Section Number and Title	Description of Change	Reason for Revision
Cover and Text: Study Protocol Name	Updated the following text as follows (new text in bold):<br>A Multicenter, Double-blind, Randomized, Placebo-controlled, Add-on Phase III Clinical Study to Evaluate the Safety and Efficacy of Meplazumab for Injection in Patients with- ~~COVID-19 Severe~~ **Severe SARS-CoV-2 Infection**	Updated the disease name according to the requirements of the newly issued *Diagnosis and Treatment Protocol for COVID-19 Patients (Tentative 10th Version)*.
Cover: Version No. and Date	Updated to:<br>Version No.: V1.1. Version date: January 6, 2023	Updated version No. and version date.
Cover: Study Period	Deleted "Study Period: January 2023–January 2024"	Deleted unnecessary content of the document.
Compliance Statement, 13 Adverse Event Reporting	Updated the following text as follows (new text in bold):<br>2) *Provisions for Drug Registration* (~~2007~~**2020**)	Updated according to the latest version of regulations.
Signature Page	Updated the following text as follows (new text in bold):<br>~~Phase I Clinical Group~~**Pharmacy Department**, The First Affiliated Hospital of Fourth Military Medical University	Corrected the name of department involved in the study
Synopsis: Indication, 4 Study Population	Updated the following text as follows (new text in bold):<br>Patients diagnosed with severe virus infection as per the *Diagnosis and Treatment Protocol for COVID-19 Patients (Tentative ~~9th~~**10th** Version)* issued by the National Health Commission (NHC) of China.	Updated the disease name according to the requirements of the newly issued *Diagnosis and Treatment Protocol for COVID-19 Patients (Tentative 10th Version)*.
Synopsis: Study Objectives, 2 Study Objectives	Updated the following text as follows (new text in bold):<br>To evaluate the safety and efficacy of Meplazumab for Injection when added to the standard of care, compared to the standard of care alone, in patients with ~~COVID-19~~**SARS-CoV-2 infection**.	Updated the disease name according to the requirements of the newly issued *Diagnosis and Treatment Protocol for COVID-19 Patients (Tentative 10th Version)*.
Synopsis: Study Design, 17	Updated the following text as follows (new text in bold):	Updated and improved the

Section Number and Title	Description of Change	Reason for Revision
References	The study is based on the standard of care protocol (SoC) in the *Diagnosis and Treatment Protocol for COVID-19 Patients (Tentative ~~9th~~**10th** Version)* [1], the ***Technical Guidelines for Clinical Trials of New Antiviral Drugs for COVID-19 (Trial)*** **[2]** and previous clinical study results	justification for protocol formulation, and added corresponding literature in the References.
Synopsis: Study Design	Changed "Tentative 9th Version" to "Tentative 10th Version"	Updated relevant contents according to the requirements of the newly issued *Diagnosis and Treatment Protocol for COVID-19 Patients (Tentative 10th Version)*.
Synopsis, 4.1: Inclusion Criteria	(2) ~~Adults~~Subjects diagnosed with SARS-CoV2 infection in the laboratory by PCR nucleic acid amplification test	Deleted repeated description due to the age range defined in Criterion 1 of the Inclusion Criteria.
Synopsis, 4.1: Inclusion Criteria	Updated the following text as follows (new text in bold):<br>(3) Patients diagnosed with severe SARS-CoV-2 infection as per the *Diagnosis and Treatment Protocol for COVID-19 Patients (Tentative ~~9th~~**10th** Version)* issued by NHC;	Updated relevant contents according to the requirements of the newly issued *Diagnosis and Treatment Protocol for COVID-19 Patients (Tentative 10th Version)*.
Synopsis, 4.1: Inclusion Criteria	Updated the following text as follows (new text in bold):<br>(4) Subjects who agree to take effective non-drug contraceptive measures from signing the ICF to ~~6~~**3** months after the end of the study;	Revised as per study period
Synopsis, 4.1: Inclusion Criteria	(5) Subjects who are capable of comprehending the study, willing to participate in the study and sign the ICF (for incapable subjects whose participation is deemed beneficial by the investigator, their legal guardians may sign the ICF on their behalf~~, or inform them of consent by telephone (audio recording)~~ with explanations recorded in relevant documents such as	Deleted since all patients involved in this study are hospitalized and there is no telephone notification.

Section Number and Title	Description of Change	Reason for Revision
	original medical records).	
Synopsis, 4.2: Exclusion Criteria	Updated the following text as follows (new text in bold):<br>(2) Patients diagnosed with critical SARS-CoV-2 infection as per the *Diagnosis and Treatment Protocol for COVID-19 Patients (Tentative ~~9th~~**10th** Version)* issued by NHC;	Updated relevant contents according to the requirements of the newly issued *Diagnosis and Treatment Protocol for COVID-19 Patients (Tentative* 10th *Version)*.
Synopsis, 4.2: Exclusion Criteria	Combined Exclusion Criteria 3 and 13 to "(3) Subjects with stage 4 severe chronic kidney disease or requiring dialysis (estimated glomerular filtration rate [eGFR] <30 mL/min/1.73 m^2), or an increase in serum creatinine of 44.2 µmol/L within 7 days, or oliguria (<400 mL/24 h) or anuria (<100 mL/24 h);"	Combined associated exclusion criteria.
Synopsis, 4.2: Exclusion Criteria	Modified exclusion criterion (6) from "individuals allergic to any of the investigational products" to "individuals known to be allergic to the investigational product and its components"	Specified the description of drug allergy.
Synopsis, 4.2: Exclusion Criteria	Updated the following text as follows (new text in bold):<br>(7) Use of anti-cancer drugs, anti-rejection drugs after a transplant, or immunomodulatory biologics (within 30 days or 5 half-lives [whichever is longer] ~~after~~**before** enrollment);	Corrected the incorrect description in Version 1.0.
Synopsis, 4.2: Exclusion Criteria	Added the following contents:<br>(13) Patients with other factors deemed by investigators as not suitable for the trial.	Added and improved the exclusion criteria
Synopsis: Study Drug, Usage	Change "Intravenous infusion" to "Dissolve Meplazumab for Injection in 1 mL of sterile water for injection and add the resulting solution to 100 mL of 0.9% sodium chloride for IV drip over 30–60 min. (Placebo: 100 mL of 0.9% sodium chloride, IV drip.)"	Improved the original content and described the infusion method in detail
Synopsis, 9.1: Secondary	Updated the following text as follows (new text in bold):	Revised according to the new

Section Number and Title	Description of Change	Reason for Revision
Evaluation Indicators	2) ~~Proportion of subjects who are alive and not receiving oxygen supplementation at discharge~~ **Discharge rate** on Day 28 (D28)	diagnosis and treatment protocol because there is no clear requirement for oxygen supplementation in the discharge criteria according to the newly issued *Diagnosis and Treatment Protocol for COVID-19 Patients (Tentative 10th Version).*
Synopsis, 9.1: Secondary Evaluation Indicators	Updated the following text as follows (new text in bold):<br>4) Time to sustained recovery (days; time range: D0 to D28), defined as the first day ~~to meet~~ **at which** one of the following **is achieved**: 1. ~~without hospitalization~~ **discharge from hospital**; 2. hospitalization without oxygen ~~supplementation~~ **inhalation** requirement	Corrected the original text description.
Synopsis, 9.1: Secondary Evaluation Indicators	5) Duration of ~~oxygen use and days without~~ oxygen supplementation (days)	Simplified the content due to the consistent observation contents of the two indicators.
Synopsis, 9.1: Secondary Evaluation Indicators	6) Duration of mechanical ~~and non-mechanical~~ ventilation (days)	Simplified the content due to the consistent observation contents of the two indicators.
Synopsis, 9.1: Secondary Evaluation Indicators	7) Incidence ~~and duration~~ of new mechanical ventilation use **and incidence of re-ventilation use and duration of ventilation 24 hours after new withdrawal** (days)	Clarified the definition of "new mechanical ventilation".
Synopsis, 9.1: Secondary Evaluation Indicators	Updated the following text as follows (new text in bold):<br>9) SARS-CoV-2 nucleic acid negative conversion rate on **D7**, **D14**, and D28	Added observation points on D7 and D14, and increased the integrity of evaluation for this indicator
Synopsis, 9.1: Secondary	Updated the following text as follows (new text in bold):	Clarified the specific evaluation

Section Number and Title	Description of Change	Reason for Revision
Evaluation Indicators	10) **Proportion of subjects** with improvement in lung imaging **(defined as any of the following changes from baseline in lung imaging during the study: 1) reduction in lesion diameter; 2) decrease in the number of lesions; 3) decrease in the number of lung lobes involved by lesions)**	indicators of lung imaging improvement.
Synopsis, 9.1: Secondary Evaluation Indicators	Updated the following text as follows (new text in bold):<br>11) Changes from **D0 (predose)** in the levels of cytokines and chemokines associated with inflammatory and immune states on D1, **D4**, D7 (before the second dose), D8, **D10** and D28, including CRP, L-2, IL-4, IL-6, INF-α, IL-8, IL-10, IL-12p70, IL-17, IL-1β, TNF-α and IFN-γ	Added observation time points, increased the integrity of evaluation for this indicator, and improved the time points for comparing changes in indicators.
Synopsis, 9.1: Secondary Evaluation Indicators	Updated the following text as follows (new text in bold):<br>12) Changes from D0 (pre-dose) in lymphocyte subsets CD3+, CD4+ and CD8+ on D0 (pre-dose), D1, **D4**, D7 (before the second dose), D8, **D10** and D28	Added observation time points, increased the integrity of evaluation for this indicator, and improved the time points for comparing changes in indicators.
Synopsis, 9.3: Safety Evaluation	Updated the following text as follows (new text in bold):<br>Before and after each dose, ~~respiration should be measured and~~ **infusion safety monitoring should be performed, including** blood pressure **(systolic and diastolic blood pressure)**, pulse~~, body temperature,~~ **and** finger oxygen saturation **SpO_2 or**~~/~~ oxygenation index, **respiration and body temperature, which should be measured before each dose (within 30 min) and at 1 h ± 10 min, 6 h ± 1 h and 12 h ± 1 h after each dose;** ~~respiration, blood pressure, pulse, body temperature and oxygen saturation/oxygenation index are measured every 6 hours for 24 hours after administration;~~ hematology, blood biochemistry, urinalysis, **chest imaging, ECG and other indicators** will be measured after administration in the treatment period.	Revised and incorporated the text description, and clarified the time points and time windows for post-dose observation indicators.
5 Treatment Assignment	Updated the following text as follows (new text in bold):	Updated the subject assignment

Section Number and Title	Description of Change	Reason for Revision
and Blinding	Eligible subjects will be randomized to the Meplazumab group or placebo group in a 1:1 ratio according to the principle of randomization, **and subject randomization will be completed using an interactive web response system (IWRS)**. The randomization statistician will use SAS9.4 or higher version software to generate the **Subject** Randomization Schedule by **stratified** blocked randomization. **The stratification factor is age (age < 65 years old or ≥ 65 years old), and the schedule will be imported into the IWRS by the system engineer. After the subjects are successfully screened, the authorized investigators will log in to the IWRS to randomize the subjects and generate a randomization number, obtain the treatment group assignment information of the subjects, and collect the drugs of the corresponding groups for the subjects according to the system prompt to complete drug preparation and injection; in case of drug damage and other conditions, the unblinded investigator may re-collect the drugs for the same group to complete drug preparation and injection for the subjects.** ~~. The investigators at each site assign the randomization numbers of the corresponding sites in turn in strict accordance with the enrollment order of eligible subjects after screening, and obtain and inject the investigational products according to the numbers.~~<br>~~An additional 70 spare drugs are prepared for this trial, numbered B001-B070. The subjects are divided into Meplazumab group or placebo group according to the ratio of 1:1. A spare drug randomization schedule is generated by the randomization statistician using SAS 9.4 or above. When the spare vaccines need to be used, the investigator will log in to the spare drug acquisition system to obtain the corresponding spare drug number.~~<br>~~The drug blind codes are prepared by the randomization statistician, sealed in duplicate and kept by the sponsor and investigator, respectively.~~<br>~~Blinding of investigational products: This trial is blinded. The randomization statistician and other blinding personnel performed drug blinding, that is, the printed drug label is pasted to the~~	method from the original manual blinding to IWRS system, so as to improve the study efficiency and preciseness.

Section Number and Title	Description of Change	Reason for Revision
	~~designated location of each drug according to the blinding code. The randomization statistician should supervise the drug blinding and guide the blinding operators to label according to the blinding codes. After blinding, the blinding codes should be sealed by the randomization statistician. The whole process of blinding should be documented. The blinding personnel should not participate in other relevant work of this clinical study, nor should they disclose the blinding codes to any personnel participating in this clinical study.~~	
5.1 Study Blindness	Revised "The preparation of the study drug is performed by an independent medical staff, and the medical staff responsible for dispensing will prepare the study drug according to the drug group to which the subject is assigned. The dispensing nurse cannot disclose relevant information about therapeutic drugs to subjects and any other personnel, nor participate in all subsequent study evaluation processes." to "In this study, the investigational drug should be dissolved with sterile water for injection and then prepared into 100 mL of 0.9% normal saline, while the placebo is used as the control, so it is proposed to directly use 0.9% normal saline. Double-blind in the strict sense cannot be achieved due to the different appearances of the two drugs. In this study, the traditional packaging blinding of the investigational product cannot be performed before the trial. To avoid the bias on the study results caused by people obtaining specific grouping information, the blindness assurance work plan in this study is mainly carried out as follows:<br>1） It is planned to set up a blinded team and an unblinded team at the site level to conduct the study. The unblinded study personnel include drug management personnel, personnel logging into the IWRS to obtain treatment grouping information, and drug preparation personnel. The remaining study personnel will remain blinded.<br>2） The project management and study monitoring team will have blinded and unblinded monitors. The unblinded monitors will perform site-specific drug accountability and monitor whether the subjects' drug preparation and administration process meet the protocol	Improved the blind content of the study, and added the blinding method and process, as well as the division of labor between blinded and unblinded personnel and their respective workflows.

Section Number and Title	Description of Change	Reason for Revision
	requirements. The remaining study personnel will remain blinded.<br>3） Drug preparation: Unblinded personnel will log in to the IWRS to obtain treatment grouping information and complete solution preparation before administration. After preparation, the appearance of drugs in both the test group and control group will be consistent to maintain blindness. The drug preparation personnel cannot disclose relevant information about therapeutic drugs to subjects and any other persons, nor participate in all subsequent study evaluation processes.<br>4） After the start of the trial, both study members in the blinded group and unblinded group should ensure that they strictly follow the protocol requirements to carry out trial operations within their respective responsibilities."	
5.2 Emergency Unblinding	Revised "The randomization statistician prepares emergency letters for the trial at the same time as making blind codes to meet individual emergency unblinding needs. Each site is equipped with 5 emergency letters, which are sent to each site along with blinded drugs and kept by the responsible investigator of the site" to "When the specific treatment grouping information of a subject needs to be obtained to treat the subject due to serious adverse events or other emergencies, the authorized investigator can perform emergency unblinding for individual subjects through the emergency unblinding module of IWRS system to obtain the specific grouping information of the subject. If possible, prior to unblinding the investigational product, the authorized investigator must notify the Principal Investigator and relevant personnel of the sponsor first, and obtain approval from the Principal Investigator before obtaining specific subject grouping information. If the sponsor is not contacted before emergency unblinding, the investigator must contact the sponsor within 24 hours after unblinding. The investigator should record the date and reason for the unblinding of the subject, as well as the operation process of unblinding in the source documents."	Updated and improved the process and method of emergency unblinding based on the IWRS system.
6.2 Method of	Updated the following text as follows (new text in bold):	Revised the language description

Section Number and Title	Description of Change	Reason for Revision
Administration	The dose determined through the ~~Phase I~~ **previous** clinical study is 0.2 mg/kg<br>The drip infusion should be completed within ~~1 h~~ **30-60 min**	to clarify the infusion time.
6.4.2 Treatment phase	Updated the following text as follows (new text in bold):<br>• Vital signs ~~(including respiratory rate and finger oxygen saturation/oxygenation index)~~: pre-dose, 1 h ± 10 min, ~~and then once every~~ 6 **± 1** h ~~to 24 h after dosing, monitoring will be performed every 6 h with dosing time as baseline and window periods of 6 ± 1 h~~ **and 12 ± 1 h** after the end of dosing;<br>• The antibody is dissolved in 1 mL of sterile water for injection, and the ~~maximum tolerated~~ dose determined through the ~~Phase I~~ **previous** clinical study is **0.2 mg/kg**	Simplified the text description, added a time point of 12 ± 1 h, and clarified the dose.
8 Concomitant Therapy	Changed "9th Version" to "10th Version"	Updated relevant contents according to the requirements of the newly issued *Diagnosis and Treatment Protocol for COVID-19 Patients (Tentative 10th Version)*.
9.2 Efficacy Evaluation	Revised "Before administration, until the Ct values of N gene and ORF gene in two consecutive SARS-CoV-2 nucleic acid tests are ≥ 35 (by fluorescent quantitative PCR method with a cutoff value of 40 and sampling time interval of at least 24 hours), or until two consecutive SARS-CoV-2 nucleic acid tests are negative (by fluorescent quantitative PCR method with a cutoff value of less than 35 and sampling time interval of at least 24 hours), if the subject withdraws early, laboratory testing and chest imaging should be performed as much as possible to complete the efficacy evaluation.<br>If a subject is discharged according to the criteria of "Ct values of N gene and ORF gene in two consecutive SARS-CoV-2 nucleic acid tests are ≥ 35 (by fluorescent quantitative PCR method with a cutoff value of 40 and sampling time interval of at least 24 hours)", it will be considered as "negative" conversion and recorded in the medical record." to "Nucleic acid	The determination criteria for Ct value of nucleic acid negative conversion and the specific detection genes are based on the standard methods approved by the People's Republic of China, which will not be described in the protocol. Clarified the definition of nucleic acid negative conversion.

Section Number and Title	Description of Change	Reason for Revision
	negative conversion is defined as two consecutive SARS-CoV-2 nucleic acid tests with negative results (sampling time interval of at least 24 hours) are judged as nucleic acid negative conversion, and the sampling time of the first negative nucleic acid test is taken as the nucleic acid negative conversion time."	
11.2 Early Termination/Withdrawal: Handling of Withdrawals	Updated the following text as follows (new text in bold):<br>All existing study-related toxicities and SAEs at the time of withdrawal from the study must be followed up until they are ~~relieved~~ **resolved or corresponding indicators return to baseline levels, unless in the opinion of the investigator,**<br>If it is an SAE, it must be reported to the Sponsor within 24 hours and followed up until the above adverse event is ~~relieved~~ **resolved or returned to the baseline level.**	Defined "relieved" as "resolved or returned to the baseline level" specifically in the new version due to the vague original definition of "relieved".
13.1.2 Definition and Classification of Adverse Events: Definition of Adverse Event	Modify the definition of adverse event (AE) as: "It refers to any adverse medical event, experienced by a subject administered an investigational drug, which may present with symptoms, signs, diseases, or laboratory test abnormalities but does not necessarily have a causal relationship with the investigational drug."	Improved the definition of adverse event.
13.1.2 Definition and Classification of Adverse Events: Definition of Serious Adverse Event	Revised the definition of serious adverse event to:<br>"A serious adverse event is any untoward medical event in a subject after administration of the investigational product that meets one or more of the following criteria:<br>（1） Resulting in death;<br>（2） Result in life-threatening consequences;<br>（3） Resulting in hospitalization or prolongation of existing hospitalization;<br>（4） Resulting in significant or permanent disability/incapacity;<br>（5） Resulting in congenital anomaly or birth defect;<br>（6） Resulting in other important medical events: For example, important medical events that are not immediately life-threatening, or fatal, or require hospitalization but may jeopardize the patient, or require medical intervention to prevent the outcomes listed above,	Improve the definition of serious adverse events.

Section Number and Title	Description of Change	Reason for Revision
	scientific medical judgment should be made immediately. These should also be considered SAEs."	
13.1.2 Definition and Classification of Adverse Events: Definition of Adverse Reaction	Modify the definition of adverse drug reaction (ADR)as:"It refers to reactions harmful to the human body or unintended reactions that are considered to be related to the investigational product in a clinical trial. The investigational product and the AE are at least in one possibly reasonable causality, i.e., the correlation cannot be ruled out."	Improved the definition of adverse reaction.
13.6 Outcome of Adverse Events	（5） ~~Fatal~~**Death**~~: End date of (serious) AE should be noted. When an adverse event cause death,~~ **End date of (serious) AE" should be indicated for the death caused by an AE, and "end date of (serious) AE" is not required for death not caused by an AE. If the subject dies,** the time of death should be recorded;	Updated and improved the recording methods for death events caused by adverse reactions and events.
Cases of Special Reporting	Deleted "Section 13.7 Cases of Special Reporting"	Relevant contents have been included in the expedited report
13.7.2 Collection and Reporting of Serious Adverse Events: Collection and Reporting of SAEs	Updated the following text as follows (new text in bold):<br>Whether it is related to the investigational ~~drug~~ **product** or not, the investigator should give timely rescue treatment and ~~fill in the *Serious Adverse Event (SAE) Report Form* of National Medical Products Administration (NMPA) within 24 hours after being informed~~ **fill in the *SAE Report Form* formulated for this trial as detailed as possible,** sign and date~~, and immediately report to the sponsor (or CRO designated by the sponsor), the Ethics Committee of the study site, National Medical Products Administration (NMPA), provincial and municipal local medical products administrations, as well as the Bureau of Medical Administration, National Health Commission of the People's Republic of China by fax~~. **The investigator should report the SAE to the sponsor (or CRO designated by the sponsor) by email within 24 hours after becoming aware of it. The sponsor (or the CRO designated by the sponsor) will conduct an assessment, and then report the assessed suspected unexpected serious adverse reactions (SUSARs) to the principal investigator and project manager as**	Updated the process for collecting and reporting SAEs due to the addition of SUSAR reporting in the study.

Section Number and Title	Description of Change	Reason for Revision
	**well as their clinical trial institution and ethics committee in a rapid manner, and report to the national drug regulatory authority and health authorities. The investigator should sign and read the relevant safety information of the clinical study provided by the sponsor (or CRO designated by the sponsor) in a timely manner after receiving it, and report the SUSAR report provided by the sponsor (or CRO designated by the sponsor) to the ethics committee.**	
13.7.2 Collection and Reporting of Serious Adverse Events: Expedited Reporting	Added the following contents:<br>Time limit requirements for expedited reporting:<br>(I) For SUSARs that are fatal or life-threatening, the sponsor (or the CRO designated by the sponsor) should report them as soon as possible, but not more than 7 days after initial awareness and submit a complete follow-up report within 8 days after the first report.<br>(II) For SUSARs that are not fatal or life-threatening, the sponsor (or the CRO designated by the sponsor) should report them as soon as possible, but not more than 15 days after initial awareness. After report submission, serious adverse reactions should continue to be followed up. Any new information or changes to the previous report should be promptly submitted in the form of a follow-up report, within 15 days of receiving the new information.	Added details of the time limit for expedited reporting, and reporting channels for expedited reporting.
13.10 Pregnancy	Delete "Pregnancy will be determined by serum pregnancy test. Serum pregnancy test is required for female subjects of childbearing age during the screening period, but most laboratories within the COVID-19 closed-loop at study sites do not provide serum tests and only provide urine tests. Therefore, the investigator can assess whether the patient is pregnant by combining the menstrual history of the subject with the results of a urine pregnancy test, instead of a serum pregnancy test."<br>Updated the following text as follows (new text in bold):<br>All pregnancy events must be reported. The investigator should ~~use the Preliminary Pregnancy Report Form~~**fill in the *Pregnancy Event Report Form* of the clinical trial** and submit it to the	Updated the method for determining pregnancy and the process for reporting pregnancy events.

Section Number and Title	Description of Change	Reason for Revision
	sponsor within 30 days after being informed of the pregnancy **event**.<br>All pregnancy outcomes must be reported ~~by the investigator to the sponsor using the Pregnancy Outcome Report Form within 30 days of learning of normal delivery or elective abortion~~**, pregnancy events in the study should be followed up to the end of pregnancy or 1 month after birth as far as possible, and follow-up information should be reported in the form of *Pregnancy Event Report Form*.**	
14.3 Drug Packaging and Label	Added the following contents:<br>14.3 Drug Packaging and Label<br>The production, packaging and labeling of the investigational product will be carried out in accordance with the Good Manufacturing Practice (GMP) and applicable laws and regulations. The investigational product will be appropriately packaged to protect it from contamination during transportation and storage.<br>The investigational product will be appropriately labeled in accordance with the GCP and GMP, and the label will include instructions that meet regulatory requirements. The drug label shall at least include the following contents: clinical trial applicant, name of investigational product, usage, strength, batch number, protocol number or other unique code corresponding to the clinical trial used, drug number, shelf life, storage conditions, and texts "For Clinical Study Use Only".	Added the requirements for drug packaging and labeling.
17 References	Added the following contents:<br>[1]. Diagnosis and Treatment Protocol for COVID-19 Patients (Tentative 10th Version). 2023.<br>[2]. Technical Guidelines for Clinical Trials of New Antiviral Drugs for COVID-19, 2022.<br>[3]. Rong Yan, Analysis on Clinical, Imaging and Serum Antibody Characteristics of Mild and Moderate COVID-19, 2021, Southern Medical University.	Added references

Section Number and Title	Description of Change	Reason for Revision
	[4]. Fang Xu, et al. Clinical features and chest CT findings in moderate and severe COVID-19 patients: an analysis of 506 cases from Wuhan Huoshenshan Hospital. Chinese Journal of Clinical Infectious Diseases, 2020. 03(13): p161-166.	
Study Schedule - Header	Deleted "168 h, 336 h, 672 h and 1344 h"	Optimized time description
Study Schedule - Header	Added "Evaluation Procedure"<br>Changed "**day ± time window**" to "**visit time**"<br>Deleted "Randomization" and extra space	Adjusted the incorrect table format, and deleted extra space
Study Schedule - Left First Column	Updated the following text as follows (new text in bold):<br>Demographic and medical history ~~Disease~~<br>Lung imaging (~~X-ray,~~ CT scan)<br>~~Targeted physical examination~~<br>**Concomitant Medications**<br>~~Infusion-related safety procedures~~ **Drug infusion safety monitoring** [h]<br>Days of **low-flow** oxygen supplementation**, (**~~low-flow~~ nasal cannula, simple mask**)**<br>Days of high-flow ~~oxygen device use~~ **oxygen supplementation (**Venturi mask, high-flow nasal cannula**)**<br>Safety hematology, blood chemistry and ~~liver function test~~ [i] **coagulation** [j]<br>**Blood** pregnancy test for women of childbearing potential	Combined duplicates and optimized assessment/procedure description
Study Schedule - Remark	Revised Remark a to: "Subjects are required to undergo scheduled visits on site. However, if a subject is unable to return to the study site due to epidemic quarantine or other factors, post-discharge remote or home visits are acceptable."	Combined similar items and optimized the description
Study Schedule - Remark	Add "If a subject has been discharged before dosing on D7, no more dosing is required on D7."<br>Deleted "Clinical data and efficacy data to be collected include: oxygen demand and ventilator oxygen demand" "Any laboratory test performed within a defined visit window as part of routine clinical care may be used for safety laboratory test."	Clarified the definition and deleted the repetition

Section Number and Title	Description of Change	Reason for Revision
Study Schedule - Remark	Updated the following text as follows (new text in bold):<br>Perform lung imaging (~~X-ray,~~ CT scan). Additional CT scans may be performed as needed ~~depending on~~**according to** the changes in the subject's conditions.	Deleted X-ray
Study Schedule - Remark	Updated the following text as follows (new text in bold):<br>Dates recorded include: ....date of discharge from ICU **(if any)**, **date of admission and date of discharge**.	Added the dates to be recorded
Study Schedule - Remark	Revised Remark b to:<br>"Medical history records: COVID-19 history and other past medical histories, such as chronic lung disease (including asthma), chronic kidney disease, diabetes mellitus, heart disease, hypertension, autoimmune diseases, tumors and organ transplantation."	Optimized description
Study Schedule - Remark	Revised Remark e to:<br>Physical examination: Appearance, eyes, ears, nose, throat, thyroid/neck, lymph nodes, heart, lungs, gastrointestinal tract, abdomen, skeletal muscle/extremities, skin, and neurology/psychiatry.	Revised inspection items
Study Schedule - Remark	Body temperature, ~~pulse rate (radial)~~ **pulse**...~~and blood~~ finger oxygen saturation (SpO_2)~~/~~ **or** oxygenation index.	Corrected the errors
Study Schedule - Remark	Revised Remark h to:<br>Drug infusion safety monitoring: Vital signs, including blood pressure (systolic and diastolic), pulse rate, blood oxygen saturation SpO2 or oxygenation index, respiration rate and body temperature, will be measured before each dose (within 30 min) and 1 h ± 10 min, 6 h ± 1 h and 12 h ± 1 h after each dose during the treatment period.	Revised the contents of safety monitoring and clarified the test time points.
Study Schedule - Remark	Revised Remark j to:<br>Safety hematology, blood chemistry, coagulation and urinalysis:<br>Urinalysis: White blood cells (WBC), red blood cells (RBC), pH and protein;<br>Hematology: WBC, RBC, hemoglobin (HGB), platelet (PLT) count, absolute lymphocytes, absolute neutrophils, absolute monocytes, absolute eosinophils, absolute basophils, hematocrit (HCT), mean corpuscular volume (MCV), mean corpuscular hemoglobin (MCH), and mean	Clarified the contents of safety test items

Section Number and Title	Description of Change	Reason for Revision
	corpuscular hemoglobin concentration (MCHC);<br>Blood chemistry: Liver function (total protein, albumin, total bilirubin [TBL], direct bilirubin, indirect bilirubin, alanine aminotransferase [ALT], aspartate aminotransferase [AST], alkaline phosphatase and gamma-glutamyl transferase); renal function (urea, creatinine, uric acid, cystatin C); lactate dehydrogenase, cardiac troponin, D-dimer and ferritin; blood lipids (triglyceride, high-density lipoprotein, low-density lipoprotein and total cholesterol); postprandial glucose; electrolytes (sodium, potassium, chloride and calcium);<br>Coagulation: Prothrombin time, activated partial thromboplastin time, fibrinogen and thrombin time;	
Study Schedule - Remark	Updated the following text as follows (new text in bold):<br>Blood samples will be collected at the following time points, when available: pre-dose, ~~within 20 to 52 h after infusion (~~D1~~ or D2)~~, **D4**, before~~ within 20 to 52 h after~~ the second infusion~~ (D8 or D9)~~ (D7), **D8**, **D10** and D28 (optional).	Clarified sampling time points
Study Schedule - Remark	Added the following contents:<br>"m. If the first nucleic acid test is negative, the test should be repeated at least 24 hours later. If a subject is tested negative for nucleic acid before discharge (two consecutive negative results, at least 24 hours apart), no more nucleic acid testing is required at subsequent visits; if a subject does not achieve negative conversion before discharge, nucleic acid testing must continue at subsequent visits."	Clarified the nucleic acid test due to the change in guidelines

A Multicenter, Double-blind, Randomized, Placebo-controlled, Add-on Phase III Clinical Study to Evaluate the Safety and Efficacy of Meplazumab for Injection in Patients with Severe SARS-CoV-2 Infection

Statistical Analysis Plan

Sponsors: Jiangsu Pacific Meinuoke Bio-pharmaceutical Co., Ltd.

Fourth Military Medical University

Study Sites: Department of Immunology, Xijing Hospital of Fourth Military Medical University

Phase I Clinical Group, Xijing Hospital of Fourth Military Medical University

Department of Respiratory Medicine, Xijing Hospital of Fourth Military Medical University

Department of Critical Care Medicine, Xijing Hospital of Fourth Military Medical University

Statistical Unit: Beijing Key Tech Statistical Technology Co., Ltd.

Approval Page

Statistical Unit

Statistical Unit: Beijing Key Tech Statistical Technology Co., Ltd.

Statistician: Ye Tian

Signature: Date:

Responsible Person: Zhiwei Jiang

Signature: Date:

Approval Page

Sponsor

Sponsors: Jiangsu Pacific Meinuoke Bio-pharmaceutical Co., Ltd.
Fourth Military Medical University

Sponsor's Representative: Shuangshuang Liu

Signature: Date:

Table of Contents

1. List of Abbreviations and Definition of Terms 1
2. Study Description 1
   2.1 Introduction 1
   2.2 Purpose of Analysis 2
   2.3 Changes in Statistical Analysis Compared to Protocol 2
3. Study Design 2
   3.1 Overall Design 2
   3.2 Randomization 2
   3.3 Sample Size 3
4. Study Objectives, Endpoints and Estimands 7
   4.1 Study Objectives and Endpoints 7
   4.2 Estimands 8
      4.2.1 Primary Estimand 8
      4.2.2 Secondary Estimand 8
5. Analysis Sets 10
6. Statistical Analysis Methods 11
   6.1 General Considerations 11
      6.1.1 General Analytical Methods 11
      6.1.2 Relevant Definitions and Derived Rules 11
      6.1.3 Statistical Analysis Window 12
      6.1.4 Analysis Software 12
      6.1.5 Tables and Listings 12
   6.2 Enrollment and Completion of the Trial 13
   6.3 Demographic and Baseline Characteristics 13
   6.4 Protocol Violations/Deviations 13
   6.5 Concomitant Medication/Non-Drug Therapy 14
   6.6 Study Hypothesis 14
   6.7 Efficacy Evaluation 14
      6.7.1 Analysis of Primary Estimand 14
      6.7.2 Analysis of Secondary Estimand 15
   6.8 PD Analysis 17
   6.9 Immunogenicity Analysis 18
   6.10 Safety Analysis 18
   6.11 Interim Analysis 20
   6.12 Subgroup Analysis 20
   6.13 Multiplicity Handling 20
   6.14 Handling of Dropouts or Missing Data 20
Version History 22

1. List of Abbreviations and Definition of Terms

ATC	Anatomical Therapeutic Chemical Classification
BMI	Body Mass Index
COVID-19	Corona Virus Disease 2019
CRF	Case Report Form
FAS	Full Analysis Set
GMT	Geometric Mean Titer
ICU	Intensive Care Unit
ITT	Intention-to-treat
IWRS	Interactive Web Response System
Max	Maximum
Mean	Mean
MedDRA	Medical Dictionary for Regulatory Activities
Median	Median
Min	Minimum
PPS	Per Protocol Set
PT	Preferred Term
SAE	Serious Adverse Event
SAP	Statistical Analysis Plan
SARS-COV-2	Severe Acute Respiratory Syndrome Coronavirus 2
SD	Standard Deviation
SDTM	Study Data Tabulation Model
SOC	System Organ Class
SS	Safety Set
TEAE	Treatment Emergent Adverse Event
TPA	Tipping Point Analysis
WHO	World Health Organization

2. Study Description

2.1 Introduction

This document is the Statistical Analysis Plan (SAP) for "A Multicenter, Double-blind, Randomized, Placebo-controlled, Add-on Phase III Clinical Study to Evaluate the Safety and Efficacy of Meplazumab for Injection in Patients with Severe SARS-CoV-2 Infection". The SAP details the specific statistical analysis methods that will be used to analyze and report baseline characteristics and evaluate efficacy safety in subjects.

This Statistical Analysis Plan (SAP) will be finalized and approved prior to the database lock, and the corresponding statistical analysis and programming will be progressively improved as the study data accumulates until the database lock.

The tables, listings and figures (TLFs) for this SAP will be provided separately as attachments.

2.2 Purpose of Analysis

The SAP is designed to evaluate the safety and efficacy of Meplazumab for Injection when added to the standard of care, compared to the standard of care alone, in patients with severe SARS-CoV-2 infection. The corresponding statistical analysis results will be presented in the final SAP and clinical summary report, and will also be used for registration application, article publication, and other clinical needs of the product.

Post-hoc exploratory analysis allows further exploration of the study data, but it is not described in this SAP due to unpredictability. In case the post-hoc exploratory analysis is subsequently conducted, its corresponding statistical analysis methods will be detailed in the final statistical analysis report and clinical study report.

Additional analyses for other purposes, such as article publication, and requirements of supervision department or sponsor, are not described in this SAP due to unpredictability. In case the additional analyses are subsequently conducted, their corresponding statistical analysis methods will be detailed in the document presenting the additional results rather than in the final clinical study report.

2.3 Changes in Statistical Analysis Compared to Protocol

The SAP is consistent with the study protocol.

3. Study Design

3.1 Overall Design

This is a multicenter, double-blind, randomized, placebo-controlled, add-on phase III clinical study. It is estimated that 352 subjects will be randomized (1:1) to receive 0.2 mg/kg Meplazumab or placebo. During hospitalization, all subjects will receive standard of care for Coronavirus Disease 2019 (COVID-19) based on the medical judgment of the investigator. See Table 1 for the study schedule.

3.2 Randomization

Eligible subjects will be randomized to the Meplazumab group or placebo group in a 1:1 ratio according to the principle of randomization, and subject randomization will be completed using an interactive web response system (IWRS). The randomization statistician will use SAS9.4 or higher version software to generate the Subject Randomization Schedule by stratified blocked randomization. The stratification factor is age (age < 65 years old or ≥ 65 years old), and the schedule will be imported into the IWRS by the system engineer. After the subjects are successfully screened, the authorized investigators will log in to the IWRS to randomize the subjects and generate a randomization number, obtain the treatment group assignment information of the subjects, and

collect the drugs of the corresponding groups for the subjects according to the system prompt to complete drug preparation and injection; in case of drug damage and other conditions, the unblinded investigator may re-collect the drugs for the same group to complete drug preparation and injection for the subjects.

3.3 Sample Size

Based on the available clinical study results and dose justification, subjects in the 0.2 mg/kg dose group will enter Phase III clinical trial to compare with placebo + SoC. Previous results showed a mortality of 2.4%, 14.6%, 9.1%, and 14.6% in the 0.12 mg/kg, 0.2 mg/kg, 0.3 mg/kg, and placebo groups, respectively. Considering the mortality of the 3 dose groups in the previous clinical studies and the possibility of inherent variability in the data, and estimating the mortality in the placebo group as 15%, the mortality in the 0.2 mg/kg group is expected to decrease by 10% compared with the placebo group. The one-sided test α is 0.025, and the power is set at 0.8. It is calculated using the "Tests for Two Proportions" module in PASS 21 that at least 141 subjects are required for each group. Considering a 20% dropout rate, 176 subjects per group are required, for a total of 352 subjects.

Table 1 Study Schedule

Assessments/Procedures	Screening	Treatment					End of Treatment
**Visit Time**	**D-3 to D-1**	**First Dose (D0)**	**QD Until Discharge**	**D7 Post-Dose**	**D14[a] ± 3 Days Post-Dose**	**D28[a] ± 3 Days Post-Dose**	**D56[a] ± 7 Days Post-Dose**
**Enrollment**							
Informed consent	X						
Demographic and medical history [b]	X						
Date of symptom onset	X						
Inclusion/exclusion criteria	X						
**Study Intervention**							
Randomization		X					
Dosing with Meplazumab or placebo; subjects will be weighed prior to each dose on each dosing day to determine accurate dosage		X		X[c]			
SoC until discharge at the discretion of treating physician		X					
**Study Procedures**							
Admission and discharge dates [d]	X	X	X				
COVID-19 diagnosis date	X						
Physical examination [e]	X						
Lung imaging (CT scan) [f]	X	X (pre-dose)		X (before the second dose)	X		
Vital signs, including SpO_2/oxygenation index [g]		X[i]	X	X	X	X	
Ordinal Scale for Clinical Improvement	X	X[i]	X	X	X	X	X
- Concurrent medications	X	X[i]	X	X	X	X	X
AE evaluation	X	X	X	X	X	X	X
Drug infusion safety monitoring [h]		X		X			

Assessments/Procedures	Screening	Treatment					End of Treatment
**Visit Time**	**D-3 to D-1**	**First Dose (D0)**	**QD Until Discharge**	**D7 Post-Dose**	**D14[a] ± 3 Days Post-Dose**	**D28[a] ± 3 Days Post-Dose**	**D56[a] ± 7 Days Post-Dose**
Survival status (dead or not)		X	X			X	X
Days of low-flow oxygen supplementation Nasal cannulas and simple masks	X	X	X			X	
Days of high-flow oxygen supplementation Venturi masks and high-flow nasal cannulas	X	X	X			X	
Number of days with invasive mechanical ventilation		X	X			X	
Number of days without invasive mechanical ventilation		X	X			X	
**Safety Laboratory**							
Urinalysis [j]	X[k]		X (within 3 days before discharge)				
Safety hematology, blood chemistry and coagulation [j]	X[k]	X[i,]	If hospitalized, on D2, D4, D7, D10 (± 1 d) and the day of discharge (± 1 d)			X	
12-lead ECG		X[i]				X	
Blood pregnancy test for women of childbearing potential	X						X
Hepatitis and serological tests (HIV, HBV, and HCV)	X[k]						
**Study Laboratory**							
Blood collection for pharmacodynamic (PD) assessment [l]		X	X[l]	X[l]		X	
ADA assessment		X[i]				X	X
Nasopharyngeal swab collection for nucleic acid testing [m]		X[i]	X[m]	X[m]	X[m]	X[m]	X[m]

Abbreviations: CT = computed tomography; ECG = electrocardiogram; HBV = hepatitis B virus; HCV = hepatitis C virus; HIV = human immunodeficiency virus.

a. Subjects are required to undergo scheduled visits on site. However, if a subject is unable to return to the study site due to epidemic quarantine or other factors, post-discharge remote or home visits are acceptable. If a patient has been discharged before D14, a telephone follow-up on D14 is acceptable. For those who do not achieve negative conversion, nucleic acid test results obtained from another hospital are acceptable.
b. Medical history records: COVID-19 history and other past medical histories, such as chronic lung disease (including asthma), chronic kidney disease, diabetes mellitus, heart disease, hypertension, autoimmune diseases, tumors and organ transplantation.
c. If a subject has been discharged before dosing on D7, no more dosing is required on D7.
d. Dates to be recorded: The dates of admission to and discharge from ICU, the dates of re-admission to and re-discharge from ICU (if available), and the dates of admission to and discharge from hospital.
e. Physical examination: Appearance, eyes, ears, nose, throat, thyroid/neck, lymph nodes, heart, lungs, gastrointestinal tract, abdomen, skeletal muscle/extremities, skin, and neurology/psychiatry.
f. Lung imaging (CT scan) will be performed before the first dose (results obtained within 72 h before the first dose are acceptable), on D7 (± 1d) and D14 (± 1d). If a patient is discharged before D14 or meets COVID-19 discharge criteria, a CT scan is not required in subsequent visits; however, at least one CT scan should be performed at discharge. Additional CT scans may be performed as needed depending on the changes in the subject's conditions.
g. Body temperature, pulse rate, respiratory rate, blood pressure (systolic and diastolic) and blood oxygen saturation (SpO_2) or oxygenation index.
h. Drug infusion safety monitoring: Vital signs, including blood pressure (systolic and diastolic), pulse rate, blood oxygen saturation SpO_2 or oxygenation index, respiration rate and body temperature, will be measured before each dose (within 30 min) and 1 h ± 10 min, 6 h ± 1 h and 12 h ± 1 h after each dose during the treatment period.
i. Baseline assessments should be performed prior to dosing of study drug.
j. Safety hematology, blood chemistry, coagulation and urinalysis:

- Urinalysis: White blood cells (WBC), red blood cells (RBC), pH and protein;
- Hematology: WBC, RBC, hemoglobin (HGB), platelet (PLT) count, absolute lymphocytes, absolute neutrophils, absolute monocytes, absolute eosinophils, absolute basophils, hematocrit (HCT), mean corpuscular volume (MCV), mean corpuscular hemoglobin (MCH), and mean corpuscular hemoglobin concentration (MCHC);
- Blood chemistry: Liver function (total protein, albumin, total bilirubin [TBL], direct bilirubin, indirect bilirubin, alanine aminotransferase [ALT], aspartate aminotransferase [AST], alkaline phosphatase and gamma-glutamyl transferase); renal function (urea, creatinine, uric acid, cystatin C); lactate dehydrogenase, cardiac troponin, D-dimer and ferritin; blood lipids (triglyceride, high-density lipoprotein, low-density lipoprotein and total cholesterol); postprandial glucose; electrolytes (sodium, potassium, chloride and calcium);
- Coagulation: Prothrombin time, activated partial thromboplastin time, fibrinogen and thrombin time;

k. Test results obtained within 72 h prior to the first dose are acceptable.
l. PD endpoints: CRP, IL-2, IL-4, IL-6, INF-α, IL-8, IL-10, IL-12p70, IL-17, IL-1β, TNF-α and IFN-γ, as well as lymphocyte subsets CD3+, CD4+ and CD8+. Blood samples will be collected at the following time points, when available: pre-dose and on D1, D4, D7 (before the second dose), D8, D10 and D28 (optional). If the second dose is not administered, no sample will be collected on D7, D8 and D10.
m. If a subject is tested negative for nucleic acid before the first dose, no more nucleic acid testing is required during the study. For those who are still positive for nucleic acid before the first dose, nucleic acid testing will be performed during the study. If a negative nucleic acid test result is obtained, a re-test is required at least 24 hours later. If a subject is tested negative for nucleic acid before discharge (two consecutive negative results, at least 24 hours apart), no more nucleic acid testing is required at subsequent visits; if a subject does not achieve negative conversion before discharge, nucleic acid testing must continue at subsequent visits.

4. Study Objectives, Endpoints and Estimands

4.1 Study Objectives and Endpoints

Table 2 Study Objectives and Endpoints

**Primary Objective**	**Primary Endpoint**
To validate that Meplazumab is superior to placebo when added to standard of care in reducing all-cause mortality on D28 in patients with severe SARS-CoV-2 infection.	• All-cause mortality on D28
**Secondary Objective - Efficacy**	**Secondary Efficacy Endpoints**
To evaluate the efficacy of Meplazumab compared to placebo when added to the standard of care in increasing the discharge rate, reducing mortality, shortening the time to sustained clinical improvement, reducing the number of days with oxygen supplementation, mechanical ventilation, length of intensive care unit (ICU) stay and hospital stay, shortening the time to SARS-CoV-2 nucleic acid negative conversion, increasing the SARS-CoV-2 nucleic acid negative conversion rate, reducing SARS-CoV-2 viral load and improving the proportion of subjects with improvement in lung imaging in patients with severe SARS-CoV-2 infection	• Discharge rate on D28<br>• Mortality on D14 and D56<br>• Time to sustained clinical improvement (days, range: D0–D28)<br>• Duration of oxygen supplementation (days)<br>• Duration of mechanical ventilation (days)<br>• Incidence and duration of re-ventilation 24 hours after withdrawal (days)<br>• Length of stay in ICU and hospitalization time (days)<br>• Time to SARS-CoV-2 nucleic acid negative conversion<br>• Changes from baseline in SARS-CoV-2 nucleic acid negative conversion rate and SARS-CoV-2 viral load on D7, D14 and D28<br>• Proportion of subjects with improvement in lung imaging
**Secondary Objective - Pharmacodynamics**	**PD Evaluation**
To evaluate the changes from baseline in levels of cytokines and chemokines associated with inflammatory and immune states as well as lymphocyte subsets in patients with severe SARS-CoV-2 infection after dosing with Meplazumab	• Changes from D0 (pre-dose) in the levels of cytokines and chemokines associated with inflammatory and immune states on D1, D4, D7 (before the second dose), D8, D10 and D28, including CRP, IL-2, IL-4, IL-6, INF-α, IL-8, IL-10, IL-12p70, IL-17, IL-1β, TNF-α and IFN-γ<br>• Changes from D0 (pre-dose) in lymphocyte subsets CD3+, CD4+ and CD8+ on D1, D4, D7 (before the second dose), D8, D10 and D28
**Secondary Objective - Safety**	**Safety Endpoints**
To evaluate the safety of Meplazumab in patients with severe SARS-CoV-2 infection	• Incidence of adverse events (AEs) and serious adverse events (SAEs);<br>• Vital signs (blood pressure, pulse, blood oxygen saturation SpO_2 or oxygenation index, respiration, and body temperature);<br>• Laboratory tests (hematology, urinalysis, blood chemistry and coagulation);<br>• 12-lead ECG;<br>• Chest imaging.
**Secondary Objective - Immunogenicity**	**Immunogenicity Evaluation**
To evaluate the immunogenicity of Meplazumab in patients with severe SARS-CoV-2 infection	• Anti-drug antibody

4.2 Estimands

4.2.1 Primary Estimand

The primary estimand of this trial is the difference between the test group and control group in the all-cause mortality on D28 after treatment in patients with severe SARS-CoV-2 infection. Specifically:

Population: The target population of this trial is patients with severe SARS-CoV-2 infection.

Variable: Whether the subject dies on D28;

Treatment: Subjects in the test group will receive Meplazumab and standard of care (SoC), while subjects in the control group will receive placebo and SoC.

Intercurrent Events and Handling Strategies: The main intercurrent events and handling strategies in the study are defined as follows:

Table 2 Intercurrent Events of Primary Estimand and Handling Strategies

Intercurrent Events	Handling Strategies	Remarks
Early discontinuation of treatment due to poor efficacy	**Therapeutic Strategy**<br>Continue to collect and use data despite the intercurrent event	Reflect actual clinical condition
Early discontinuation of treatment due to adverse events	**Therapeutic Strategy**<br>Continue to collect and use data despite the intercurrent event	Reflect actual clinical condition
Concomitant use of prohibited medications/treatments affecting efficacy evaluation	**Therapeutic Strategy**<br>Continue to collect and use data despite the intercurrent event	Reflect actual clinical condition
Disease aggravation (progression to critical illness)	**Therapeutic Strategy**<br>Continue to collect and use data despite the intercurrent event	

Population-level Summary: Difference between the test group and control group in all-cause mortality on D28.

4.2.2 Secondary Estimand

Population: The target population of this trial is patients with severe SARS-CoV-2 infection. Refer to Section 4 "Study Population" for specific inclusion requirements. The target population of estimand 6 is severe SARS-CoV-2 infected patients with mechanical ventilation withdrawal during the trial; the target population of estimands 8–10 is severe SARS-CoV-2 infected patients with positive baseline nucleic acid testing.

Variable:

1) Whether the subject is discharged on D28;
2) Whether the subject dies on D14 and D56;

3) Time to sustained clinical improvement in subjects (days; range: D0–D28); sustained clinical improvement is defined as patient discharge (discharge criteria: body temperature returning to normal for at least 3 days, significant improvement in respiratory symptoms without oxygen support, and two consecutive negative nucleic acid test results at least 24 hours apart) or a reduction of at least 2 points on the Ordinal Scale for Clinical Improvement;
4) Duration of oxygen supplementation for the subject (days);
5) Duration of mechanical ventilation for the subject (days);
6) Whether mechanical ventilation is provided again 24 hours after withdrawal of mechanical ventilation for the subject, and the duration of the second mechanical ventilation (days);
7) Length of stay in ICU (days) and hospitalization time of the subject (days);
8) Time to negative nucleic acid conversion (days)
9) Negative conversion rate of SARS-CoV-2 nucleic acid test on D7, D14, and D28;
10) Change from baseline in SARS-CoV-2 viral load;
11) Whether the subject's lung imaging is improved; Improvement in lung imaging is defined as any of the following changes from baseline in lung imaging during the study: 1) reduction in lesion diameter; 2) decrease in the number of lesions; 3) decrease in the number of lung lobes involved by lesions

Treatment: Subjects in the test group will receive Meplazumab and standard of care (SoC), while subjects in the control group will receive placebo and SoC. Refer to Section 6 "Dosage and Method of Administration" for the specific treatment regimen.

Intercurrent Events and Handling Strategies: The main intercurrent events and handling strategies in the study are defined as follows:

Table 3 Intercurrent Events of Secondary Estimand and Handling Strategies

Intercurrent Events	Handling Strategies	Remarks
Early discontinuation of treatment due to poor efficacy	**Therapeutic Strategy**<br>Continue to collect and use data despite the intercurrent event	Reflect actual clinical condition
Early discontinuation of treatment due to adverse events	**Therapeutic Strategy**<br>Continue to collect and use data despite the intercurrent event	Reflect actual clinical condition
Concomitant use of prohibited medications/treatments affecting efficacy evaluation	**Therapeutic Strategy**<br>Continue to collect and use data despite the intercurrent event	Reflect actual clinical condition
Death	Secondary estimand 1: Composite strategy, handle as "not	

Intercurrent Events	Handling Strategies	Remarks
	discharged".<br>Secondary estimands 3 and 8: Hypothetical strategy, perform "censoring" according to the time of the last follow-up.<br>Secondary estimands 4–7: Therapeutic strategy, handle according to the actual observation days.<br>Secondary estimand 9: Composite strategy, handle as "no negative conversion".<br>Secondary estimand 10: Therapeutic strategy, handle according to the actual observed value.<br>Secondary estimand 11: Composite strategy, handle as "not improved".	

Population-level Summary:

1) Difference between the test group and control group in discharge rate on D28;
2) Difference between the test group and control group in mortality on D14 and D56;
3) Hazard ratio of time to sustained clinical improvement between the test group and control group;
4) Difference between the test group and control group in days of oxygen supplementation;
5) Difference between the test group and control group in days of mechanical ventilation;
6) Difference between the test group and control group in the incidence of the second mechanical ventilation 24 hours after withdrawal of mechanical ventilation and the duration of the second mechanical ventilation;
7) Difference between the test group and control group in ICU days and hospitalization days;
8) Hazard ratio of time to SARS-CoV-2 nucleic acid negative conversion between the test group and control group;
9) Difference between the test group and control group in SARS-CoV-2 nucleic acid negative conversion rate on D7, D14, and D28;
10) Difference between the test group and control group in the change from baseline in SARS-CoV-2 viral load on D7, D14, and D28;
11) Difference between the test group and control group in the proportion of subjects with improvement in lung imaging.

5. Analysis Sets

The primary estimand will be analyzed mainly based on the Full Analysis Set (FAS). FAS includes all randomized subjects who receive at least one dose of the investigational product, as per the intention-to-treat principle. In addition, it will be analyzed based on the Per-Protocol Set (PPS), which will exclude subjects who do not complete treatment according to the protocol or have major protocol violations from the FAS.

Secondary estimands will be analyzed based on the FAS and PPS.

Safety analysis will be conducted based on the Safety Set (SS), which includes all randomized subjects who receive at least one dose of the investigational product.

All the above analysis sets will be jointly discussed and decided by the sponsor, principal investigator, statistician, and data manager during the data blinding review meeting prior to database lock.

6. Statistical Analysis Methods

6.1 General Considerations

6.1.1 General Analytical Methods

- **Descriptive Statistics**

Unless otherwise specified, the following descriptive statistical summaries will be presented by variable type:

- Continuous variables will be summarized by mean, standard deviation, median, quartiles (Q1, Q3), minimum and maximum.
- Categorical or ordinal variables will be summarized by frequency and percentage, with the percentage calculated using the non-null participant number in the corresponding analysis set as the denominator. Two-sided 95% CIs for the percentages will be calculated using the Clopper-Pearson method.

- **Decimal Places**

Unless otherwise specified, the number of decimal places in the SAP will processed be as follows:

- The minimum and maximum values are consistent with the maximum decimal places of the original data;
- The median, Q1, Q3, mean, geometric mean, standard deviation, and 2-sided 95% CI have 1 more decimal place than the maximum decimal place of the primary data;
- Percent or rate values are rounded to 2 decimal places;
- If the P value ≥ 0.0001, it will be rounded to 4 decimal places; if the P value < 0.0001, it will be reported as "< 0.0001";
- All statistical test data will be rounded to 3 decimal places;
- Derived data will be rounded to 2 decimal places.

6.1.2 Relevant Definitions and Derived Rules

- **Baseline**

Unless otherwise specified, the "baseline" in this study is defined as the last non-null test value before the first dose.

- **Treatment-Emergent Adverse Events**

Treatment-emergent adverse events (TEAEs) are defined as AEs that occur after the first dose (including the day of the first dose) or worsen after dosing. TEAEs are judged based on the following rules:

  - An AE will be counted as a TEAE if it occurs after (at) the start time of treatment with an investigational product;
  - An AE will be counted as a TEAE if it occurs before the start time of treatment with an investigational product, but worsens in severity after the treatment;
  - An AE will be counted as a TEAE if its onset time or the start time of treatment with an investigational drug is missing, rendering it impossible to judge the temporal relationship between the onset time of AE and the start time of treatment with the investigational drug;
  - An AE will be counted as a TEAE if it occurs before the start time of treatment with an investigational product but the severity is missing, rendering it impossible to judge if the severity worsens after the treatment.

- **TEAE Causality**
  - "Related to the investigational product" suggests that the relationship between AEs and the investigational product is "related", "possibly related" or "probably related".
  - "Unrelated to the investigational product" suggests that the relationship between AEs and the investigational AEs is "unrelated" or "unlikely related".

6.1.3 Statistical Analysis Window

For post-baseline visits, statistical analysis will be performed by visit time points scheduled in the protocol. Unscheduled visits should be considered when statistical analysis is performed on the most severe results (such as laboratory tests) after the first dose. All test results (at scheduled and unscheduled visit time points per protocol) will be presented in listings.

6.1.4 Analysis Software

All statistical analyses will be performed using the statistical software SAS 9.4 or above version.

6.1.5 Tables and Listings

- **Tables**

The obtained data are typically summarized by group (Meprazolumab group and placebo group), with each group presented in separate columns.

- **Listings**

Unless otherwise specified, all listings will include groups and subject numbers, and priority will be given to the data in the Study Data Tabulation Model (SDTM). Listings will be generally sorted by group, subject number, visit time, or other relevant time (e.g., AE occurrence time).

6.2 Enrollment and Completion of the Trial

Analysis will be made on the subjects who are screened, have failed in screening (including the reasons), are randomized, have completed the trial, withdrawn early form the trial (including the reasons), and are included in each analysis set. The listing of subjects who have failed screening, withdrawn early and are not included in each analysis set will be presented respectively. Prepare a flow chart of subject disposition.

6.3 Demographic and Baseline Characteristics

The following demographic data and other baseline indicators will be summarized descriptively:

- Demographic data (including age, gender, nationality, height, weight and BMI)
- Clinical characteristics at screening (including smoking history, COVID-19 history, type of COVID-19, SARS-CoV-2 test result and duration of disease); duration of disease (days) = date of signing ICF - date of COVID-19 diagnosis + 1.
- Baseline vital signs;
- Baseline physical examination.

Surgical history, prior or present medical history and prior non-drug therapy will be coded as per MedDRA (27.0 or the latest version) and summarized by System Organ Classification (SOC) and Preferred Term (PT).

Prior medications will be coded as per WHODrug (2024-Mar-1 or higher version) and summarized descriptively by ATC2 and drug standard name.

Demographic data, surgical history, prior or present medical history, prior non-drug therapies and prior medications of subjects will be listed.

The above analyses will be based on the FAS.

6.4 Protocol Violations/Deviations

The number of events, number of subjects and incidence of protocol violations/deviations will be statistically described for the Meplazumab group and placebo group respectively, and a list of protocol violations/deviations will be presented.

Protocol violations/deviations will be analyzed based on randomized subjects.

6.5 Concomitant Medication/Non-Drug Therapy

Concurrent medications recorded in clinical report forms (CRFs) will be coded as per WHODrug (2024-Mar-1 or higher version). Concurrent medications will be summarized descriptively by ATCII and drug standard name. A list of subjects' concurrent medications will be presented.

Concurrent non-drug therapies will be coded as per MedDRA (27.0 or higher version) and statistically described for the number of events, number of subjects and incidence by SOC and PT. A list of subjects' concurrent non-drug therapies will be presented.

The concomitant medication and non-drug therapy analysis will be based on SS.

6.6 Study Hypothesis

Hypothesis testing will be performed on all-cause mortality on D28 postdose in subjects with severe SARS-CoV-2 infection, i.e.,

Null hypothesis H_0: $\pi_C \le \pi_T$, Alternative hypothesis H_1:$\pi_C > \pi_T$

Where, π_T,π_C represent the all-cause mortality in the Meplazumab group and placebo group, respectively, with one-sided $\alpha=0.025$ used for the test level.

6.7 Efficacy Evaluation

Unless otherwise specified, the following efficacy analyses will be performed based on FAS and PPS.

6.7.1 Analysis of Primary Estimand

For the Meplazumab and placebo groups, the all-cause mortality on D28 post-dose will be calculated. The Clopper-Pearson method will be used to calculate its two-sided 95% confidence interval (CI). CMH Chi-square test will be used for the statistical test of inter-group difference, with age (< 65 years or ≥ 65 years) as the stratification factor. Meanwhile, the difference in the all-cause mortality on D28 post-dose (placebo group-Meplazumab group) and its two-sided 95% CI will be calculated using the CMH method, with age (< 65 years or ≥65 years) as the stratification factor. Also, Miettinen & Nurminen's method without considering stratification factors will be used to calculate the difference between the groups (placebo group-Meplazumab group) and its two-sided 95% confidence interval (CI). The chi-square test/Fisher's exact test without considering stratification factors will be used for statistical testing of the difference between the two groups.

Logistic regression model will be fitted to calculate the odds ratio (OR) between the Meplazumab group and the placebo group as well as the 95% CI. The model will include all-cause mortality on

D28 postdose as the dependent variable and group and age group (< 65 years vs. ≥ 65 years) as fixed effects.

Also, based on FAS, TPA will be used to perform sensitivity analysis on missing data and evaluate the impact of missing data on the robustness of test results.

Supplementary analysis: Based on FAS, efficacy evaluation will be performed with intercurrent events "early discontinuation of treatment due to poor efficacy", "concomitant use of prohibited medications/treatments affecting efficacy evaluation", and "disease aggravation" analyzed as "death" according to the composite variable strategy.

6.7.2 Analysis of Secondary Estimand

(1) Discharge Rate on D28 after the First Dose

The discharge rate on D28 after the first dose will be calculated for the Meplazumab group and placebo group, respectively. The two-sided 95% CI will be calculated using the Clopper-Pearson method. The difference in discharge rate on D28 postdose (placebo group-Meplazumab group) and the two-sided 95% CI will be calculated using the CMH method considering stratification factor (age group (< 65 years vs. ≥ 65 years)).

(2) All-cause Mortality on D14 and D56 after the First Dose

The all-cause mortality on D14 and D56 after the first dose will be calculated for the Meplazumab group and placebo group, respectively. The two-sided 95% CI will be calculated using the Clopper-Pearson method. The difference in all-cause mortality on D14 and D56 postdose (placebo group-Meplazumab group) and the two-sided 95% CI will be calculated using the CMH method considering stratification factor (age group (< 65 years vs. ≥ 65 years)).

(3) Time to Sustained Clinical Improvement (Days)

Kaplan-Meier method will be used to calculate the recovery rates at different time points and the lower quartile, median, and upper quartile of time to sustained clinical improvement and their two-sided 95% CIs (Greenwood method: log-log transformation) in the Meplazumab group and placebo group, respectively, and stratified log-rank test will be used for statistical testing of differences between the groups. Also, the Cox model with the time to sustained clinical improvement as the dependent variable, and group and stratification factor age group (< 65 years old vs. ≥ 65 years old) as the fixed effects will be used to estimate the hazard ratio between the groups (Meplazumab group-placebo group) as well as the two-sided 95% CI. Kaplan-Meier curves will be plotted for the time to sustained clinical improvement in the Meplazumab group and placebo group.

(4) Duration of Oxygen Supplementation (Days)

Days of oxygen supplementation (including cumulative days of oxygen supplementation, days of low-flow oxygen supplementation and days of high-flow oxygen supplementation) in the Meplazumab group and placebo group will be statistically described, respectively, and the difference between the groups (placebo group-Meplazumab group) and its two-sided 95% CI will be calculated.

It can be calculated by the following formula:

Duration of oxygen supplementation (days) = end date - start date + 1.

(5) Duration of Mechanical Ventilation (Days)

The cumulative days of mechanical ventilation in the Meplazumab treatment group and placebo group will be statistically described, respectively, and the difference between the groups (placebo group-Meplazumab group) and its two-sided 95% CI will be calculated.

It can be calculated by the following formula:

Duration of mechanical ventilation (days) = end date - start date + 1.

(6) Incidence and Duration of Re-ventilation 24 Hours After Withdrawal (Days)

The incidence and duration of the second mechanical ventilation 24 hours after withdrawal of mechanical ventilation in the Meplazumab group and placebo group will be statistically described, respectively, and the difference between the groups (placebo group-Meplazumab group) and its two-sided 95% CI will be calculated.

It can be calculated by the following formula:

Duration of re-ventilation 24 hours after withdrawal (days) = end date of re-ventilation - start date of re-ventilation + 1.

(7) Length of Stay in ICU and Hospitalization Time (Days)

The ICU time and hospitalization time in the Meplazumab group and placebo group will be statistically described, respectively, and the difference between the groups (placebo group-Meplazumab group) and its two-sided 95% CI will be calculated.

It can be calculated by the following formula:

Length of ICU stay (days) = date of discharge - date of admission + 1.

Length of hospital stay (days) = date of discharge - date of admission + 1.

(8) Time to Negative Nucleic Acid Conversion (Days)

Kaplan-Meier method will be used to calculate the negative conversion rates at different time points and the lower quartile, median, and upper quartile of time to negative conversion and their two-sided 95% CIs (Greenwood method: log-log transformation) in the Meplazumab group and placebo group, respectively, and stratified log-rank test will be used for statistical testing of differences between the

groups. Also, the Cox model with the time to negative conversion as the dependent variable, and group and stratification factor age group (< 65 years old vs. ≥ 65 years old) as the fixed effects will be used to estimate the hazard ratio between the groups (Meplazumab group-placebo group) as well as the two-sided 95% CI. Kaplan-Meier curves will be plotted for the time to negative conversion in the Meplazumab group and placebo group.

It can be calculated by the following formula:

Time to nucleic acid negative conversion (days) = date of SARS-CoV-2 nucleic acid negative conversion - date of first dosing + 1.

SARS-CoV-2 nucleic acid negative conversion is defined as two consecutive negative nucleic acid test results at least 24 hours apart. The time to nucleic acid negative conversion is defined as the sampling time of the first of these two consecutive negative nucleic acid test results.

(9) SARS-CoV-2 Nucleic Acid Negative Conversion Rate on D7, D14, and D28

The SARS-CoV-2 nucleic acid negative conversion rate on D7, D14 and D28 in the Meplazumab group and placebo group will be statistically described, respectively, and the difference between the groups (placebo group-Meplazumab group) and its two-sided 95% CI will be calculated.

(10) Change from Baseline in SARS-CoV-2 Viral Load on D7, D14 and D28

The changes from baseline in SARS-CoV-2 viral load (log 10 copies/mL) on D7, D14 and D28 in the Meplazumab group and placebo group will be statistically described, respectively, and the difference between the groups (placebo group-Meplazumab group) and its two-sided 95% CI will be calculated.

(11) Lung Imaging Improvement Rate

The lung imaging improvement rate on D7 and D14 after the first dose in the Meplazumab group and placebo group will be calculated, respectively, the 95% CI will be calculated by the Clopper-Pearson method, and the difference between the groups (placebo group-Meplazumab group) and its two-sided 95% CI will be calculated.

6.8 PD Analysis

The following analyses will be based on the FAS.

(1) Changes from Baseline in Cytokines and Chemokines

Cytokines and chemokines on D1, D4, D7 (before the second dose), D8, D10 and D28 after the first dose and their changes from baseline in the Meplazumab group and placebo group will be statistically described, respectively.

(2) Change from Baseline in Lymphocyte Subsets

Lymphocyte subsets on D1, D4, D7 (before the second dose), D8, D10 and D28 after the first dose and their changes from baseline in the Meplazumab group and placebo group will be statistically described, respectively.

6.9 Immunogenicity Analysis

The positive rate and cumulative positive rate of anti-drug antibodies (ADAs) pre-dose and post-dose will be calculated for the Meplazumab group and placebo group respectively; their two-sided 95% confidence intervals (CIs) will be calculated using the Clopper-Pearson method; the geometric mean titer (GMT) will be statistically described for subjects tested positive for ADAs at each time point.

The above analyses will be based on the FAS.

6.10 Safety Analysis

6.10.1 Subject Exposure

The study time, theoretical total dose, actual total dose and compliance will be statistically described for each group.

Where, Study duration (months) = ((study end date – ICF signing date) + 1)/30.4375.

For subjects who withdraw early from the study, the study duration is calculated using the date of withdrawal as the end date.

Compliance (%) = actual total dose/theoretical total dose * 100%.

The actual total dose is the sum of the doses actually administered across all visits. The theoretical total dose is the sum of the planned doses across all visits.

6.10.2 Adverse Events (AEs)

AEs will be coded as per MedDRA (27.0 or the latest version). Classified statistics will be performed by SOC and PT. In this trial, the treatment-emergent adverse events (TEAEs) will mainly be statistically analyzed; pretreatment AEs will be presented in a list. Unless otherwise specified, adverse events below are TEAEs.

The number of events, number of subjects and incidence of the following AEs will be calculated for subjects in the Meplazumab group and placebo group, respectively:

- **All TEAEs;**
  - Treatment-related AEs (TRAEs);
- **AEs of different severity;**
  - TRAEs of different severity;
- **AEs with incidence ≥ 1% in any group;**
  - TRAEs with incidence ≥ 1% in any group;

- **AEs with incidence ≥ 5% in any group;**
  - TRAEs with incidence ≥ 5% in any group;
- **Serious Adverse Events (SAEs);**
  - Treatment-related SAEs;
- **Significant AEs;**
  - Treatment-related significant AEs;
- **AEs leading to withdrawal from the trial;**
  - TRAEs leading to withdrawal from the trial;
- **AEs leading to death;**
  - TRAEs leading to death;
- **AEs leading to discontinuation;**
  - TRAEs leading to discontinuation;
- **AEs leading to dose reduction;**
  - TRAEs leading to dose reduction;
- **AEs leading to dose interruption;**
  - TRAEs leading to dose interruption

The severity and correlation of adverse events will be tabulated.

When calculating the incidence of AEs in each group, count multiple occurrences of the same adverse event in a single subject as one event. When calculating the number of cases, count multiple occurrences of the same adverse event in a single subject as multiple events. For the analysis of the severity and drug correlation of adverse events, if a subject experiences the same adverse event multiple times, the most severe occurrence or the one most related to the drug should be used for analysis.

Listings of AEs, AEs leading to withdrawal and SAEs will be presented, respectively.

6.10.3 Laboratory Tests

Baseline values of laboratory test indicators (including hematology, blood chemistry, urinalysis, and coagulation function) and changes from baseline to the worst grade will be described in a shift table (based on the normal range and investigator's judgment of clinical significance) by Meplazumab group and placebo group.

The test results and abnormalities of all laboratory tests during the study will be listed.

6.10.4 Physical Examination

All physical examination results during the study will be listed.

6.10.5 Vital Signs

The vital signs in the Meplazumab group and placebo group will be statistically described, respectively.

Baseline values of vital signs examination indicators and changes from baseline to the worst grade will be described in a shift table (based on the normal range and investigator's judgment of clinical significance) by Meplazumab group and placebo group.

The test results and abnormalities of vital signs during the study will be listed.

6.10.6 12-Lead ECG

Baseline values of 12-ECG indicators and changes from baseline to the worst grade will be described in a shift table (based on the normal range and investigator's judgment of clinical significance) by Meplazumab group and placebo group.

The test results and abnormalities of all 12-lead ECG examinations during the study will be listed.

6.11 Interim Analysis

Interim analysis will not be performed in this study.

6.12 Subgroup Analysis

Subgroup analyses of the primary efficacy endpoint will be performed by age (<65 years vs. ≥65 years), gender, concomitant use of antiviral drugs (Yes vs. No), BMI ≥30 (kg/m^2) (Yes vs. No), underlying disease (Yes vs. No), SARS-CoV-2 vaccination status (Yes vs. No) and smoking (Yes vs. No), and forest plots will be provided.

6.13 Multiplicity Handling

There is only one primary endpoint for this trial; therefore, type I error correction is not required.

6.14 Handling of Dropouts or Missing Data

The handling and censoring rules for missing data of primary and secondary efficacy endpoints and safety endpoints in this study are specified as follows. In addition, a sensitivity analysis will be performed using TPA to evaluate the impact of missing values on the primary estimand. For the TPA, the possible values of missing data for all-cause mortality on D28 post-dose in the placebo group will be enumerated to find a tipping point that reverses the analysis conclusions of all-cause mortality on D28 post-dose; a smaller tipping point correlates with less impact of missing data on conclusions, indicating the conclusions are more robust. In addition, TPA results will be plotted to offer a visual basis for judgment.

Endpoints	Indicators	Handling Methods
Primary Efficacy Endpoints	All-cause mortality on D28	Imputation based on the status of last follow-up visit
	Discharge Rate on D28 after the First Dose	Imputation based on the status of last follow-up visit
	Time to Sustained Clinical Improvement	Censoring based on D28 post-dose
Secondary Efficacy Endpoints	Duration of oxygen supplementation (days), duration of mechanical ventilation (days), duration of re-ventilation 24 hours after withdrawal (days), length of ICU stay (days) and length of hospital stay (days)	No imputation
	Time to Negative Nucleic Acid Conversion (Days)	Censoring based on the time of last follow-up
	SARS-CoV-2 Nucleic Acid Negative Conversion Rate	No imputation
	Change from baseline in SARS-CoV-2 viral load	No imputation
	Lung Imaging Improvement Rate	No imputation
Safety Endpoints	Safety indicators	No imputation

Version History

Version	Version Date	Prepared by	Changes
V1.0	April 09, 2024	Ye Tian	Initial Version
